# Supplementary material for: GA4GH phenopacket-driven characterization of genotype-phenotype correlations in Mendelian disorders
Source: Am J Hum Genet. 2025 Dec 23;113(1):57–70. doi: 10.1016/j.ajhg.2025.12.001 (PMC12824607; doi:10.1016/j.ajhg.2025.12.001)
Supplement: Document S1. Figures S1–S88; Tables S1 and S3–S11 [file mmc1.pdf]

The American Journal of Human Genetics, Volume *113*

Supplemental information

GA4GH phenopacket-driven characterization of genotype-phenotype correlations in Mendelian disorders

Lauren Rekerle, Daniel Danis, Filip Rehburg, Adam S.L. Graefe, Viktor Bily, Andrés Caballero-Oteyza, Pilar Cacheiro, Leonardo Chimirri, Jessica X. Chong, Evan Connelly, Bert B.A. de Vries, Alexander J.M. Dingemans, Michael H. Duyzend, Tomas Freiberger, Petra Gehle, Tudor Groza, Peter Hansen, Julius O.B. Jacobsen, Adam Klocperk, Markus S. Ladewig, Michael I. Love, Allison J. Marcello, Alexander Mordhorst, Monica C. Munoz-Torres, Justin Reese, Catharina Schuetz, Damian Smedley, Timmy Strauss, Ondrej Vladyka, David Zocche, Sylvia Thun, Christopher J. Mungall, Melissa A. Haendel, and Peter N. Robinson

ABCB7

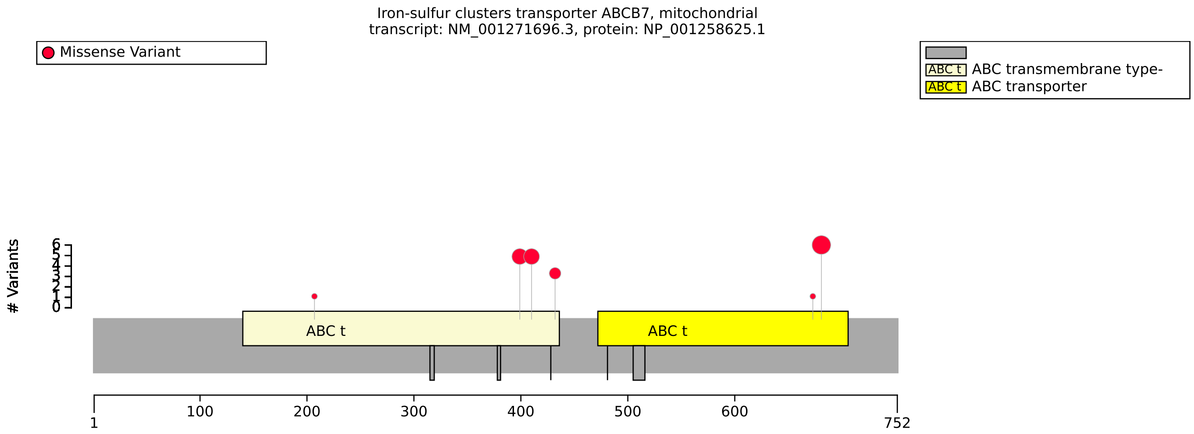

(a) Distribution of variants in *ABCB7*

Genotype (A)	Genotype (B)	total tests performed	significant results
ABC transmembrane type-1	Other region	12	0
p.Gly682Ser	Other variant	10	0

(b) Fisher Exact Test performed to compare HPO annotation frequency with respect to variants located in the ABC transmembrane type-1 region and p.Gly682Ser.

Figure S1: The cohort comprised 18 individuals (0 females, 18 males). A total of 52 HPO terms were used to annotate the cohort. Disease diagnosis: Anemia, sideroblastic, and spinocerebellar ataxia (OMIM:301310). No statistically significant results identified. A total of 18 unique variant alleles were found in *ABCB7* (transcript: NM_001271696.3, protein id: NP_001258625.1).

ACADM

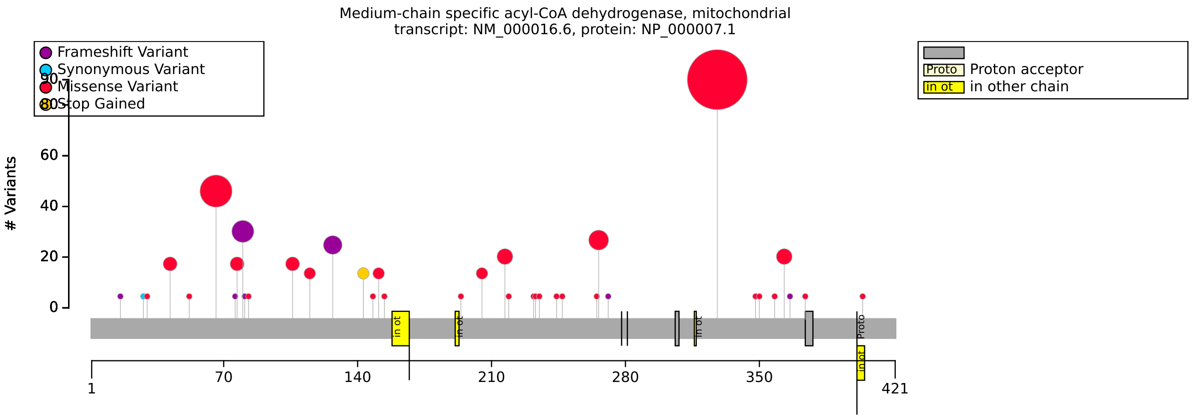

(a) Distribution of variants in *ACADM*

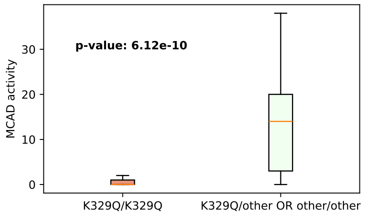

(b) Lys329Glu: t test for MCAD Activity (% normal; LOINC:74892-1): p=6.12×10^{-10}.

Description	Variable	Genotype (A)	Genotype (B)	p-value	ref
Value of MCAD Activity% [LOINC:74892-1]	LOINC:74892-1	K329Q/K329Q	K329Q/other OR other/other	6.12×10^{-10}	[1]

(c) t-test to compare K329Q/K329Q and K329Q/other OR other/other with respect to LOINC:74892-1. Mean MCAD activity for K329/K329: 0.52%, and for K329/other or other/other: 13.23%

Description	Variable	Genotype (A)	Genotype (B)	p-value	xrefs
Value of MCAD Activity% [LOINC:74892-1]	LOINC:74892-1	Y67H/Y67H OR Y67H/other	other/other	2.01×10^{-5}	[1]

(d) t-test to compare Y67H/Y67H OR Y67H/other and other/other with respect to LOINC:74892-1. Mean MCAD activity for Y67H/Y67H: 18.60%, and for Y67H/other or other/other: 7.68%

Figure S2: The cohort comprised 115 individuals (0 females, 0 males, 115 with unknown sex). The cohort had data about medium chain Acyl-CoA dehydrogenase (MCAD), expressed as percentage of normal. The variant c.985G>A (p.Lys329Glu) is known to be severe, and the variant c.199T>C (p.Tyr67His) is known to be mild [1]. Disease diagnosis: Acyl-CoA dehydrogenase, medium chain, deficiency of (OMIM:201450). No statistically significant results identified. A total of 47 unique variant alleles were found in *ACADM* (transcript: `NM_000016.6`, protein id: `NP_000007.1`).

ACBD6

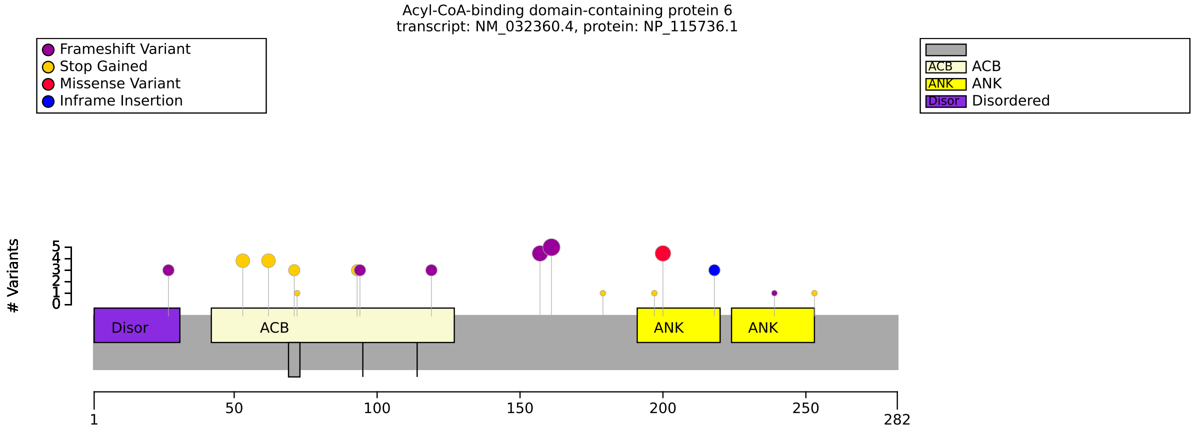

(a) Distribution of variants in *ACBD6*

Genotype (A)	Genotype (B)	total tests performed	significant results
missense/missense OR missense/other	other/other	99	0

(b) Fisher Exact Test performed to compare HPO annotation frequency with respect to missense/missense OR missense/other and other/other.

Genotype (A)	Genotype (B)	total tests performed	significant results
ACB/ACB OR ACB/other	other/other	99	0

(c) Fisher Exact Test performed to compare HPO annotation frequency with respect to ACB/ACB OR ACB/other and other/other.

Genotype (A)	Genotype (B)	total tests performed	significant results
FEMALE	MALE	99	0

(d) Fisher Exact Test performed to compare HPO annotation frequency with respect to FEMALE and MALE.

Figure S3: The cohort comprised 45 individuals (22 females, 23 males). A total of 79 HPO terms were used to annotate the cohort. Disease diagnosis: Neurodevelopmental disorder with progressive movement abnormalities (OMIM:620785). No statistically significant results identified. A total of 20 unique variant alleles were found in *ACBD6* (transcript: `NM_032360.4`, protein id: `NP_115736.1`).

AIRE

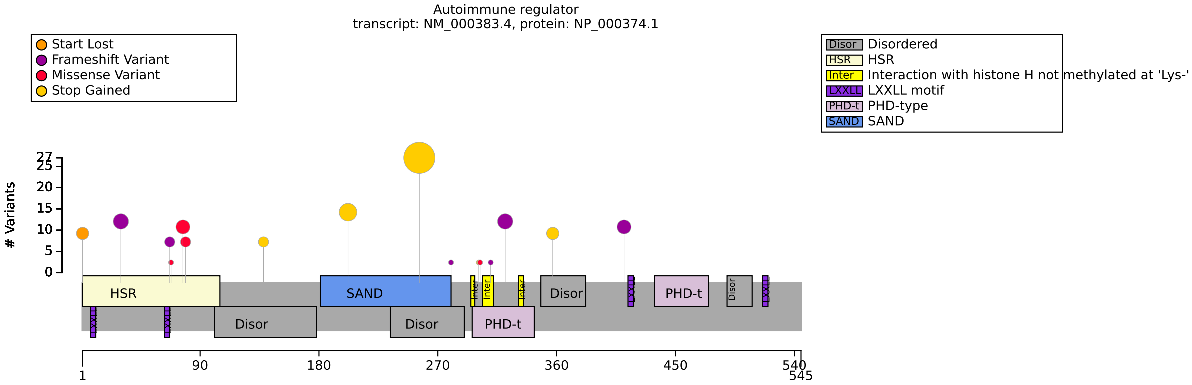

(a) Distribution of variants in *AIRE*

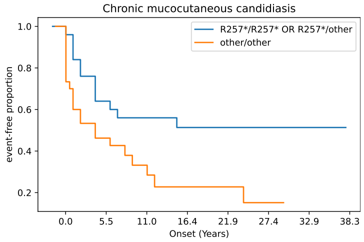

(b) log-rank p-value for Chronic mucocutaneous candidiasis (HP:0002728) and R357*/R357* or R357*/other versus other/other 0.0192

Genotype (A)	Genotype (B)	total tests performed	significant results
R257*/R257* OR R257*/other	other/other	18	0

(c) Fisher Exact Test performed to compare HPO annotation frequency with respect to R257*/R257* OR R257*/other and other/other.

Description	Variable	Genotype (A)	Genotype (B)	p-value	xrefs
Survival analysis: Chronic mucocutaneous candidiasis	Onset of HP:0002728	R257*/R257* OR R257*/other	other/other	0.019	[2]

(d) Onset of Chronic mucocutaneous candidiasis (HP:0002728) to compare R257*/R257* OR R257*/other and other/other with respect to Onset of HP:0002728.

Figure S4: The cohort comprised 58 individuals (31 females, 27 males). 2 of these individuals were reported to be deceased. A total of 47 HPO terms were used to annotate the cohort. Disease diagnosis: Autoimmune polyendocrinopathy syndrome , type I, with or without reversible metaphyseal dysplasia (OMIM:240300). A higher prevalence of chronic mucocutanous candidiasis with the variant Arg357Ter than with other variants was reported previously [2]. We did not identify a significant difference in prevalence A total of 18 unique variant alleles were found in *AIRE* (transcript: `NM_000383.4`, protein id: `NP_000374.1`).

ANKRD11

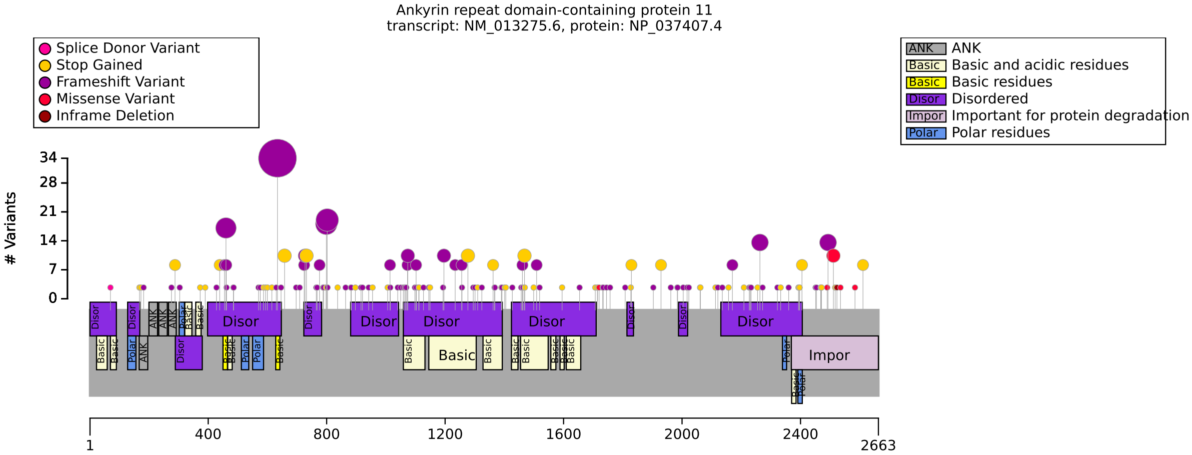

(a) Distribution of variants in *ANKRD11*

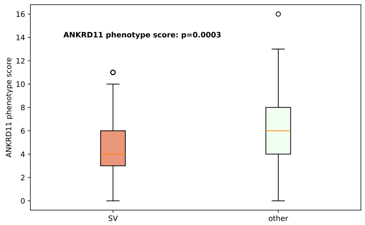

(b) ANKRD11 phenotypical score. Mean for structural variants: 4.62, and for other variants: 5.94

Genotype (A)	Genotype (B)	total tests performed	significant results
SV	other	23	0
exon 9	other	23	0
c.1903_1907del	other	23	0

(c) Fisher Exact Test performed to compare HPO annotation frequency with respect to SV, exon 9, and c.1903_1907del.

Description	Variable	Genotype (A)	Genotype (B)	p-value	xrefs
ANKRD11 phenotype score	HPO group count	SV	other	2.64×10^{-4}	[3]
ANKRD11 phenotype score	HPO group count	FEMALE	MALE	0.0075	-

(d) AKKRD11 phenotype score for structural variants: 4.62; other variants: 5.94. male/female comparison: Female 5.28, Male: 6.15.

Figure S5: The cohort comprised 337 individuals (143 females, 175 males, 19 with unknown sex). A total of 48 HPO terms were used to annotate the cohort. Disease diagnosis: KBG syndrome (OMIM:148050). Validated results on ANKRD11 phenotypical score. Other results did not survive multiple testing correction. Awamleh et al. (2023) stated that no significant DNAm differences based on sex at the identified KBGS-specific signature sites [4]. We are aware of no other publication that mentions sex-specific differences in KBGS. A total of 163 unique variant alleles were found in *ANKRD11* (transcript: `NM_013275.6`, protein id: `NP_037407.4`).

ASPM

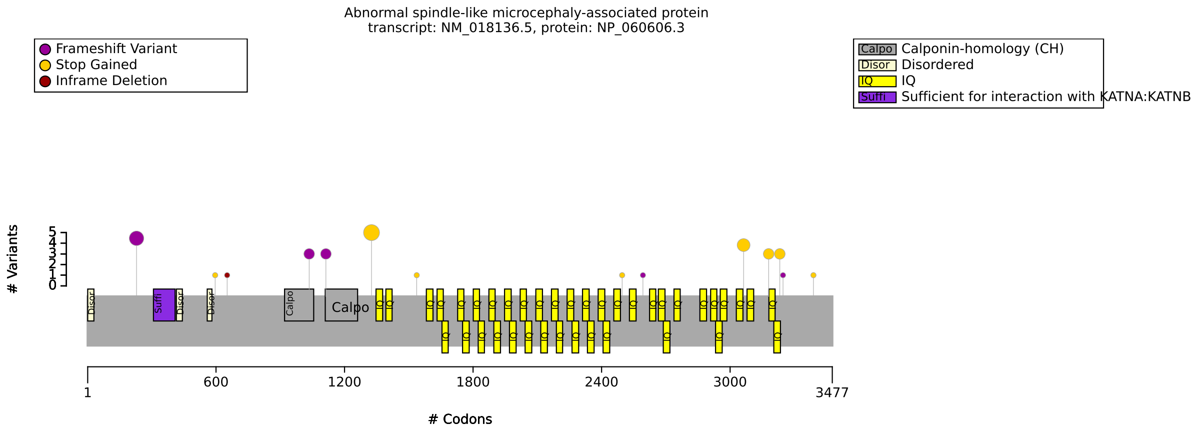

(a) Distribution of variants in *ASPM*

Genotype (A)	Genotype (B)	total tests performed	significant results
N Term/N Term OR N Term/other	other/other	14	0
FEMALE	MALE	14	0

(b) Fisher Exact Test performed to compare HPO annotation frequency with respect to N Term/N Term OR N Term/other and other/other, as well as male/female comparison.

Figure S6: The cohort comprised 22 individuals (14 females, 8 males). A total of 17 HPO terms were used to annotate the cohort. Disease diagnosis: Microcephaly 5, primary, autosomal recessive (OMIM:608716). No statistically significant results identified. A total of 15 unique variant alleles were found in *ASPM* (transcript: `NM_018136.5`, protein id: `NP_060606.3`).

ATP6V0C

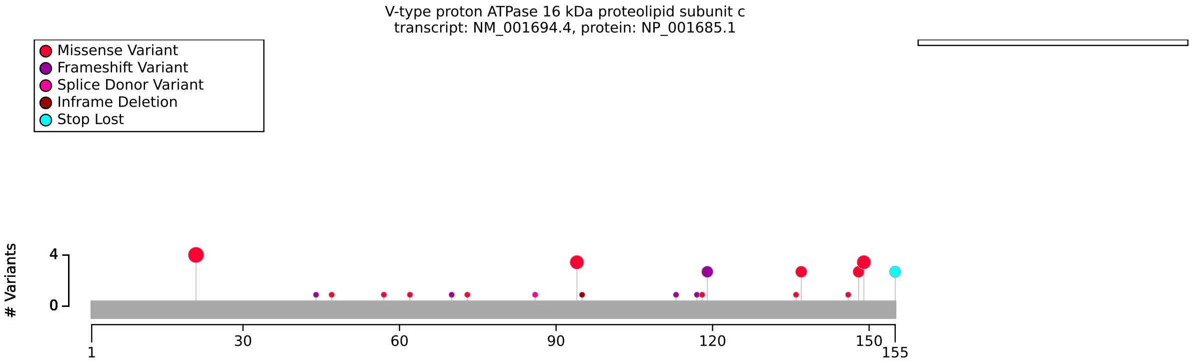

(a) Distribution of variants in *ATP6V0C*

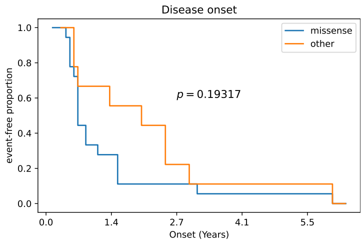

(b) Disease onset for *ATP6V0C* missense vs. other variants.

Genotype (A)	Genotype (B)	total tests performed	significant results
1-100	100+	80	0
missense	other	80	0
FEMALE	MALE	80	0

(c) Fisher Exact Test performed to compare HPO annotation frequency with respect to genotypes.

Description	Variable	Genotype (A)	Genotype (B)	p-value	xrefs
Compute time until OMIM:620465 onset	Onset of OMIM:620465	missense	other	0.193	-

(d) Onset of OMIM:620465 to compare missense and other with respect to Onset of OMIM:620465.

Figure S7: The cohort comprised 31 individuals (12 females, 19 males). A total of 83 HPO terms were used to annotate the cohort. Disease diagnosis: Epilepsy, early-onset, 3, with or without developmental delay (OMIM:620465). to do. A total of 23 unique variant alleles were found in *ATP6V0C* (transcript: `NM_001694.4`, protein id: `NP_001685.1`).

ATP13A2

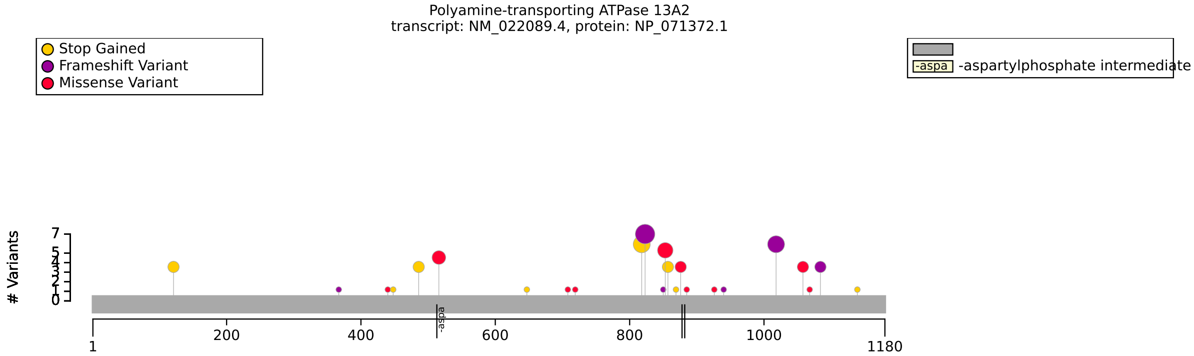

(a) Distribution of variants in *ATP13A2*

HPO term	OMIM:606693	OMIM:617225	p-value	adj. p-value
Parkinsonism [HP:0001300]	28/28 (100%)	3/11 (27%)	2.68×10^{-6}	7.78×10^{-5}
Bradykinesia [HP:0002067]	30/32 (94%)	4/10 (40%)	9.15×10^{-4}	0.013

(b) Fisher Exact Test performed to compare HPO annotation frequency with respect to Kufor-Rakeb syndrome (OMIM:606693) and Spastic paraplegia 78, autosomal recessive (OMIM:617225). Total of 29 tests were performed.

Genotype (A)	Genotype (B)	total tests performed	significant results
missense/missense OR missense/other	other/other	30	0

(c) Fisher Exact Test performed to compare HPO annotation frequency with respect to genotypes.

Figure S8: The cohort comprised 45 individuals (18 females, 27 males). A total of 71 HPO terms were used to annotate the cohort. Disease diagnoses: Kufor-Rakeb syndrome (OMIM:606693) (34 individuals), Spastic paraplegia 78, autosomal recessive (OMIM:617225) (11 individuals). As expected, the frequency of Parkinsonian manifestations is higher in Kufor-Rakeb syndrome, a rare autosomal recessive form of juvenile-onset atypical Parkinson disease. A total of 24 unique variant alleles were found in *ATP13A2* (transcript: NM_022089.4, protein id: NP_071372.1).

BRD4

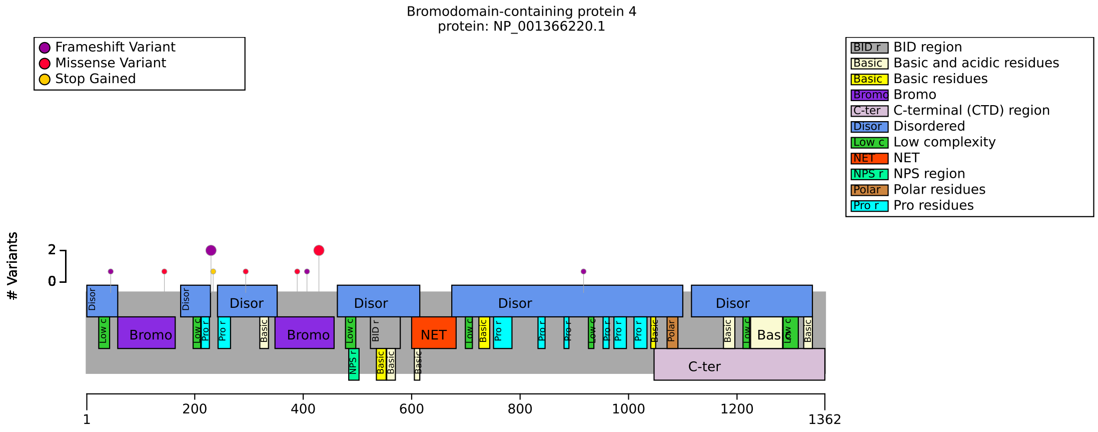

(a) Distribution of variants in *BRD4*

Genotype (A)	Genotype (B)	total tests performed	significant results
ablation	other	27	0
NIPBL	BRD4	41	0

(b) Fisher Exact Test performed to compare HPO annotation frequency with respect to genotypes.

Figure S9: The cohort comprised 18 individuals (8 females, 10 males). A total of 40 HPO terms were used to annotate the cohort. Disease diagnosis: Cornelia de Lange syndrome 6 (OMIM:620568). A total of 10 unique variant alleles were found in *BRD4* (transcript: `NM_001379291.1`, protein id: `NP_001366220.1`).

CHD8

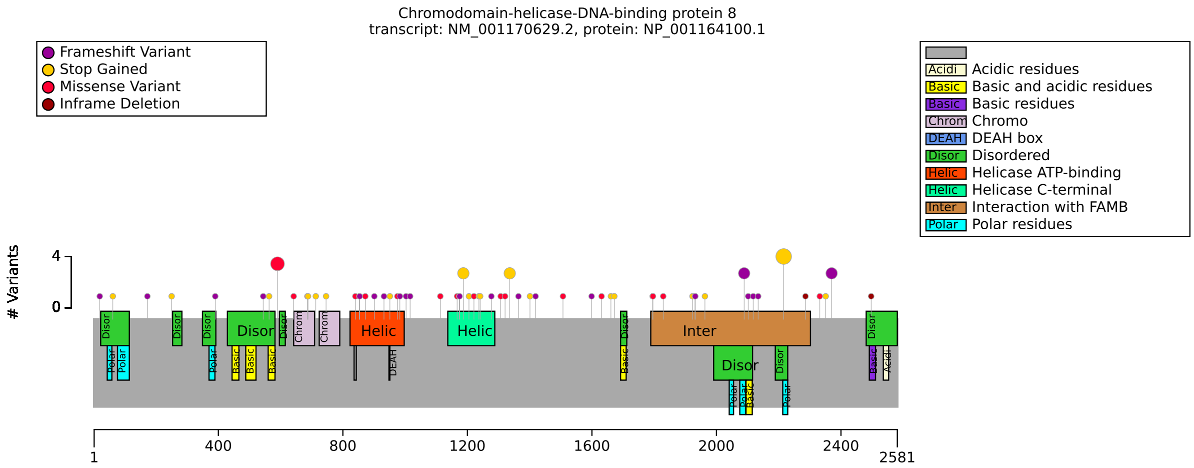

(**a**) Distribution of variants in *CHD8*

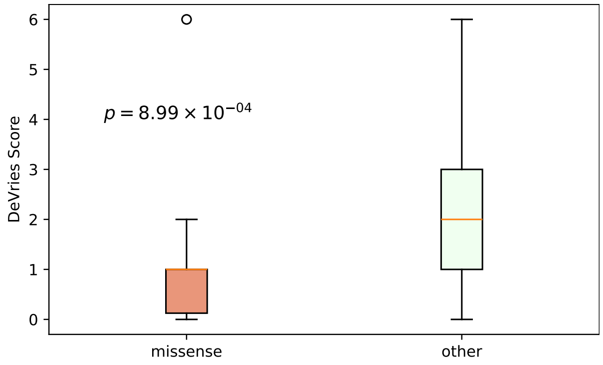

(**b**) De Vries Score to compare Missense and other variants.

Genotype (A)	Genotype (B)	total tests performed	significant results
Missense	Not Missense	36	0
FEMALE	MALE	36	0

(**c**) Fisher Exact Test performed to compare HPO annotation frequency with respect to genotypes.

Description	Variable	Genotype (A)	Genotype (B)	p-value	xrefs
De Vries score	De Vries score	Missense	Not Missense	8.99×10^{-4}	[5]
De Vries score	De Vries score	FEMALE	MALE	0.006	-

(d) De Vries Score to compare Missense and Other variants and for M/F sex differences.

Figure S10: The cohort comprised 79 individuals (26 females, 53 males). 1 of these individuals was reported to be deceased. A total of 81 HPO terms were used to annotate the cohort. Disease diagnosis: Intellectual developmental disorder with autism and macrocephaly (OMIM:615032). Dingemans et al. (2022) identified a correlation between the severity of the phenotypes (as measured by a phenotype severity score termed a DeVries test) and missense variants on the CHD8 gene, specifically that those with a missense variant were significantly less affected than other individuals [5]. A total of 70 unique variant alleles were found in *CHD8* (transcript: `NM_001170629.2`, protein id: `NP_001164100.1`).

CLDN16

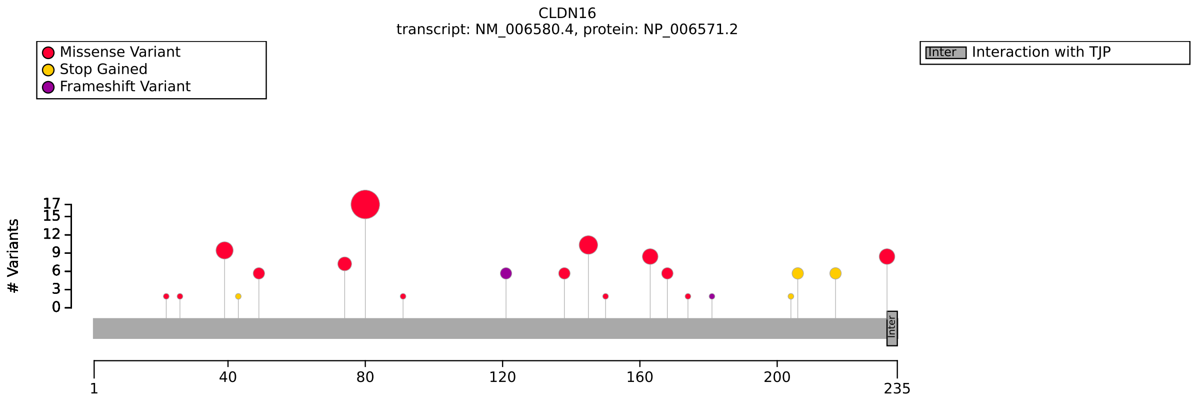

(a) Distribution of variants in *CLDN16*

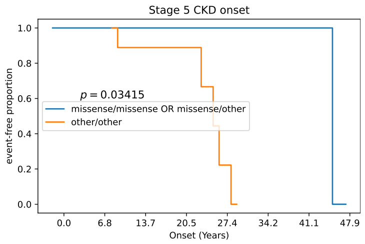

(b) Onset of stage 5 chronic kidney disease for *CLDN16* missense vs. other variants. This result is similar to that of Konrad et al. (2008) [6].

Genotype (A)	Genotype (B)	total tests performed	significant results
Leu81Phe/Leu81Phe	other/other OR Leu81Phe/other	26	0
missense/missense OR missense/other	other/other	23	0
FEMALE	MALE	27	0

(c) Fisher Exact Test performed to compare HPO annotation frequency with respect to genotypes.

Description	Variable	Genotype (A)	Genotype (B)	p-value	xrefs
Hypomagnesemia 3, renal (OMIM:248250) disease onset	Onset of OMIM:248250	missense/missense OR missense/other	other/other	0.333	[6]

(d) Onset of OMIM:248250 to compare missense/missense OR missense/other and other/other with respect to Onset of OMIM:248250.

Description	Variable	Genotype (A)	Genotype (B)	p-value	xrefs
Survival analysis: Stage 5 chronic kidney disease	Onset of HP:0003774	missense/missense OR missense/other	other/other	0.034	[6]

(e) Onset of Stage 5 chronic kidney disease to compare missense/missense OR missense/other and other/other with respect to Onset of HP:0003774.

Figure S11: The cohort comprised 51 individuals (21 females, 29 males, 1 with unknown sex). A total of 53 HPO terms were used to annotate the cohort. Disease diagnosis: Hypomagnesemia 3, renal (OMIM:248250). A total of 23 unique variant alleles were found in *CLDN16* (transcript: `NM_006580.4`, protein id: `NP_006571.2`).

CNTNAP2

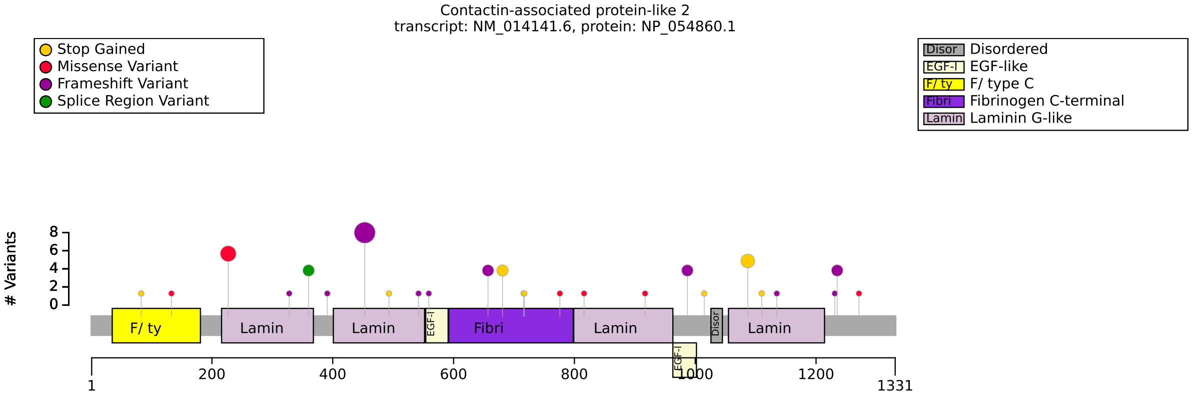

(a) Distribution of variants in *CNTNAP2*

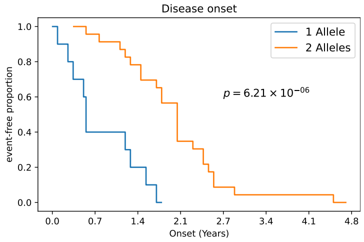

(b) Disease onset: Count of pathogenic *CNTNAP2* alleles

Genotype (A)	Genotype (B)	total tests performed	significant results
Ablation/Ablation	other/other OR Ablation/other	46	0
FEMALE	MALE	48	0
1 allele	2 alleles	43	0

(c) Fisher Exact Test performed to compare HPO annotation frequency with respect to genotypes.

Description	Variable	Genotype (A)	Genotype (B)	p-value	xrefs
Pitt-Hopkins like syndrome 1 (OMIM:610042) disease onset	Onset of OMIM:610042	1 allele	2 alleles	6.21×10^{-6}	-

(d) Onset of OMIM:610042 to compare 1 allele and 2 alleles with respect to Onset of OMIM:610042.

Figure S12: The cohort comprised 63 individuals (29 females, 32 males, 2 with unknown sex). 2 of these individuals were reported to be deceased. A total of 72 HPO terms were used to annotate the cohort. Disease diagnosis: Pitt-Hopkins like syndrome 1 (OMIM:610042). D'Onofrio et al. (2023) reported several GPCs, but the authors did not apply multiple-testing correction [7]. These authors did not perform survival analysis for age of onset of disease. A total of 30 unique variant alleles were found in *CNTNAP2* (transcript: `NM_014141.6`, protein id: `NP_054860.1`).

COQ4

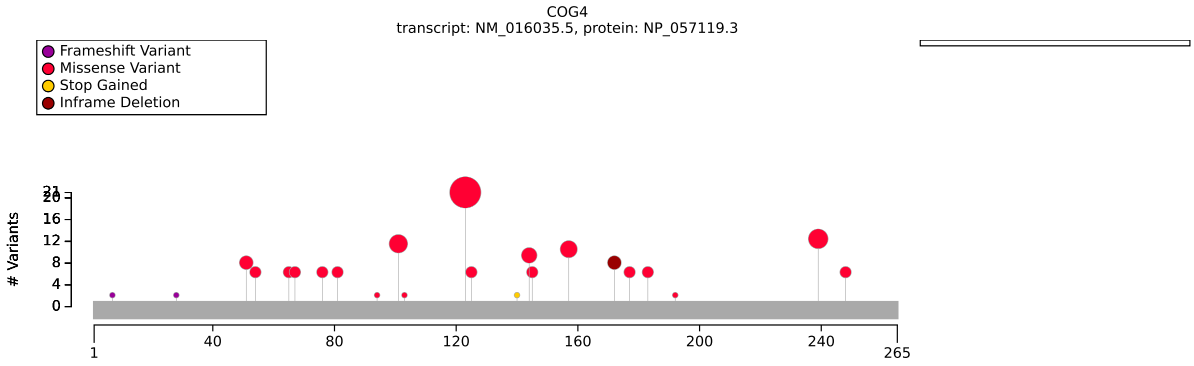

(a) Distribution of variants in *COQ4*

Genotype (A)	Genotype (B)	total tests performed	significant results
missense/other OR missense/missense	other/other	4	0
G124S/other OR G124S/G124S	other/other	13	0
G124S/other OR G124S/G124S	other/other	13	0
Exon 1-4/other OR Exon 1-4/Exon 1-4	other/other	13	0

(b) Fisher Exact Test performed to compare HPO annotation frequency with respect to genotypes.

Figure S13: The cohort comprised 51 individuals (30 females, 21 males). 19 of these individuals were reported to be deceased. A total of 91 HPO terms were used to annotate the cohort. A previous work found that pathogenic *COQ4* variants in exons 1-4 are associated with less life-threating presentations, late onset, responsiveness to CoQ10 therapy, and a relatively long lifespan, but correction for multiple testing was not performed [8]. Disease diagnoses: Coenzyme Q10 deficiency, primary, 7 (OMIM:616276) (35 individuals), Spastic ataxia 10, autosomal recessive (OMIM:620666) (16 individuals). A total of 32 unique variant alleles were found in *COQ4* (transcript: `NM_016035.5`, protein id: `NP_057119.3`).

COL3A1

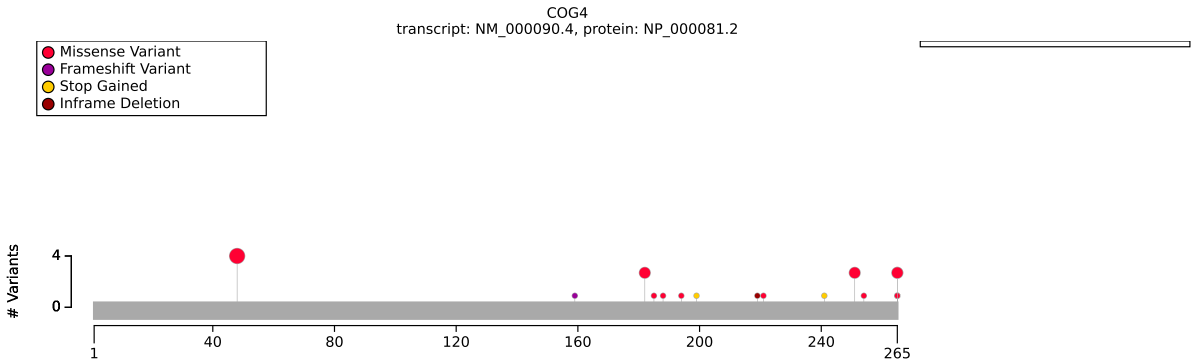

(a) Distribution of variants in *COL3A1*

Genotype (A)	Genotype (B)	total tests performed	significant results
Missense	Other	20	0
Missense	Other	20	0
triple helix region	Other	20	0
OMIM:130050	OMIM:618343	15	0
Gly missense	Other	20	0

(b) Fisher Exact Test performed to compare HPO annotation frequency with respect to genotypes.

Description	Variable	Genotype (A)	Genotype (B)	p-value	xrefs
Age of OMIM:130050 onset	Onset of OMIM:130050	missense	other	0.317	-

(c) Onset of OMIM:130050 to compare missense and other with respect to Onset of OMIM:130050.

Figure S14: The cohort comprised 41 individuals (24 females, 17 males). A total of 43 HPO terms were used to annotate the cohort. Disease diagnoses: Ehlers-Danlos syndrome, vascular type (OMIM:130050) (35 individuals), Polymicrogyria with or without vascular-type EDS (OMIM:618343) (6 individuals). No significant GPC identified. Frank et al (2015) found that glycine missense variants were associated with a higher degree of severity in a cohort of 215 individuals. Primary data was not made available. A total of 38 unique variant alleles were found in *COL3A1* (transcript: `NM_000090.4`, protein id: `NP_000081.2`).

CTCF

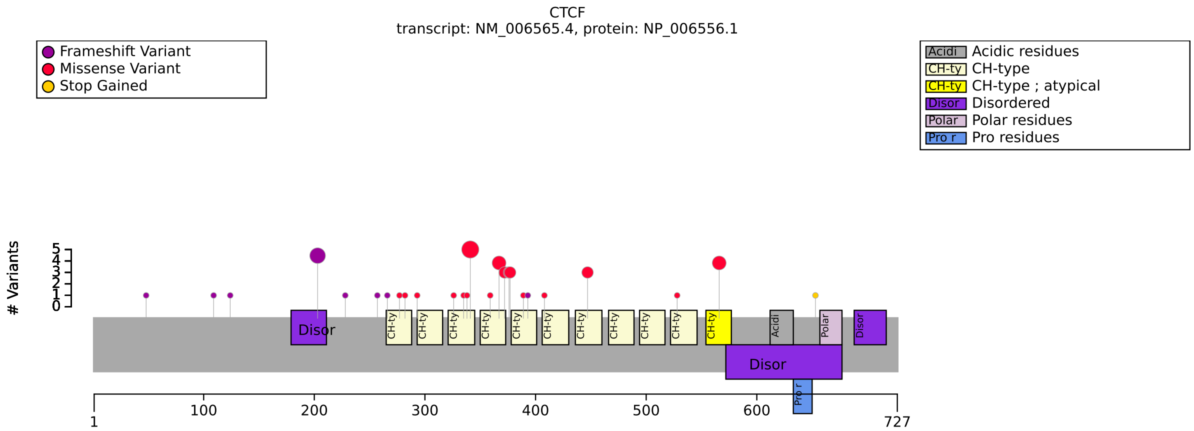

(a) Distribution of variants in *CTCF*

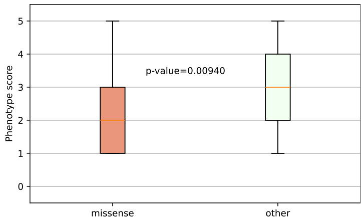

(b) DeVries score to compare *CTCF* missense vs. other variants.

Genotype (A)	Genotype (B)	total tests performed	significant results
missense	other	27	0

(c) Fisher Exact Test performed to compare HPO annotation frequency with respect to genotypes.

Description	Variable	Genotype (A)	Genotype (B)	p-value	xrefs
A phenotypic severity score for individuals with intellectual disability	De Vries score	missense	other	0.009	-

(d) De Vries Score to compare missense and other with respect to De Vries score.

Figure S15: The cohort comprised 46 individuals (18 females, 28 males). A total of 110 HPO terms were used to annotate the cohort. Disease diagnosis: Intellectual developmental disorder, autosomal dominant 21 (OMIM:615502). Previously, a group did not observe a correlation between the location of the variants and the overall severity of various phenotypes, including the level of intellectual function, presence of congenital anomalies, or poor growth [9], but analysis using a score such as the DeVries score was not attempted. A total of 32 unique variant alleles were found in *CTCF* (transcript: `NM_006565.4`, protein id: `NP_006556.1`).

CYP21A2

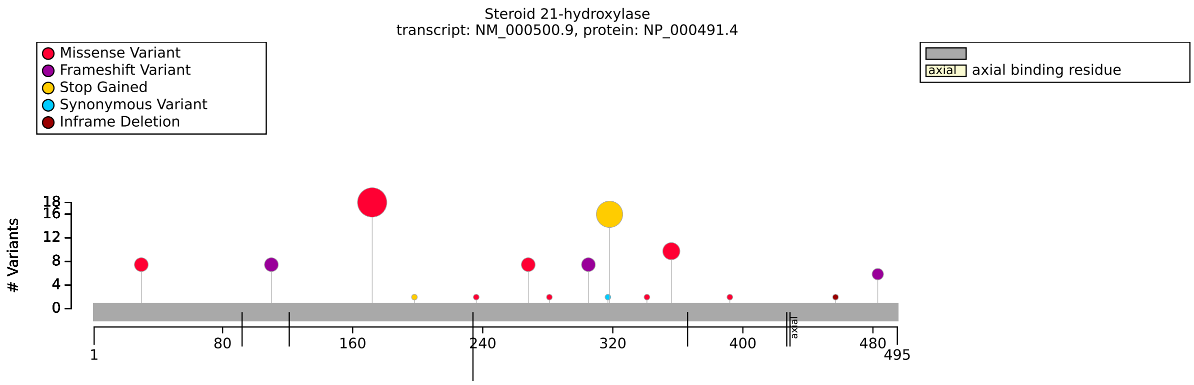

(a) Distribution of variants in *CYP21A2*

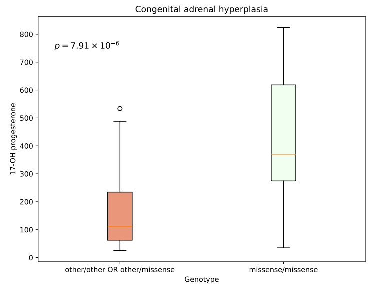

(b) t-test. 17-hydroxyprogesterone level. *CYP21A2* missense variants are compared with other variants.

Description	Variable	Genotype (A)	Genotype (B)	p-value	xrefs
17-Hydroxyprogesterone [Mass/volume] in Serum or Plasma [LOINC:1668-3]	LOINC:1668-3	other/other OR other/missense	missense/missense	7.91×10^{-6}	[10]

(c) t-test to compare other/other OR other/missense and missense/missense with respect to LOINC:1668-3.

Figure S16: The cohort comprised 69 individuals (34 females, 35 males). A total of 27 HPO terms were used to annotate the cohort. Disease diagnosis: Adrenal hyperplasia, congenital, due to 21-hydroxylase deficiency (OMIM:201910). It is assumed that the mildest mutation determines the phenotype in compound heterozygotes. Several missense variants display the highest residual activities. High levels of 17-hydroxyprogesterone may be observed with 21-hydroxylase deficiency [10]. A total of 22 unique variant alleles were found in *CYP21A2* (transcript: `NM_000500.9`, protein id: `NP_000491.4`).

EHMT1

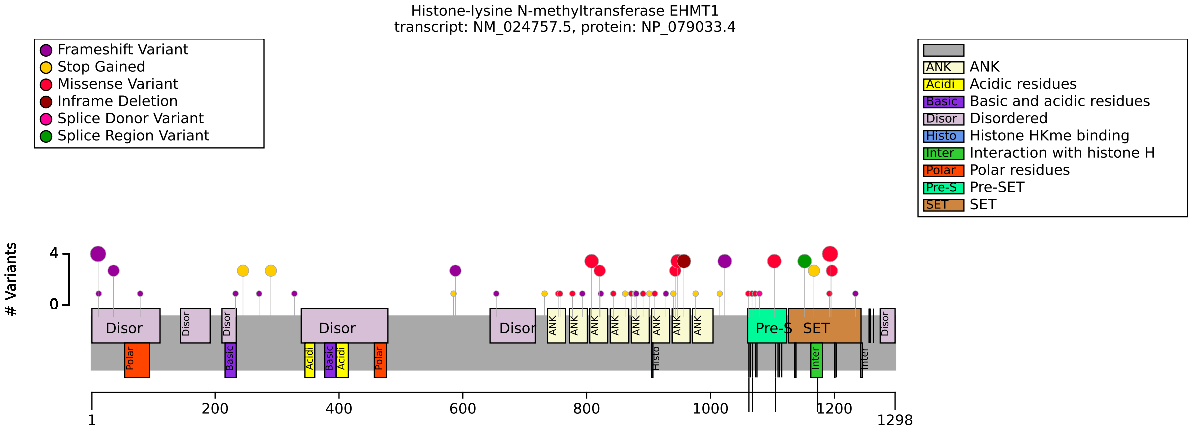

(a) Distribution of variants in *EHMT1*

HPO term	N Term Frameshift	other	p-value	adj. p-value
Attention deficit hyperactivity disorder [HP:0007018]	4/8 (50%)	3/96 (3%)	4.83×10^{-4}	0.029

(b) Fisher Exact Test performed to compare HPO annotation frequency with respect to N Term Frameshift and other. Total of 60 tests were performed.

Genotype (A)	Genotype (B)	total tests performed	significant results
Missense	Other	62	0
FEMALE	MALE	62	0
ANKR	other	62	0
SET	other	62	0

(c) Fisher Exact Test performed to compare HPO annotation frequency with respect to genotypes.

Figure S17: The cohort comprised 125 individuals (81 females, 44 males). A total of 60 HPO terms were used to annotate the cohort. Disease diagnosis: Kleefstra syndrome 1 (OMIM:610253). Rots et al. reported several correlations [11]. Multiple-testing correction was not performed. Frazier et al (2025) also reported significant correlations but the original data was not made available [12]. A total of 62 unique variant alleles were found in *EHMT1* (transcript: `NM_024757.5`, protein id: `NP_079033.4`).

EZH1

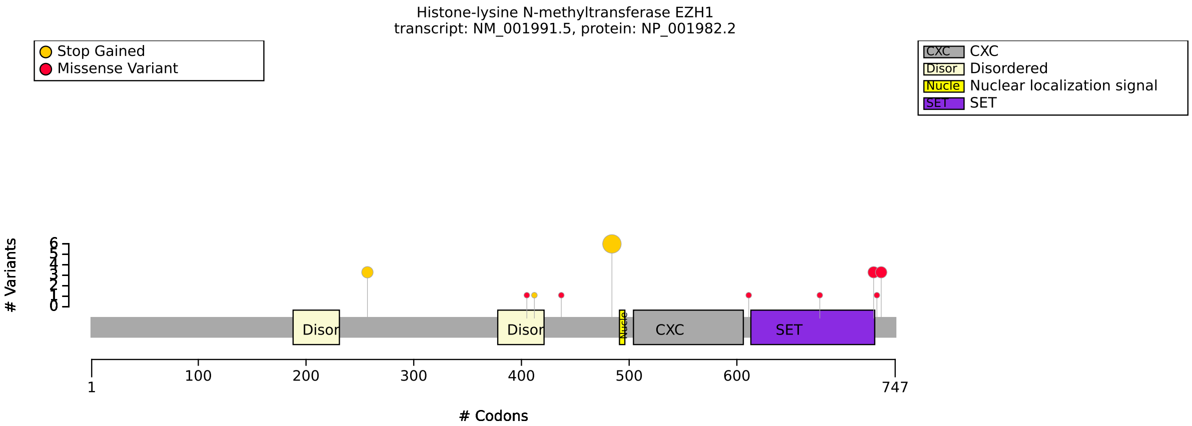

(a) Distribution of variants in *EZH1*

Genotype (A)	Genotype (B)	total tests performed	significant results
1 allele	2 alleles	33	0

(b) Fisher Exact Test performed to compare HPO annotation frequency with respect to monoallelic and biallelic pathogenic variants.

Figure S18: The cohort comprised 19 individuals (10 females, 8 males, 1 with unknown sex). A total of 106 HPO terms were used to annotate the cohort. Disease diagnosis: *EZH1*-related neurodevelopmental disorder (OMIM:601674). No statistically significant results identified. A total of 12 unique variant alleles were found in *EZH1* (transcript: `NM_001991.5`, protein id: `NP_001982.2`). In the original publication, it is stated that patients show neurodevelopmental delay with variable clinical presentations regardless of variant type and zygosity [13].

FBN1

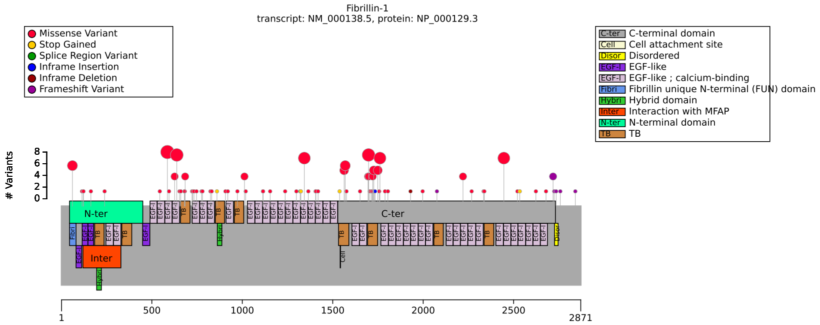

(a) Distribution of variants in *FBN1*

HPO term	missense	other	p-value	adj. p-value
Arachnodactyly [HP:0001166]	34/81 (42%)	18/22 (82%)	0.001	0.026
Thoracic aortic aneurysm [HP:0012727]	25/64 (39%)	12/14 (86%)	0.002	0.026
Hyperextensibility of the finger joints [HP:0001187]	0/78 (0%)	3/14 (21%)	0.003	0.026

(b) Fisher Exact Test: missense vs. other. Total of 27 tests were performed.

HPO term	TB domain	cbEGF	p-value	adj. p-value
Ectopia lentis [HP:0001083]	9/18 (50%)	48/59 (81%)	0.013	0.038
Mitral valve prolapse [HP:0001634]	1/28 (4%)	13/47 (28%)	0.012	0.038
Disproportionate tall stature [HP:0001519]	2/40 (5%)	12/43 (28%)	0.007	0.029
Tall stature [HP:0000098]	7/38 (18%)	21/40 (52%)	0.002	0.011
Severe short stature [HP:0003510]	15/36 (42%)	0/24 (0%)	1.36×10^{-4}	9.04×10^{-4}
Proportionate short stature [HP:0003508]	20/36 (56%)	0/24 (0%)	2.35×10^{-6}	2.35×10^{-5}
Short stature [HP:0004322]	23/39 (59%)	0/24 (0%)	4.39×10^{-7}	8.79×10^{-6}

(c) Fisher Exact Test: TB domain vs cbEGF. Total of 20 tests were performed.

HPO term	exon 37	other	p-value	adj. p-value
Arachnodactyly [HP:0001166]	0/8 (0%)	52/95 (55%)	0.003	0.019
Ectopia lentis [HP:0001083]	1/9 (11%)	78/100 (78%)	1.12×10^{-4}	0.001
Stiff skin [HP:0030053]	8/9 (89%)	0/50 (0%)	4.06×10^{-9}	8.52×10^{-8}

(d) Fisher Exact Test: exon 37 vs. other. Total of 21 tests were performed.

HPO term	fs last two	other	p-value	adj. p-value
Hyperextensibility of the finger joints [HP:0001187]	2/2 (100%)	1/90 (1%)	7.17×10^{-4}	0.012

(e) Fisher Exact Test: Frameshift in last two exons vs other. Total of 12 tests were performed.

Figure S19: The cohort comprised 144 individuals (57 females, 55 males, 32 with unknown sex). 4 of these individuals were reported to be deceased. A total of 109 HPO terms were used to annotate the cohort. Numerous articles on genotype-phenotype correlations have been published [14, 15, 16], but the available data is said to be be imprecise and incomplete [17] and many studies only share aggregate data. Disease diagnoses: Marfan syndrome (n=51), Ectopia lentis, familial (n=44), Geleophysic dysplasia 2 (n=19), Acromicric dysplasia (n=13), Marfan lipodystrophy syndrome (OMIM:616914) (n=9), Stiff skin syndrome (n=8). A total of 94 unique variant alleles were found in *FBN1* (transcript: `NM_000138.5`, protein id: `NP_000129.3`).

FBXL4

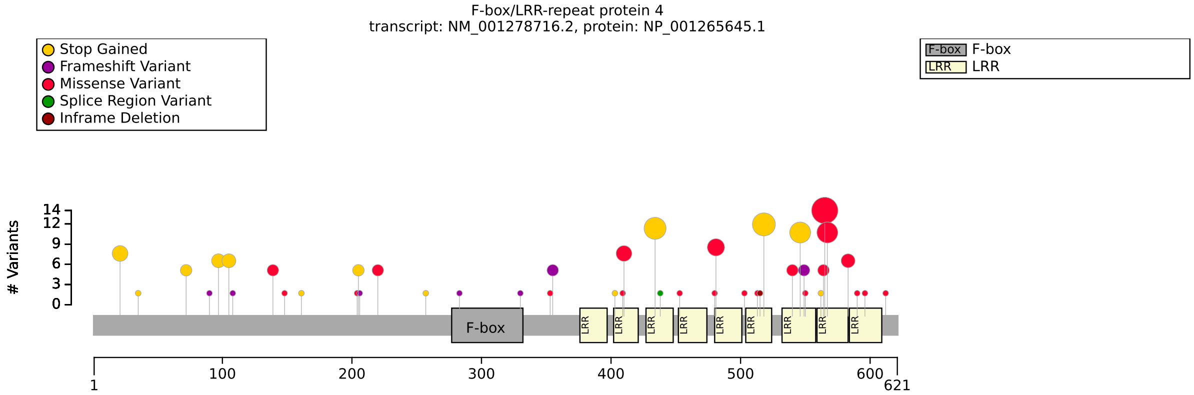

(a) Distribution of variants in *FBXL4*

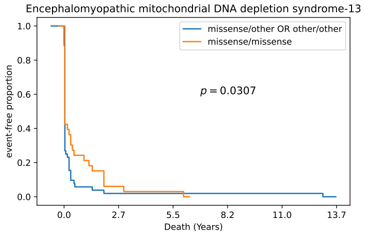

(b) Survival analysis.

HPO term	missense/other OR other/other	missense/missense	p-value	adj. p-value
Feeding difficulties [HP:0011968]	23/24 (96%)	13/27 (48%)	1.73×10^{-4}	0.010

(c) Fisher Exact Test performed to compare HPO annotation frequency with respect to missense/other OR other/other and missense/missense. Total of 55 tests were performed.

Genotype (A)	Genotype (B)	total tests performed	significant results
LRR domain/LRR domain OR LRR domain/other	other/other	55	0
N-Terminal (1-276)/N-Terminal (1-276) OR N-Terminal (1-276)/other	other/other	55	0
FEMALE	MALE	53	0

(d) Fisher Exact Test performed to compare HPO annotation frequency with respect to genotypes.

Description	Variable	Genotype (A)	Genotype (B)	p-value	xrefs
Age of OMIM:615471 onset	Onset of OMIM:615471	missense/other OR other/other	missense/missense	0.031	-
Age at postnatal death	Age of death	missense/other OR other/other	missense/missense	0.080	[18]

(e) Survival analysis for Age of death and onset, comparing compare missense/other OR other/other and missense/missense.

Figure S20: The cohort comprised 95 individuals (37 females, 58 males). 26 of these individuals were reported to be deceased. A total of 91 HPO terms were used to annotate the cohort. Disease diagnosis: Mitochondrial DNA depletion syndrome 13 (encephalomyopathic type) (OMIM:615471). It was reported that pathological variants in *FBXL4* indicated that genotypes with missense variants are frequently associated with longer survival [18]. Using the larger cohort analyzed here, mortality is not significantly associated with missense variants (p-value 0.08), but there was a significant association with age of onset (being later for missense variants) [18]. A total of 51 unique variant alleles were found in *FBXL4* (transcript: `NM_001278716.2`, protein id: `NP_001265645.1`).

FBXO11

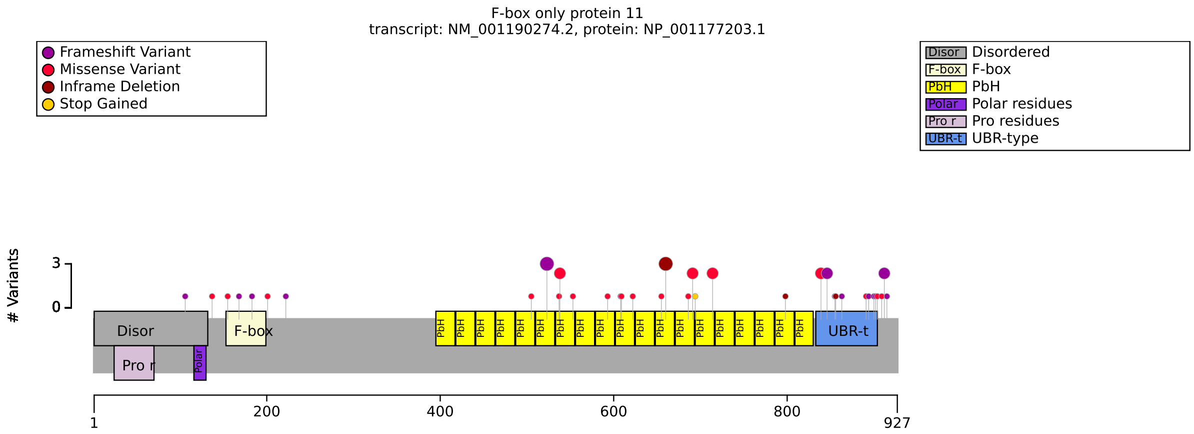

(**a**) Distribution of variants in *FBXO11*

Genotype (A)	Genotype (B)	total tests performed	significant results
Missense	Other	96	0
FEMALE	MALE	96	0

(**b**) Fisher Exact Test performed to compare HPO annotation frequency with respect to genotypes.

Figure S21: The cohort comprised 56 individuals (21 females, 35 males). A total of 128 HPO terms were used to annotate the cohort. Disease diagnosis: Intellectual developmental disorder with dysmorphic facies and behavioral abnormalities (OMIM:618089). No statistically significant results identified. A total of 43 unique variant alleles were found in *FBXO11* (transcript: `NM_001190274.2`, protein id: `NP_001177203.1`).

FGD1

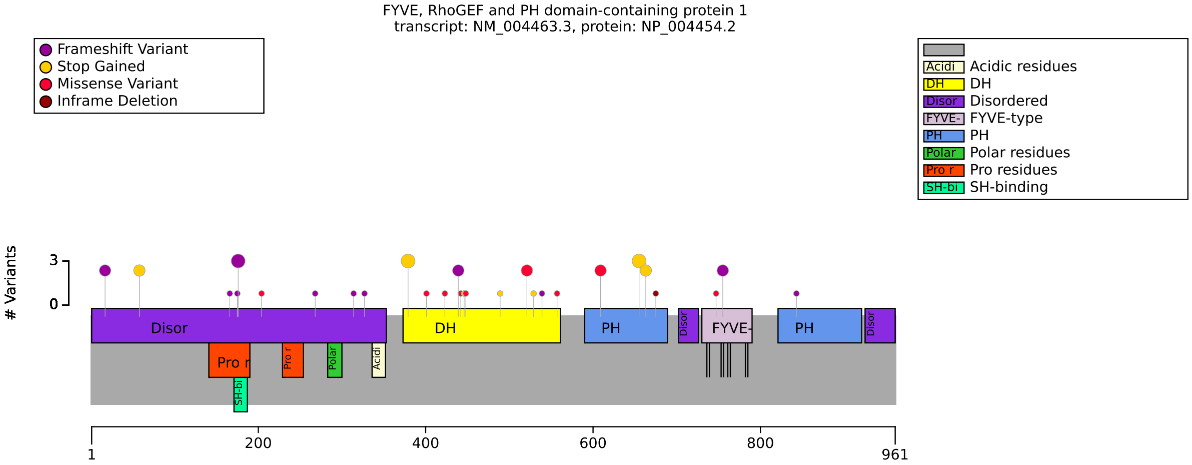

(a) Distribution of variants in *FGD1*

HPO term	Missense	Other	p-value	adj. p-value
Broad foot [HP:0001769]	1/7 (14%)	14/15 (93%)	6.22×10^{-4}	0.032

(b) Fisher Exact Test performed to compare HPO annotation frequency with respect to Missense and Other. Total of 52 tests were performed.

Genotype (A)	Genotype (B)	total tests performed	significant results
DH domain	Other	52	0

(c) Fisher Exact Test performed to compare HPO annotation frequency with respect to genotypes.

Figure S22: The cohort comprised 48 individuals (0 females, 48 males). A total of 73 HPO terms were used to annotate the cohort. Disease diagnosis: Aarskog-Scott syndrome (OMIM:305400). Li et al (2024) identified a number of correlations including a lower frequency of Deformity of foot (8/20) with missense than with ”drastic” variants (29/41); p=0.03. Multiple testing correction was not performed [19, 20]. A total of 35 unique variant alleles were found in *FGD1* (transcript: `NM_004463.3`, protein id: `NP_004454.2`).

FZD5

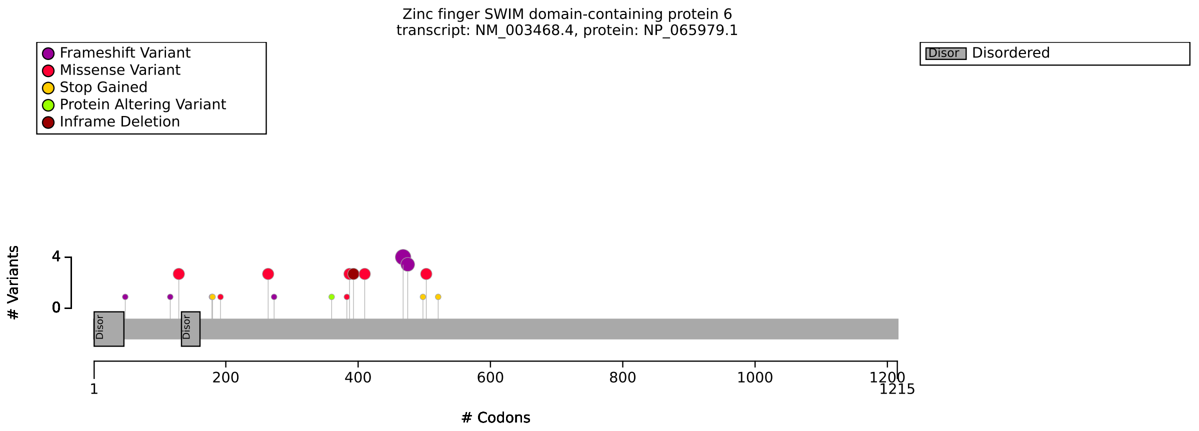

(a) Distribution of variants in *FZD5*

Genotype (A)	Genotype (B)	total tests performed	significant results
missense	other	18	0

(b) Fisher Exact Test performed to compare HPO annotation frequency with respect to genotypes.

Figure S23: The cohort comprised 29 individuals (11 females, 10 males, 8 with unknown sex). A total of 16 HPO terms were used to annotate the cohort. Disease diagnosis: Microphthalmia/coloboma 11 (OMIM:620731). No significant correlations identified. A total of 19 unique variant alleles were found in *FZD5* (transcript: NM_003468.4, protein id: NP_065979.1).

GLI3

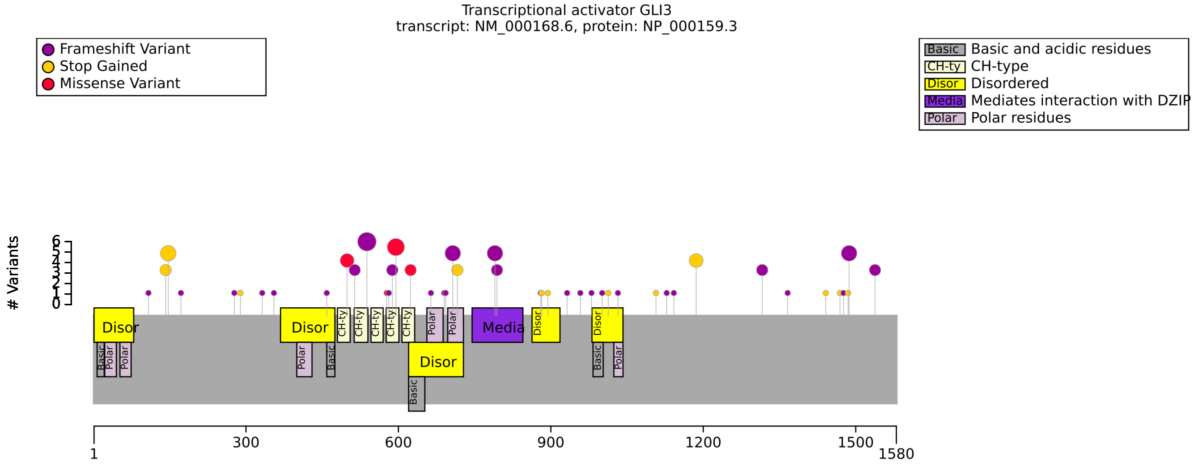

(a) Distribution of variants in *GLI3*

HPO term	Fs in mid region or splice	other	p-value	adj. p-value
Preaxial foot polydactyly [HP:0001841]	5/25 (20%)	32/57 (56%)	0.003	0.021
Y-shaped metacarpals [HP:0006042]	11/23 (48%)	4/56 (7%)	9.94×10^{-5}	0.001
Y-shaped metatarsals [HP:0010567]	11/19 (58%)	4/50 (8%)	3.25×10^{-5}	7.80×10^{-4}
Nail dysplasia [HP:0002164]	7/20 (35%)	2/54 (4%)	0.001	0.009
Anal atresia [HP:0002023]	7/25 (28%)	3/57 (5%)	0.007	0.036

(b) Fisher Exact Test performed to compare HPO annotation frequency with respect to Fs in mid region or splice and other. Total of 24 tests were performed.

HPO term	Truncating variants in Exon 15	other	p-value	adj. p-value
Macrocephaly [HP:0000256]	13/16 (81%)	15/42 (36%)	0.003	0.017
Preaxial foot polydactyly [HP:0001841]	7/28 (25%)	30/54 (56%)	0.010	0.050
Syndactyly [HP:0001159]	5/17 (29%)	33/38 (87%)	4.82×10^{-5}	0.001
Postaxial foot polydactyly [HP:0001830]	9/20 (45%)	2/35 (6%)	8.91×10^{-4}	0.011
Anal atresia [HP:0002023]	8/28 (29%)	2/54 (4%)	0.002	0.017

(c) Fisher Exact Test performed to compare HPO annotation frequency with respect to Truncating variants in Exon 15 and other. Total of 24 tests were performed.

HPO term	Variants in C-terminal third	other	p-value	adj. p-value
Macrocephaly [HP:0000256]	13/15 (87%)	15/43 (35%)	7.31×10^{-4}	0.004
Postaxial hand polydactyly [HP:0001162]	15/16 (94%)	21/49 (43%)	3.40×10^{-4}	0.003
Syndactyly [HP:0001159]	5/16 (31%)	33/39 (85%)	2.25×10^{-4}	0.003
Postaxial foot polydactyly [HP:0001830]	9/16 (56%)	2/39 (5%)	7.35×10^{-5}	0.002

(d) Fisher Exact Test performed to compare HPO annotation frequency with respect to Variants in C-terminal third and other. Total of 24 tests were performed.

Figure S24: The cohort comprised 82 individuals (3 females, 7 males, 72 with unknown sex). A total of 34 HPO terms were used to annotate the cohort. Disease diagnoses: Greig cephalopolysyndactyly syndrome (OMIM:175700) (51 individuals), Pallister-Hall syndrome (OMIM:146510) (21 individuals), Polydactyly, postaxial, types A1 and B (OMIM:174200) (10 individuals). Genotype-Phenotype-Correlations in *GLI3* have been extensively investigated, with findings being similar but not identical to those reported here [21, 22, 23, 24, 25, 26, 27]. A total of 49 unique variant alleles were found in *GLI3* (transcript: `NM_000168.6`, protein id: `NP_000159.3`).

HMGCS2

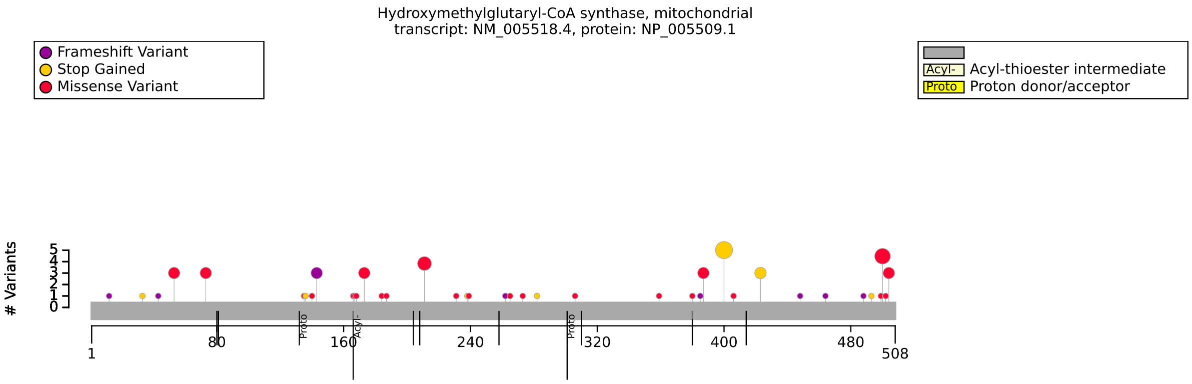

(a) Distribution of variants in *HMGCS2*

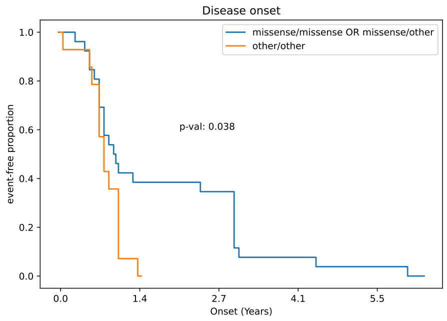

(b) Onset of *HMGCS2* for missense/missense and missense/other vs. other/other variants.

Genotype (A)	Genotype (B)	total tests performed	significant results
missense/missense OR missense/other	other/other	19	0
missense/missense OR missense/other	other/other	19	0
FEMALE	MALE	17	0

(c) Fisher Exact Test performed to compare HPO annotation frequency with respect to genotypes.

Description	Variable	Genotype (A)	Genotype (B)	p-value	xrefs
HMG-CoA synthase-2 deficiency (OMIM:605911) disease onset	Onset of OMIM:605911	missense/missense OR missense/other	other/other	0.038	-

(d) Onset of OMIM:605911 to compare missense/missense OR missense/other and other/other with respect to Onset of OMIM:605911.

Figure S25: The cohort comprised 40 individuals (14 females, 13 males, 13 with unknown sex). 1 of these individuals were reported to be deceased. A total of 44 HPO terms were used to annotate the cohort. Disease diagnosis: HMG-CoA synthase-2 deficiency (OMIM:605911). No previous publications with results on genotype-phenotype correlations in *HMGCS2* were identified. A total of 46 unique variant alleles were found in *HMGCS2* (transcript: `NM_005518.4`, protein id: `NP_005509.1`).

IKZF1

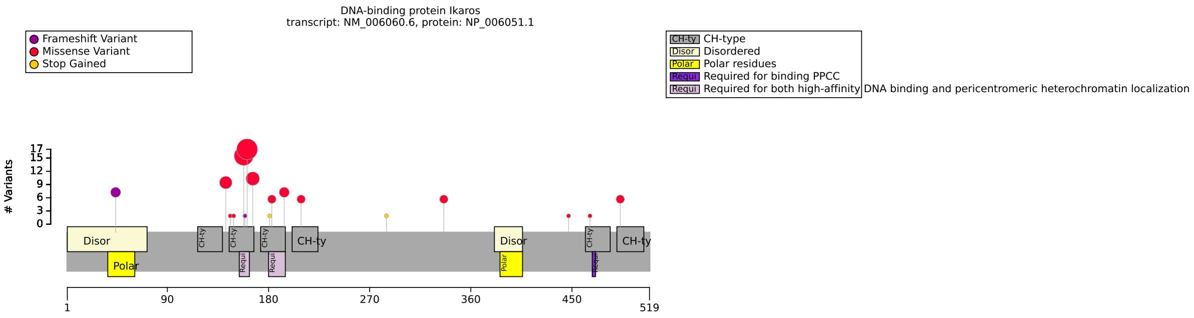

(**a**) Distribution of variants in *IKZF1*

HPO term	p.Asn159Ser	Other variant	p-value	adj. p-value
Recurrent pneumonia [HP:0006532]	7/9 (78%)	7/39 (18%)	0.001	0.035
T-cell acute lymphoblastic leukemias [HP:0006727]	3/13 (23%)	0/69 (0%)	0.003	0.047

(**b**) Fisher Exact Test performed to compare HPO annotation frequency with respect to p.Asn159Ser and Other variant. Total of 29 tests were performed.

Genotype (A)	Genotype (B)	total tests performed	significant results
Missense	oher	34	0

(**c**) Fisher Exact Test performed to compare HPO annotation frequency with respect to genotypes.

Figure S26: The cohort comprised 82 individuals (34 females, 37 males, 11 with unknown sex). 5 of these individuals were reported to be deceased. A total of 66 HPO terms were used to annotate the cohort. Disease diagnosis: Immunodeficiency, common variable, 13 (OMIM:616873). The variant p.Asn159Ser was functionally characterized to be dominant-negative and result in a combined-immunodeficiency phenotype that was distinct from other *IKZF1* variants [28]. A total of 26 unique variant alleles were found in *IKZF1* (transcript: `NM_006060.6`, protein id: `NP_006051.1`).

ITPR1

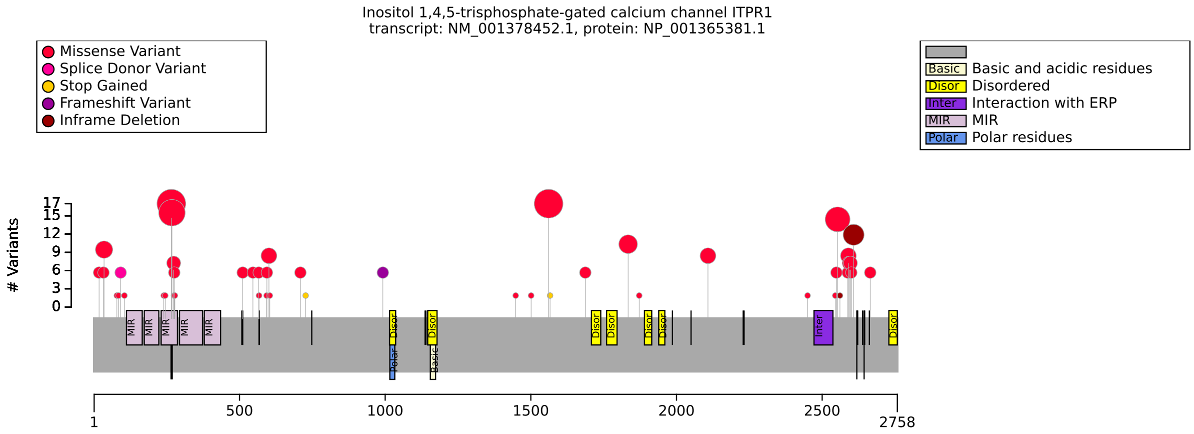

(a) Distribution of variants in *ITPR1*

HPO term	IP3 binding	other	p-value	adj. p-value
Delayed speech and language development [HP:0000750]	13/13 (100%)	37/63 (59%)	0.003	0.019
Neurodevelopmental delay [HP:0012758]	29/29 (100%)	70/90 (78%)	0.003	0.019
Aniridia [HP:0000526]	0/14 (0%)	20/54 (37%)	0.007	0.024
Delayed gross motor development [HP:0002194]	18/18 (100%)	57/83 (69%)	0.005	0.024
Motor delay [HP:0001270]	24/24 (100%)	63/89 (71%)	0.002	0.019
Nystagmus [HP:0000639]	25/26 (96%)	53/78 (68%)	0.003	0.019
Delayed ability to sit [HP:0025336]	11/11 (100%)	42/70 (60%)	0.013	0.043
Delayed ability to walk [HP:0031936]	13/13 (100%)	41/68 (60%)	0.004	0.019

(b) Fisher Exact Test performed to compare HPO annotation frequency with respect to IP3 binding and other. Total of 26 tests were performed.

HPO term	SV Deletion	other	p-value	adj. p-value
Delayed speech and language development [HP:0000750]	0/19 (0%)	50/57 (88%)	1.72×10^{-12}	7.57×10^{-12}
Neurodevelopmental delay [HP:0012758]	0/19 (0%)	99/100 (99%)	4.07×10^{-21}	8.96×10^{-20}
Delayed gross motor development [HP:0002194]	0/19 (0%)	75/82 (91%)	4.04×10^{-15}	2.96×10^{-14}
Motor delay [HP:0001270]	0/19 (0%)	87/94 (93%)	3.90×10^{-16}	4.29×10^{-15}
Global developmental delay [HP:0001263]	0/19 (0%)	48/57 (84%)	1.81×10^{-11}	5.68×10^{-11}
Nystagmus [HP:0000639]	17/17 (100%)	61/87 (70%)	0.006	0.016
Delayed ability to sit [HP:0025336]	0/19 (0%)	53/62 (85%)	4.56×10^{-12}	1.67×10^{-11}
Delayed ability to walk [HP:0031936]	0/19 (0%)	54/62 (87%)	1.47×10^{-12}	7.57×10^{-12}

(c) Fisher Exact Test performed to compare HPO annotation frequency with respect to SV Deletion and other. Total of 22 tests were performed.

HPO term	GS Hotspot	other	p-value	adj. p-value
Aniridia [HP:0000526]	14/20 (70%)	6/48 (12%)	6.13×10^{-6}	1.59×10^{-4}
Delayed gross motor development [HP:0002194]	18/18 (100%)	57/83 (69%)	0.005	0.032
Motor delay [HP:0001270]	19/19 (100%)	68/94 (72%)	0.006	0.032
Delayed ability to sit [HP:0025336]	13/13 (100%)	40/68 (59%)	0.003	0.030
Delayed ability to walk [HP:0031936]	14/14 (100%)	40/67 (60%)	0.003	0.030

(d) Fisher Exact Test performed to compare HPO annotation frequency with respect to GS Hotspot and other. Total of 25 tests were performed.

Figure S27: The cohort comprised 170 individuals (65 females, 41 males, 64 with unknown sex). 1 of these individuals was reported to be deceased. A Fisher exact test for male/female differences revealed no significant associations. A total of 196 HPO terms were used to annotate the cohort. Disease diagnoses: Spinocerebellar ataxia 29, congenital nonprogressive (OMIM:117360) (104 individuals), Gillespie syndrome (OMIM:206700) (39 individuals), Spinocerebellar ataxia 15 (OMIM:606658) (27 individuals). A total of 62 unique variant alleles were found in *ITPR1* (transcript: `NM_001378452.1`, protein id: `NP_001365381.1`).

Kabuki

HPO term	OMIM:147920	OMIM:300867	p-value	adj. p-value
Feeding difficulties [HP:0011968]	8/25 (32%)	55/63 (87%)	7.41×10^{-7}	2.52×10^{-5}
Motor delay [HP:0001270]	4/10 (40%)	58/61 (95%)	1.04×10^{-4}	0.002

(a) Fisher Exact Test performed to compare HPO annotation frequency with respect to OMIM:147920 and OMIM:300867. Total of 34 tests were performed.

Figure S28: The cohort comprised 146 individuals (81 females, 65 males). 5 of these individuals were reported to be deceased. A total of 200 HPO terms were used to annotate the cohort. Disease diagnoses: Kabuki syndrome 2 (OMIM:300867) (81 individuals), Kabuki Syndrome 1 (OMIM:147920) (65 individuals). Previous studies have found that the phenotype of KS2 is different from that of KS1, for example, patients with KS1 type have a higher risk of typical facial features, short stature, and frequent infections compared to those with KS2 [29, 30, 31]. A total of 105 unique variant alleles were found in *n/a* (transcript: `n/a`, protein id: `n/a`).

KCNH5

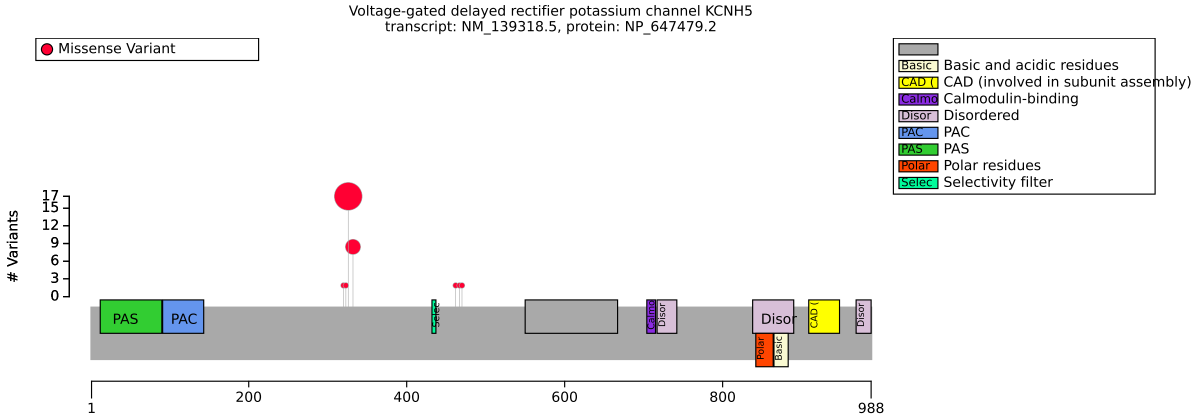

(**a**) Distribution of variants in *KCNH5*

HPO term	Arg327His	Arg333His	p-value	adj. p-value
Epileptic encephalopathy [HP:0200134]	15/15 (100%)	0/3 (0%)	0.001	0.032

(**b**) Fisher Exact Test performed to compare HPO annotation frequency with respect to Arg327His and Arg333His. Total of 26 tests were performed.

Genotype (A)	Genotype (B)	total tests performed	significant results
FEMALE	MALE	32	0

(**c**) Fisher Exact Test performed to compare HPO annotation frequency with respect to genotypes.

Figure S29: The cohort comprised 27 individuals (14 females, 13 males). A total of 28 HPO terms were used to annotate the cohort. Disease diagnosis: Developmental and epileptic encephalopathy 112 (OMIM:620537). Two recurrent variants have been reported, p.Arg327His and p.Arg333His, both of which are located in or near the functionally critical voltage-sensing or pore domains. Happ et al (2023) state that in their cohort, Individuals with the recurrent p.Arg333His variant had a self-limited drug-responsive focal or generalized epilepsy and normal intellect, whereas the recurrent p.Arg327His variant was associated with infantile-onset DEE [32]. A total of 8 unique variant alleles were found in *KCNH5* (transcript: `NM_139318.5`, protein id: `NP_647479.2`).

KDM6A

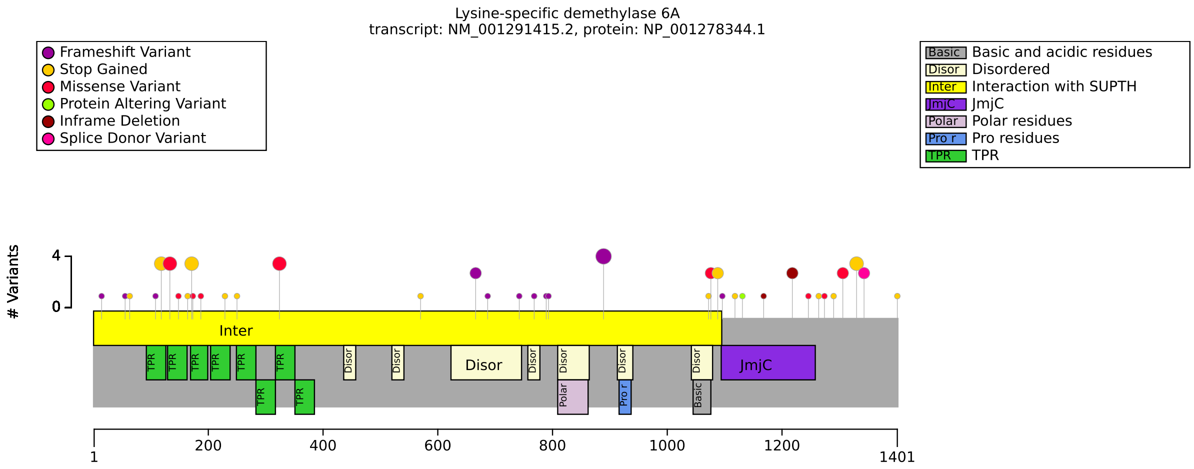

(a) Distribution of variants in *KDM6A*

HPO term	FEMALE	MALE	p-value	adj. p-value
Intellectual disability, severe [HP:0010864]	7/25 (28%)	14/18 (78%)	0.002	0.008

(b) Fisher Exact Test performed to compare HPO annotation frequency with respect to FEMALE and MALE. Total of 4 tests were performed. A similar sex difference was reported in ref. [33].

HPO term	p.Asn891ValfsTer27	Other	p-value	adj. p-value
Pulmonic stenosis [HP:0001642]	2/2 (100%)	0/59 (0%)	5.46×10^{-4}	0.016

(c) Fisher Exact Test performed to compare HPO annotation frequency with respect to p.Asn891ValfsTer27 and Other. Total of 29 tests were performed.

Genotype (A)	Genotype (B)	total tests performed	significant results
missense	other	47	0
SV	other	47	0

(d) Fisher Exact Test performed to compare HPO annotation frequency with respect to genotypes.

Figure S30: The cohort comprised 81 individuals (46 females, 35 males). A total of 87 HPO terms were used to annotate the cohort. Disease diagnosis: Kabuki syndrome 2 (OMIM:300867). . A total of 53 unique variant alleles were found in *KDM6A* (transcript: NM_001291415.2, protein id: NP_001278344.1).

KDM6B

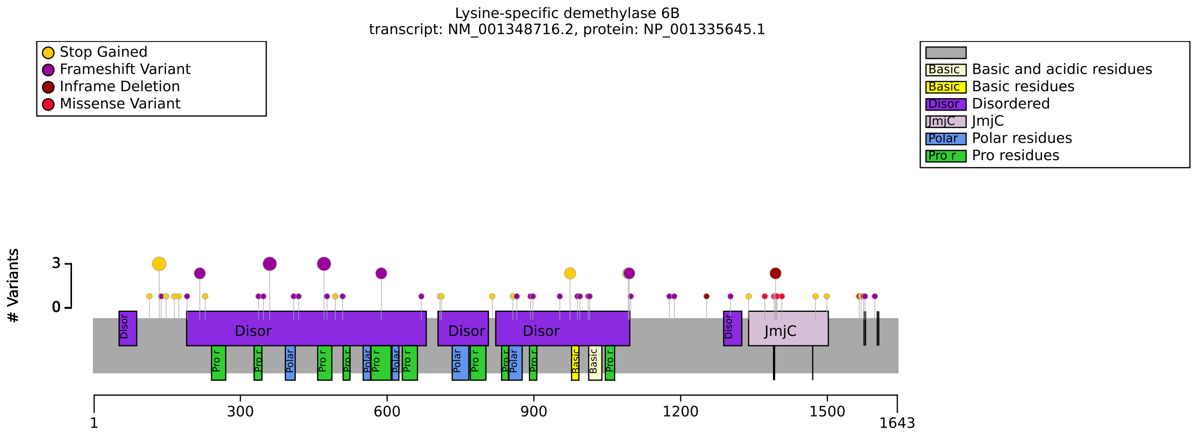

(a) Distribution of variants in *KDM6B*

Genotype (A)	Genotype (B)	total tests performed	significant results
JmjC domain	Other	54	0
N term	C term	54	0

(b) Fisher Exact Test performed to compare HPO annotation frequency with respect to genotypes.

Figure S31: The cohort comprised 73 individuals (19 females, 54 males). A total of 251 HPO terms were used to annotate the cohort. Disease diagnosis: Neurodevelopmental disorder with coarse facies and mild distal skeletal abnormalities (OMIM:618505). Stolerman et al (2016) identified no significant genotype-phenotype correlations [34] A total of 61 unique variant alleles were found in *KDM6B* (transcript: `NM_001348716.2`, protein id: `NP_001335645.1`).

KMT2D

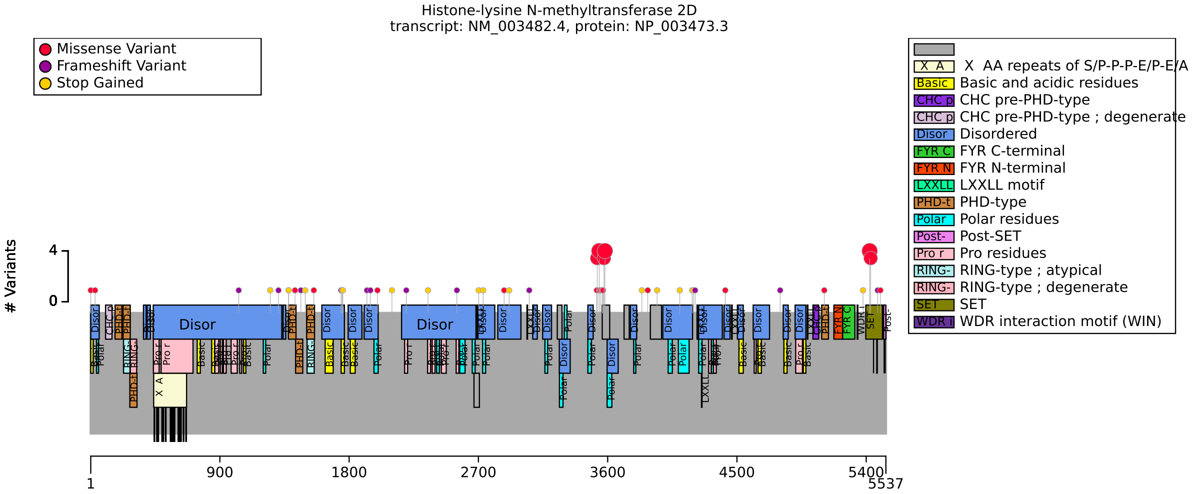

(a) Distribution of variants in *KMT2D*

Genotype (A)	Genotype (B)	total tests performed	significant results
FEMALE	MALE	42	0
missense	other	42	0
n_term	other	41	0
p.Glu5425Lys	other	36	0
p.Leu3542Pro	Other variant	8	0

(b) Fisher Exact Test performed to compare HPO annotation frequency with respect to genotypes.

Figure S32: The cohort comprised 65 individuals (35 females, 30 males). 5 of these individuals were reported to be deceased. A total of 151 HPO terms were used to annotate the cohort. Disease diagnosis: Kabuki Syndrome 1 (OMIM:147920). A total of 52 unique variant alleles were found in *KMT2D* (transcript: `NM_003482.4`, protein id: `NP_003473.3`).

LDS 1 and 2

Genotype (A)	Genotype (B)	total tests performed	significant results
OMIM:609192	OMIM:610168	36	0

(a) Fisher Exact Test performed to compare HPO annotation frequency with respect to genotypes.

Figure S33: The cohort comprised 94 individuals (30 females, 46 males, 18 with unknown sex). 6 of these individuals were reported to be deceased. A total of 110 HPO terms were used to annotate the cohort. Disease diagnoses: Loeys-Dietz syndrome 2 (OMIM:610168) (53 individuals), Loeys-Dietz syndrome 1 (OMIM:609192) (23 individuals), Multiple self-healing squamous epithelioma, susceptibility to (OMIM:132800) (18 individuals). A total of 61 unique variant alleles were found.

LDS 1 and 3

HPO term	OMIM:609192	OMIM:613795	p-value	adj. p-value
Scoliosis [HP:0002650]	18/21 (86%)	20/43 (47%)	0.003	0.027
Hypertelorism [HP:0000316]	15/19 (79%)	13/35 (37%)	0.004	0.027
Aortic aneurysm [HP:0004942]	11/11 (100%)	26/48 (54%)	0.004	0.027
Osteoarthritis [HP:0002758]	0/11 (0%)	26/38 (68%)	4.64×10^{-5}	0.001

(a) Fisher Exact Test performed to compare HPO annotation frequency with respect to OMIM:609192 and OMIM:613795. Total of 24 tests were performed.

Figure S34: The cohort comprised 90 individuals (29 females, 43 males, 18 with unknown sex). 2 of these individuals were reported to be deceased. A total of 89 HPO terms were used to annotate the cohort. Disease diagnoses: Loeys-Dietz syndrome 3 (OMIM:613795) (49 individuals), Loeys-Dietz syndrome 1 (OMIM:609192) (23 individuals), Multiple self-healing squamous epithelioma, susceptibility to (OMIM:132800) (18 individuals). A total of 37 unique variant alleles were found.

LDS 3 and 6

HPO term	OMIM:613795	OMIM:619656	p-value	adj. p-value
Thoracic aortic aneurysm [HP:0012727]	0/22 (0%)	10/16 (62%)	1.69×10^{-5}	4.23×10^{-4}

(a) Fisher Exact Test performed to compare HPO annotation frequency with respect to OMIM:613795 and OMIM:619656. Total of 25 tests were performed.

Figure S35: The cohort comprised 72 individuals (35 females, 35 males, 2 with unknown sex). A total of 98 HPO terms were used to annotate the cohort. Disease diagnoses: Loeys-Dietz syndrome 3 (OMIM:613795) (49 individuals), Loeys-Dietz syndrome 6 (OMIM:619656) (18 individuals), Congenital heart defects, multiple types, 8, with or without heterotaxy (OMIM:619657) (5 individuals). A total of 25 unique variant alleles were found.

LMNA (Part 1 of 2)

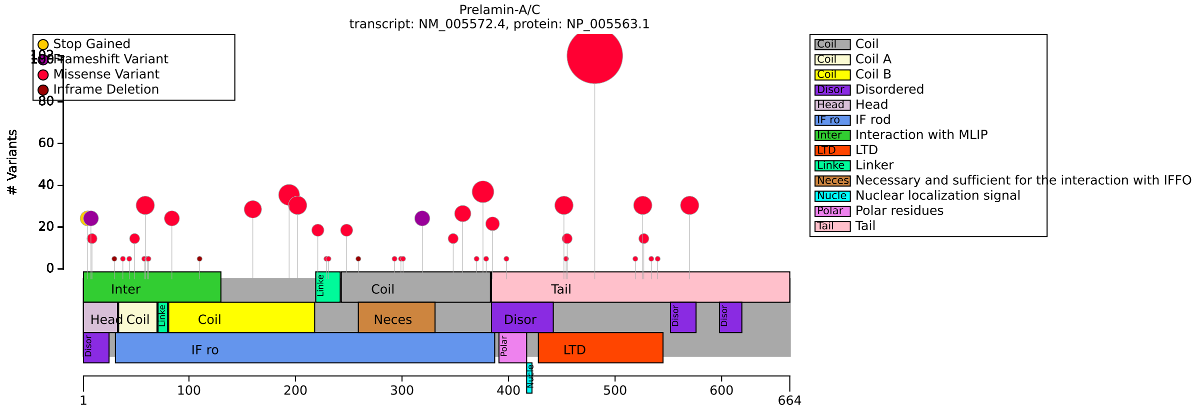

(a) Distribution of variants in *LMNA*

HPO term	missense	other	p-value	adj. p-value
Loss of truncal subcutaneous adipose tissue [HP:0009002]	104/104 (100%)	4/11 (36%)	7.53×10^{-9}	2.86×10^{-7}
Elevated hemoglobin A1c [HP:0040217]	73/101 (72%)	6/24 (25%)	3.11×10^{-5}	5.90×10^{-4}

(b) Fisher Exact Test performed to compare HPO annotation frequency with respect to missense and other. Total of 38 tests were performed.

HPO term	Upstream of NLS	other	p-value	adj. p-value
Lipodystrophy [HP:0009125]	10/93 (11%)	132/158 (84%)	1.73×10^{-31}	6.58×10^{-30}
Pancreatitis [HP:0001733]	4/7 (57%)	14/110 (13%)	0.011	0.033
Dilated cardiomyopathy [HP:0001644]	35/70 (50%)	5/102 (5%)	4.20×10^{-12}	7.98×10^{-11}
Second degree atrioventricular block [HP:0011706]	6/68 (9%)	1/115 (1%)	0.011	0.033
Atrioventricular block [HP:0001678]	17/36 (47%)	8/116 (7%)	2.11×10^{-7}	2.67×10^{-6}
Achilles tendon contracture [HP:0001771]	21/85 (25%)	19/30 (63%)	2.66×10^{-4}	0.001

(c) Fisher Exact Test: HPO annotation frequency and NLS Upstream vs other variants. 38 tests were performed in total.

Figure S36: See following page for caption

LMNA (Part 2 of 2)

HPO term	Upstream of NLS	other	p-value	adj. p-value
Foot joint contracture [HP:0008366]	21/81 (26%)	19/27 (70%)	6.20×10^{-5}	5.89×10^{-4}
Lower-limb joint contracture [HP:0005750]	22/82 (27%)	19/27 (70%)	8.20×10^{-5}	6.23×10^{-4}
Limb joint contracture [HP:0003121]	23/83 (28%)	19/27 (70%)	1.71×10^{-4}	8.14×10^{-4}
Elbow contracture [HP:0034391]	21/83 (25%)	18/26 (69%)	1.04×10^{-4}	6.59×10^{-4}
Upper-limb joint contracture [HP:0100360]	21/81 (26%)	18/26 (69%)	1.23×10^{-4}	6.66×10^{-4}
Hip contracture [HP:0003273]	4/83 (5%)	9/26 (35%)	2.68×10^{-4}	0.001
First degree atrioventricular block [HP:0011705]	7/63 (11%)	2/115 (2%)	0.010	0.033

(a) Fisher Exact Test performed to compare HPO annotation frequency with respect to NLS Upstream and other. Total of 38 tests were performed.

HPO term	Gly608=	Other	p-value	adj. p-value
Lipodystrophy [HP:0009125]	15/15 (100%)	127/236 (54%)	1.87×10^{-4}	7.47×10^{-4}
Elevated hemoglobin A1c [HP:0040217]	0/15 (0%)	79/110 (72%)	5.65×10^{-8}	6.77×10^{-7}
Muscle weakness [HP:0001324]	0/15 (0%)	63/124 (51%)	6.07×10^{-5}	3.64×10^{-4}
Proximal muscle weakness [HP:0003701]	0/15 (0%)	53/114 (46%)	3.63×10^{-4}	0.001
Distal muscle weakness [HP:0002460]	0/15 (0%)	36/97 (37%)	0.002	0.004
Proximal muscle weakness in upper limbs [HP:0008997]	0/15 (0%)	35/102 (34%)	0.005	0.007
Upper limb muscle weakness [HP:0003484]	0/15 (0%)	35/96 (36%)	0.003	0.004
Limb muscle weakness [HP:0003690]	0/15 (0%)	38/99 (38%)	0.002	0.004

(b) Fisher Exact Test performed to compare HPO annotation frequency with respect to A and B. Total of 12 tests were performed.

Description	Variable	Genotype (A)	Genotype (B)	p-value	xrefs
Phenotype score (see notebook)	HPO group count	NLS Upstream	other	1.83×10^{-16}	[35]

(c) HPO Group Count to compare NLS Upstream and other with respect to HPO group count.

Figure S37: The cohort comprised 266 individuals (150 females, 99 males, 17 with unknown sex). 11 of these individuals were reported to be deceased. A total of 152 HPO terms were used to annotate the cohort. Disease diagnoses: Lipodystrophy, familial partial, type 2 (OMIM:151660) (127 individuals), Cardiomyopathy, dilated, 1A (OMIM:115200) (68 individuals), Emery-Dreifuss muscular dystrophy 2, autosomal dominant (OMIM:181350) (41 individuals), Hutchinson-Gilford progeria (OMIM:176670) (15 individuals), *LMNA*-related congenital muscular dystrophy (OMIM:613205) (15 individuals). Numerous *LMNA* genotype-phenotype correlations have been described. The literature is summarized in [36]. Cardiac involvement in multisystem laminopathies prevails with mutations upstream of the nuclear localisation signal [35]. A total of 55 unique variant alleles were found in *LMNA* (transcript: `NM_005572.4`, protein id: `NP_005563.1`).

LZTR1

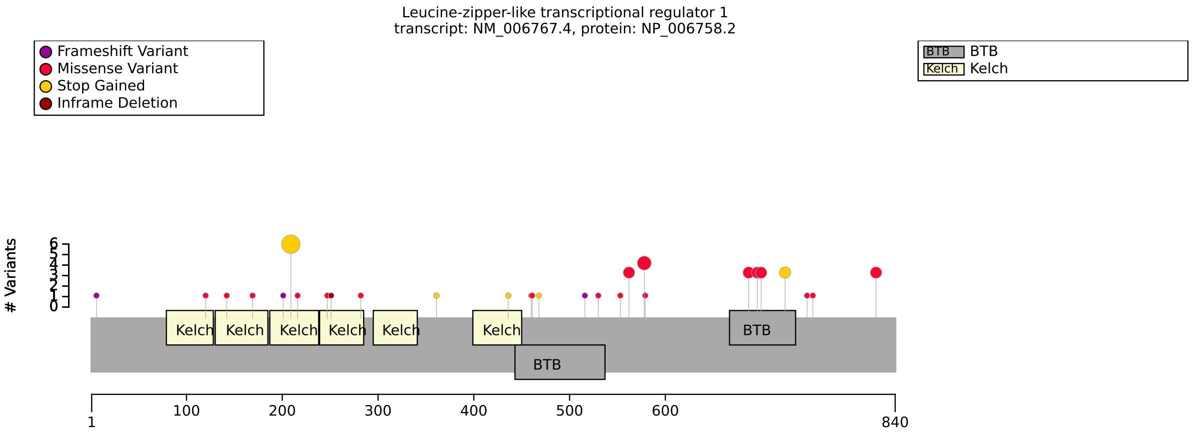

(a) Distribution of variants in *LZTR1*

Genotype (A)	Genotype (B)	total tests performed	significant results
N Term/N Term OR N Term/Other	Other/Other	49	0
Missense/Missense OR Missense/Other	Other/Other	49	0
FEMALE	MALE	54	0

(b) Fisher Exact Test performed to compare HPO annotation frequency with respect to genotypes.

Figure S38: The cohort comprised 38 individuals (18 females, 20 males). 3 of these individuals were reported to be deceased. A total of 96 HPO terms were used to annotate the cohort. Disease diagnosis: Noonan syndrome 2 (OMIM:605275). No significant association identified. A total of 38 unique variant alleles were found in *LZTR1* (transcript: `NM_006767.4`, protein id: `NP_006758.2`).

MAPK8IP3

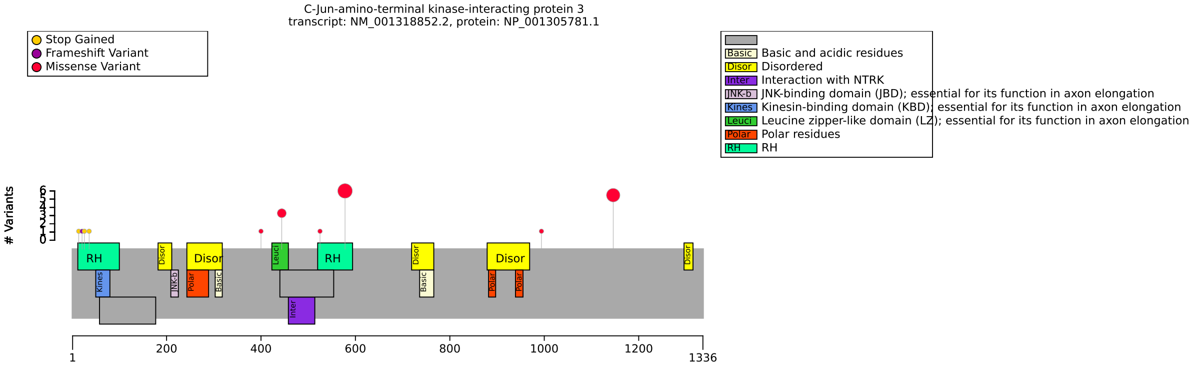

(a) Distribution of variants in *MAPK8IP3*

Genotype (A)	Genotype (B)	total tests performed	significant results
N term	other	68	0
p.Arg579Cys	Other variant	68	0
RH2	Other region	68	0
FEMALE	MALE	68	0

(b) Fisher Exact Test performed to compare HPO annotation frequency with respect to genotypes.

Figure S39: The cohort comprised 20 individuals (9 females, 11 males). A total of 93 HPO terms were used to annotate the cohort. Disease diagnosis: Neurodevelopmental disorder with or without variable brain abnormalities (OMIM:618443). No significant association identified. A total of 10 unique variant alleles were found in *MAPK8IP3* (transcript: `NM_001318852.2`, protein id: `NP_001305781.1`).

MPV17

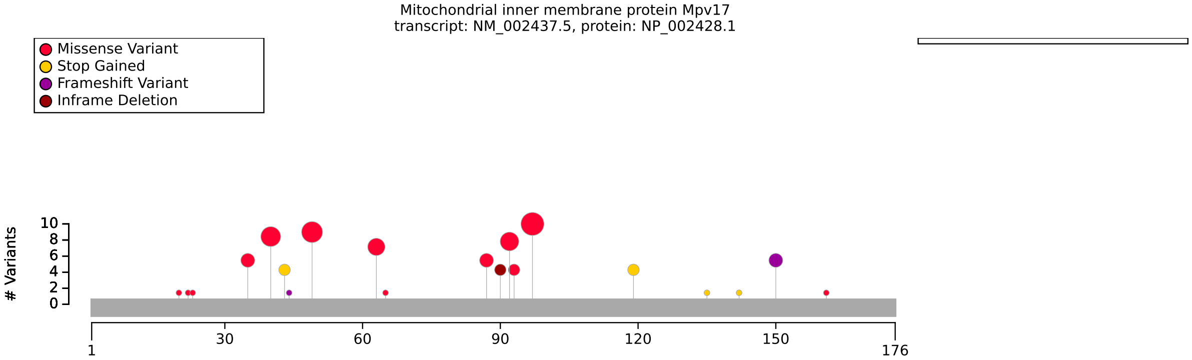

(a) Distribution of variants in *MPV17*

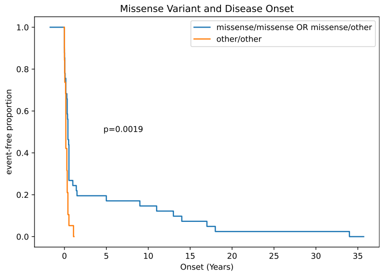

(b) *MPV17* disease onset

HPO term	Pro98Leu/Pro98Leu	other/other OR Pro98Leu/other	p-value	adj. p-value
Peripheral axonal neuropathy [HP:0003477]	3/3 (100%)	1/22 (5%)	0.002	0.037

(c) Fisher Exact Test performed to compare HPO annotation frequency with respect to Pro98Leu/Pro98Leu and other/other OR Pro98Leu/other. Total of 21 tests were performed.

Genotype (A)	Genotype (B)	total tests performed	significant results
missense/missense OR missense/other	other/other	32	0
Arg50Gln/Arg50Gln OR Arg50Gln/other	other/other	29	0
FEMALE	MALE	32	0

(d) Fisher Exact Test performed to compare HPO annotation frequency with respect to genotypes.

Description	Variable	Genotype (A)	Genotype (B)	p-value	xrefs
MTDPS6 (OMIM:256810) age at death	Age of death	Pro98Leu/Pro98Leu	other/other OR Pro98Leu/other	0.010	[37, 38]

(e) Age of death to compare Pro98Leu/Pro98Leu and other/other OR Pro98Leu/other with respect to Age of death.

Description	Variable	Genotype (A)	Genotype (B)	p-value	xrefs
MTDPS6 (OMIM:256810) disease onset	Onset of OMIM:256810	missense/missense OR missense/other	other/other	0.002	-

(f) Onset of OMIM:256810 to compare missense/missense OR missense/other and other/other with respect to Onset of OMIM:256810.

Figure S40: The cohort comprised 60 individuals (30 females, 30 males). 39 of these individuals were reported to be deceased. A total of 156 HPO terms were used to annotate the cohort. Disease diagnosis: Mitochondrial DNA depletion syndrome 6 (hepatocerebral type) (MTDPS6; OMIM:256810). No clear genotype-phenotype correlation exists. However, a trend for longer survival can be observed in individuals with biallelic pathogenic missense variants compared to individuals with biallelic null [37, 38]. A total of 31 unique variant alleles were found in *MPV17* (transcript: `NM_002437.5`, protein id: `NP_002428.1`).

NBAS

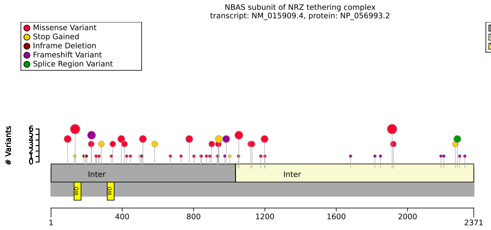

(a) Distribution of variants in *NBAS*

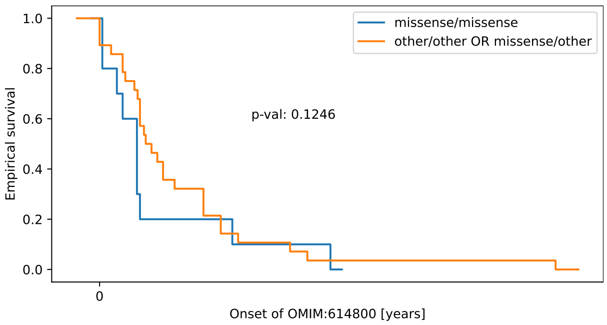

(b) *NBAS* missense variants vs. age at death.

HPO term	missense/missense	other/other OR missense/other	p-value	adj. p-value
Decreased circulating IgG concentration [HP:0004315]	1/12 (8%)	15/23 (65%)	0.002	0.035

(c) Fisher Exact Test performed to compare HPO annotation frequency with respect to missense/missense and other/other OR missense/other. Total of 22 tests were performed.

Description	Variable	Genotype (A)	Genotype (B)	p-value	xrefs
Age of death	Age of death	missense/missense	other/other OR missense/other	0.125	-

(d) Age of death to compare missense/missense and other/other OR missense/other with respect to Age of death.

Figure S41: The cohort comprised 67 individuals (19 females, 21 males, 27 with unknown sex). 9 of these individuals were reported to be deceased. A total of 134 HPO terms were used to annotate the cohort. Disease diagnosis: Short stature, optic nerve atrophy, and Pelger-Huet anomaly (OMIM:614800). It was reported that missense or in-frame deletions in the C-terminal region of *NBAS* are associated with SOPH syndrome (614800), missense or in-frame deletions in the Sec30 domain of *NBAS* are associated with infantile liver failure syndrome type 2 (616483), while missense or in-frame deletions in the beta-propeller domain of *NBAS* are associated with a combined phenotype of multisystem involvement with acute liver failure [39]. In our dataset, we found an association with missense or in-frame deletions in the Sec30 with decreased circulating IgG. A similar finding was obtain for missense variants in general. Due to the small size of our cohort, this finding should be regarded as preliminary. A total of 74 unique variant alleles were found in *NBAS* (transcript: `NM_015909.4`, protein id: `NP_056993.2`).

NF1

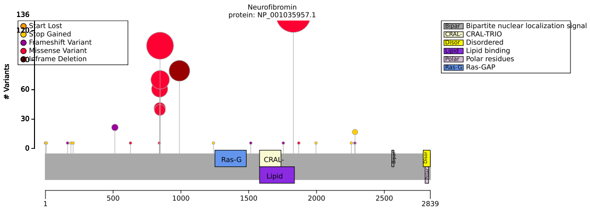

(a) Distribution of variants in *NF1*

HPO term	Missense	other	p-value	adj. p-value
Freckling [HP:0001480]	181/278 (65%)	96/117 (82%)	7.20×10^{-4}	0.009
Glioma [HP:0009733]	35/282 (12%)	11/27 (41%)	5.31×10^{-4}	0.009
Lipoma [HP:0012032]	4/277 (1%)	5/51 (10%)	0.006	0.030
Spinal neurofibroma [HP:0009735]	23/228 (10%)	0/67 (0%)	0.003	0.021
Neurofibroma by anatomical location [HP:0034761]	23/206 (11%)	0/67 (0%)	0.002	0.015

(b) Fisher Exact Test performed to compare HPO annotation frequency with respect to Missense and other. Total of 26 tests were performed.

HPO term	p.Arg1830	other	p-value	adj. p-value
Freckling [HP:0001480]	76/133 (57%)	201/262 (77%)	1.11×10^{-4}	2.99×10^{-4}
Lisch nodules [HP:0009737]	11/91 (12%)	72/193 (37%)	6.58×10^{-6}	1.97×10^{-5}
Neurofibroma [HP:0001067]	5/133 (4%)	153/275 (56%)	2.48×10^{-28}	6.71×10^{-27}
Axillary freckling [HP:0000997]	20/79 (25%)	105/175 (60%)	3.66×10^{-7}	1.41×10^{-6}
Inguinal freckling [HP:0030052]	8/79 (10%)	84/170 (49%)	4.86×10^{-10}	3.28×10^{-9}
Plexiform neurofibroma [HP:0009732]	0/135 (0%)	68/255 (27%)	1.45×10^{-14}	1.96×10^{-13}
Scoliosis [HP:0002650]	8/125 (6%)	48/240 (20%)	4.02×10^{-4}	9.87×10^{-4}
Optic nerve glioma [HP:0009734]	0/135 (0%)	39/217 (18%)	1.92×10^{-9}	1.04×10^{-8}
Glioma [HP:0009733]	2/136 (1%)	44/173 (25%)	2.36×10^{-10}	2.13×10^{-9}
Spinal neurofibroma [HP:0009735]	0/128 (0%)	23/167 (14%)	1.90×10^{-6}	6.42×10^{-6}
Neurofibroma by anatomical location [HP:0034761]	0/128 (0%)	23/145 (16%)	2.05×10^{-7}	9.23×10^{-7}
Pulmonic stenosis [HP:0001642]	13/105 (12%)	5/162 (3%)	0.005	0.011

(c) Fisher Exact Test performed to compare HPO annotation frequency with respect to p.Arg1830 and other. Total of 27 tests were performed.

HPO term	Met992del	other	p-value	adj. p-value
Lisch nodules [HP:0009737]	3/36 (8%)	80/248 (32%)	0.003	0.008
Neurofibroma [HP:0001067]	0/44 (0%)	158/364 (43%)	1.68×10^{-10}	2.68×10^{-9}
Axillary freckling [HP:0000997]	0/14 (0%)	125/240 (52%)	8.57×10^{-5}	6.86×10^{-4}
Inguinal freckling [HP:0030052]	0/14 (0%)	92/235 (39%)	0.003	0.008
Plexiform neurofibroma [HP:0009732]	0/44 (0%)	68/346 (20%)	2.00×10^{-4}	0.001
Lipoma [HP:0012032]	5/44 (11%)	4/284 (1%)	0.003	0.008

(d) Fisher Exact Test performed to compare HPO annotation frequency with respect to Met992del and other. Total of 16 tests were performed.

NF1 (part 2)

HPO term	Leu847Pro	other	p-value	adj. p-value
Freckling [HP:0001480]	54/64 (84%)	223/331 (67%)	0.007	0.023
Lisch nodules [HP:0009737]	22/42 (52%)	61/242 (25%)	7.48×10^{-4}	0.004
Neurofibroma [HP:0001067]	47/67 (70%)	111/341 (33%)	1.58×10^{-8}	2.14×10^{-7}
Axillary freckling [HP:0000997]	50/61 (82%)	75/193 (39%)	3.55×10^{-9}	9.58×10^{-8}
Inguinal freckling [HP:0030052]	34/60 (57%)	58/189 (31%)	3.92×10^{-4}	0.003
Plexiform neurofibroma [HP:0009732]	24/65 (37%)	44/325 (14%)	4.45×10^{-5}	4.00×10^{-4}
Optic nerve glioma [HP:0009734]	15/65 (23%)	24/287 (8%)	0.002	0.008
Glioma [HP:0009733]	18/67 (27%)	28/242 (12%)	0.003	0.013
Neurofibroma by anatomical location [HP:0034761]	6/26 (23%)	17/247 (7%)	0.014	0.041

(a) Fisher Exact Test performed to compare HPO annotation frequency with respect to Leu847Pro and other. Total of 27 tests were performed.

HPO term	SV	other	p-value	adj. p-value
Freckling [HP:0001480]	53/60 (88%)	224/335 (67%)	6.36×10^{-4}	0.005
Neurofibroma [HP:0001067]	41/60 (68%)	117/348 (34%)	6.03×10^{-7}	1.39×10^{-5}
Plexiform neurofibroma [HP:0009732]	16/50 (32%)	52/340 (15%)	0.008	0.037
Scoliosis [HP:0002650]	18/57 (32%)	38/308 (12%)	9.71×10^{-4}	0.006
Glioma [HP:0009733]	8/11 (73%)	38/298 (13%)	1.60×10^{-5}	1.84×10^{-4}

(b) Fisher Exact Test performed to compare HPO annotation frequency with respect to SV and other. Total of 23 tests were performed.

Figure S43: The cohort comprised 419 individuals (192 females, 169 males, 58 with unknown sex). A total of 36 HPO terms were used to annotate the cohort. Disease diagnosis: Neurofibromatosis, type 1 (OMIM:162200). A substantial body of literature exists about genotype-phenotype correlations. The correlations described in this notebook have been previously reported [40, 41, 42]. A total of 36 unique variant alleles were found in *NF1* (transcript: `NM_001042492.3`, protein id: `NP_001035957.1`).

NIPBL

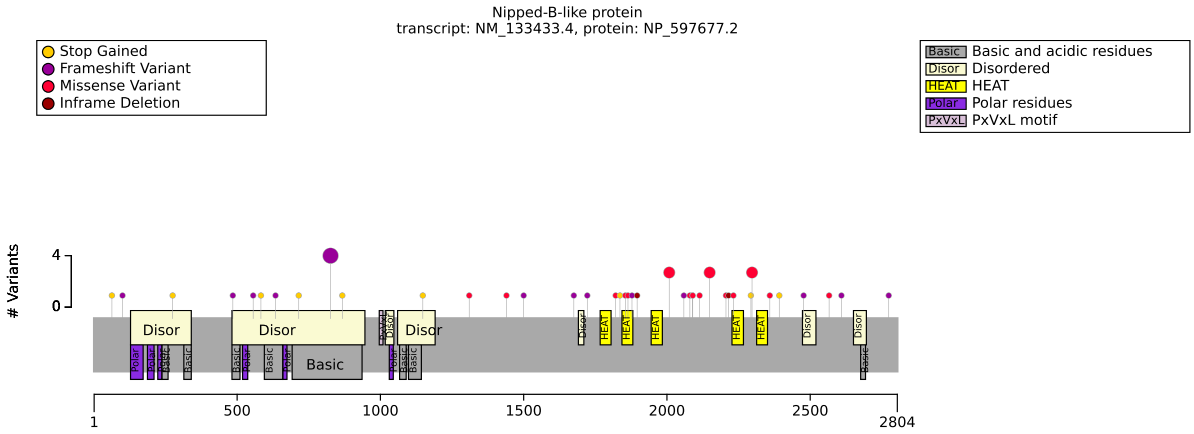

(a) Distribution of variants in *NIPBL*

Genotype (A)	Genotype (B)	total tests performed	significant results
missense	other	86	0
disordered	other	86	0
FEMALE	MALE	81	0

(b) Fisher Exact Test performed to compare HPO annotation frequency with respect to genotypes.

Figure S44: The cohort comprised 60 individuals (21 females, 31 males, 8 with unknown sex). A total of 90 HPO terms were used to annotate the cohort. Disease diagnosis: Cornelia de Lange syndrome 1 (OMIM:122470). No statistically significant genotype-phenotype correlation identified. A total of 50 unique variant alleles were found in *NIPBL* (transcript: `NM_133433.4`, protein id: `NP_597677.2`).

NKX6-2

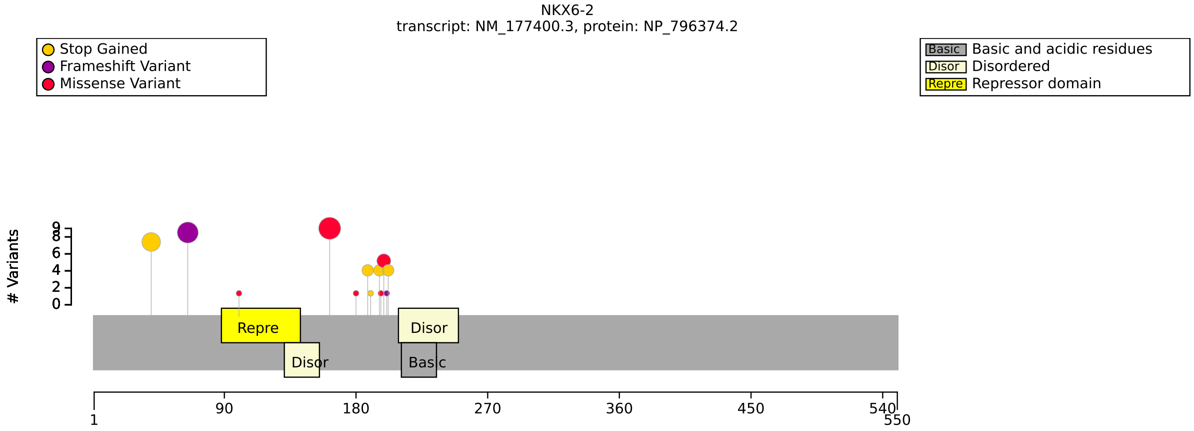

(a) Distribution of variants in *NKX6-2*

Genotype (A)	Genotype (B)	total tests performed	significant results
missense/missense	other/other OR missense/other	21	0
L163V/L163V	L163V/other OR other/other	21	0
N term/N term OR N term/other	other/other	21	0
FEMALE	MALE	21	0

(b) Fisher Exact Test performed to compare HPO annotation frequency with respect to genotypes.

Figure S45: The cohort comprised 33 individuals (15 females, 18 males). A total of 39 HPO terms were used to annotate the cohort. Disease diagnosis: Spastic ataxia 8, autosomal recessive, with hypomyelinating leukodystrophy (OMIM:617560). No statistically significant results identified. A total of 13 unique variant alleles were found in *NKX6-2* (transcript: NM_177400.3, protein id: NP_796374.2).

PIGA

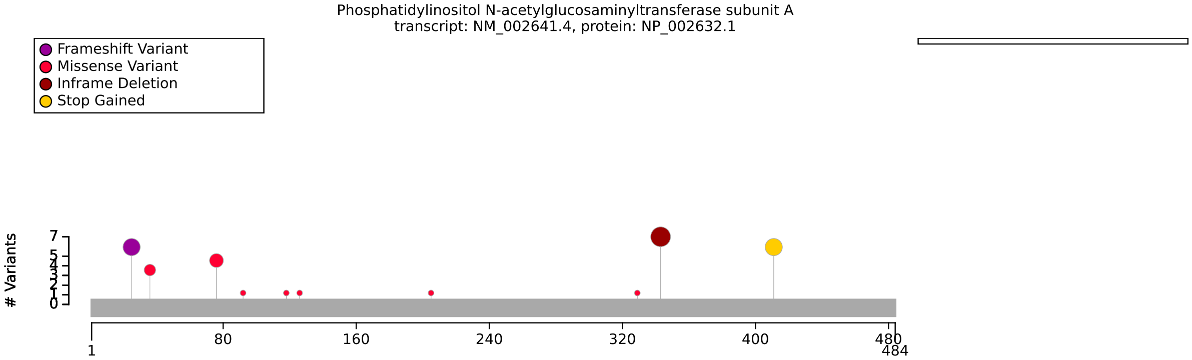

(a) Distribution of variants in *PIGA*

Genotype (A)	Genotype (B)	total tests performed	significant results
missense	other	23	0
p.Leu344del	other	20	0
1-100	100+	23	0

(b) Fisher Exact Test performed to compare HPO annotation frequency with respect to missense, p.Leu344del, and N-terminal (1-100) and other.

Figure S46: The cohort comprised 27 individuals (0 females, 27 males). A total of 188 HPO terms were used to annotate the cohort. Disease diagnoses: Multiple congenital anomalies-hypotonia-seizures syndrome 2 (OMIM:300868) (21 individuals), Neurodevelopmental disorder with epilepsy and hemochromatosis (OMIM:301072) (6 individuals). No statistically significant results identified. A total of 12 unique variant alleles were found in *PIGA* (transcript: `NM_002641.4`, protein id: `NP_002632.1`).

POGZ

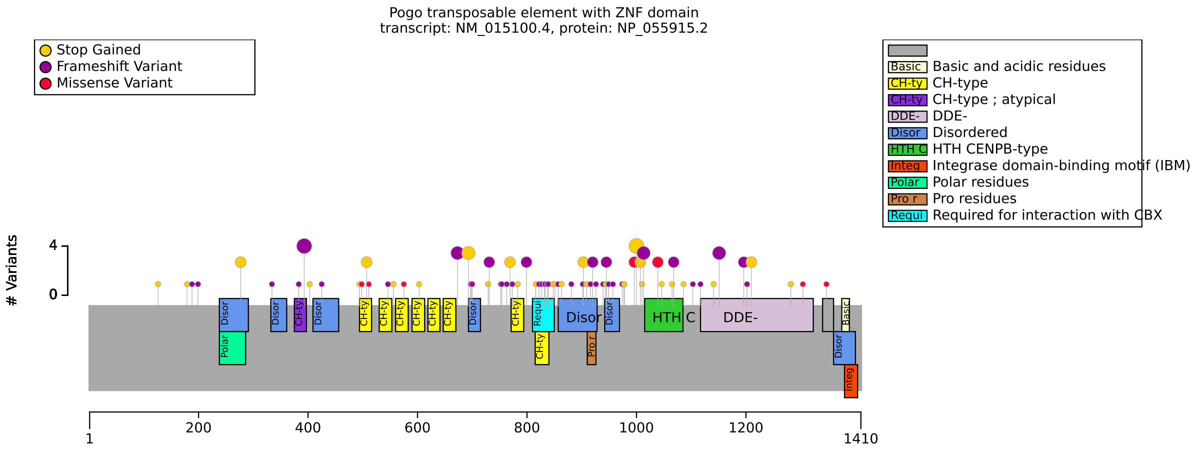

(a) Distribution of variants in *POGZ*

Genotype (A)	Genotype (B)	total tests performed	significant results
Missense	Other	24	0
Frameshift	Other	24	0

(b) Fisher Exact Test performed to compare HPO annotation frequency with respect to genotypes.

Description	Variable	Genotype (A)	Genotype (B)	p-value	xrefs
A phenotypic severity score according to Nagy et al. 2022	*POGZ* Severity Score	Missense	Other	0.429	-

(c) POGZ Severity Score to compare Missense and Other with respect to *POGZ* Severity Score.

Description	Variable	Genotype (A)	Genotype (B)	p-value	xrefs
A phenotypic severity score for individuals with intellectual disability	De Vries score	Missense	Other	0.429	-

(d) De Vries Score to compare Missense and Other with respect to De Vries score.

Figure S47: The cohort comprised 117 individuals (54 females, 62 males, 1 with unknown sex). A total of 94 HPO terms were used to annotate the cohort. Disease diagnosis: White-Sutton syndrome (OMIM:616364). Negy et al. (2022) summarized data on 117 individuals with White-Sutton syndrome. They identified a correlation between a severity score and nonsense-mediated RNA decay (NMD). Missense variants were more often associated with mild phenotypes and truncating variants predicted to escape NMD presented with more severe phenotypes. Within this group, variants in the proline-rich region of the POGZ protein were associated with the most severe phenotypes. These authors did not apply multiple testing correction [43]. Our analysis did not identify a significant GPC. A total of 90 unique variant alleles were found in *POGZ* (transcript: `NM_015100.4`, protein id: `NP_055915.2`).

PPP2R1A

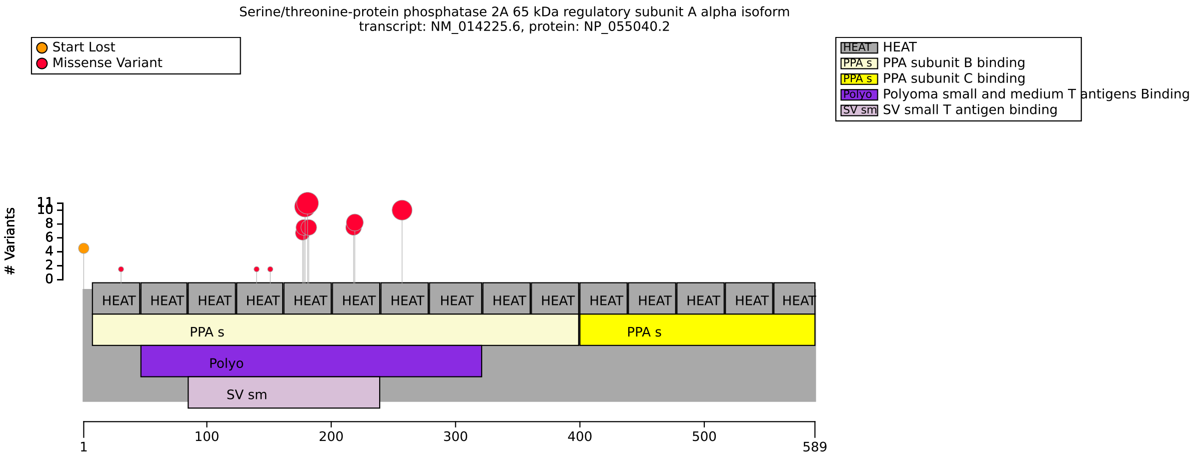

(a) Distribution of variants in *PPP2R1A*

Genotype (A)	Genotype (B)	total tests performed	significant results
Absent in COSMIC	Present in COSMIC	18	0
Arg182Trp	other	19	0
SV40 small T antigen binding	other	19	0

(b) Fisher Exact Test performed to compare HPO annotation frequency with respect to genotypes.

Figure S48: The cohort comprised 60 individuals (24 females, 32 males, 4 with unknown sex). A total of 52 HPO terms were used to annotate the cohort. Disease diagnosis: Houge-Janssen syndrome 2 (OMIM:616362). No statistically significant results identified. A total of 20 unique variant alleles were found in *PPP2R1A* (transcript: `NM_014225.6`, protein id: `NP_055040.2`).

PTPN11

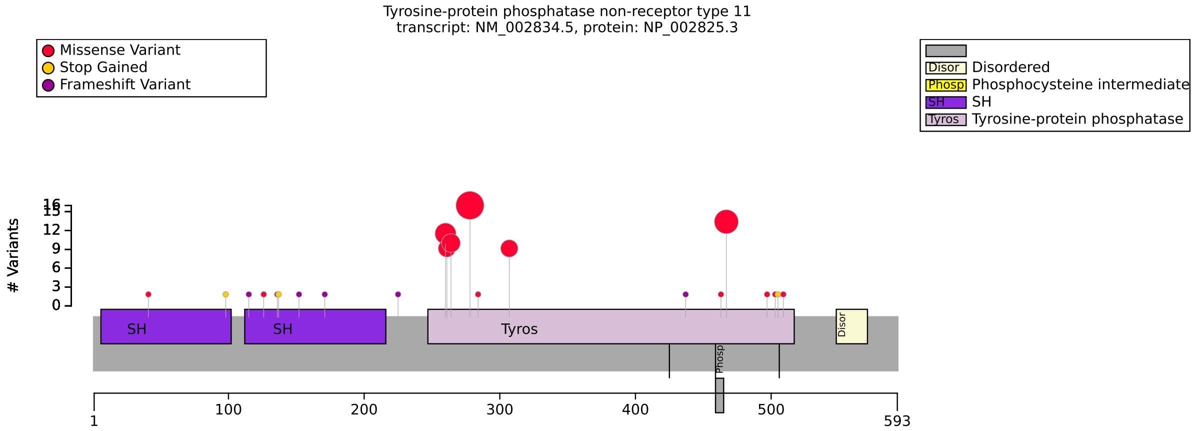

(a) Distribution of variants in *PTPN11*

HPO term	Missense	Other	p-value	adj. p-value
Hypertelorism [HP:0000316]	37/41 (90%)	0/12 (0%)	6.82×10^{-9}	2.73×10^{-8}
Intellectual disability, mild [HP:0001256]	8/23 (35%)	0/12 (0%)	0.032	0.032
Pulmonic stenosis [HP:0001642]	18/34 (53%)	0/12 (0%)	0.001	0.002
Webbed neck [HP:0000465]	15/20 (75%)	0/12 (0%)	2.94×10^{-5}	5.88×10^{-5}

(b) Fisher Exact Test performed to compare HPO annotation frequency with respect to Missense and Other. Total of 4 tests were performed.

Genotype (A)	Genotype (B)	total tests performed	significant results
Tyr279Cys	Other	8	0
TK domain N term	TK domain C term	5	0
FEMALE	MALE	6	0

(c) Fisher Exact Test performed to compare HPO annotation frequency with respect to genotypes.

Figure S49: The cohort comprised 70 individuals (27 females, 33 males, 10 with unknown sex). A total of 69 HPO terms were used to annotate the cohort. Disease diagnoses: LEOPARD syndrome 1 (OMIM:151100) (31 individuals), Noonan syndrome 1 (OMIM:163950) (27 individuals), Metachondromatosis (OMIM:156250) (12 individuals). No previous statistical analysis of correlations with *PTPN11* missense variants was identified in the medical literature. A total of 27 unique variant alleles were found in *PTPN11* (transcript: `NM_002834.5`, protein id: `NP_002825.3`).

RERE

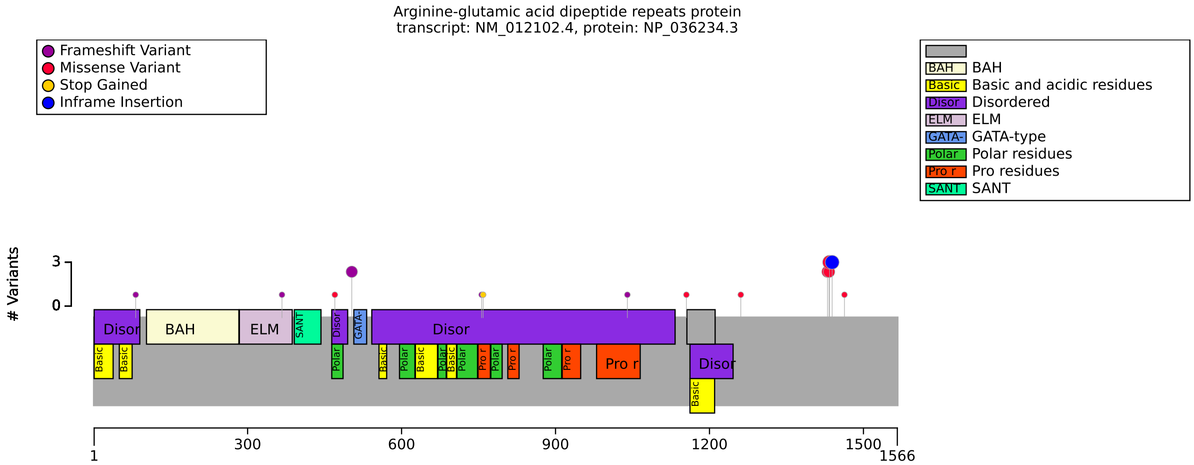

(a) Distribution of variants in *RERE*

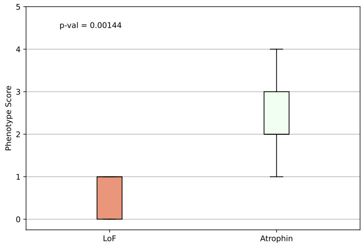

(b) Phenotype score adapted from Jordan et al. [44]. Variants in atrophin domain (residues 1425-1445) vs. others. Mann-Whitney U test.

Genotype (A)	Genotype (B)	total tests performed	significant results
LoF	Atrophin	56	0

(c) Fisher Exact Test performed to compare HPO annotation frequency with respect to genotypes.

Description	Variable	Genotype (A)	Genotype (B)	p-value	xrefs
Phenotype score (see notebook)	HPO group count	LoF	Atrophin	0.001	[44]

(d) HPO Group Count to compare LoF and Atrophin with respect to HPO group count (HP:0012443, HP:0012372,HP:0001627,HP:0012210, HP:0000407).

Figure S50: The cohort comprised 22 individuals (9 females, 13 males). A total of 115 HPO terms were used to annotate the cohort. Disease diagnosis: Neurodevelopmental disorder with or without anomalies of the brain, eye, or heart (OMIM:616975). Our results recapitulate the results of Jordan et al. [44] that The total number of structural defects and sensorineural hearing loss diagnoses seen in individuals with point mutations in the Atrophin-1 domain is significantly higher than expected based on the number of similar defects seen in individuals with putative loss-of-function variants. A total of 18 unique variant alleles were found in *RERE* (transcript: `NM_012102.4`, protein id: `NP_036234.3`).

RNU4-2

Genotype (A)	Genotype (B)	total tests performed	significant results
n.64_65insT	other	176	0
insertion	other	176	0
FEMALE	MALE	176	0

(a) Fisher Exact Test performed to compare HPO annotation frequency with respect to genotypes.

Figure S51: The cohort comprised 61 individuals (28 females, 33 males). A total of 209 HPO terms were used to annotate the cohort. Disease diagnosis: ReNU syndrome (OMIM:620851). No previous statistical analysis of correlations with *RNU4-2* missense variants identified in the medical literature. A total of 7 unique variant alleles were found in *RNU4-2* (transcript: `NR_003137.3`, protein id:).

Robinow syndrome

HPO term	OMIM:268310	OMIM:616331	p-value	adj. p-value
Mesomelia [HP:0003027]	31/31 (100%)	10/15 (67%)	0.002	0.048
Hearing impairment [HP:0000365]	3/22 (14%)	7/7 (100%)	7.69×10^{-5}	0.003
Short stature [HP:0004322]	29/29 (100%)	3/11 (27%)	2.15×10^{-6}	1.89×10^{-4}
Cleft palate [HP:0000175]	0/17 (0%)	5/8 (62%)	0.001	0.031

(a) Fisher Exact Test performed to compare HPO annotation frequency with respect to Robinow syndrome, autosomal recessive (OMIM:268310) and Robinow syndrome, autosomal dominant 2 (OMIM:616331). Total of 88 tests were performed.

Figure S52: The cohort comprised 48 individuals (10 females, 14 males, 24 with unknown sex). A total of 103 HPO terms were used to annotate the cohort. Disease diagnoses: Robinow syndrome, autosomal recessive (OMIM:268310) (32 individuals), Robinow syndrome, autosomal dominant 2 (OMIM:616331) (16 individuals). Robinow syndrome is a skeletal dysplasia characterized by dysmorphic facial features, short-limbed dwarfism, vertebral segmentation, and genital hypoplasia. A total of 44 unique variant alleles were found.

ROR2

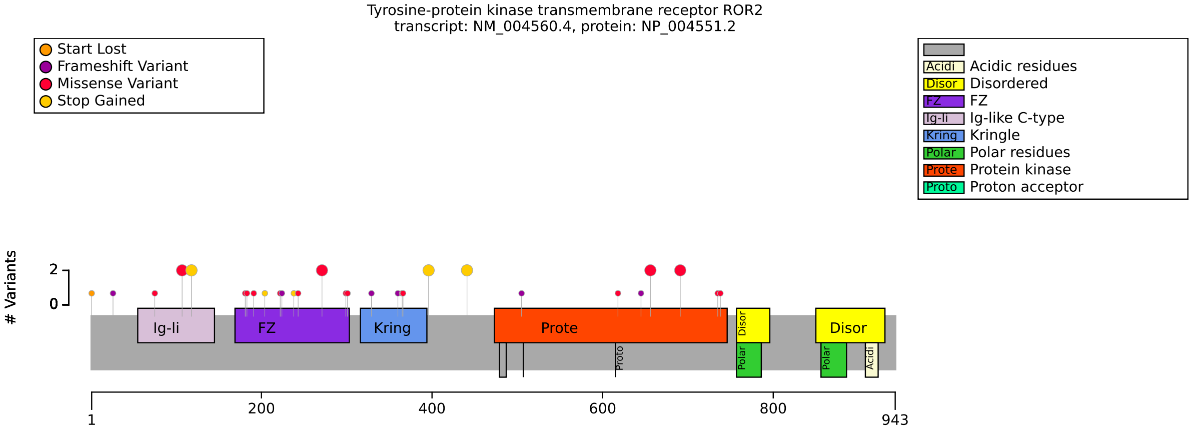

(a) Distribution of variants in *ROR2*

Genotype (A)	Genotype (B)	total tests performed	significant results
missense/missense OR missense/other	other/other	116	0
FZ domain/FZ domain OR FZ domain/other	other/other	116	0

(b) Fisher Exact Test performed to compare HPO annotation frequency with respect to genotypes.

Figure S53: The cohort comprised 32 individuals (3 females, 7 males, 22 with unknown sex). A total of 81 HPO terms were used to annotate the cohort. Disease diagnosis: Robinow syndrome, autosomal recessive (OMIM:268310). No statistically significant genotype-phenotype correlation was identified. A total of 32 unique variant alleles were found in *ROR2* (transcript: `NM_004560.4`, protein id: `NP_004551.2`).

RPGRIP1

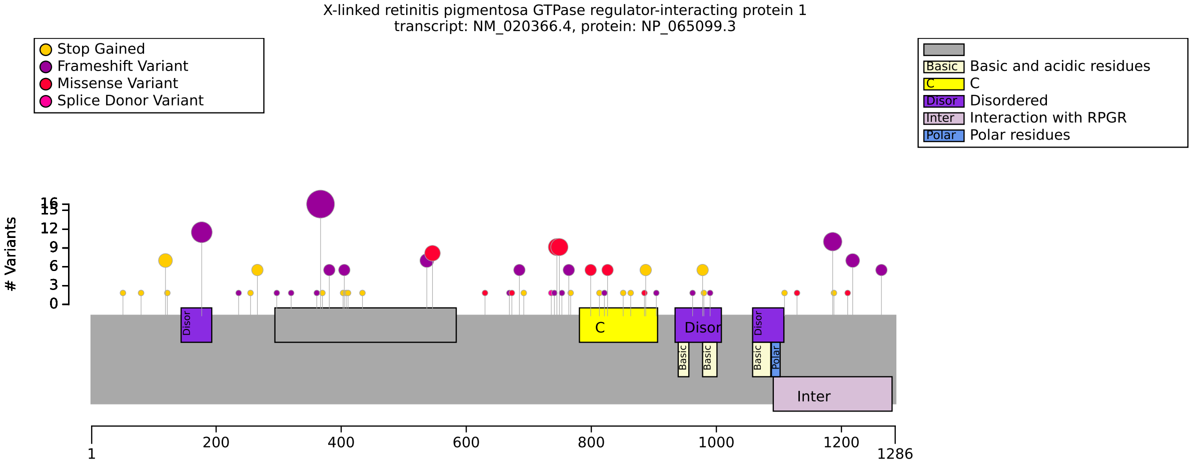

(a) Distribution of variants in *RPGRIP1*

HPO term	1107del/1107del OR 1107del/other	other/other	p-value	adj. p-value
Eye poking [HP:0001483]	16/16 (100%)	19/41 (46%)	1.30×10^{-4}	0.002

(b) Fisher Exact Test performed to compare HPO annotation frequency with respect to 1107del/1107del OR 1107del/other and other/other. Total of 16 tests were performed.

HPO term	OMIM:613826	OMIM:608194	p-value	adj. p-value
Nystagmus [HP:0000639]	64/66 (97%)	11/16 (69%)	0.003	0.020
Very low visual acuity [HP:0032122]	35/39 (90%)	4/16 (25%)	5.21×10^{-6}	7.81×10^{-5}

(c) Fisher Exact Test performed to compare HPO annotation frequency with respect to OMIM:613826 and OMIM:608194. Total of 15 tests were performed.

Genotype (A)	Genotype (B)	total tests performed	significant results
missense/missense	missense/other OR other/other	17	0
frameshift/frameshift	frameshift/other OR other/other	16	0
FEMALE	MALE	16	0

(d) Fisher Exact Test performed to compare HPO annotation frequency with respect to genotypes.

Figure S54: The cohort comprised 100 individuals (45 females, 49 males, 6 with unknown sex). A total of 45 HPO terms were used to annotate the cohort. Disease diagnoses: Leber congenital amaurosis 6 (OMIM:613826) (79 individuals), Cone-rod dystrophy 13 (OMIM:608194) (21 individuals). It was reported that Patients with a double null genotype may develop symptoms earlier and have worse vision [45]. We did not observe a corresponding significant correlation in our dataset. A total of 71 unique variant alleles were found in *RPGRIP1* (transcript: `NM_020366.4`, protein id: `NP_065099.3`).

SAMD9L

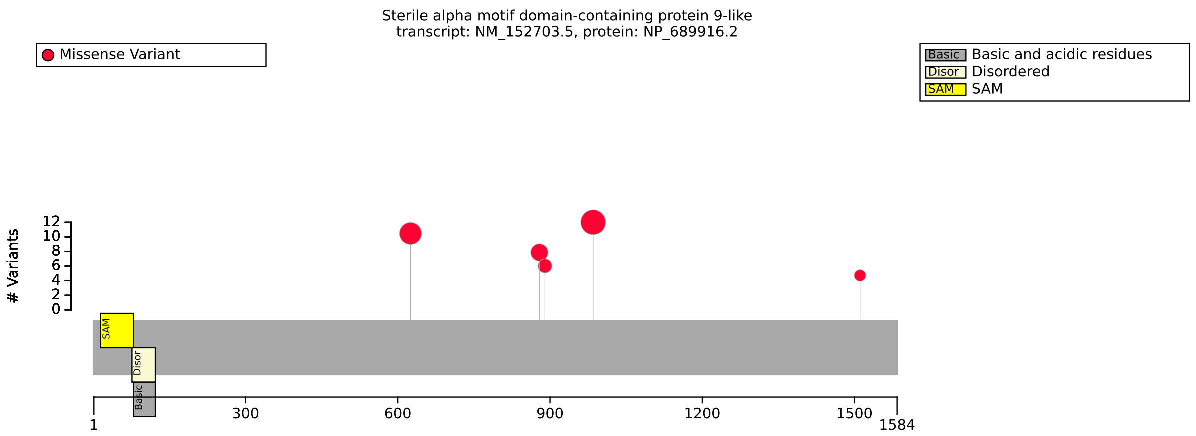

(**a**) Distribution of variants in *SAMD9L*

HPO term	Arg986Cys	Ser626Leu	p-value	adj. p-value
Pancytopenia [HP:0001876]	4/6 (67%)	0/9 (0%)	0.011	0.026
Thrombocytopenia [HP:0001873]	7/9 (78%)	0/9 (0%)	0.002	0.008
Neutropenia [HP:0001875]	7/9 (78%)	0/9 (0%)	0.002	0.008

(**b**) Fisher Exact Test performed to compare HPO annotation frequency with respect to Arg986Cys and Ser626Leu. Total of 7 tests were performed.

Genotype (A)	Genotype (B)	total tests performed	significant results
N Term	other	16	0

(**c**) Fisher Exact Test performed to compare HPO annotation frequency with respect to genotypes.

Figure S55: The cohort comprised 31 individuals (15 females, 16 males). 5 of these individuals were reported to be deceased. A total of 41 HPO terms were used to annotate the cohort. Disease diagnoses: Ataxia-pancytopenia syndrome (OMIM:159550) (22 individuals), Spinocerebellar ataxia 49 (OMIM:619806) (9 individuals). A recent summary of *SAMD9L* variants stated there was no evidence of phenotype–genotype correlation [46]. A total of 6 unique variant alleles were found in *SAMD9L* (transcript: `NM_152703.5`, protein id: `NP_689916.2`).

SATB2

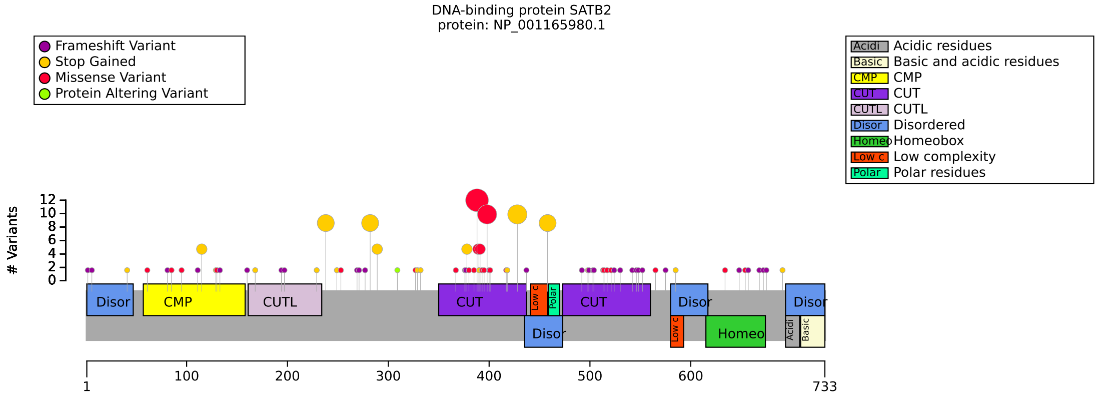

(a) Distribution of variants in *SATB2*

HPO term	Missense	Other	p-value	adj. p-value
Cleft palate [HP:0000175]	11/49 (22%)	59/105 (56%)	1.11×10^{-4}	0.002

(b) Fisher Exact Test performed to compare HPO annotation frequency with respect to Missense and Other. Total of 20 tests were performed.

Genotype (A)	Genotype (B)	total tests performed	significant results
ULD	Other	20	0
FEMALE	MALE	20	0
transcript ablation	other	20	0

(c) Fisher Exact Test performed to compare HPO annotation frequency with respect to genotypes.

Figure S56: The cohort comprised 158 individuals (62 females, 90 males, 6 with unknown sex). A total of 11 HPO terms were used to annotate the cohort. Disease diagnosis: Glass syndrome (OMIM:612313). Individuals with large chromosomal deletions were diagnosed at earlier ages (mean 2.5 years, $p \leq 0.0006$). Individuals with missense or disruptive pathogenic variants were more commonly reported to have sialorrhea (p = 0.0115), and those with large deletions were more likely to have a history of growth retardation (p = 0.0033). The authors reported a higher prevalence of chronic mucocutanous candidiasis with the variant Arg357Ter than with other variants. We did not identify a significant difference in prevalence [47]. A total of 92 unique variant alleles were found in *SATB2* (transcript: `NM_001172509.2`, protein id: `NP_001165980.1`).

SCN2A

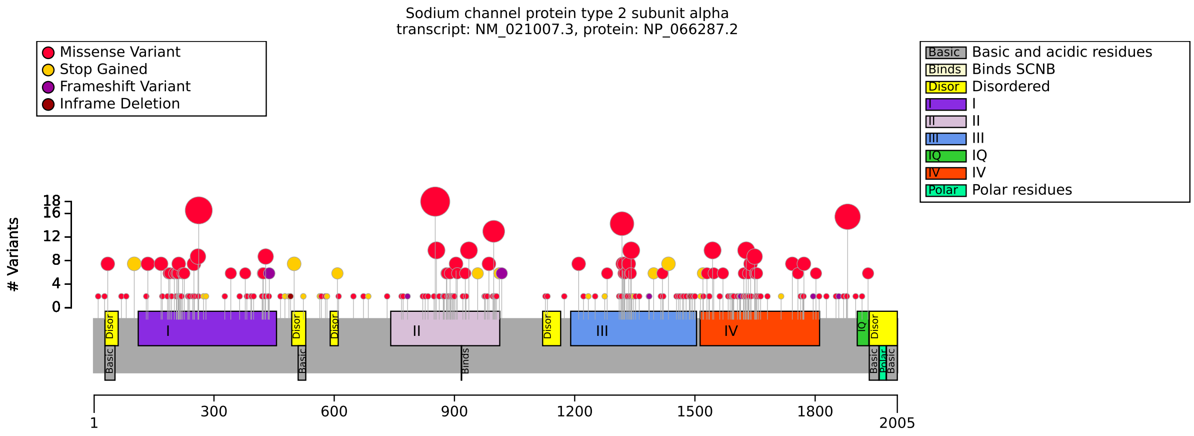

(a) Distribution of variants in *SCN2A*

HPO term	Missense	Other	p-value	adj. p-value
Neurodevelopmental abnormality [HP:0012759]	201/238 (84%)	45/45 (100%)	0.001	0.003
Motor seizure [HP:0020219]	146/175 (83%)	6/31 (19%)	4.51×10^{-12}	7.22×10^{-11}
Seizure [HP:0001250]	298/327 (91%)	28/53 (53%)	1.57×10^{-10}	8.38×10^{-10}
Autism [HP:0000717]	59/146 (40%)	33/43 (77%)	2.69×10^{-5}	8.61×10^{-5}
Focal-onset seizure [HP:0007359]	141/170 (83%)	8/33 (24%)	8.66×10^{-11}	6.93×10^{-10}
Generalized-onset seizure [HP:0002197]	104/133 (78%)	6/31 (19%)	1.43×10^{-9}	5.72×10^{-9}
Intellectual disability [HP:0001249]	144/198 (73%)	34/34 (100%)	9.39×10^{-5}	2.50×10^{-4}

(b) Fisher Exact Test performed to compare HPO annotation frequency with respect to Missense and Other. Total of 16 tests were performed.

HPO term	I repeat	Other	p-value	adj. p-value
Neurodevelopmental abnormality [HP:0012759]	48/65 (74%)	198/218 (91%)	0.001	0.009
Intellectual disability [HP:0001249]	21/42 (50%)	157/190 (83%)	2.59×10^{-5}	4.15×10^{-4}

(c) Fisher Exact Test performed to compare HPO annotation frequency with respect to I repeat and Other. Total of 16 tests were performed.

Genotype (A)	Genotype (B)	total tests performed	significant results
Arg853Gln	Other	16	0
Exon 27	Other	16	0

(d) Fisher Exact Test performed to compare HPO annotation frequency with respect to genotypes.

Figure S57: The cohort comprised 393 individuals (0 females, 0 males, 393 with unknown sex). A total of 289 HPO terms were used to annotate the cohort. Disease diagnoses: Developmental and epileptic encephalopathy 11 (OMIM:613721) (342 individuals), Seizures, benign familial infantile, 3 (OMIM:607745) (51 individuals). Similar genotype-phenotype correlations have been previously published [48]. A total of 264 unique variant alleles were found in *SCN2A* (transcript: `NM_021007.3`, protein id: `NP_066287.2`).

SCO2

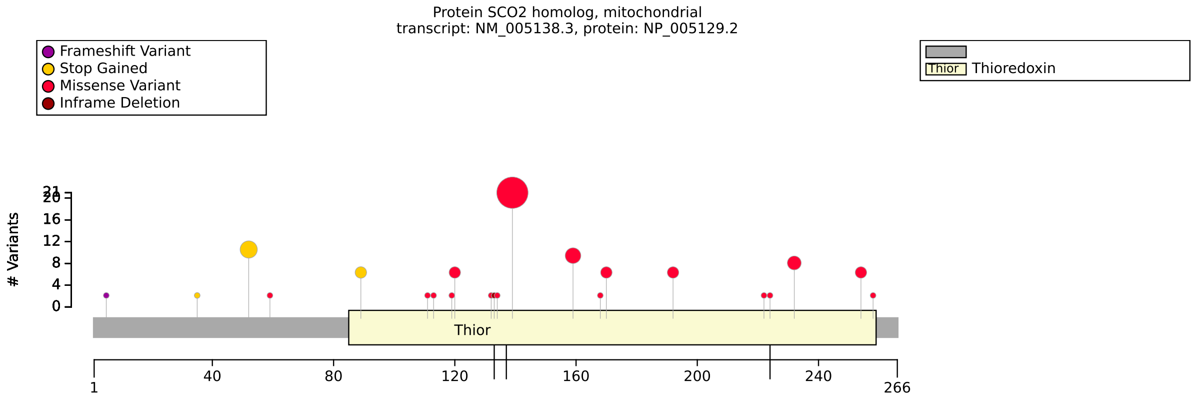

(a) Distribution of variants in *SCO2*

HPO term	Glu140Lys/Glu140Lys	other/other OR Glu140Lys/other	p-value	adj. p-value
Hypertrophic cardiomyopathy [HP:0001639]	2/6 (33%)	13/13 (100%)	0.004	0.027

(b) Fisher Exact Test performed to compare HPO annotation frequency with respect to Glu140Lys/Glu140Lys and other/other OR Glu140Lys/other. Total of 7 tests were performed.

Description	Variable	Genotype (A)	Genotype (B)	p-value	xrefs
Survival analysis: Hypertrophic cardiomyopathy	Onset of HP:0001639	Glu140Lys/Glu140Lys	other/other OR Glu140Lys/other	0.219	-

(c) Onset of Hypertrophic cardiomyopathy to compare Glu140Lys/Glu140Lys and other/other OR Glu140Lys/other with respect to Onset of HP:0001639.

Description	Variable	Genotype (A)	Genotype (B)	p-value	xrefs
Compute time until OMIM:604377 onset	Onset of OMIM:604377	Glu140Lys/Glu140Lys	other/other OR Glu140Lys/other	0.415	-

(d) Onset of OMIM:604377 to compare Glu140Lys/Glu140Lys and other/other OR Glu140Lys/other with respect to Onset of OMIM:604377.

Figure S58: The cohort comprised 37 individuals (10 females, 23 males, 4 with unknown sex). 24 of these individuals were reported to be deceased. A total of 107 HPO terms were used to annotate the cohort. Disease diagnoses: Mitochondrial complex IV deficiency, nuclear type 2 (OMIM:604377) (31 individuals), Myopia 6 (OMIM:608908) (6 individuals). The glu140lys correlation is driven by 5 patients found to be homozygous for the Glu140Lys variant [49]. The children were between the age of 8 months and 1 year and 8 months. Because of the relatively small cohort and the fact that other members of the cohort were not as young, caution is advised in the interpretation of this finding. A total of 25 unique variant alleles were found in *SCO2* (transcript: `NM_005138.3`, protein id: `NP_005129.2`).

SEC61A1

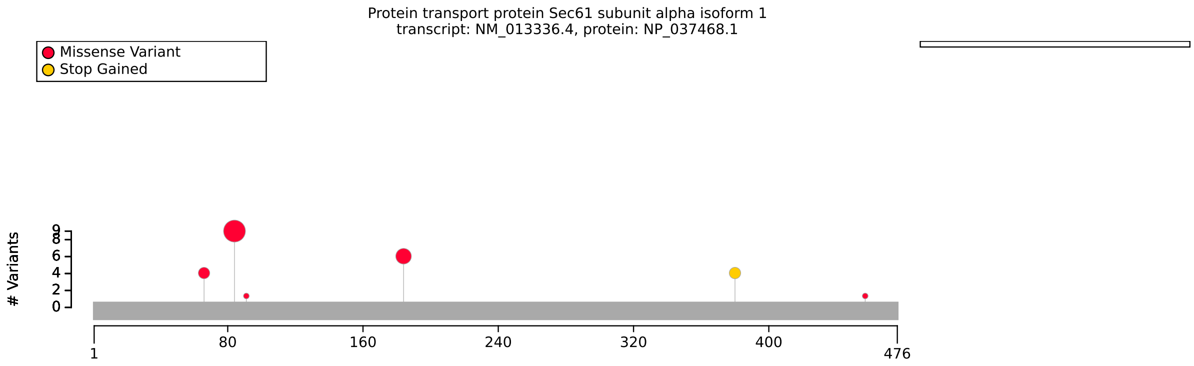

(a) Distribution of variants in *SEC61A1*

Genotype (A)	Genotype (B)	total tests performed	significant results
p.Val85Asp	Other variant	24	0
FEMALE	MALE	30	0

(b) Fisher Exact Test performed to compare HPO annotation frequency with respect to genotypes.

Figure S59: The cohort comprised 19 individuals (8 females, 11 males). 1 of these individuals were reported to be deceased. A total of 76 HPO terms were used to annotate the cohort. Disease diagnoses: Immunodeficiency, common variable, 15 (OMIM:620670) (11 individuals), Tubulointerstitial kidney disease, autosomal dominant, 5 (OMIM:617056) (7 individuals), Neutropenia, severe congenital, 11, autosomal dominant (OMIM:620674) (1 individuals). The origin of clinical diversity in patients with *SEC61A1* mutation is currently unclear. With our present patient set, a particular phenotype cannot be predicted on the basis of location or nature of the mutation [50]. A total of 6 unique variant alleles were found in *SEC61A1* (transcript: `NM_013336.4`, protein id: `NP_037468.1`).

SETD2

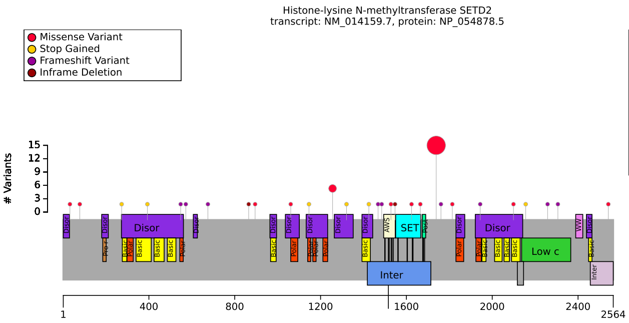

(a) Distribution of variants in *SETD2*

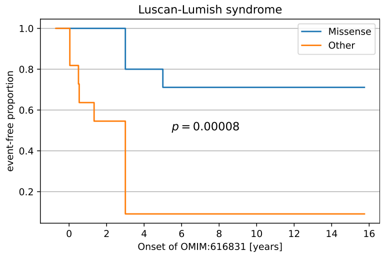

(b) Luscan-Lumish syndrome onset.

HPO term	Arg1740Trp	Other	p-value	adj. p-value
Macrocephaly [HP:0000256]	0/11 (0%)	19/28 (68%)	1.45×10^{-4}	0.002
Hypertelorism [HP:0000316]	11/11 (100%)	5/23 (22%)	1.53×10^{-5}	2.60×10^{-4}
Wide nasal bridge [HP:0000431]	9/9 (100%)	2/9 (22%)	0.002	0.012
Ventriculomegaly [HP:0002119]	4/4 (100%)	2/17 (12%)	0.003	0.012
Severe global developmental delay [HP:0011344]	9/9 (100%)	0/12 (0%)	3.40×10^{-6}	1.16×10^{-4}
Delayed ability to walk [HP:0031936]	8/8 (100%)	1/10 (10%)	4.11×10^{-4}	0.003
Scoliosis [HP:0002650]	6/6 (100%)	2/14 (14%)	7.22×10^{-4}	0.005

(c) Fisher Exact Test:HPO annotation frequency vs. Arg1740Trp / Other. Total of 34 tests were performed.

HPO term	Missense	Other	p-value	adj. p-value
Macrocephaly [HP:0000256]	4/24 (17%)	15/15 (100%)	1.54×10^{-7}	6.32×10^{-6}

(d) Fisher Exact Test: HPO annotation frequency vs. Missense / Other. Total of 41 tests were performed.

Genotype (A)	Genotype (B)	total tests performed	significant results
FEMALE	MALE	41	0

(e) Fisher Exact Test performed to compare HPO annotation frequency with respect to genotypes.

Description	Variable	Genotype (A)	Genotype (B)	p-value	xrefs
Luscan-Lumish syndrome (OMIM:616831) onset	Onset of OMIM:616831	Missense	Other	8.47×10^{-5}	-

(f) Onset of OMIM:616831 to compare Missense and Other variant.

Figure S60: The cohort comprised 45 individuals (18 females, 27 males). 1 of these individuals was reported to be deceased. A total of 202 HPO terms were used to annotate the cohort. Disease diagnoses: Luscan-Lumish syndrome (OMIM:616831) (28 individuals), Rabin-Pappas syndrome (OMIM:620155) (14 individuals), Intellectual developmental disorder, autosomal dominant 70 (OMIM:620157) (3 individuals). Van Nieuwenhove et al. (2020) stated that the origin of clinical diversity in patients with SEC61A1 mutation is currently unclear [50]. Rabin et al. (2023) found that variants in codon 1740 of *SETD2* whose features differ from those with LLS [51]. A total of 31 unique variant alleles were found in *SETD2* (transcript: `NM_014159.7`, protein id: `NP_054878.5`).

SF3B4

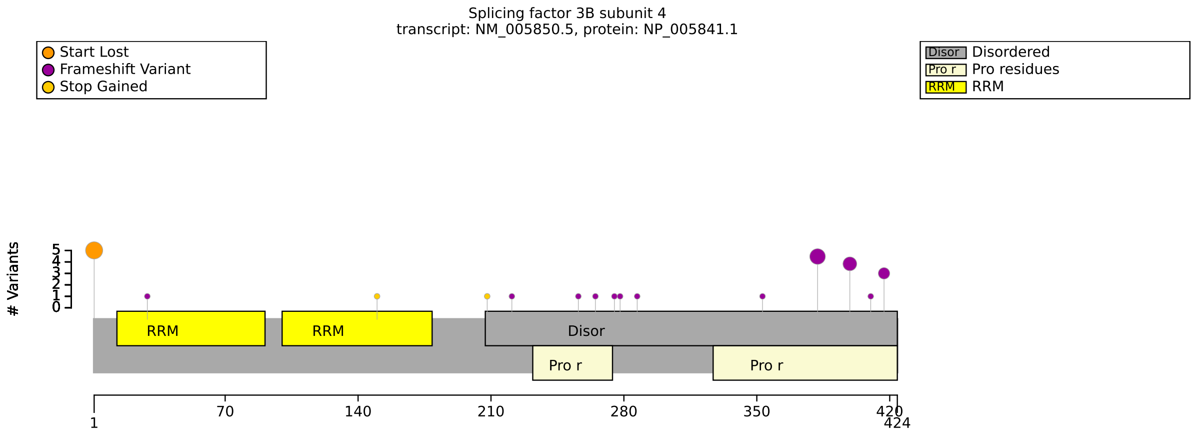

(a) Distribution of variants in *SF3B4*

Genotype (A)	Genotype (B)	total tests performed	significant results
p.Met1?	Other	46	0
FEMALE	MALE	46	0

(b) Fisher Exact Test performed to compare HPO annotation frequency with respect to genotypes.

Figure S61: The cohort comprised 26 individuals (18 females, 8 males). A total of 41 HPO terms were used to annotate the cohort. Disease diagnosis: Acrofacial dysostosis 1, Nager type (OMIM:154400). In one published analysis, it was stated that "although no significant genotype–phenotype association was found, it is notable that patients with frameshift *SF3B4* variants and predicted to lead to nonsense-mediated RNA decay (NMD) of the transcripts tended to have a more severe clinical manifestation". The authors did not correct for multiple testing and did not find nominally significant associations [52]. No significant correlation found in our study. A total of 18 unique variant alleles were found in *SF3B4* (transcript: NM_005850.5, protein id: NP_005841.1).

SLC4A1

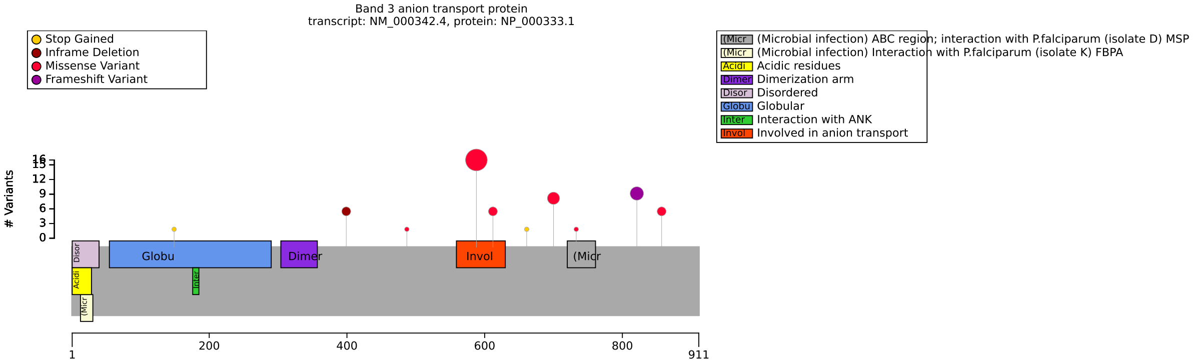

(a) Distribution of variants in *SLC4A1*

Genotype (A)	Genotype (B)	total tests performed	significant results
r149w	other	14	0

(b) Fisher Exact Test performed to compare HPO annotation frequency with respect to genotypes.

Figure S62: The cohort comprised 33 individuals (16 females, 16 males, 1 with unknown sex). A total of 49 HPO terms were used to annotate the cohort. Disease diagnoses: Distal renal tubular acidosis 1 (OMIM:179800) (18 individuals), Spherocytosis, type 4 (OMIM:612653) (7 individuals), Distal renal tubular acidosis 4 with hemolytic anemia (OMIM:611590) (7 individuals), Cryohydrocytosis (OMIM:185020) (1 individuals). No statistically significant results identified. A total of 11 unique variant alleles were found in *SLC4A1* (transcript: NM_000342.4, protein id: NP_000333.1).

SLC32A1

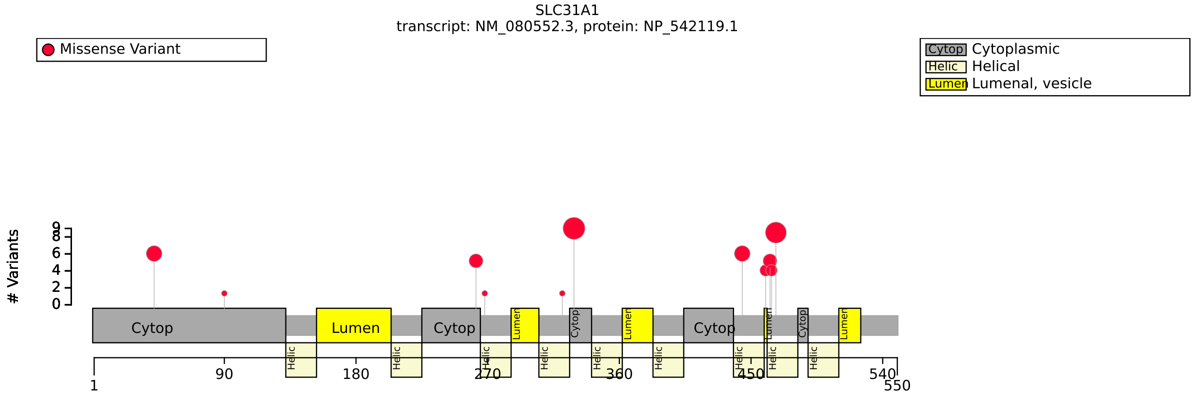

(a) Distribution of variants in *SLC32A1*

Genotype (A)	Genotype (B)	total tests performed	significant results
N term	other	11	0
p.Met330Thr	Other variant	11	0
FEMALE	MALE	11	0

(b) Fisher Exact Test performed to compare HPO annotation frequency with respect to genotypes.

Figure S63: The cohort comprised 38 individuals (19 females, 19 males). A total of 44 HPO terms were used to annotate the cohort. Disease diagnoses: Generalized epilepsy with febrile seizures plus, type 12 (OMIM:620755) (34 individuals), Developmental and epileptic encephalopathy 114 (OMIM:620774) (4 individuals). No significant correlation identified. A total of 12 unique variant alleles were found in *SLC32A1* (transcript: `NM_080552.3`, protein id: `NP_542119.1`).

SLC45A2

Genotype (A)	Genotype (B)	total tests performed	significant results
missense/missense OR missense/other	other/other	24	0
FEMALE	MALE	25	0

(a) Fisher Exact Test performed to compare HPO annotation frequency with respect to genotypes.

Figure S64: The cohort comprised 30 individuals (17 females, 13 males). A total of 16 HPO terms were used to annotate the cohort. Disease diagnosis: Albinism, oculocutaneous, type IV (OMIM:606574). No significant association identified. A total of 28 unique variant alleles were found in *SLC45A2* (transcript: `NM_016180.5`, protein id: `NP_057264.4`).

SMAD2

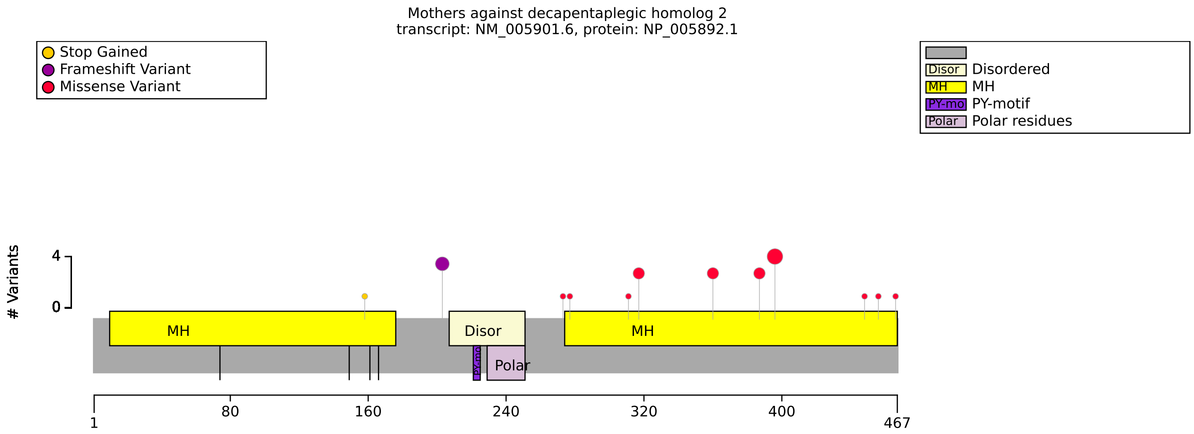

(a) Distribution of variants in *SMAD2*

Genotype (A)	Genotype (B)	total tests performed	significant results
Ser397Tyr	Other	26	0
N Term	Other	27	0
FEMALE	MALE	27	0

(b) Fisher Exact Test performed to compare HPO annotation frequency with respect to genotypes.

Figure S65: The cohort comprised 23 individuals (13 females, 8 males, 2 with unknown sex). A total of 89 HPO terms were used to annotate the cohort. Disease diagnoses: Loeys-Dietz syndrome 6 (OMIM:619656) (18 individuals), Congenital heart defects, multiple types, 8, with or without heterotaxy (OMIM:619657) (5 individuals). No significant correlations with specific *SMAD2* residues or variant categories were identified. A total of 15 unique variant alleles were found in *SMAD2* (transcript: `NM_005901.6`, protein id: `NP_005892.1`).

SMAD3

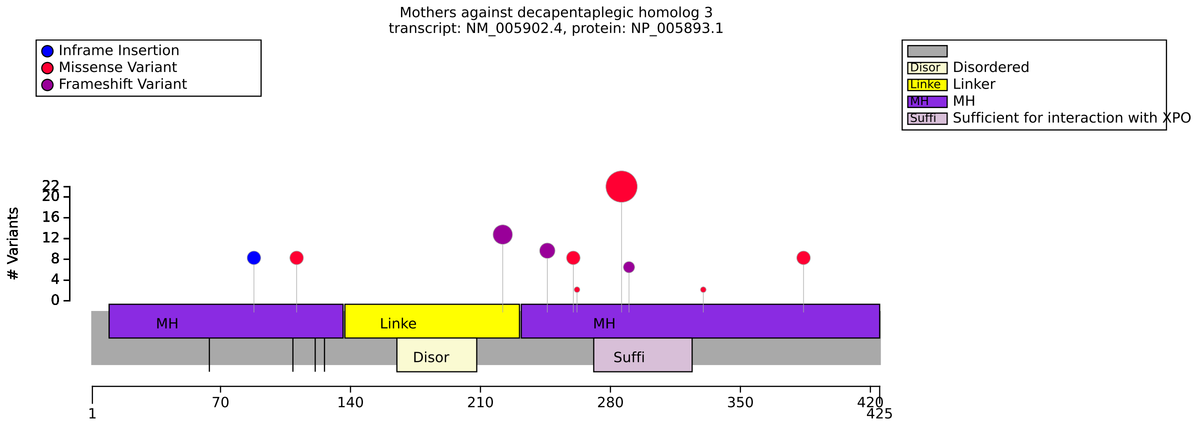

(a) Distribution of variants in *SMAD3*

HPO term	p.Arg287Trp	Other	p-value	adj. p-value
Osteoarthritis [HP:0002758]	19/19 (100%)	7/19 (37%)	3.72×10^{-5}	8.56×10^{-4}

(b) Fisher Exact Test performed to compare HPO annotation frequency with respect to p.Arg287Trp and Other. Total of 23 tests were performed.

Genotype (A)	Genotype (B)	total tests performed	significant results
missense	other	31	0
FEMALE	MALE	31	0

(c) Fisher Exact Test performed to compare HPO annotation frequency with respect to genotypes.

Figure S66: The cohort comprised 49 individuals (22 females, 27 males). A total of 30 HPO terms were used to annotate the cohort. Disease diagnosis: Loeys-Dietz syndrome 3 (OMIM:613795). There was no evidence of a correlation between missense variants and specific phenotypic abnormalities. In contrast, there was a statistically significant correlation with Arg287Trp and Osteoarthritis. The residue Arg287 is located in the Mad homology 2 (MH2) domain in a region that mediates interaction with exportin 4 [53]. Chesneau et al stated that there is an absence of correlation between the *SMAD3* variant type and the occurrence of aortic phenotypes [54]. To the best of our knowledge an association between Arg287Trp and Osteoarthritis has not been previously noted. A total of 10 unique variant alleles were found in *SMAD3* (transcript: `NM_005902.4`, protein id: `NP_005893.1`).

SMARCB1

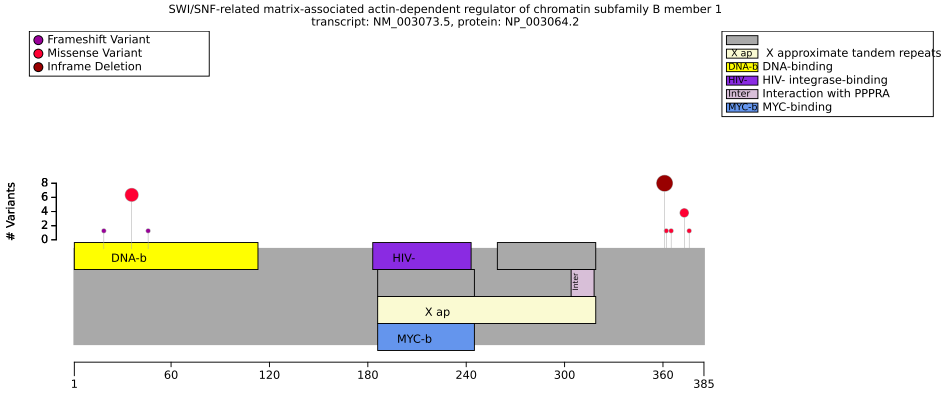

(a) Distribution of variants in *SMARCB1*

HPO term	Structural variant	Other	p-value	adj. p-value
Atypical teratoid/rhabdoid tumor [HP:0034401]	8/9 (89%)	2/19 (11%)	1.19×10^{-4}	5.94×10^{-4}
Embryonal neoplasm [HP:0002898]	8/8 (100%)	2/19 (11%)	2.03×10^{-5}	1.52×10^{-4}
Neoplasm by histology [HP:0011792]	11/11 (100%)	4/21 (19%)	1.06×10^{-5}	1.52×10^{-4}
Rhabdoid tumor [HP:0034557]	4/4 (100%)	2/19 (11%)	0.002	0.006
Neoplasm by anatomical site [HP:0011793]	3/3 (100%)	3/20 (15%)	0.011	0.028
Neuroepithelial neoplasm [HP:0030063]	2/2 (100%)	0/17 (0%)	0.006	0.018

(b) Fisher Exact Test. Total of 15 tests were performed.

Genotype (A)	Genotype (B)	total tests performed	significant results
Lys364del	Other	52	0
DNA binding	Other	51	0

(c) Fisher Exact Test performed to compare HPO annotation frequency with respect to genotypes.

Figure S67: The cohort comprised 32 individuals (12 females, 8 males, 12 with unknown sex). 9 of these individuals were reported to be deceased. A total of 110 HPO terms were used to annotate the cohort. Disease diagnoses: Coffin-Siris syndrome 3 (OMIM:614608) (18 individuals), Rhabdoid tumor predisposition syndrome 1 (OMIM:609322) (14 individuals). Our analysis of *SMARCB1* included variants associated with Coffin-Siris syndrome 3 (OMIM:614608). A total of 11 unique variant alleles were found in *SMARCB1* (transcript: `NM_003073.5`, protein id: `NP_003064.2`). Presumably the result of embryonal neoplasm being signficantly associated with structural variants relates to a different distribution of variants in rhabdoid tumor predisposition syndrome-1 than in Coffin-Siris syndrome 3. A published analysis of GPCs in Coffin-Siris syndrome did not present a statistical analysis and did not note the current association [55].

SMARCC2

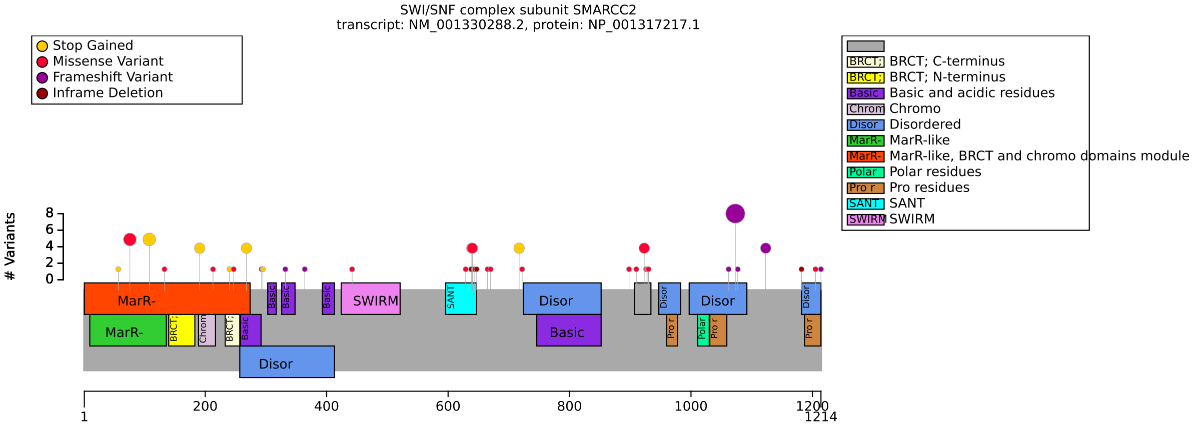

(a) Distribution of variants in *SMARCC2*

HPO term	c.3222del	other	p-value	adj. p-value
Intellectual disability [HP:0001249]	1/6 (17%)	49/52 (94%)	6.99×10^{-5}	0.006

(b) Fisher Exact Test performed to compare HPO annotation frequency with respect to c.3222del and other. Total of 83 tests were performed.

Genotype (A)	Genotype (B)	total tests performed	significant results
Missense	Truncating	72	0
N term	Other	83	0
FEMALE	MALE	83	0

(c) Fisher Exact Test performed to compare HPO annotation frequency with respect to genotypes.

Figure S68: The cohort comprised 65 individuals (24 females, 37 males, 4 with unknown sex). A total of 118 HPO terms were used to annotate the cohort. Disease diagnosis: Coffin-Siris syndrome 8 (OMIM:618362). Bosch et al. (2023) reported correlations for missense versus truncating variant cohorts for Global developmental delay; Intellectual disability (HP:0001263;HP:0001249): p = 0.005 (FDR-corr: 0.033); Muscular hypotonia (HP:0001252): p = 0.004 (FDR-corr: 0.033); mild GDD/ID (HP:0011342;HP:0001256): p = 0.012 (FDR-corr: 0.051); Abnormality of the outer ear (HP:0000356): p = 0.013 (FDR-corr: 0.051); Decreased body weight (HP:0004325): p = 0.002 (FDR-corr: 0.024); Abnormality of the eye (HP:0000478): p = 0.000 (FDR-corr: 0.008); Short stature (HP:0004322): p = 0.006 (FDR-corr: 0.035); Feeding difficulties/failure to thrive (HP:0011968;HP:0001508): p = 0.013 (FDR-corr: 0.051) [56]. The authors did not specifically test c.3222del. Differences in results may be due to different assumptions of the analysis procedure. Code to reproduce the results in Bosch et al. was not available. A total of 46 unique variant alleles were found in *SMARCC2* (transcript: `NM_001330288.2`, protein id: `NP_001317217.1`).

SON

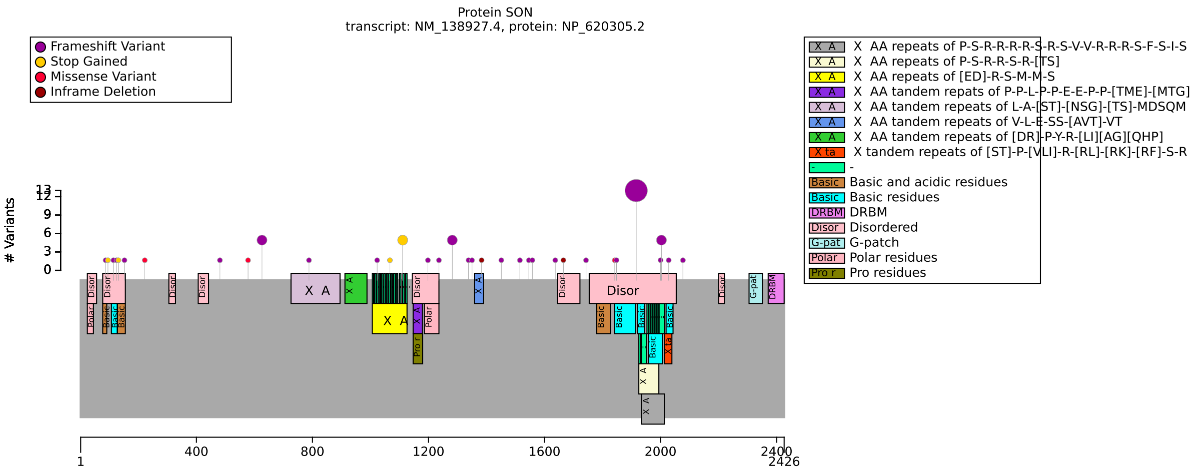

(a) Distribution of variants in *SON*

Genotype (A)	Genotype (B)	total tests performed	significant results
missense	Other	48	0
c.5753_5756del	Other	51	0
FEMALE	MALE	51	0

(b) Fisher Exact Test performed to compare HPO annotation frequency with respect to genotypes.

Figure S69: The cohort comprised 52 individuals (26 females, 26 males). A total of 47 HPO terms were used to annotate the cohort. Disease diagnosis: ZTTK SYNDROME (OMIM:617140). Dingemans et al (2020) suggested a different pathomechanism for missense variants, but our cohort only contains 3 individuals with missense variants, so there is no statistical power [57]. A total of 35 unique variant alleles were found in *SON* (transcript: `NM_138927.4`, protein id: `NP_620305.2`).

SPTAN1 (Part 1/2)

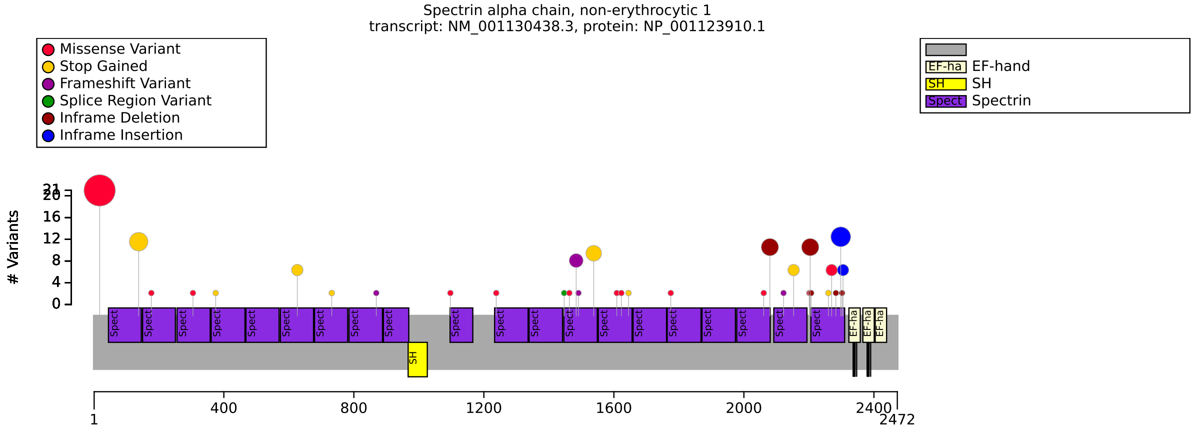

(a) Distribution of variants in *SPTAN1*

HPO term	missense	other	p-value	adj. p-value
Spastic paraplegia [HP:0001258]	21/25 (84%)	0/16 (0%)	4.70×10^{-8}	6.58×10^{-7}
Lower limb spasticity [HP:0002061]	21/22 (95%)	0/12 (0%)	2.37×10^{-8}	6.58×10^{-7}
Appendicular spasticity [HP:0034353]	21/22 (95%)	2/14 (14%)	8.72×10^{-7}	8.14×10^{-6}
Spasticity [HP:0001257]	22/23 (96%)	4/16 (25%)	5.22×10^{-6}	2.92×10^{-5}
Motor axonal neuropathy [HP:0007002]	0/19 (0%)	12/16 (75%)	2.18×10^{-6}	1.53×10^{-5}
Peripheral axonal neuropathy [HP:0003477]	4/23 (17%)	12/16 (75%)	6.69×10^{-4}	0.002
Motor seizure [HP:0020219]	7/27 (26%)	13/17 (76%)	0.002	0.004
Seizure [HP:0001250]	13/33 (39%)	24/28 (86%)	2.48×10^{-4}	9.92×10^{-4}
Intellectual disability [HP:0001249]	9/32 (28%)	18/23 (78%)	3.43×10^{-4}	0.001
Lower limb muscle weakness [HP:0007340]	15/16 (94%)	16/28 (57%)	0.015	0.030
Distal lower limb muscle weakness [HP:0009053]	9/19 (47%)	0/16 (0%)	0.001	0.004
Infantile spasms [HP:0012469]	2/29 (7%)	13/31 (42%)	0.002	0.005
Epileptic spasm [HP:0011097]	2/22 (9%)	13/17 (76%)	2.93×10^{-5}	1.37×10^{-4}
Microcephaly [HP:0000252]	4/34 (12%)	15/32 (47%)	0.002	0.005

(b) Fisher Exact Test performed to compare HPO annotation frequency with respect to missense and other. Total of 28 tests were performed.

HPO term	truncating	other	p-value	adj. p-value
Spastic paraplegia [HP:0001258]	0/8 (0%)	21/33 (64%)	0.001	0.005
Lower limb spasticity [HP:0002061]	0/8 (0%)	21/26 (81%)	7.09×10^{-5}	3.54×10^{-4}
Appendicular spasticity [HP:0034353]	0/8 (0%)	23/28 (82%)	4.25×10^{-5}	2.66×10^{-4}
Spasticity [HP:0001257]	0/8 (0%)	26/31 (84%)	2.09×10^{-5}	1.74×10^{-4}
Hypotonia [HP:0001252]	1/11 (9%)	15/24 (62%)	0.004	0.015
Motor axonal neuropathy [HP:0007002]	12/12 (100%)	0/23 (0%)	1.20×10^{-9}	3.00×10^{-8}
Peripheral axonal neuropathy [HP:0003477]	12/12 (100%)	4/27 (15%)	4.65×10^{-7}	5.82×10^{-6}

(c) Fisher Exact Test performed to compare HPO annotation frequency with respect to truncating and other. Total of 25 tests were performed.

Figure S70: See next page for caption

SPTAN1 (Part 2/2)

HPO term	Arg19Trp	Other	p-value	adj. p-value
Spastic paraplegia [HP:0001258]	21/21 (100%)	0/20 (0%)	3.72×10^{-12}	9.29×10^{-11}
Lower limb spasticity [HP:0002061]	21/21 (100%)	0/13 (0%)	1.08×10^{-9}	1.35×10^{-8}
Appendicular spasticity [HP:0034353]	21/21 (100%)	2/15 (13%)	4.54×10^{-8}	1.89×10^{-7}
Spasticity [HP:0001257]	21/21 (100%)	5/18 (28%)	1.05×10^{-6}	3.30×10^{-6}
Motor axonal neuropathy [HP:0007002]	0/16 (0%)	12/19 (63%)	6.26×10^{-5}	1.74×10^{-4}
Peripheral axonal neuropathy [HP:0003477]	4/20 (20%)	12/19 (63%)	0.010	0.017
Motor seizure [HP:0020219]	0/19 (0%)	20/25 (80%)	3.34×10^{-8}	1.67×10^{-7}
Seizure [HP:0001250]	2/21 (10%)	35/40 (88%)	2.36×10^{-9}	1.47×10^{-8}
Intellectual disability [HP:0001249]	0/21 (0%)	27/34 (79%)	1.76×10^{-9}	1.47×10^{-8}
Lower limb muscle weakness [HP:0007340]	14/14 (100%)	17/30 (57%)	0.003	0.007
Distal lower limb muscle weakness [HP:0009053]	8/15 (53%)	1/20 (5%)	0.002	0.004
Infantile spasms [HP:0012469]	0/19 (0%)	15/41 (37%)	0.001	0.003
Epileptic spasm [HP:0011097]	0/19 (0%)	15/20 (75%)	7.71×10^{-7}	2.75×10^{-6}
Microcephaly [HP:0000252]	0/21 (0%)	19/45 (42%)	2.45×10^{-4}	6.14×10^{-4}

(a) Fisher Exact Test performed to compare HPO annotation frequency with respect to Arg19Trp and Other. Total of 25 tests were performed.

Genotype (A)	Genotype (B)	total tests performed	significant results
Lys2083del	Other	21	0
FEMALE	MALE	28	0

(b) Fisher Exact Test performed to compare HPO annotation frequency with respect to genotypes.

Figure S71: The cohort comprised 85 individuals (40 females, 45 males). 3 of these individuals were reported to be deceased. A total of 96 HPO terms were used to annotate the cohort. Disease diagnoses: Spastic paraplegia 91, autosomal dominant, with or without cerebellar ataxia (OMIM:620538) (28 individuals), Developmental and epileptic encephalopathy 5 (OMIM:613477) (22 individuals), Developmental delay with or without epilepsy (OMIM:620540) (21 individuals), Neuronopathy, distal hereditary motor, autosomal dominant 11 (OMIM:620528) (14 individuals). Several publications have identified genotype-phenotype correlations in *SPTAN1* that are comparable to those identified here [58, 59, 60]. A total of 36 unique variant alleles were found in *SPTAN1* (transcript: `NM_001130438.3`, protein id: `NP_001123910.1`).

STXBP1

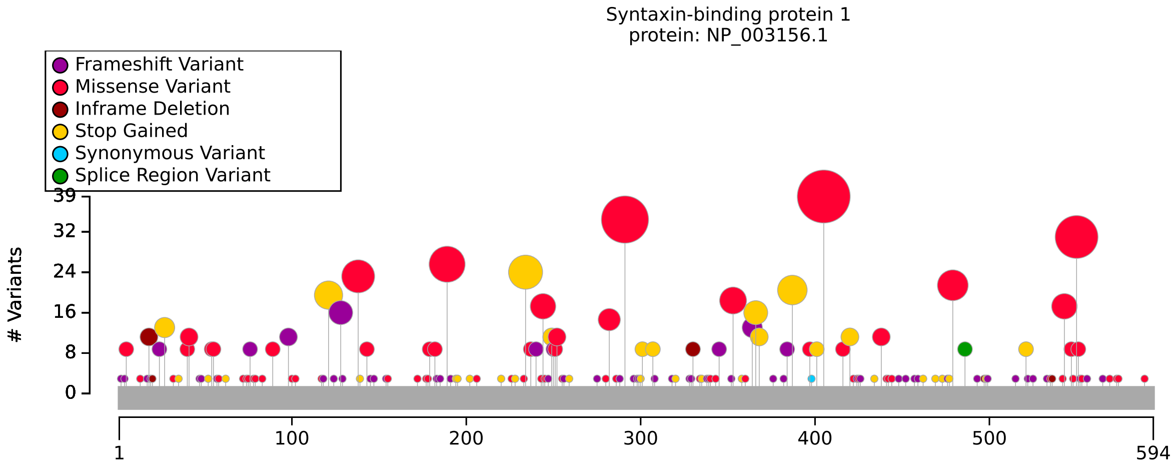

(a) Distribution of variants in *STXBP1*

Genotype (A)	Genotype (B)	total tests performed	significant results
Missense	Other	18	0
Arg406 Variants	Other	18	0
Exon 14	Other	18	0

(b) Fisher Exact Test performed to compare HPO annotation frequency with respect to genotypes.

Figure S72: The cohort comprised 462 individuals (206 females, 220 males, 36 with unknown sex). A total of 516 HPO terms were used to annotate the cohort. Disease diagnosis: Developmental and epileptic encephalopathy 4 (OMIM:612164). No significant genotype-phenotype correlations identified. A total of 259 unique variant alleles were found in *STXBP1* (transcript: `NM_003165.6`, protein id: `NP_003156.1`).

SUOX

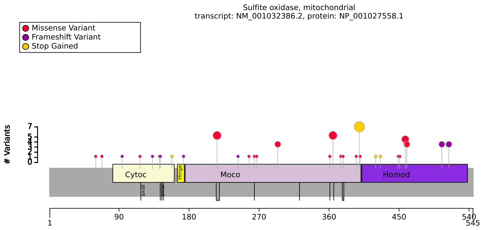

(a) Distribution of variants in *SUOX*

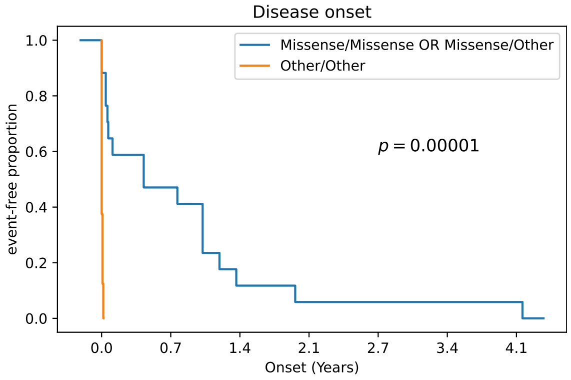

(b) Survival analysis: *SUOX* missense variants and disease onset

HPO term	homodimerization/homodimerization OR homodimerization/Other	Other/Other	p-value	adj. p-value
Microcephaly [HP:0000252]	0/9 (0%)	10/12 (83%)	2.21×10^{-4}	0.003

(c) Fisher Exact Test performed to compare HPO annotation frequency with respect to homodimerization/homodimerization OR homodimerization/Other and Other/Other. Total of 15 tests were performed.

Genotype (A)	Genotype (B)	total tests performed	significant results
Missense/Missense OR Missense/Other	Other/Other	15	0
Moco domain/Moco domain OR Moco domain/Other	Other/Other	15	0

(d) Fisher Exact Test performed to compare HPO annotation frequency with respect to genotypes.

Description	Variable	Genotype (A)	Genotype (B)	p-value	xrefs
Onset of Sulfite oxidase deficiency (OMIM:272300)	Onset of OMIM:272300	Missense/Missense OR Missense/Other	Other/Other	9.18×10^{-6}	-

(e) Onset of OMIM:272300 to compare Missense/Missense OR Missense/Other and Other/Other with respect to Onset of OMIM:272300.

Description	Variable	Genotype (A)	Genotype (B)	p-value	xrefs
Onset of Sulfite oxidase deficiency (OMIM:272300)	Onset of OMIM:272300	homodimerization/homodimerization OR homodimerization/Other	Other/Other	0.853	[61]

(f) Onset of OMIM:272300 to compare homodimerization/homodimerization OR homodimerization/Other and Other/Other with respect to Onset of OMIM:272300.

Figure S73: The cohort comprised 35 individuals (11 females, 14 males, 10 with unknown sex). 8 of these individuals were reported to be deceased. A total of 20 HPO terms were used to annotate the cohort. Disease diagnosis: Sulfite oxidase deficiency (OMIM:272300). A published Genotype-phenotype analysis demonstrated patients with biallelic missense mutations had milder symptoms (P = 0.023), later age of onset (P < 0.001), and a higher incidence of regression (P = 0.017) than other genotypes [61]. A total of 32 unique variant alleles were found in *SUOX* (transcript: `NM_001032386.2`, protein id: `NP_001027558.1`).

TBCK

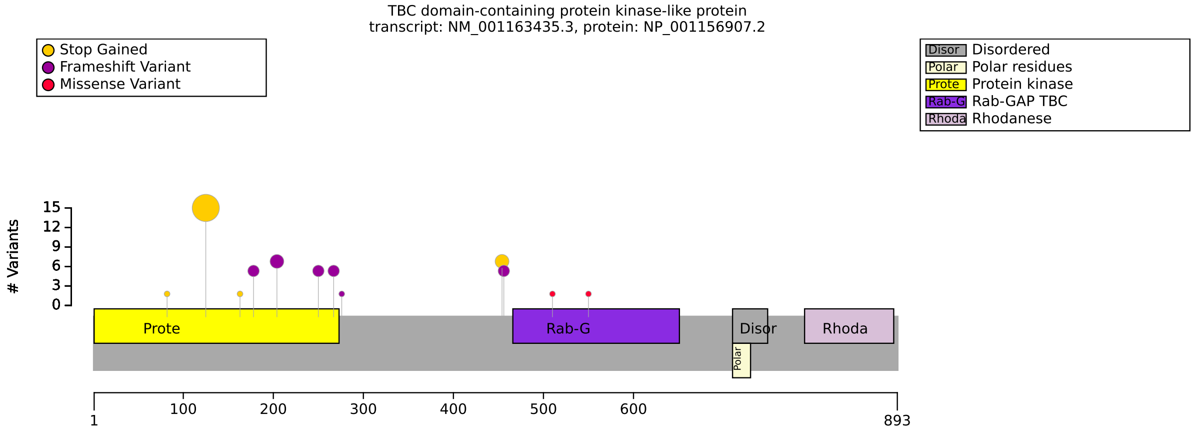

(a) Distribution of variants in *TBCK*

HPO term	R126*/R126*	R126*/other OR other/other	p-value	adj. p-value
Macroglossia [HP:0000158]	11/12 (92%)	3/22 (14%)	1.34×10^{-5}	4.30×10^{-4}
Developmental regression [HP:0002376]	9/12 (75%)	2/22 (9%)	1.83×10^{-4}	0.003

(b) Fisher Exact Test performed to compare HPO annotation frequency with respect to R126*/R126* and R126*/other OR other/other. Total of 32 tests were performed.

Genotype (A)	Genotype (B)	total tests performed	significant results
missense/missense OR missense/other	other/other	30	0
FEMALE	MALE	32	0

(c) Fisher Exact Test performed to compare HPO annotation frequency with respect to genotypes.

Figure S74: The cohort comprised 41 individuals (17 females, 24 males). 3 of these individuals were reported to be deceased. A total of 97 HPO terms were used to annotate the cohort. Disease diagnosis: Hypotonia, infantile, with psychomotor retardation and characteristic facies 3 (OMIM:616900). Durham et al (2023) stated that several studies have touched on a genotype-phenotype correlation of TBCK syndrome; however, more data are required for statistically significant conclusions [62]. A total of 18 unique variant alleles were found in *TBCK* (transcript: `NM_001163435.3`, protein id: `NP_001156907.2`).

TBX1

HPO term	Tyr418PhefsTer42	Other	p-value	adj. p-value
Global developmental delay [HP:0001263]	5/5 (100%)	3/20 (15%)	0.001	0.023
Narrow nose [HP:0000460]	5/5 (100%)	0/6 (0%)	0.002	0.024

(a) Fisher Exact Test performed to compare HPO annotation frequency with respect to Tyr418PhefsTer42 and Other. Total of 22 tests were performed.

Genotype (A)	Genotype (B)	total tests performed	significant results
Missense	Other	21	0

(b) Fisher Exact Test performed to compare HPO annotation frequency with respect to genotypes.

Figure S75: The cohort comprised 26 individuals (10 females, 15 males, 1 with unknown sex). A total of 25 HPO terms were used to annotate the cohort. Disease diagnosis: DiGeorge syndrome (OMIM:188400). A total of 12 unique variant alleles were found in *TBX1* (transcript: `NM_001379200.1`, protein id: `NP_001366129.1`).

TBX5

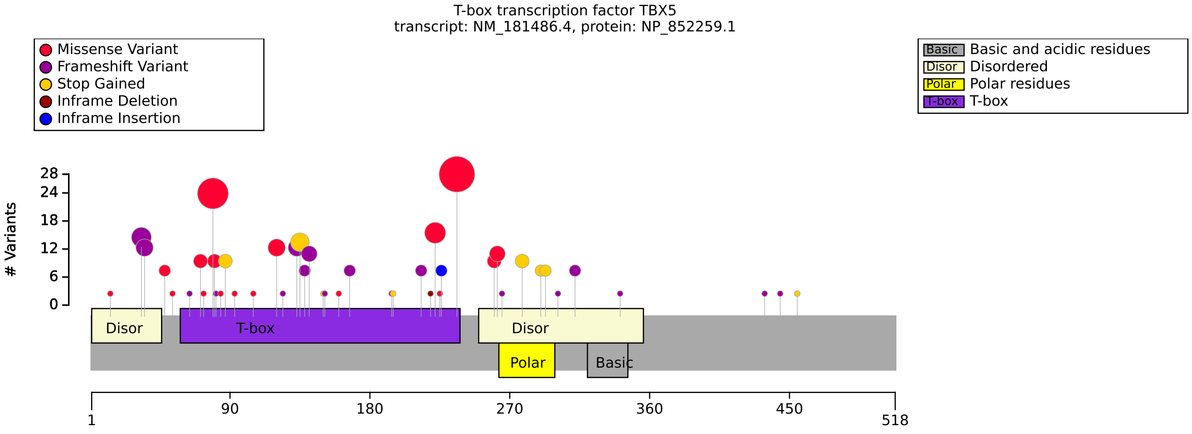

(a) Distribution of variants in *TBX5*

HPO term	missense	other	p-value	adj. p-value
Ventricular septal defect [HP:0001629]	31/60 (52%)	30/30 (100%)	4.63×10^{-7}	1.48×10^{-5}

(b) Fisher Exact Test performed to compare HPO annotation frequency with respect to missense and other. Total of 32 tests were performed.

HPO term	Arg237Gln	other	p-value	adj. p-value
Ventricular septal defect [HP:0001629]	0/17 (0%)	61/73 (84%)	5.55×10^{-11}	1.78×10^{-9}
Upper limb phocomelia [HP:0009813]	7/22 (32%)	3/131 (2%)	4.55×10^{-5}	7.28×10^{-4}

(c) Fisher Exact Test performed to compare HPO annotation frequency with respect to Arg237Gln and other. Total of 32 tests were performed.

Genotype (A)	Genotype (B)	total tests performed	significant results
Gly80Arg	other	28	0
FEMALE	MALE	13	0

(d) Fisher Exact Test performed to compare HPO annotation frequency with respect to genotypes.

Figure S76: The cohort comprised 156 individuals (56 females, 46 males, 54 with unknown sex). A total of 90 HPO terms were used to annotate the cohort. Disease diagnosis: Holt-Oram syndrome (OMIM:142900). Vanlerberghe et al. (2019) observed that isolated septal CHD are more common in the truncating than in the missense variants (p=0.02) [63]. A total of 53 unique variant alleles were found in *TBX5* (transcript: `NM_181486.4`, protein id: `NP_852259.1`).

TGFB2

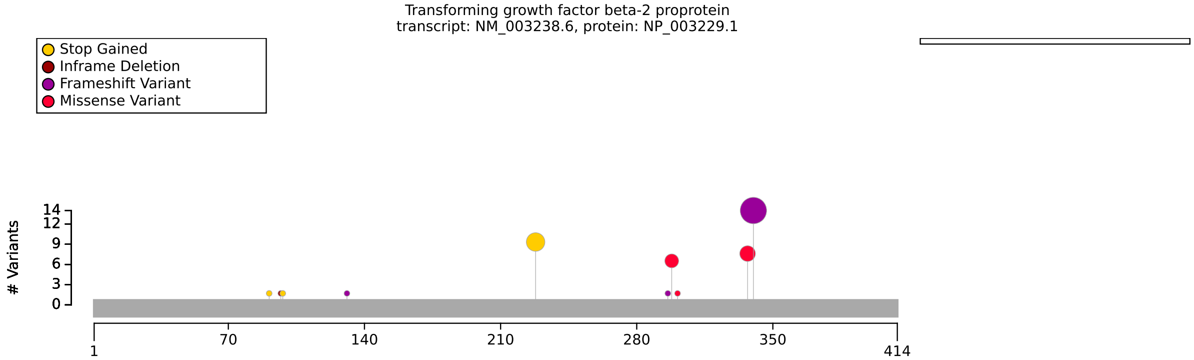

(a) Distribution of variants in *TGFB2*

Genotype (A)	Genotype (B)	total tests performed	significant results
missense	other	37	0
FEMALE	MALE	38	0

(b) Fisher Exact Test performed to compare HPO annotation frequency with respect to genotypes.

Figure S77: The cohort comprised 36 individuals (10 females, 26 males). 1 of these individuals were reported to be deceased. A total of 68 HPO terms were used to annotate the cohort. Disease diagnosis: Loeys-Dietz syndrome 4 (OMIM:614816). No significant association found. A total of 12 unique variant alleles were found in *TGFB2* (transcript: `NM_003238.6`, protein id: `NP_003229.1`).

TGFB3

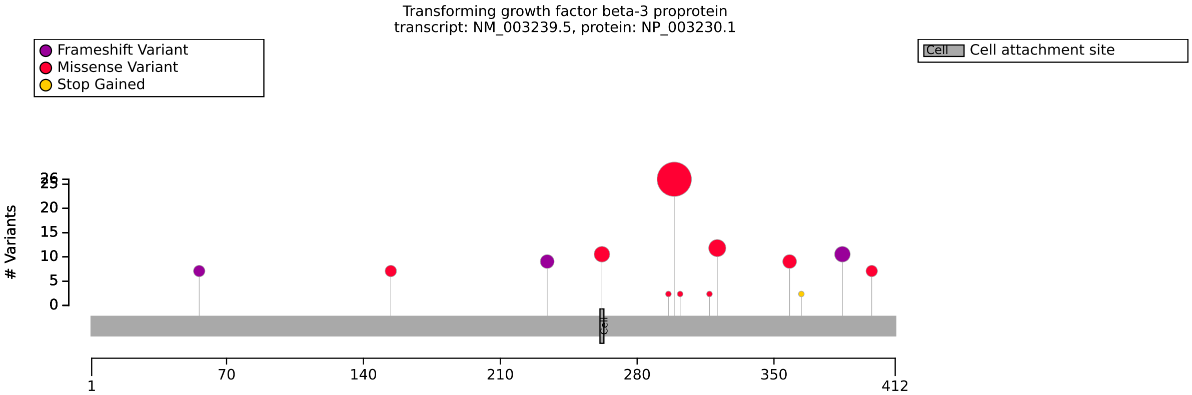

(a) Distribution of variants in *TGFB3*

Genotype (A)	Genotype (B)	total tests performed	significant results
Missense	Other	57	0
Asp263His	Other	18	0
FEMALE	MALE	57	0

(b) Fisher Exact Test performed to compare HPO annotation frequency with respect to genotypes.

Figure S78: The cohort comprised 75 individuals (34 females, 41 males). 7 of these individuals were reported to be deceased. A total of 73 HPO terms were used to annotate the cohort. Disease diagnosis: Loeys-Dietz syndrome 5 (OMIM:615582). No significant correlations identified. A total of 18 unique variant alleles were found in *TGFB3* (transcript: `NM_003239.5`, protein id: `NP_003230.1`).

TGFBR1

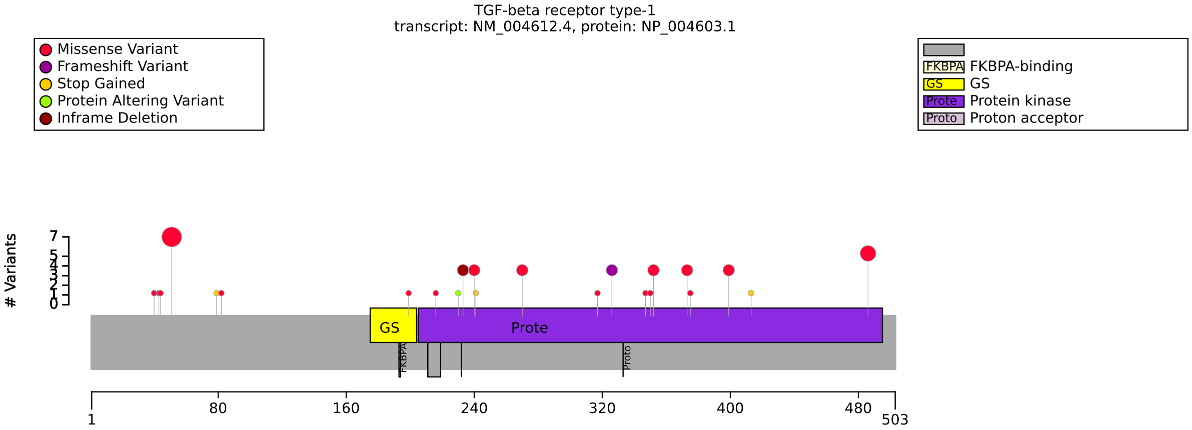

(a) Distribution of variants in *TGFBR1*

HPO term	MSSE var	other	p-value	adj. p-value
Self-healing squamous epithelioma [HP:0034720]	18/19 (95%)	0/21 (0%)	1.68×10^{-10}	2.68×10^{-9}
Hypertelorism [HP:0000316]	0/18 (0%)	15/19 (79%)	5.01×10^{-7}	4.01×10^{-6}
Arterial tortuosity [HP:0005116]	1/19 (5%)	8/16 (50%)	0.005	0.026

(b) Fisher Exact Test performed to compare HPO annotation frequency with respect to MSSE var and other. Total of 16 tests were performed.

HPO term	Gly52Arg	Other	p-value	adj. p-value
Self-healing squamous epithelioma [HP:0034720]	7/7 (100%)	11/33 (33%)	0.002	0.012

(c) Fisher Exact Test performed to compare HPO annotation frequency with respect to Gly52Arg and Other. Total of 7 tests were performed.

Genotype (A)	Genotype (B)	total tests performed	significant results
FEMALE	MALE	21	0

(d) Fisher Exact Test performed to compare HPO annotation frequency with respect to genotypes.

Figure S79: The cohort comprised 41 individuals (7 females, 16 males, 18 with unknown sex). 2 of these individuals were reported to be deceased. A total of 78 HPO terms were used to annotate the cohort. Disease diagnoses: Loeys-Dietz syndrome 1 (OMIM:609192) (23 individuals), Multiple self-healing squamous epithelioma, susceptibility to (OMIM:132800) (18 individuals). A total of 27 unique variant alleles were found in *TGFBR1* (transcript: `NM_004612.4`, protein id: `NP_004603.1`). Goudie et al. (2011) reported that the nature of the sequence variants, which include mutations in the extracellular ligand-binding domain and a series of truncating mutations in the kinase domain, indicates a clear genotype-phenotype correlation between loss-of-function *TGFBR1* mutations and MSSE [64].

TGFBR2

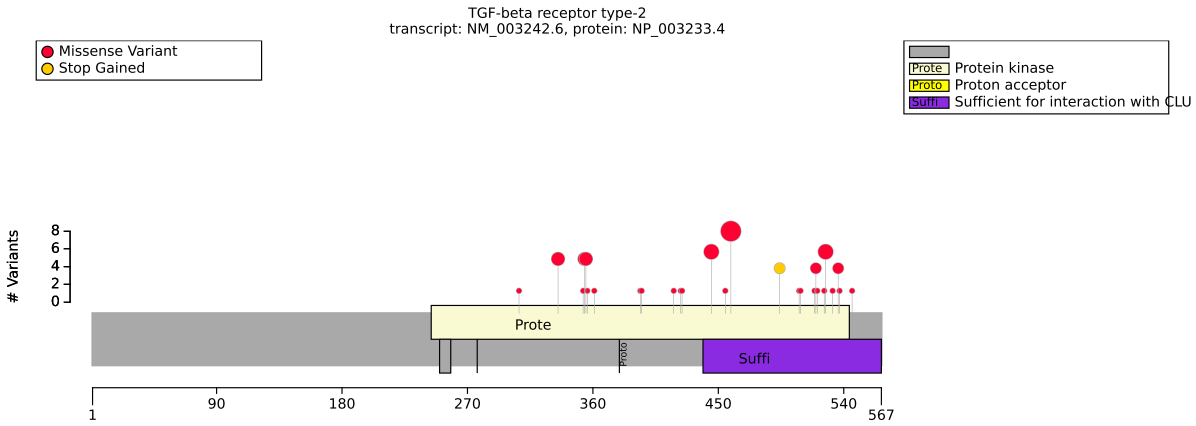

(a) Distribution of variants in *TGFBR2*

Genotype (A)	Genotype (B)	total tests performed	significant results
CLU interaction region	Other	42	0
Missense	Other	42	0
FEMALE	MALE	42	0

(b) Fisher Exact Test performed to compare HPO annotation frequency with respect to genotypes.

Figure S80: The cohort comprised 53 individuals (23 females, 30 males). 4 of these individuals were reported to be deceased. A total of 96 HPO terms were used to annotate the cohort. Disease diagnosis: Loeys-Dietz syndrome 2 (OMIM:610168). No statistically significant results identified. A total of 34 unique variant alleles were found in *TGFBR2* (transcript: `NM_003242.6`, protein id: `NP_003233.4`).

TRAF7

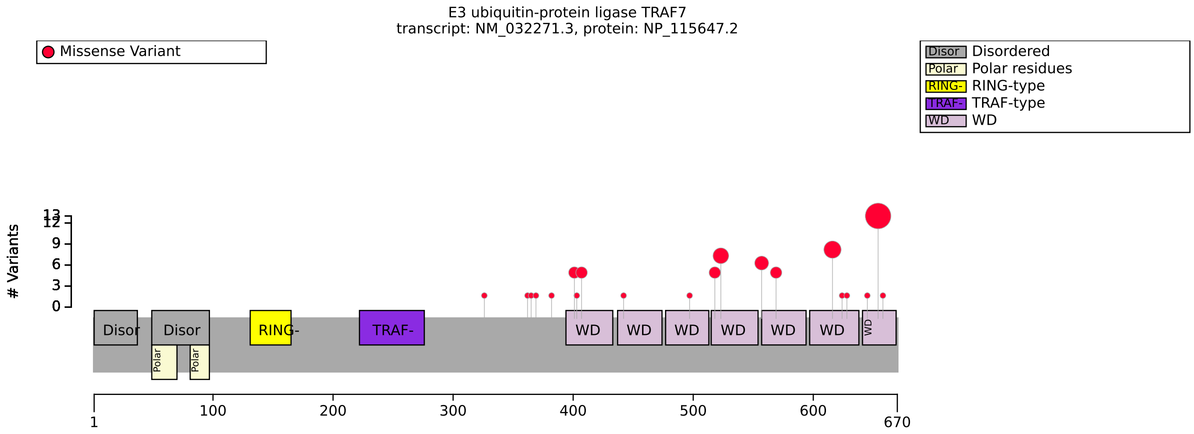

(a) Distribution of variants in *TRAF7*

Genotype (A)	Genotype (B)	total tests performed	significant results
WD7	other	40	0
Arg655Gln	Other variant	40	0
FEMALE	MALE	40	0

(b) Fisher Exact Test performed to compare HPO annotation frequency with respect to genotypes.

Figure S81: The cohort comprised 45 individuals (17 females, 28 males). A total of 360 HPO terms were used to annotate the cohort. Disease diagnosis: Cardiac, facial, and digital anomalies with developmental delay (OMIM:618164). No significant correlations identified. A total of 23 unique variant alleles were found in *TRAF7* (transcript: `NM_032271.3`, protein id: `NP_115647.2`).

U2AF2

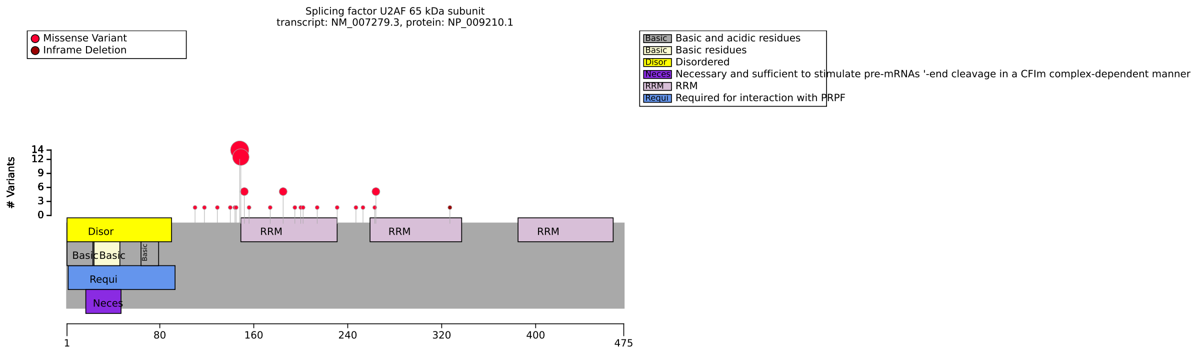

(a) Distribution of variants in *U2AF2*

Genotype (A)	Genotype (B)	total tests performed	significant results
r149W	other	177	0
R149,R150 variants	other	177	0
RRM 1	other	177	0
FEMALE	MALE	177	0

(b) Fisher Exact Test performed to compare HPO annotation frequency with respect to genotypes.

Figure S82: The cohort comprised 48 individuals (28 females, 20 males). 2 of these individuals were reported to be deceased. A total of 178 HPO terms were used to annotate the cohort. Disease diagnosis: Developmental delay, dysmorphic facies, and brain anomalies (OMIM:620535). No statistical analysis of GPCs in *U2AF2* identified in published literature. A total of 24 unique variant alleles were found in *U2AF2* (transcript: `NM_007279.3`, protein id: `NP_009210.1`).

UMOD

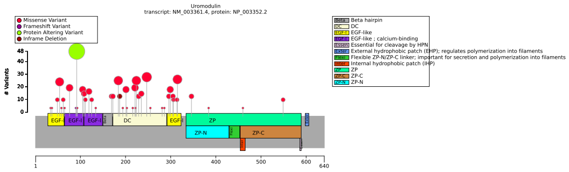

(a) Distribution of variants in *UMOD*

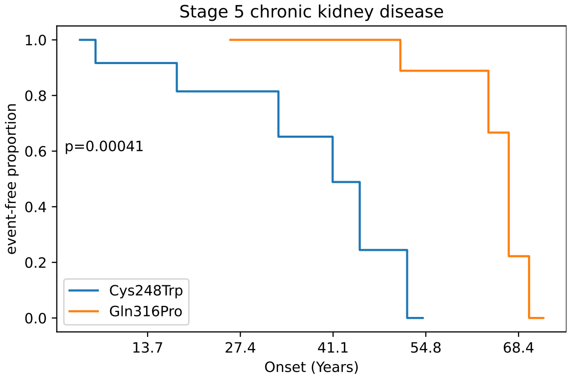

(b) Onset of Stage 5 chronic kidney disease (HP:0003774)

HPO term	EGF	other	p-value	adj. p-value
Hyperuricemia [HP:0002149]	14/32 (44%)	50/57 (88%)	1.77×10^{-5}	1.06×10^{-4}

(c) Fisher Exact Test: HPO annotation frequency and EGF vs. other. Total of 6 tests were performed.

HPO term	cysteine	other	p-value	adj. p-value
Hyperuricemia [HP:0002149]	38/41 (93%)	26/48 (54%)	4.48×10^{-5}	2.69×10^{-4}

(d) Fisher Exact Test: HPO annotation frequency and cysteine vs other. Total of 6 tests were performed.

Genotype (A)	Genotype (B)	total tests performed	significant results
FEMALE	MALE	5	0

(e) Fisher Exact Test performed to compare HPO annotation frequency with respect to genotypes.

Description	Variable	Genotype (A)	Genotype (B)	p-value	xrefs
Survival analysis: Stage 5 chronic kidney disease	Onset of HP:0003774	EGF	other	0.284	-

(f) Onset of Stage 5 chronic kidney disease to compare EGF and other with respect to Onset of HP:0003774.

Description	Variable	Genotype (A)	Genotype (B)	p-value	xrefs
Survival analysis: Stage 5 chronic kidney disease	Onset of HP:0003774	278_289delins	other	0.835	-

(g) Onset of Stage 5 chronic kidney disease to compare 278_289delins and other with respect to Onset of HP:0003774.

Description	Variable	Genotype (A)	Genotype (B)	p-value	xrefs
Survival analysis: Stage 5 chronic kidney disease	Onset of HP:0003774	Cys248Trp	Gln316Pro	4.10×10^{-4}	-

(h) Onset of Stage 5 chronic kidney disease to compare Cys248Trp and Gln316Pro with respect to Onset of Stage 5 chronic kidney disease (HP:0003774).

Figure S83: The cohort comprised 207 individuals (80 females, 88 males, 39 with unknown sex). 30 HPO terms were used to annotate the cohort. Disease diagnoses: Tubulointerstitial kidney disease, autosomal dominant, 1 (OMIM:162000) (183 individuals), Tubulointerstitial kidney disease, autosomal dominant, 3 (OMIM:162002) (12 individuals), Tubulointerstitial kidney disease, autosomal dominant, 2 (OMIM:162001) (12 individuals). One study on *UMOD* variants showed that median ages at ESRD development were lowest with Cys77Tyr and highest with Gln316Pro [65], which is compatible with our finding here. Another showed that indel mutation p.Val93_Gly97delinsAlaAlaSerCys is associated with a relatively mild clinical UAKD phenotype [66]. We did not observe a significant association between this variant and age of onset with our dataset. A total of 53 unique variant alleles were found in *UMOD* (transcript: `NM_003361.4`, protein id: `NP_003352.2`).

WWOX

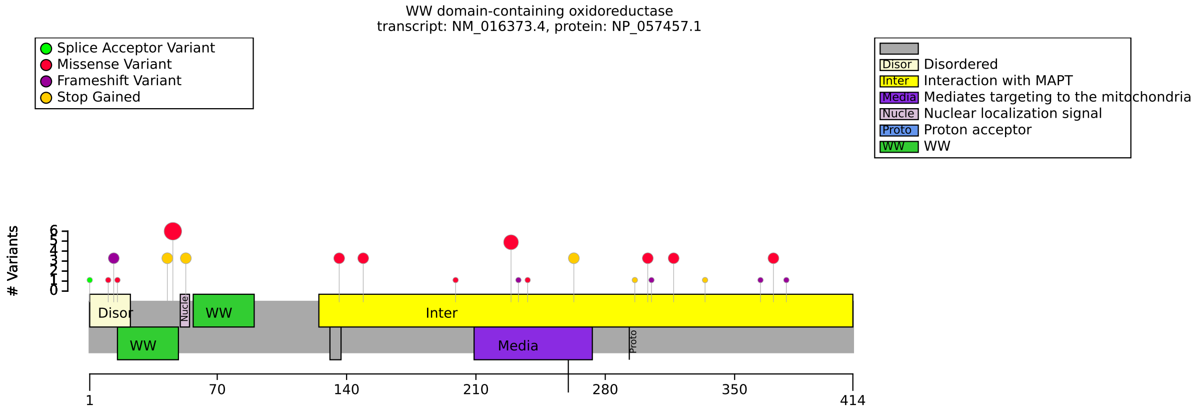

(a) Distribution of variants in *WWOX*

HPO term	MAPT Interaction/MAPT Interaction OR MAPT Interaction/Other	Other/Other	p-value	adj. p-value
Bilateral tonic-clonic seizure with focal onset [HP:0007334]	0/18 (0%)	7/9 (78%)	4.05×10^{-5}	0.002

(b) Fisher Exact Test performed to compare HPO annotation frequency with respect to MAPT Interaction/MAPT Interaction OR MAPT Interaction/Other and Other/Other. Total of 44 tests were performed.

Genotype (A)	Genotype (B)	total tests performed	significant results
Missense/Missense OR Missense/Other	Other/Other	44	0
FEMALE	MALE	44	0

(c) Fisher Exact Test performed to compare HPO annotation frequency with respect to genotypes.

Figure S84: The cohort comprised 38 individuals (25 females, 13 males). 11 of these individuals were reported to be deceased. A total of 72 HPO terms were used to annotate the cohort. Disease diagnoses: Developmental and epileptic encephalopathy 28 (OMIM:616211) (32 individuals), Spinocerebellar ataxia, autosomal recessive 12 (OMIM:614322) (6 individuals). Phenotype/genotype correlations were recently suggested for *WWOX*-related neurodevelopmental disorders with a classification of *WWOX* genotypes into three groups. According to the tentative classification, patients carrying two predicted null alleles were more likely to present with the most severe WOREE phenotype whereas hypomorphic genotypes with two missense variants would instead result in spinocerebellar ataxia (SCAR12) [67, 68, 69]. A total of 33 unique variant alleles were found in *WWOX* (transcript: `NM_016373.4`, protein id: `NP_057457.1`).

ZFX

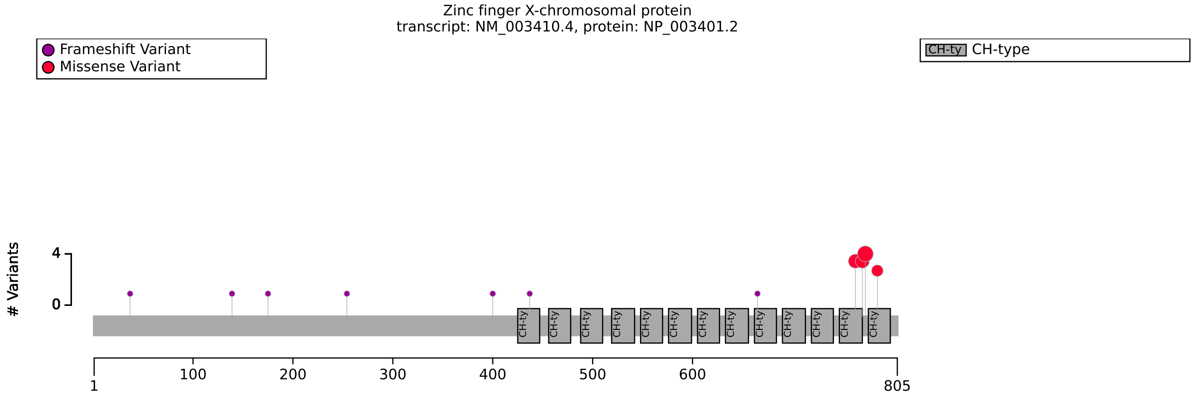

(a) Distribution of variants in *ZFX*

HPO term	missense	other	p-value	adj. p-value
Hyperparathyroidism [HP:0000843]	7/9 (78%)	0/5 (0%)	0.021	0.021

(b) Fisher Exact Test performed to compare HPO annotation frequency with respect to missense and other. Total of 1 test was performed.

Genotype (A)	Genotype (B)	total tests performed	significant results
missense	other	225	0

(c) Fisher Exact Test performed to compare HPO annotation frequency with respect to genotypes.

Figure S85: The cohort comprised 19 individuals (4 females, 14 males, 1 with unknown sex). A total of 203 HPO terms were used to annotate the cohort. Disease diagnosis: Intellectual developmental disorder, X-linked syndromic 37 (OMIM:301118). The small cohort size and the fact that the majority of the *ZFX* variants were private to each proband or family made the assessment of genotype-phenotype correlation difficult. A recent report of a germline *ZFX* missense variant in a patient With primary hyperparathyroidism suggested the hypothesis of testing for correlation between missense variants and hyperparathyroidism [70, 71]. A total of 11 unique variant alleles were found in *ZFX* (transcript: `NM_003410.4`, protein id: `NP_003401.2`).

ZMYM3

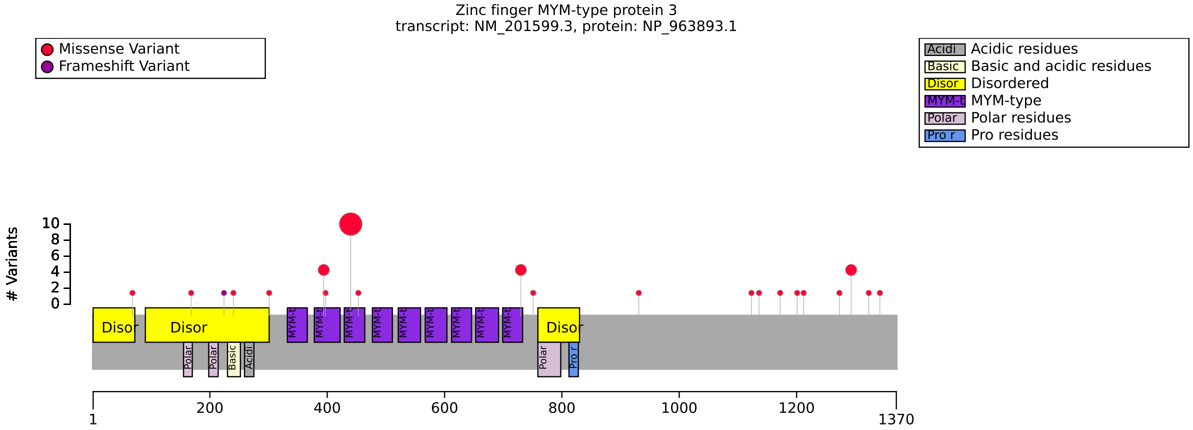

(**a**) Distribution of variants in *ZMYM3*

HPO term	R441	other	p-value	adj. p-value
Cupped ear [HP:0000378]	7/10 (70%)	1/23 (4%)	2.02×10^{-4}	0.012

(**b**) Fisher Exact Test performed to compare HPO annotation frequency with respect to R441 and other. Total of 60 tests were performed.

Genotype (A)	Genotype (B)	total tests performed	significant results
N term	other	60	0

(**c**) Fisher Exact Test performed to compare HPO annotation frequency with respect to genotypes.

Figure S86: The cohort comprised 33 individuals (3 females, 30 males). 1 of these individuals were reported to be deceased. A total of 78 HPO terms were used to annotate the cohort. Disease diagnosis: Intellectual developmental disorder, X-linked 112 (OMIM:301111). A total of 22 unique variant alleles were found in *ZMYM3* (transcript: `NM_201599.3`, protein id: `NP_963893.1`). No analysis of *ZMYM3* genotype phgenotype correlation was presented in the two studies on *ZMYM3* variants study to be published to date [72, 73].

ZNF462

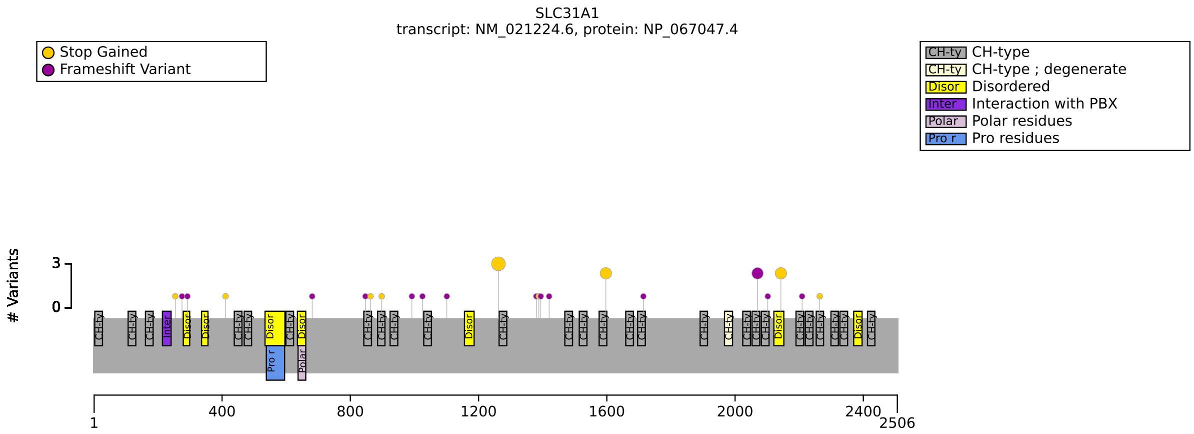

(a) Distribution of variants in *ZNF462*

Genotype (A)	Genotype (B)	total tests performed	significant results
Ablation	Other	43	0
FEMALE	MALE	41	0

(b) Fisher Exact Test performed to compare HPO annotation frequency with respect to genotypes.

Figure S87: The cohort comprised 39 individuals (11 females, 25 males, 3 with unknown sex). A total of 32 HPO terms were used to annotate the cohort. Disease diagnosis: Weiss-Kruszka syndrome (OMIM:618619). No statistically significant genotype phenotype association was identified. A total of 26 unique variant alleles were found in *ZNF462* (transcript: `NM_021224.6`, protein id: `NP_067047.4`).

ZSWIM6

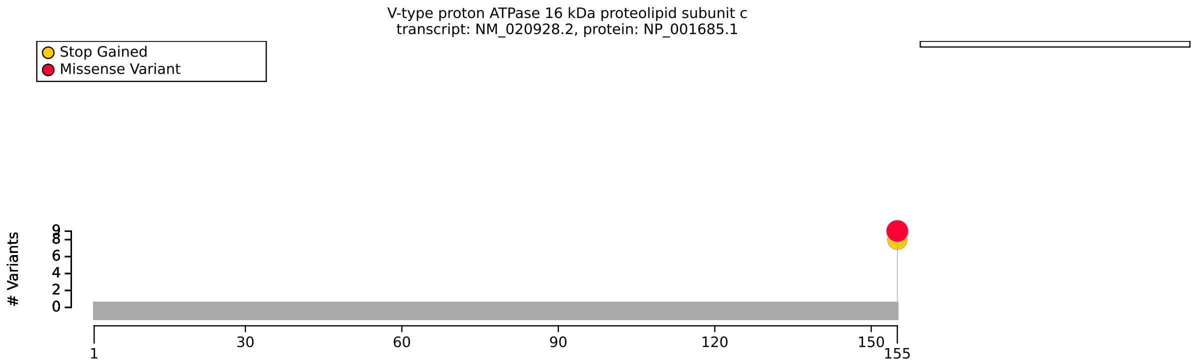

(a) Distribution of variants in *ZSWIM6*

Genotype (A)	Genotype (B)	total tests performed	significant results
p.Arg913Ter	Arg1163Trp	12	0

(b) Fisher Exact Test performed to compare HPO annotation frequency with respect to genotypes.

Figure S88: The cohort comprised 16 individuals (8 females, 8 males). A total of 85 HPO terms were used to annotate the cohort. Disease diagnoses: Acromelic frontonasal dysostosis (OMIM:603671) (9 individuals), Neurodevelopmental disorder with movement abnormalities, abnormal gait, and autistic features (OMIM:617865) (7 individuals). No statistically significant results identified. A total of 2 unique variant alleles were found in *ZSWIM6* (transcript: `NM_020928.2`, protein id: `NP_001685.1`).

Name	Match criterion	Example
variant key	Specific variant	1_8364773_8364773_A_AC (NM_001042681.2:c.1512dup)
variant effect	Leads to the target effect	START_LOST
variant class	Variant has the target type	SNV, DEL, DUP, INS, INV
gene	Variant affects gene	Affects *RERE*
transcript	Variant affects transcript	Overlaps NM_12345.01
exon	Variant overlaps exon	Located in exon 3
region	Variant overlaps region on protein	Located between amino acid residues 1 to 77
is large imprecise sv	Variant is structural variant without exact breakpoint coordinates	–
is structural variant	The variant affects at least n base pairs (n=50 by default) or is a large imprecise SV or a translocation	–
structural type	The target ontology class for a variant with imprecise/unknown breakpoints	Chromosomal deletion (SO:1000029)
is structural deletion	Variant is a Chromosomal deletion (SO:1000029) or an SV with known breakpoints that deletes at least a n base pairs (`n=50bp` by default)	–
ref length	Length of the reference sequence is above, below, or (not) equal to n bases	$n > 50$ for variants affecting more than 50 bp
change length	Change of length between the REF/ALT is above, below, or (not) equal to `n` bases	$n \leq -50$ for testing if a variant removes at least 50 base pairs
protein feature type	Variant affects protein feature type	DOMAIN
protein feature	Variant region overlaps a protein feature	Overlaps with ”Bipartite nuclear localization signal”
allof	Logical AND for two or more predicates to test if *all* predicates match	missense AND exon 7
anyof	Logical OR for two or more predicates to test if *any* predicate matches	START_LOST OR STOP_GAINED
not	Logical NOT	NOT(missense)

Table S1: Variant predicates. Version 0.9.11 of GPSEA offers the variant predicates shown in this table. Each predicate evaluates a variant and returns `True` or `False`.

cohort	genotype (A)	genotype (B)	Scorer	p-val	xrefs
ANKRD11	FEMALE	MALE	HPO Group Count	0.007	-
ANKRD11	SV	C	HPO Group Count	2.64×10^{-4}	[3]
CHD8	FEMALE	MALE	De Vries Score	0.006	-
CHD8	missense	C	De Vries Score	9.0×10^{-4}	-
CTCF	missense	C	De Vries Score	0.009	-
LMNA	Upstream of NLS	C	HPO Group Count	1.83×10^{-16}	-
RERE	LoF	Atrophin	HPO Group Count	1.0×10^{-3}	-

Table S3: Phenotype severity scores. Mann-Whitney U tests performed using GPSEA to assess the association between a genotype and the total value of a phenotype severity score. The references in the xrefs column show previous publications that have presented similar findings. HPO Group Count scorer assigns a phenotype score that is equivalent to the count of present phenotypes that are either an exact match to the query terms or their descendants. DeVries scorer is an adaption of the DeVries score [57] using HPO. C: set complement of a variant predicate.

cohort	genotype (A)	genotype (B)	Outcome Variable	p-val	xrefs
ACADM	K329Q: 1/1	$1/\complement$ OR $\complement/\complement$	MCAD Activity% [LOINC:74892-1]	6.1×10^{-10}	[1]
ACADM	Y67H: 1/1 OR $1/\complement$	$\complement/\complement$	MCAD Activity% [LOINC:74892-1]	2.0×10^{-5}	[1]
CYP21A2	missense: $1/\complement$ OR $\complement/\complement$	1/1	17-Hydroxyprogesterone [LOINC:1668-3]	7.9×10^{-6}	-

Table S4: Student t-tests performed using GPSEA. $\complement$: set complement of a variant predicate. Citations in the xrefs column show previous publications that have presented similar findings.

cohort	genotype (A)	genotype (B)	HPO Term	p-val	xrefs
AIRE	R257*/R257* OR R257*/∁	∁/∁	Survival analysis: Chronic mucocutaneous candidiasis	0.019	-
CLDN16	missense/missense OR missense/∁	∁/∁	Survival analysis: Stage 5 chronic kidney disease	0.034	-
UMOD	Cys248Trp	Gln316Pro	Survival analysis: Stage 5 chronic kidney disease	4.1×10^{-4}	-

Table S5: Age of onset of phenotypic abnormality. Logrank tests performed using GPSEA to assess association between a genotype and the age of onset of a phenotypic feature represented by an HPO term. ∁: set complement of a variant predicate. Citations in the xrefs column show previous publications that have presented similar findings.

cohort	genotype (A)	genotype (B)	Disease onset	p-val	xrefs
CNTNAP2	1 allele	2 alleles	Compute time until OMIM:610042 onset	6.2×10^{-6}	-
FBXL4	missense/$\complement$ OR $\complement$/$\complement$	missense/missense	Compute time until OMIM:615471 onset	0.031	-
HMGCS2	missense/missense OR missense/$\complement$	$\complement$/$\complement$	Compute time until OMIM:605911 onset	0.038	-
MPV17	missense/missense OR missense/$\complement$	$\complement$/$\complement$	Compute time until OMIM:256810 onset	0.002	-
SETD2	Missense	$\complement$	Compute time until OMIM:616831 onset	8.5×10^{-5}	-
SUOX	Missense/Missense OR Missense/$\complement$	$\complement$/$\complement$	Compute time until OMIM:272300 onset	9.2×10^{-6}	-

Table S6: Age of onset of disease. Log rank tests performed using GPSEA to assess association between a genotype and the age of onset of a disease. $\complement$: set complement of a variant predicate. Citations in the xrefs column show previous publications that have presented similar findings. `hdim`: homodimerization; `OMIM:610042`: Pitt-Hopkins like syndrome 1; `OMIM:615471`: Mitochondrial DNA depletion syndrome 13 (encephalomyopathic type); `OMIM:605911`: HMG-CoA synthase-2 deficiency; `OMIM:256810`: Mitochondrial DNA depletion syndrome 6 (hepatocerebral type); `OMIM:616831`: Luscan-Lumish syndrome; `OMIM:272300`: Sulfite oxidase deficiency.

cohort	genotype (A)	genotype (B)	Disease	p-val	xrefs
MPV17	Pro98Leu: 1/1	Pro98Leu: 1/C or C/C	OMIM:256810	0.010	-

Table S7: Age of death. Log rank tests performed using GPSEA to assess association between a genotype and the age of death of individuals with a disease. C: set complement of a variant predicate. Citations in the xrefs column show previous publications that have presented similar findings. OMIM:256810: Mitochondrial DNA depletion syndrome 6 (hepatocerebral type).

Cohort	HPO	disease A		disease B		p-val	adj. p
ATP13A2	Bradykinesia [HP:0002067]	OMIM:606693	30/32 (94%)	OMIM:617225	4/10 (40%)	9.2×10^{-4}	0.013
ATP13A2	Parkinsonism [HP:0001300]	OMIM:606693	28/28 (100%)	OMIM:617225	3/11 (27%)	2.7×10^{-6}	7.8×10^{-5}
Kabuki	Feeding difficulties [HP:0011968]	OMIM:147920	8/25 (32%)	OMIM:300867	55/63 (87%)	7.4×10^{-7}	2.5×10^{-5}
Kabuki	Motor delay [HP:0001270]	OMIM:147920	4/10 (40%)	OMIM:300867	58/61 (95%)	1.0×10^{-4}	0.002
LDS 1 and 3	Aortic aneurysm [HP:0004942]	OMIM:609192	11/11 (100%)	OMIM:613795	26/48 (54%)	0.004	0.027
LDS 1 and 3	Hypertelorism [HP:0000316]	OMIM:609192	15/19 (79%)	OMIM:613795	13/35 (37%)	0.004	0.027
LDS 1 and 3	Osteoarthritis [HP:0002758]	OMIM:609192	0/11 (0%)	OMIM:613795	26/38 (68%)	4.6×10^{-5}	1.0×10^{-3}
LDS 1 and 3	Scoliosis [HP:0002650]	OMIM:609192	18/21 (86%)	OMIM:613795	20/43 (47%)	0.003	0.027
LDS 3 and 6	Thoracic aortic aneurysm [HP:0012727]	OMIM:613795	0/22 (0%)	OMIM:619656	10/16 (62%)	1.7×10^{-5}	4.2×10^{-4}
RPGRIP1	Nystagmus [HP:0000639]	OMIM:613826	64/66 (97%)	OMIM:608194	11/16 (69%)	0.003	0.020
RPGRIP1	Very low visual acuity [HP:0032122]	OMIM:613826	35/39 (90%)	OMIM:608194	4/16 (25%)	5.2×10^{-6}	7.8×10^{-5}
Robinow	Cleft palate [HP:0000175]	OMIM:268310	0/17 (0%)	OMIM:616331	5/8 (62%)	1.0×10^{-3}	0.031
Robinow	Hearing impairment [HP:0000365]	OMIM:268310	3/22 (14%)	OMIM:616331	7/7 (100%)	7.7×10^{-5}	0.003
Robinow	Mesomelia [HP:0003027]	OMIM:268310	31/31 (100%)	OMIM:616331	10/15 (67%)	0.002	0.048
Robinow	Short stature [HP:0004322]	OMIM:268310	29/29 (100%)	OMIM:616331	3/11 (27%)	2.2×10^{-6}	1.9×10^{-4}

Table S8: Fischer exact test for association between disease diagnosis and phenotypic features. `OMIM:606693`: Kufor-Rakeb syndrome (KRS); `OMIM:617225`: Spastic paraplegia 78, autosomal recessive (SPG78); `OMIM:147920`: Kabuki syndrome 1 (KABUK1); `OMIM:300867`: Kabuki syndrome 2 (KABUK2); `OMIM:609192`: Loeys-Dietz syndrome 1 (LDS1); `OMIM:613795`: Loeys-Dietz syndrome 3 (LDS3); `OMIM:619656`: Loeys-Dietz syndrome 6 (LDS6); `OMIM:613826`: Leber congenital amaurosis 6 (LCA6); `OMIM:608194`: Cone-rod dystrophy 13 (CORD13); `OMIM:268310`: Robinow syndrome, autosomal recessive 1 (RRS1); `OMIM:616331`: Robinow syndrome, autosomal dominant 2 (DRS2).

cohort	HPO	genotype (A)	Counts (A)	genotype (B)	Counts (B)	p-val	adj. p
KDM6A	Intellectual disability, severe [HP:0010864]	FEMALE	7/25 (28%)	MALE	14/18 (78%)	0.002	0.008

Table S9: Fischer exact test for association between phenotypic features and sex (male, female). Adj. p: p value adjusted with Benjamini-Hochberg method.

Table S10: Detailed results for Table 1 of the main manuscript. Sig: Number of significant associations. **Pub**: Number of these identified in the literature. **Categorical**: Association of genotypes with phenotypes by Fisher exact test. **t test**: Test of means of continuous values by Student t test. **HPO**: Logrank test for association of genotypes with age of onset of a phenotypic abnormality represented by an HPO term. **Disease**: Logrank test for association of genotypes with age of onset of a disease. **Mortality**: Logrank test for association of genotypes with age of death. **Phenotype score**: Mann Whitney U test for association of genotypes with magnitude of a phenotype severity score. Cohorts with no found significant associations are not reported. **Disease diagnosis**: Comparison of two or more diseases associated with the variants in the same gene. **Sex differences**: Comparison of frequencies of phenotypic features in a disease between males and females.

Cohort	Sig	Pub	Categorical	t test	HPO	Disease onset	Mortality	Phenotype score	Disease diagnosis	Sex differences
ACADM	2	2	-	2/2	-	-	-	-	-	-
AIRE	1	1	-	-	1/1	-	-	-	-	-
ANKRD11	2	1	-	-	-	-	-	1/2	-	-
ATP13A2	2	2	-	-	-	-	-	-	2/2	-
CHD8	2	1	-	-	-	-	-	1/2	-	-
CLDN16	1	1	-	-	1/1	-	-	-	-	-
CNTNAP2	1	0	-	-	-	0/1	-	-	-	-
CTCF	1	0	-	-	-	-	-	0/1	-	-
CYP21A2	1	1	-	1/1	-	-	-	-	-	-
EHMT1	1	1	1/1	-	-	-	-	-	-	-
FBN1	14	14	14/14	-	-	-	-	-	-	-
FBXL4	2	0	0/1	-	-	0/1	-	-	-	-
FGD1	1	1	1/1	-	-	-	-	-	-	-
GLI3	14	14	14/14	-	-	-	-	-	-	-
HMGCS2	1	0	-	-	-	0/1	-	-	-	-
IKZF1	2	2	2/2	-	-	-	-	-	-	-
ITPR1	21	0	0/21	-	-	-	-	-	-	-
Kabuki	2	0	-	-	-	-	-	-	0/2	-
KCNH5	1	1	1/1	-	-	-	-	-	-	-
KDM6A	2	1	1/1	-	-	-	-	-	-	0/1
LDS 1 and 3	4	0	-	-	-	-	-	-	0/4	-
LDS 3 and 6	1	0	-	-	-	-	-	-	0/1	-
LMNA	36	36	35/35	-	-	-	-	1/1	-	-

Continued on next page

Table S10 – continued from previous page

Cohort	Sig	Pub	Categorical	t test	HPO	Disease onset	Mortality	Phenotype score	Disease diagnosis	Sex differences
MPV17	3	1	0/1	-	-	0/1	1/1	-	-	-
NBAS	1	0	0/1	-	-	-	-	-	-	-
NF1	37	37	37/37	-	-	-	-	-	-	-
PTPN11	4	0	0/4	-	-	-	-	-	-	-
RERE	1	1	-	-	-	-	-	1/1	-	-
Robinow	4	0	-	-	-	-	-	-	0/4	-
RPGRIP1	3	1	1/1	-	-	-	-	-	0/2	-
SAMD9L	3	0	0/3	-	-	-	-	-	-	-
SATB2	1	0	0/1	-	-	-	-	-	-	-
SCN2A	9	9	9/9	-	-	-	-	-	-	-
SCO2	1	0	0/1	-	-	-	-	-	-	-
SETD2	9	8	8/8	-	-	0/1	-	-	-	-
SMAD3	1	0	0/1	-	-	-	-	-	-	-
SMARCB1	6	0	0/6	-	-	-	-	-	-	-
SMARCC2	1	1	1/1	-	-	-	-	-	-	-
SPTAN1	35	35	35/35	-	-	-	-	-	-	-
SUOX	2	1	1/1	-	-	0/1	-	-	-	-
TBCK	2	0	0/2	-	-	-	-	-	-	-
TBX1	2	0	0/2	-	-	-	-	-	-	-
TBX5	3	3	3/3	-	-	-	-	-	-	-
TGFBR1	4	1	1/4	-	-	-	-	-	-	-
UMOD	3	3	2/2	-	1/1	-	-	-	-	-
WWOX	1	1	1/1	-	-	-	-	-	-	-
ZFX	1	1	1/1	-	-	-	-	-	-	-
ZMYM3	1	0	0/1	-	-	-	-	-	-	-

HPO	id	Count	Observed (%)	Expected (%)
Abnormality of the musculoskeletal system	HP:0033127	38	21.8%	17.9%
Abnormality of limbs	HP:0040064	19	10.9%	11.4%
Abnormality of the nervous system	HP:0000707	40	23.0%	10.7%
Abnormality of metabolism/homeostasis	HP:0001939	2	1.1%	9.5%
Abnormality of the genitourinary system	HP:0000119	0	0.0%	6.5%
Abnormality of the cardiovascular system	HP:0001626	11	6.3%	5.7%
Abnormality of head or neck	HP:0000152	8	4.6%	5.6%
Abnormality of the immune system	HP:0002715	5	2.9%	5.3%
Abnormality of the eye	HP:0000478	6	3.4%	4.6%
Abnormality of the integument	HP:0001574	10	5.7%	4.2%
Abnormality of blood and blood-forming tissues	HP:0001871	6	3.4%	3.6%
Abnormality of the digestive system	HP:0025031	3	1.7%	3.3%
Neoplasm	HP:0002664	16	9.2%	2.7%
Abnormality of the respiratory system	HP:0002086	1	0.6%	2.5%
Abnormality of the endocrine system	HP:0000818	1	0.6%	1.8%
Abnormal cellular phenotype	HP:0025354	1	0.6%	1.2%
Abnormality of the ear	HP:0000598	2	1.1%	1.2%
Abnormality of prenatal development or birth	HP:0001197	0	0.0%	0.9%
Constitutional symptom	HP:0025142	0	0.0%	0.6%
Growth abnormality	HP:0001507	5	2.9%	0.4%
Abnormality of the breast	HP:0000769	0	0.0%	0.2%
Abnormality of the voice	HP:0001608	0	0.0%	0.1%
Abnormality of the thoracic cavity	HP:0045027	0	0.0%	0.0%

Table S11: Distribution of significant Fisher exact test results according to top-level HPO term

References

[1] S. Tucci, C. Wagner, S. C. Grünert, U. Matysiak, N. Weinhold, J. Klein, F. Porta, M. Spada, A. Bordugo, G. Rodella, et al. (2021). Genotype and residual enzyme activity in medium-chain acyl-CoA dehydrogenase (MCAD) deficiency: Are predictions possible? en. J. Inherit. Metab. Dis. *44*, 916–925.

[2] M. Halonen, P. Eskelin, A.-G. Myhre, J. Perheentupa, E. S. Husebye, O. Kämpe, F. Rorsman, L. Peltonen, I. Ulmanen, and J. Partanen (2002). AIRE mutations and human leukocyte antigen genotypes as determinants of the autoimmune polyendocrinopathy-candidiasis-ectodermal dystrophy phenotype. en. J. Clin. Endocrinol. Metab. *87*, 2568–2574.

[3] E. Martinez-Cayuelas, F. Blanco-Kelly, F. Lopez-Grondona, S. T. Swafiri, R. Lopez-Rodriguez, R. Losada-Del Pozo, I. Mahillo-Fernandez, B. Moreno, M. Rodrigo-Moreno, D. Casas-Alba, et al. (2023). Clinical description, molecular delineation and genotype-phenotype correlation in 340 patients with KBG syndrome: addition of 67 new patients. en. J. Med. Genet. *60*, 644–654.

[4] Z. Awamleh, S. Choufani, C. Cytrynbaum, F. S. Alkuraya, S. Scherer, S. Fernandes, C. Rosas, P. Louro, P. Dias, M. T. Neves, et al. (2023). ANKRD11 pathogenic variants and 16q24.3 microdeletions share an altered DNA methylation signature in patients with KBG syndrome. Human molecular genetics, *32*, 1429–1438. https://doi.org/10.1093/hmg/ddac289.

[5] A. J. M. Dingemans, K. M. G. Truijen, S. van de Ven, R. Bernier, E. M. H. F. Bongers, A. Bouman, L. de Graaff-Herder, E. E. Eichler, E. H. Gerkes, C. M. De Geus, et al. (2022). The phenotypic spectrum and genotype-phenotype correlations in 106 patients with variants in major autism gene CHD8. en. Transl. Psychiatry, *12*, 421.

[6] M. Konrad, J. Hou, S. Weber, J. Dötsch, J. A. Kari, T. Seeman, E. Kuwertz-Bröking, A. Peco-Antic, V. Tasic, K. Dittrich, et al. (2008). CLDN16 genotype predicts renal decline in familial hypomagnesemia with hypercalciuria and nephrocalcinosis. en. J. Am. Soc. Nephrol. *19*, 171–181.

[7] G. D'Onofrio, A. Accogli, M. Severino, H. Caliskan, T. Kokotović, A. Blazekovic, K. G. Jercic, S. Markovic, T. Zigman, K. Goran, et al. (2023). Genotype-phenotype correlation in contactin-associated protein-like 2 (CNTNAP-2) developmental disorder. en. Hum. Genet. *142*, 909–925.

[8] J. Xie, J. Jiang, and Q. Guo (2021). Primary Coenzyme Q10 Deficiency-7 and Pathogenic COQ4 Variants: Clinical Presentation, Biochemical Analyses, and Treatment. Frontiers in genetics, *12*, 776807. https://doi.org/10.3389/fgene.2021.776807.

[9] H. G. Valverde de Morales, H.-L. V. Wang, K. Garber, X. Cheng, V. G. Corces, and H. Li (2023). Expansion of the genotypic and phenotypic spectrum of CTCF-related disorder guides clinical management: 43 new subjects and a comprehensive literature review. en. Am. J. Med. Genet. A, *191*, 718–729.

[10] C. Xu, W. Jia, X. Cheng, H. Ying, J. Chen, J. Xu, Q. Guan, X. Zhou, D. Zheng, G. Li, et al. (2019). Genotype-phenotype correlation study and mutational and hormonal analysis in a Chinese cohort with 21-hydroxylase deficiency. Molecular genetics and genomic medicine, *7*, e671. https://doi.org/10.1002/mgg3.671.

[11] D. Rots, A. Bouman, A. Yamada, M. Levy, A. J. M. Dingemans, B. B. A. de Vries, M. Ruiterkamp-Versteeg, N. de Leeuw, C. W. Ockeloen, R. Pfundt, et al. (2024). Comprehensive EHMT1 variants analysis broadens genotype-phenotype associations and molecular mechanisms in Kleefstra syndrome. American journal of human genetics, *111*, 1605–1625. https://doi.org/10.1016/j.ajhg.2024.06.008.

[12] Z. J. Frazier, S. Kilic, H. Osika, A. Mo, M. Quinn, S. Ballal, T. Katz, A. E. Shearer, M. A. Horlbeck, L. S. Pais, et al. (2025). Novel Phenotypes and Genotype-Phenotype Correlations in a Large Clinical Cohort of Patients With Kleefstra Syndrome. Clinical genetics. https://doi.org/10.1111/cge.14697.

[13] C. Gracia-Diaz, Y. Zhou, Q. Yang, R. Maroofian, P. Espana-Bonilla, C.-H. Lee, S. Zhang, N. Padilla, R. Fueyo, E. A. Waxman, et al. (2023). Gain and loss of function variants in EZH1 disrupt neurogenesis and cause dominant and recessive neurodevelopmental disorders. en. Nat. Commun. *14*, 4109.

[14] V. M. Becerra-Muñoz, J. J. Gómez-Doblas, C. Porras-Martín, M. Such-Martínez, M. G. Crespo-Leiro, R. Barriales-Villa, E. de Teresa-Galván, M. Jiménez-Navarro, and F. Cabrera-Bueno (2018). The importance of genotype-phenotype correlation in the clinical management of Marfan syndrome. Orphanet journal of rare diseases, *13*, 16. https://doi.org/10.1186/s13023-017-0754-6.

[15] P. Arnaud, O. Milleron, N. Hanna, J. Ropers, N. Ould Ouali, A. Affoune, M. Langeois, L. Eliahou, F. Arnoult, P. Renard, et al. (2021). Clinical relevance of genotype-phenotype correlations beyond vascular events in a cohort study of 1500 Marfan syndrome patients with FBN1 pathogenic variants. Genetics in medicine : official journal of the American College of Medical Genetics, *23*, 1296–1304. https://doi.org/10.1038/s41436-021-01132-x.

[16] A. Hernándiz, A. Zúñiga, F. Valera, D. Domingo, I. Ontoria-Oviedo, J. F. Marí, J. A. Román, I. Calvo, B. Insa, R. Gómez, et al. (2021). Genotype FBN1/phenotype relationship in a cohort of patients with Marfan syndrome. Clinical genetics, *99*, 269–280. https://doi.org/10.1111/cge.13879.

[17] K. A. Groth, Y. Von Kodolitsch, K. Kutsche, M. Gaustadnes, K. Thorsen, N. H. Andersen, and C. H. Gravholt (2017). Evaluating the quality of Marfan genotype-phenotype correlations in existing FBN1 databases. Genetics in medicine : official journal of the American College of Medical Genetics, *19*, 772–777. https://doi.org/10.1038/gim.2016.181.

[18] A. W. El-Hattab, H. Dai, M. Almannai, J. Wang, E. A. Faqeih, A. Al Asmari, M. A. M. Saleh, M. A. O. Elamin, M. Alfadhel, F. S. Alkuraya, et al. (2017). Molecular and clinical spectra of FBXL4 deficiency. en. Hum. Mutat. *38*, 1649–1659.

[19] S. Li, A. Tian, Y. Wen, W. Gu, W. Li, X. Qiao, C. Zhang, and X. Luo (2024). FGD1-related Aarskog-Scott syndrome: Identification of four novel variations and a literature review of clinical and molecular aspects. European journal of pediatrics, *183*, 2257–2272. https://doi.org/10.1007/s00431-024-05484-9.

[20] V. Zanetti Drumond, L. Sousa Salgado, C. Sousa Salgado, V. A. d. L. Oliveira, E. M. de Assis, M. Campos Ribeiro, A. Furtado Valadão, and A. Orrico (2021). The Prevalence of Clinical Features in Patients with Aarskog-Scott Syndrome and Assessment of Genotype-Phenotype Correlation: A Systematic Review. Genetics research, *2021*, 6652957. https://doi.org/10.1155/2021/6652957.

[21] J. J. Johnston, I. Olivos-Glander, C. Killoran, E. Elson, J. T. Turner, K. F. Peters, M. H. Abbott, D. J. Aughton, A. S. Aylsworth, M. J. Bamshad, et al. (2005). Molecular and clinical analyses of Greig cephalopolysyndactyly and Pallister-Hall syndromes: robust phenotype prediction from the type and position of GLI3 mutations. American journal of human genetics, *76*, 609–622. https://doi.org/10.1086/429346.

[22] A. Jamsheer, A. Sowińska, T. Trzeciak, M. Jamsheer-Bratkowska, A. Geppert, and A. Latos-Bieleńska (2012). Expanded mutational spectrum of the GLI3 gene substantiates genotype-phenotype correlations. Journal of applied genetics, *53*, 415–422. https://doi.org/10.1007/s13353-012-0109-x.

[23] R. Patel, S. K. Singh, V. Bhattacharya, and A. Ali (2021). Novel GLI3 pathogenic variants in complex pre- and postaxial polysyndactyly and Greig cephalopolysyndactyly syndrome. American journal of medical genetics. Part A, *185*, 97–104. https://doi.org/10.1002/ajmg.a.61919.

[24] F. Démurger, A. Ichkou, S. Mougou-Zerelli, M. Le Merrer, G. Goudefroye, A.-L. Delezoide, C. Quélin, S. Manouvrier, G. Baujat, M. Fradin, et al. (2015). New insights into genotype-phenotype correlation for GLI3 mutations. European journal of human genetics : EJHG, *23*, 92–102. https://doi.org/10.1038/ejhg.2014.62.

[25] L. G. Biesecker (1997). Strike three for GLI3. Nature genetics, *17*, 259–260. https://doi.org/10.1038/ng1197-259.

[26] M. M. Al-Qattan, H. E. Shamseldin, M. A. Salih, and F. S. Alkuraya (2017). GLI3-related polydactyly: a review. Clinical genetics, *92*, 457–466. https://doi.org/10.1111/cge.12952.

[27] H. L. Sczakiel, W. Hülsemann, M. Holtgrewe, A. T. Abad-Perez, J. Elsner, S. Schwartzmann, D. Horn, M. Spielmann, S. Mundlos, and M. A. Mensah (2021). GLI3 variants causing isolated polysyndactyly are not restricted to the protein's C-terminal third. Clinical genetics, *100*, 758–765. https://doi.org/10.1111/cge.14059.

[28] D. Boutboul, H. S. Kuehn, Z. Van de Wyngaert, J. E. Niemela, I. Callebaut, J. Stoddard, C. Lenoir, V. Barlogis, C. Farnarier, F. Vely, et al. (2018). Dominant-negative IKZF1 mutations cause a T, B, and myeloid cell combined immunodeficiency. The Journal of clinical investigation, *128*, 3071–3087. https://doi.org/10.1172/JCI98164.

[29] M. P. Adam, S. Banka, H. T. Bjornsson, O. Bodamer, A. E. Chudley, J. Harris, H. Kawame, B. C. Lanpher, A. W. Lindsley, G. Merla, et al. (2019). Kabuki syndrome: international consensus diagnostic criteria. Journal of medical genetics, *56*, 89–95. https://doi.org/10.1136/jmedgenet-2018-105625.

[30] P. Makrythanasis, B. W. van Bon, M. Steehouwer, B. Rodríguez-Santiago, M. Simpson, P. Dias, B. M. Anderlid, P. Arts, M. Bhat, B. Augello, et al. (2013). MLL2 mutation detection in 86 patients with Kabuki syndrome: a genotype-phenotype study. Clinical genetics, *84*, 539–545. https://doi.org/10.1111/cge.12081.

[31] Y. Wang, Y. Xu, Y. Chen, Y. Hu, Q. Li, S. Liu, J. Wang, and X. Wang (2024). Sex-specific difference in phenotype of Kabuki syndrome type 2 patients: a matched case-control study. BMC pediatrics, *24*, 133. https://doi.org/10.1186/s12887-024-04562-z.

[32] H. C. Happ, L. G. Sadleir, M. Zemel, G. de Valles-Ibáñez, M. S. Hildebrand, A. McConkie-Rosell, M. McDonald, H. May, T. Sands, V. Aggarwal, et al. (2023). Neurodevelopmental and Epilepsy Phenotypes in Individuals With Missense Variants in the Voltage-Sensing and Pore Domains of KCNH5. Neurology, *100*, e603–e615. https://doi.org/10.1212/WNL.0000000000201492.

[33] V. Faundes, S. Goh, R. Akilapa, H. Bezuidenhout, H. T. Bjornsson, L. Bradley, A. F. Brady, E. Brischoux-Boucher, H. Brunner, S. Bulk, et al. (2021). Clinical delineation, sex differences, and genotype-phenotype correlation in pathogenic KDM6A variants causing X-linked Kabuki syndrome type 2. Genetics in medicine : official journal of the American College of Medical Genetics, *23*, 1202–1210. https://doi.org/10.1038/s41436-021-01119-8.

[34] E. S. Stolerman, E. Francisco, J. L. Stallworth, J. R. Jones, K. G. Monaghan, J. Keller-Ramey, R. Person, I. M. Wentzensen, K. McWalter, B. Keren, et al. (2019). Genetic variants in the KDM6B gene are associated with neurodevelopmental delays and dysmorphic features. American journal of medical genetics. Part A, *179*, 1276–1286. https://doi.org/10.1002/ajmg.a.61173.

[35] G. Captur, E. Arbustini, P. Syrris, D. Radenkovic, B. O'Brien, W. J. Mckenna, and J. C. Moon (2018). Lamin mutation location predicts cardiac phenotype severity: combined analysis of the published literature. Open heart, *5*, e000915. https://doi.org/10.1136/openhrt-2018-000915.

[36] E. W. Lin, G. F. Brady, R. Kwan, A. I. Nesvizhskii, and M. B. Omary (2020). Genotype-phenotype analysis of LMNA-related diseases predicts phenotype-selective alterations in lamin phosphorylation. FASEB journal : official publication of the Federation of American Societies for Experimental Biology, *34*, 9051–9073. https://doi.org/10.1096/fj.202000500R.

[37] A. W. El-Hattab, F.-Y. Li, E. Schmitt, S. Zhang, W. J. Craigen, and L.-J. C. Wong (2010). MPV17-associated hepatocerebral mitochondrial DNA depletion syndrome: new patients and novel mutations. Molecular genetics and metabolism, *99*, 300–308. https://doi.org/10.1016/j.ymgme.2009.10.003.

[38] M. P. Adam, J. Feldman, G. M. Mirzaa, R. A. Pagon, S. E. Wallace, and A. Amemiya (1993). GeneReviews (®).

[39] N. Hammann, D. Lenz, I. Baric, E. Crushell, C. D. Vici, F. Distelmaier, F. Feillet, P. Freisinger, M. Hempel, A. L. Khoreva, et al. (2024). Impact of genetic and non-genetic factors on phenotypic diversity in NBAS-associated disease. Molecular genetics and metabolism, *141*, 108118. https://doi.org/10.1016/j.ymgme.2023.108118.

[40] K. Rojnueangnit, J. Xie, A. Gomes, A. Sharp, T. Callens, Y. Chen, Y. Liu, M. Cochran, M.-A. Abbott, J. Atkin, et al. (2015). High Incidence of Noonan Syndrome Features Including Short Stature and Pulmonic Stenosis in Patients carrying NF1 Missense Mutations Affecting p.Arg1809: Genotype-Phenotype Correlation. Human mutation, *36*, 1052–1063. https://doi.org/10.1002/humu.22832.

[41] M. Upadhyaya, S. M. Huson, M. Davies, N. Thomas, N. Chuzhanova, S. Giovannini, D. G. Evans, E. Howard, B. Kerr, S. Griffiths, et al. (2007). An absence of cutaneous neurofibromas associated with a 3-bp inframe deletion in exon 17 of the NF1 gene (c.2970-2972 delAAT): evidence of a clinically significant NF1 genotype-phenotype correlation. American journal of human genetics, *80*, 140–151. https://doi.org/10.1086/510781.

[42] E. Pasmant, A. Sabbagh, G. Spurlock, I. Laurendeau, E. Grillo, M.-J. Hamel, L. Martin, S. Barbarot, B. Leheup, D. Rodriguez, et al. (2010). NF1 microdeletions in neurofibromatosis type 1: from genotype to phenotype. Human mutation, *31*, E1506–E1518. https://doi.org/10.1002/humu.21271.

[43] D. Nagy, S. Verheyen, K. M. Wigby, A. Borovikov, A. Sharkov, V. Slegesky, A. Larson, C. Fagerberg, C. Brasch-Andersen, M. Kibæk, et al. (2022). Genotype-Phenotype Comparison in POGZ-Related Neurodevelopmental Disorders by Using Clinical Scoring. Genes, *13*. https://doi.org/10.3390/genes13010154.

[44] V. K. Jordan, B. Fregeau, X. Ge, J. Giordano, R. J. Wapner, T. B. Balci, M. T. Carter, J. A. Bernat, A. N. Moccia, A. Srivastava, et al. (2018). Genotype-phenotype correlations in individuals with pathogenic RERE variants. Human mutation, *39*, 666–675. https://doi.org/10.1002/humu.23400.

[45] M. Daich Varela, M. Jeste, T. A. C. de Guimaraes, O. A. Mahroo, G. Arno, A. R. Webster, and M. Michaelides (2024). Clinical, Ophthalmic, and Genetic Characterization of RPGRIP1-Associated Leber Congenital Amaurosis/Early-Onset Severe Retinal Dystrophy. American journal of ophthalmology, *266*, 255–263. https://doi.org/10.1016/j.ajo.2024.05.007.

[46] C. D. Zingariello, D.-H. Chen, W. H. Raskind, W. B. Slayton, S. Subramony, J. Severance, M. Feagle, and S. A. Rasmussen (2024). Assessing Long-Term Neurologic Outcomes in SAMD9L-Related Ataxia-Pancytopenia Syndrome. Movement disorders clinical practice, *11*, 728–733. https://doi.org/10.1002/mdc3.14038.

[47] Y. A. Zarate, C. L. Smith-Hicks, C. Greene, M.-A. Abbott, V. M. Siu, A. R. U. L. Calhoun, A. Pandya, C. Li, E. A. Sellars, J. Kaylor, et al. (2018). Natural history and genotype-phenotype correlations in 72 individuals with SATB2-associated syndrome. American journal of medical genetics. Part A, *176*, 925–935. https://doi.org/10.1002/ajmg.a.38630.

[48] M. Wolff, A. Brunklaus, and S. M. Zuberi (2019). Phenotypic spectrum and genetics of SCN2A-related disorders, treatment options, and outcomes in epilepsy and beyond. Epilepsia, *60 Suppl 3*, S59–S67. https://doi.org/10.1111/epi.14935.

[49] K. Vesela, H. Hansikova, M. Tesarova, P. Martasek, M. Elleder, J. Houstek, and J. Zeman (2004). Clinical, biochemical and molecular analyses of six patients with isolated cytochrome c oxidase deficiency due to mutations in the SCO2 gene. Acta paediatrica (Oslo, Norway : 1992), *93*, 1312–1317. https://doi.org/10.1080/08035250410008761.

[50] E. Van Nieuwenhove, J. S. Barber, J. Neumann, E. Smeets, M. Willemsen, E. Pasciuto, T. Prezzemolo, V. Lagou, L. Seldeslachts, B. Malengier-Devlies, et al. (2020). Defective Sec61α1 underlies a novel cause of autosomal dominant severe congenital neutropenia. en. J. Allergy Clin. Immunol. *146*, 1180–1193.

[51] R. Rabin, A. Radmanesh, I. A. Glass, W. B. Dobyns, K. A. Aldinger, J. T. Shieh, S. Romoser, H. Bombei, L. Dowsett, P. Trapane, et al. (2020). Genotype-phenotype correlation at codon 1740 of SETD2. American journal of medical genetics. Part A, *182*, 2037–2048. https://doi.org/10.1002/ajmg.a.61724.

[52] Z. S. Ulhaq, G. V. Soraya, L. A. Istifiani, S. A. Pamungkas, and W. K. F. Tse (2023). SF3B4 frameshift variants represented a more severe clinical manifestation in Nager syndrome. en. Cleft Palate Craniofac. J. *60*, 1041–1047.

[53] A. Kurisaki, K. Kurisaki, M. Kowanetz, H. Sugino, Y. Yoneda, C.-H. Heldin, and A. Moustakas (2006). The mechanism of nuclear export of Smad3 involves exportin 4 and Ran. en. Mol. Cell. Biol. *26*, 1318–1332.

[54] B. Chesneau, T. Edouard, Y. Dulac, H. Colineaux, M. Langeois, N. Hanna, C. Boileau, P. Arnaud, N. Chassaing, S. Julia, et al. (2020). Clinical and genetic data of 22 new patients with SMAD3 pathogenic variants and review of the literature. en. Mol. Genet. Genomic Med. *8*, e1132.

[55] T. Kosho, N. Okamoto, and Coffin-Siris Syndrome International Collaborators (2014). Genotype-phenotype correlation of Coffin-Siris syndrome caused by mutations in SMARCB1, SMARCA4, SMARCE1, and ARID1A. en. Am. J. Med. Genet. C Semin. Med. Genet. *166C*, 262–275.

[56] E. Bosch, B. Popp, E. Güse, C. Skinner, P. J. van der Sluijs, I. Maystadt, A. M. Pinto, A. Renieri, L. P. Bruno, S. Granata, et al. (2023). Elucidating the clinical and molecular spectrum of SMARCC2-associated NDD in a cohort of 65 affected individuals. en. Genet. Med. *25*, 100950.

[57] A. J. M. Dingemans, K. M. G. Truijen, J.-H. Kim, Z. Alaçam, L. Faivre, K. M. Collins, E. H. Gerkes, M. van Haelst, I. M. B. H. van de Laar, K. Lindstrom, et al. (2022). Establishing the phenotypic spectrum of ZTTK syndrome by analysis of 52 individuals with variants in SON. en. Eur. J. Hum. Genet. *30*, 271–281.

[58] J. Tohyama, M. Nakashima, S. Nabatame, C. Gaik-Siew, R. Miyata, Z. Rener-Primec, M. Kato, N. Matsumoto, and H. Saitsu (2015). SPTAN1 encephalopathy: distinct phenotypes and genotypes. Journal of human genetics, *60*, 167–173. https://doi.org/10.1038/jhg.2015.5.

[59] H. Morsy, M. Benkirane, E. Cali, C. Rocca, K. Zhelcheska, V. Cipriani, E. Galanaki, R. Maroofian, S. Efthymiou, D. Murphy, et al. (2023). Expanding SPTAN1 monoallelic variant associated disorders: From epileptic encephalopathy to pure spastic paraplegia and ataxia. Genetics in medicine : official journal of the American College of Medical Genetics, *25*, 76–89. https://doi.org/10.1016/j.gim.2022.09.013.

[60] L. Van de Vondel, J. De Winter, D. Beijer, G. Coarelli, M. Wayand, R. Palvadeau, M. G. Pauly, K. Klein, M. Rautenberg, L. Guillot-Noël, et al. (2022). De Novo and Dominantly Inherited SPTAN1 Mutations Cause Spastic Paraplegia and Cerebellar Ataxia. Movement disorders : official journal of the Movement Disorder Society, *37*, 1175–1186. https://doi.org/10.1002/mds.28959.

[61] J.-T. Li, Z.-X. Chen, X.-J. Chen, and Y.-X. Jiang (2022). Mutation analysis of SUOX in isolated sulfite oxidase deficiency with ectopia lentis as the presenting feature: insights into genotype-phenotype correlation. Orphanet journal of rare diseases, *17*, 392. https://doi.org/10.1186/s13023-022-02544-x.

[62] E. L. Durham, R. Angireddy, A. Black, A. Melendez-Perez, S. Smith, E. M. Gonzalez, K. G. Navarro, A. Díaz, E. J. K. Bhoj, and K. A. Katsura (2023). TBCK syndrome: a rare multi-organ neurodegenerative disease. en. Trends Mol. Med. *29*, 783–785.

[63] C. Vanlerberghe, A.-S. Jourdain, J. Ghoumid, F. Frenois, A. Mezel, G. Vaksmann, B. Lenne, B. Delobel, N. Porchet, V. Cormier-Daire, et al. (2019). Holt-Oram syndrome: clinical and molecular description of 78 patients with TBX5 variants. en. Eur. J. Hum. Genet. *27*, 360–368.

[64] D. R. Goudie, M. D'Alessandro, B. Merriman, H. Lee, I. Szeverényi, S. Avery, B. D. O'Connor, S. F. Nelson, S. E. Coats, A. Stewart, et al. (2011). Multiple self-healing squamous epithelioma is caused by a disease-specific spectrum of mutations in TGFBR1. Nature genetics, *43*, 365–369. https://doi.org/10.1038/ng.780.

[65] J. L. Moskowitz, S. E. Piret, K. Lhotta, T. M. Kitzler, A. P. Tashman, E. Velez, R. V. Thakker, and P. Kotanko (2013). Association between genotype and phenotype in uromodulin-associated kidney disease. Clinical journal of the American Society of Nephrology : CJASN, *8*, 1349–1357. https://doi.org/10.2215/CJN.11151012.

[66] G. D. Smith, C. Robinson, A. P. Stewart, E. L. Edwards, H. I. Karet, A. G. W. Norden, R. N. Sandford, and F. E. Karet Frankl (2011). Characterization of a recurrent in-frame UMOD indel mutation causing late-onset autosomal dominant end-stage renal failure. Clinical journal of the American Society of Nephrology : CJASN, *6*, 2766–2774. https://doi.org/10.2215/CJN.06820711.

[67] J. Piard, L. Hawkes, M. Milh, L. Villard, R. Borgatti, R. Romaniello, M. Fradin, Y. Capri, D. Héron, M.-C. Nougues, et al. (2019). The phenotypic spectrum of WWOX-related disorders: 20 additional cases of WOREE syndrome and review of the literature. Genetics in medicine : official journal of the American College of Medical Genetics, *21*, 1308–1318. https://doi.org/10.1038/s41436-018-0339-3.

[68] M. Valduga, C. Philippe, L. Lambert, P. Bach-Segura, E. Schmitt, J. P. Masutti, B. François, P. Pinaud, M. Vibert, and P. Jonveaux (2015). WWOX and severe autosomal recessive epileptic encephalopathy: first case in the prenatal period. Journal of human genetics, *60*, 267–271. https://doi.org/10.1038/jhg.2015.17.

[69] C. Mignot, L. Lambert, L. Pasquier, T. Bienvenu, A. Delahaye-Duriez, B. Keren, J. Lefranc, A. Saunier, L. Allou, V. Roth, et al. (2015). WWOX-related encephalopathies: delineation of the phenotypical spectrum and emerging genotype-phenotype correlation. Journal of medical genetics, *52*, 61–70. https://doi.org/10.1136/jmedgenet-2014-102748.

[70] J. L. Shepherdson, K. Hutchison, D. W. Don, G. McGillivray, T.-I. Choi, C. A. Allan, D. J. Amor, S. Banka, D. G. Basel, L. D. Buch, et al. (2024). Variants in ZFX are associated with an X-linked neurodevelopmental disorder with recurrent facial gestalt. American journal of human genetics, *111*, 487–508. https://doi.org/10.1016/j.ajhg.2024.01.007.

[71] B. Guan, S. K. Agarwal, J. M. Welch, S. Jha, L. S. Weinstein, and W. F. Simonds (2024). A Germline ZFX Missense Variant in a Patient With Primary Hyperparathyroidism. JCEM case reports, *2*, luae115. https://doi.org/10.1210/jcemcr/luae115.

[72] A. K. Philips, A. Sirén, K. Avela, M. Somer, M. Peippo, M. Ahvenainen, F. Doagu, M. Arvio, H. Kääriäinen, H. Van Esch, et al. (2014). X-exome sequencing in Finnish families with intellectual disability–four novel mutations and two novel syndromic phenotypes. Orphanet journal of rare diseases, *9*, 49. https://doi.org/10.1186/1750-1172-9-49.

[73] S. M. Hiatt, S. Trajkova, M. R. Sebastiano, E. C. Partridge, F. E. Abidi, A. Anderson, M. Ansar, S. E. Antonarakis, A. Azadi, R. Bachmann-Gagescu, et al. (2023). Deleterious, protein-altering variants in the transcriptional coregulator ZMYM3 in 27 individuals with a neurodevelopmental delay phenotype. American journal of human genetics, *110*, 215–227. https://doi.org/10.1016/j.ajhg.2022.12.007.
