# Supplementary material for: GA4GH phenopacket-driven characterization of genotype-phenotype correlations in Mendelian disorders
Source: Am J Hum Genet. 2025 Dec 23;113(1):57–70. doi: 10.1016/j.ajhg.2025.12.001 (PMC12824607; doi:10.1016/j.ajhg.2025.12.001)
Supplement: Document S2. Article plus supplemental information [file mmc3.pdf]

GA4GH phenopacket-driven characterization of genotype-phenotype correlations in Mendelian disorders

Authors

Lauren Rekerle, Daniel Danis, Filip Rehburg, ..., Christopher J. Mungall, Melissa A. Haendel, Peter N. Robinson

Correspondence

peter.robinson@bih-charite.de

GPSEA is a software tool that uses the GA4GH Phenopacket Schema to streamline discovery of genotype-phenotype correlations (GPCs) in Mendelian diseases. Analyzing data from 85 cohorts of previously published individuals, it identified 253 significant GPCs, demonstrating the power of standardized clinical data for improving clinical management and disease understanding.

Rekerle et al., 2026, The American Journal of Human Genetics *113*, 57–70
January 8, 2026

© 2025 The Author(s). Published by Elsevier Inc. on behalf of American Society of Human Genetics.

https://doi.org/10.1016/j.ajhg.2025.12.001

GA4GH phenopacket-driven characterization of genotype-phenotype correlations in Mendelian disorders

Lauren Rekerle,[1,27] Daniel Danis,[2,27] Filip Rehburg,[2] Adam S.L. Graefe,[2] Viktor Bily,[3] Andrés Caballero-Oteyza,[4,5] Pilar Cacheiro,[6] Leonardo Chimirri,[2] Jessica X. Chong,[7] Evan Connelly,[8] Bert B.A. de Vries,[9] Alexander J.M. Dingemans,[9] Michael H. Duyzend,[10,11,12] Tomas Freiberger,[3] Petra Gehle,[13] Tudor Groza,[14,15,16] Peter Hansen,[2] Julius O.B. Jacobsen,[6] Adam Klocperk,[17] Markus S. Ladewig,[18] Michael I. Love,[8,19] Allison J. Marcello,[7] Alexander Mordhorst,[20] Monica C. Munoz-Torres,[21] Justin Reese,[22] Catharina Schuetz,[23,26] Damian Smedley,[6] Timmy Strauss,[23] Ondrej Vladyka,[17] David Zocche,[24] Sylvia Thun,[2] Christopher J. Mungall,[22] Melissa A. Haendel,[8] and Peter N. Robinson[1,2,25,*]

Summary

Comprehensively characterizing genotype-phenotype correlations (GPCs) in Mendelian disease would create new opportunities for improving clinical management and understanding disease biology. However, heterogeneous approaches to data sharing, reuse, and analysis have hindered progress in the field. We developed Genotype-Phenotype Statistical Evaluation of Associations (GPSEA), a software package that leverages the Global Alliance for Genomics and Health (GA4GH) Phenopacket Schema to represent case-level clinical and genetic data about individuals. GPSEA applies an independent filtering strategy to boost statistical power to detect categorical GPCs represented by Human Phenotype Ontology terms. GPSEA additionally enables visualization and analysis of continuous phenotypes, clinical severity scores, and survival data such as age of onset of disease or clinical manifestations. We applied GPSEA to 85 cohorts with 6,179 previously published individuals with variants in one of 81 genes associated with 122 Mendelian diseases and identified 253 significant GPCs, with 48 cohorts having at least one statistically significant GPC. These results highlight the power of standardized representations of clinical data for scalable discovery of GPCs in Mendelian disease.

Introduction

There are a huge number of clinical manifestations of human disease, and even individuals with the same clinical diagnosis may present with different combinations of phenotypic abnormalities, ages of onset of these abnormalities, and degrees of clinical severity. A key question for genomic precision medicine is how specific genetic variants influence clinical phenotype. The correlation between genotype (the type of variant or variants present at a given location) and phenotype (presence or absence of medically relevant observable traits) is defined as an above-chance probability of an association between the two and is termed genotype-phenotype correlation (GPC).[1] Commonly, even individuals with an identical pathogenic variant may display variable findings, so GPCs are rarely absolute. Instead, GPCs usually signify a higher frequency of a feature in the presence of a certain genotype or an earlier age of onset of the disease or disease feature, or in some cases earlier mortality. For instance, a

[1]The Jackson Laboratory for Genomic Medicine, 10 Discovery Drive, Farmington CT 06032, USA; [2]Berlin Institute of Health at Charité, Universitätsmedizin Berlin, Berlin, Germany; [3]Centre of Cardiovascular Surgery and Transplantation Brno and Faculty of Medicine, Masaryk University, Brno, Czech Republic; [4]Clinic for Immunology and Rheumatology, Hanover Medical School, Hanover, Germany; [5]Center for Chronic Immunodeficiency, University Hospital Freiburg, Satellite Center of RESiST-Cluster of Excellence 2155, Hanover Medical School, Hanover, Germany; [6]William Harvey Research Institute, Faculty of Medicine and Dentistry, Queen Mary University of London, Charterhouse Square, London EC1M 6BQ, UK; [7]Department of Pediatrics, Division of Genetic Medicine, University of Washington, 1959 NE Pacific Street, Box 357371, Seattle, WA 98195, USA; [8]Department of Genetics, University of North Carolina Chapel Hill, Chapel Hill, NC, USA; [9]Department of Human Genetics, Donders Institute for Brain, Cognition and Behaviour, Radboud University Medical Center, P.O. Box 9101, 6500 HB Nijmegen, the Netherlands; [10]Center for Genomic Medicine, Massachusetts General Hospital, Boston, MA, USA; [11]Division of Genetics and Genomics, Boston Children's Hospital, Boston, MA, USA; [12]Program in Medical and Population Genetics, Broad Institute of MIT and Harvard, Cambridge, MA, USA; [13]Deutsches Herzzentrum der Charité, Berlin, Germany; [14]Bioinformatics Institute, Agency for Science, Technology and Research (A*STAR), 30 Biopolis Street #07-01 Matrix, Singapore 138671, Singapore; [15]Rare Care Centre, Perth Children's Hospital, Nedlands, WA 6009, Australia; [16]SingHealth Duke-NUS Institute of Precision Medicine, 5 Hospital Drive Level 9, Singapore 169609, Singapore; [17]Department of Immunology, 2nd Faculty of Medicine, Charles University and University Hospital in Motol, Prague, Czech Republic; [18]Department of Ophthalmology, University Clinic Marburg – Campus Fulda, Fulda, Germany; [19]Department of Biostatistics, University of North Carolina Chapel Hill, Chapel Hill, NC, USA; [20]Institute of Medical and Human Genetics, Charité Universitätsmedizin Berlin, Berlin, Germany; [21]Department of Biomedical Informatics, University of Colorado Anschutz Medical Campus, Aurora, CO 80045, USA; [22]Division of Environmental Genomics and Systems Biology, Lawrence Berkeley National Laboratory, 1 Cyclotron Road, Berkeley, CA 94720, USA; [23]Department of Pediatrics, Faculty of Medicine and University Hospital Carl Gustav Carus, Technische Universität Dresden, Dresden, Germany; [24]North West Thames Regional Genetics Service, Northwick Park & St Mark's Hospitals, London, UK; [25]ELLIS – the European Laboratory for Learning and Intelligent Systems, Tübingen, Germany; [26]German Center for Child and Adolescent Health, Partner Site Leipzig/Dresden, Dresden, Germany
[27]These authors contributed equally
*Correspondence: peter.robinson@bih-charite.de
https://doi.org/10.1016/j.ajhg.2025.12.001.
© 2025 The Author(s). Published by Elsevier Inc. on behalf of American Society of Human Genetics.
This is an open access article under the CC BY license (http://creativecommons.org/licenses/by/4.0/).

specific in-frame deletion of codon 992 of *NF1* (MIM: 613113) is associated with a milder phenotype characterized by café-au-lait spots and skinfold freckling and with the absence of cutaneous and visible plexiform neurofibromas, whereas individuals with missense mutations affecting any of the five codons 844–848 have a more severe phenotype characterized by a high prevalence of plexiform neurofibromas, optic pathway gliomas, malignant neoplasms, and skeletal abnormalities.[2] A core paradigm in precision genomic medicine is to match therapeutic interventions and other forms of clinical care to the pathomechanism of disease and, where appropriate, to specific genetic variants. Although this paradigm has been extremely successful in oncology, where targeted therapies are applied to treat cancer types if a certain genetic variant is identified, this approach has been less successful in Mendelian disease.[3] Historically, it has been difficult to identify GPCs for rare Mendelian diseases because the rarity of diseases implies it is generally difficult or impossible to recruit cohorts large enough to achieve statistical power.

The Human Phenotype Ontology (HPO) is a comprehensive bioinformatics resource for the analysis of human diseases and phenotypes, offering a computational bridge between genome biology and clinical medicine, and is used internationally for analysis and exchange of phenotype data in rare disease medicine.[4–6] A growing number of published works leverage HPO-encoded data for GPC analysis.[7–21] However, there are challenges to using HPO for GPCs because of the need to propagate annotations up the hierarchy of the HPO and to include only explicitly observed or excluded HPO terms in categorical testing, both being operations that are not natively supported by standard spreadsheet tools or bioinformatics packages. Additionally, it is desirable to integrate the analysis with other kinds of clinical data including numerical measurements, age of onset or mortality, and severity scoring. In addition, the community has lacked a common schema for representing individual (i.e., case-level) clinical trajectories with these and other attributes. Heterogeneous approaches to data sharing, reuse, and analysis have hindered development of software packages and data repositories that support GPC analysis. The Global Alliance for Genomics and Health (GA4GH) is an organization developing a suite of coordinated standards for genomics. The GA4GH Phenopacket Schema is a standard for sharing disease and phenotype information characterizing an individual person or biosample that addresses the challenge of documenting case-level clinical information.[22–25] Here, we present Genotype-Phenotype Statistical Evaluation of Associations (GPSEA). GPSEA leverages phenopackets, characterizing an individual person or biosample and linking the individual to detailed phenotypic descriptions, genetic information, diagnoses, and treatments.[22] GPSEA automates the process of visualizing and performing GPC analysis. We applied the software to 85 cohorts, 48 (56%) of which had at least one statistically significant GPC (there were a total of 253 statistically significant results). We show the power of utilizing individual-level characterization and discuss future utility in differential diagnostics and precision medicine.

Material and methods

Input data

Genotypic and phenotypic data about individuals with rare Mendelian disease were derived from the Phenopacket Store repository.[25] Version 0.1.25 of Phenopacket Store includes 8,207 phenopackets representing 521 Mendelian and chromosomal diseases associated with 463 genes and 4,507 unique pathogenic alleles curated from 1,238 different publications. The phenopackets are structured representations of data comprising age of onset and age at last examination, vital status, genotype of the variant(s) deemed to be causal, disease diagnosis, and HPO terms representing the clinical manifestations of the disease. Where available in the original publication, the age of onset of the clinical manifestations is indicated. Missing data were not imputed.

For each GPC analysis, a cohort was defined based on the gene or disease. The analysis code for each cohort is available in the project GitHub repository (see data and code availability). The phenopackets used for analysis are automatically imported from Phenopacket Store by each cohort notebook. If desired, the phenopackets can also be obtained directly from Phenopacket Store.

GPSEA

GPSEA is a Python package for streamlining GPC analysis. For the analysis described here, version 0.9.11 was used. GPSEA enables a stepwise workflow to characterize GPCs. (1) A collection of phenopackets is loaded into a cohort, and a report with basic descriptive statistics is displayed. The report comprises the number of individuals and the distribution of sex and age, as well as tables with the most commonly annotated HPO terms, diseases, and associated genes. The variants are summarized according to their frequency and the predicted effect on the clinically relevant transcript, including a graphic with the location and frequencies of all non-structural variants. (2) The user can generate hypotheses about the GPC analyses that are most likely to be fruitful. For instance, if roughly half of the variants are missense, then it might make sense to test whether missense variants are associated with different clinical manifestations as compared to other variants. (3) The analysis is configured with respect to multiple testing correction and other parameters. (4) A hypothesis is expressed with GPSEA's variant predicates and genotype classifiers to define a partitioning of the cohort by genotype for testing with four statistical approaches. (5) The analysis results are presented as figures and tables. The data used to perform the statistical tests can be exported as data frames for additional analysis. A detailed tutorial is available online (see data and code availability).

Statistical tests

GPSEA provides four main statistical tests, each of which can be combined with predicates to test a wide variety of GPCs. For each test performed, GPSEA checks whether data are available for each individual (observed or excluded in the case of HPO-term-based tests, numerical measurement results for the *t* test, and duration until event for survival analysis) and omits a data

		Missense	Stop gained
Strabismus	observed	17	6
	excluded	1	19

Figure 1. Computation of association between a pair of dichotomous variables
In this example, a Fisher exact test (FET) is used to assess the association of *Strabismus* with missense variants compared to stop-gained (nonsense) variants. A *p* value of 5.43×10^{-6} is obtained, meaning there is a significant difference between the groups.

point if either phenotypic or genotypic information is not available or applicable. By default, associations with adjusted *p* values of less than 0.05 are considered to be statistically significant.

Dichotomous qualitative phenotypes

The Fisher exact test (FET) calculates the exact probability value for the relationship between two dichotomous variables. In our implementation, the two dichotomous variables are the genotype and the phenotype. For instance, the individuals of the cohort may be divided according to whether they have a missense or a stop-gained (nonsense) variant and according to whether or not they have *Strabismus* (HP:0000486) (Figure 1).

Multiple testing correction

For the FET procedure, GPSEA can perform one FET for each HPO term identified in the cohort. However, in some cohorts, up to hundreds of terms are identified. GPSEA offers two approaches toward controlling the type I error rate, i.e., the probability of rejecting the null hypothesis when it is true. First, 11 classical multiple testing correction procedures are offered including Bonferroni and Benjamini-Hochberg (BH). A multiple-testing correction (MTC) procedure (by default BH) is applied to each test result, and the adjusted *p* value is reported.

The multiple testing burden can also be reduced by selecting only a subset of terms to test. If the user has a hypothesis about which HPO terms are involved in a GPC, then GPSEA can be instructed to test only this term (or subset of terms).

Independent filtering for Human Phenotype Ontology: IF-HPO

Additionally, we developed the IF-HPO procedure that applies a series of heuristics to select terms to test. The procedure was inspired by analogous strategies used in functional genomics that consist in filtering by a variable that is independent of the test statistic under the null hypothesis. By reducing the number of tests in this way, we maximize power for differential testing while preserving type I error control.[26] (1) Skip "general"-level terms. All the direct children of the root phenotype term *Phenotypic abnormality* are skipped because of the assumption that if there is a valid signal, it will derive from one of the more specific descendents. For instance, *Abnormality of the nervous system* (HP:0000707) is a child of *Phenotypic abnormality*, and this assumption implies that if there is a signal from the nervous system, it will lead to at least one of the descendants of *Abnormality of the nervous system* being significant. The top-level terms as well as their child and grandchild terms are skipped in this way, because they all represent general group terms. Details are available in the online tutorial. (2) Skip terms if all counts are identical to counts for a child term. Let's say a term such as *Posterior polar cataract* (HP:0001115) was observed in seven of 11 individuals with MISSENSE variants and in three of eight individuals with NONSENSE variants. If we find the same individual counts (7 of 11 and 3 of 8) in the parent term *Polar cataract* (HP:0010696), then we choose to not test the parent term. This is because the more specific an HPO term, the more information it has (the more interesting the correlation would be if it exists), and the result of the FET for *Polar cataract* would be exactly the same as for *Posterior polar cataract*. (3) Skip terms that are reported in less than a certain proportion of cohort members (default 0.4), because even if a correlation is identified it is unlikely to be of great interest, as the phenotype in question occurs rarely. (4) If the individuals are binned into two genotype groups and two phenotype groups (2×2) and the total count of individuals is less than 7, or into three genotype groups and two phenotype groups (3×2) and the total count of individuals is less than 6, then there is a lack of even nominal statistical power and the counts can never be significant. (5) Skip terms if there are no HPO observations in a genotype class. If one of the genotype classes has neither observed nor excluded observations for an HPO term, skip it. This situation suggests that the data are not sufficiently rich to confidently perform a test.

Phenotype scores

It is difficult to define an objective measure for the clinical severity of disease. Some published studies use the total count of features from a defined set as a proxy for severity. For instance, Jordan et al.[27] found that the total number of structural defects of the brain, eye, heart, and kidney and in sensorineural hearing loss seen in individuals with point mutations in the Atrophin domain of the *RERE* (MIM: 605226) is significantly higher than expected, compared to the number of defects seen in individuals with putative loss-of-function (LoF) variants. Since there are five potential defects, each individual has a count ranging between 0 and 5. The authors regarded higher counts as representative of a severe clinical presentation.[27]

GPSEA performs a Mann-Whitney U test (also known as a Wilcoxon rank-sum test) to compare the distribution of such counts between genotype classes. This is a non-parametric test that compares the class medians to determine whether they come from the same distribution.

A set of HPO terms that define the severity score is entered. GPSEA increments the total count by one for each of the terms (or more specific descendant terms) to which an individual is annotated in the phenopacket. If multiple HPO terms are found to be related to one of the specified terms, then only one count is incremented (e.g., if both *Ventricular septal defect* [HP:0001629] and *Atrial septal defect* [HP:0001631] are identified, a score of 1 and not 2 is entered for *Abnormal heart morphology* [HP:0001627]).

The de Vries score is a simple phenotypic severity score for individuals with intellectual disability in which points are given for (severity of) intellectual disability, growth abnormalities (prenatal and postnatal), facial dysmorphisms, non-facial dysmorphisms, and other congenital anomalies.[13,28] Our implementation of the de Vries score leverages the hierarchical structure of the HPO to include more specific descendants of phenotypic abnormalities included in the original score. For instance, *Disproportionate short stature* (HP:0003498) would be counted for *Short stature* (HP:0004322).

GPSEA can also make use of user-defined functions to support a plethora of scoring schemes used in different clinical domains. A scoring function is required to "condense" the phenotype of the individual into a numeric score or return a "not a number" (NaN) value if the individual should be excluded from the analysis. We provide examples for using user-defined functions as well as defining custom phenotype scorers in GPSEA documentation.

Genotype-specific survival analysis

We may wish to compare the genotype classes with respect to the time point of a specific event, such as age of onset, age at death, or age at onset of a specified phenotypic feature such as kidney failure. To do this, GPSEA tabulates the age of the event (if the event was observed) or whether the individual was still alive (based on the time of last evaluation) without the event having occurred—that is, the survival time was right censored. The log-rank test is used to test the null hypothesis that there is no difference between the populations in the probability of an event at any time point.[29]

Student's t *test for numerical values*

GPSEA performs an unpaired and two-sided *t* test to compare the means of the two groups defined by the genotype classifier. GPSEA expects numerical data for this test to be made available as measurement elements in the GA4GH Phenopacket Schema. GPSEA does not stipulate any specific ontology to represent the measurements, but in our examples we use LOINC codes to denote the assay and UCUM codes to represent units. GPSEA does not apply multiple-testing correction to these results, and users need to perform one analysis for each measurement to be tested.

Variant predicates

GPSEA has flexible predicates (functions that return either true or false based on the input) that can be used to partition the cohort into (usually) two groups of individuals. The predicates can be combined using "AND," "OR," and "NOT" operators of Boolean algebra logic to test complex conditions. GPSEA offers predicates for specific variants, for variant effect categories such as missense and stop-gained variants, specific exons, protein regions, types of structural variant, and others (Table S1). Besides the off-the-shelf predicates, custom predicates for testing arbitrary variant properties can be designed.

Genotype classifiers

GPSEA has five classifiers. The mono-allelic classifiers can be used to investigate autosomal-dominant (heterozygous variants) and X chromosomal diseases (hemizygous variants; if desired cohorts of males and females can be analyzed, in which case the mono-allelic classifier would identify hemizygous variants in males and heterozygous variants in females). For instance, a mono-allelic classifier might partition individuals according to whether they have a heterozygous missense variant (group A) or not (group B); another classifier might partition individuals according to whether they have a heterozygous missense variant (group A) or a heterozygous structural variant (group B). In some cases, a genotype classifier might omit certain individuals; for instance, in the previous example, the classifier would omit any individual who does not have either a missense or a structural variant, or any individuals with homozygous or compound heterozygous genotypes for missense or structural variants. The bi-allelic classifier is designed for autosomal-recessive conditions; it is possible to test three genotypes (e.g., AA, AB, and BB, where A refers to a genotype such as "stop-gained variant" and B refers to other variants), which is a 3×2 contingency table that can be analyzed by FET. Alternatively, it is possible to form two groups (e.g., AA and AB vs. BB, i.e., one or two stop-gained alleles vs. no stop-gained allele). The online documentation shows how to define partitions to perform these tests. Any of the variant predicates can be used together with the mono-allelic and bi-allelic genotype classifiers.

The disease classifier is designed to assay differences between the phenotypic features of two different diseases. For instance, in our cohort, we test for differences between Loeys-Dietz syndrome 1 (MIM: 609192) and Loeys-Dietz syndrome 3 (MIM: 613795) as well as between autosomal-recessive and -dominant forms of Robinow syndrome (MIM: 268310 and 616331). The sex classifier is designed to test differences between the phenotypic features observed in the males and females of a cohort. Individuals with unknown or unspecified sex are ignored by this classifier. The allele count classifier is designed to assay differences between individuals with one and two variant alleles in the same gene. For instance, in our cohort, we test whether individuals with mono-allelic and bi-allelic variants in *EZH1* (MIM: 601674) have distinct phenotypic profiles. This disease, sex, and allele count classifiers do not take genotype into account.

Visualization

GPSEA visualizes variants against the background of the protein domain structure. To do so, it leverages the UniProt[30] application programming interface (API) to retrieve information about protein domains. It is also possible to manually construct a dataframe with information about protein domains in cases where the UniProt API fails or does not contain information about a domain of interest. GPSEA then extracts information about all variants found in the cohort and plots each variant as a "lollipop" whose height and size reflect the number of times the variant was found in the cohort and whose color represents the functional effect predicted for the transcript of interest. Protein domains are depicted as colored boxes. Currently, GPSEA does not display non-coding or structural variants.

Cohorts

A total of 85 cohorts were chosen for GPSEA analysis from version 0.1.25 of Phenopacket Store.[25] The cohorts had a mean of 77.8 individuals (median 49, minimum 16, and maximum 462). The cohorts comprised information from 6,179 individuals. Information on the sex of participants was available for 82.6% of these individuals, with 53% being male and 47% female.

To use GPSEA with new cohorts, it will be necessary to convert data to GA4GH Phenopacket Schema format. Several software tools are available to streamline this process.[23,25]

Search for previously published genotype-phenotype correlations

For each of the gene-specific cohorts, we searched for publications that described genotype-phenotype correlations in PubMed. Each search was designed as {Disease name synonyms} AND {gene/variant synonyms} AND {genotype-phenotype correlation}. The following is an example for Loeys-Dietz syndrome 3.

```
("Loeys-Dietz syndrome type 3" OR LDS3 OR "Loeys-Dietz syndrome 3") AND
(SMAD3 OR variant OR mutation) AND
("genotype phenotype correlation" OR "phenotype genotype correlation")
```

Additionally, the relevant entries from Online Mendelian Inheritance in Man (OMIM)[31] were consulted as were the publications used for curation.

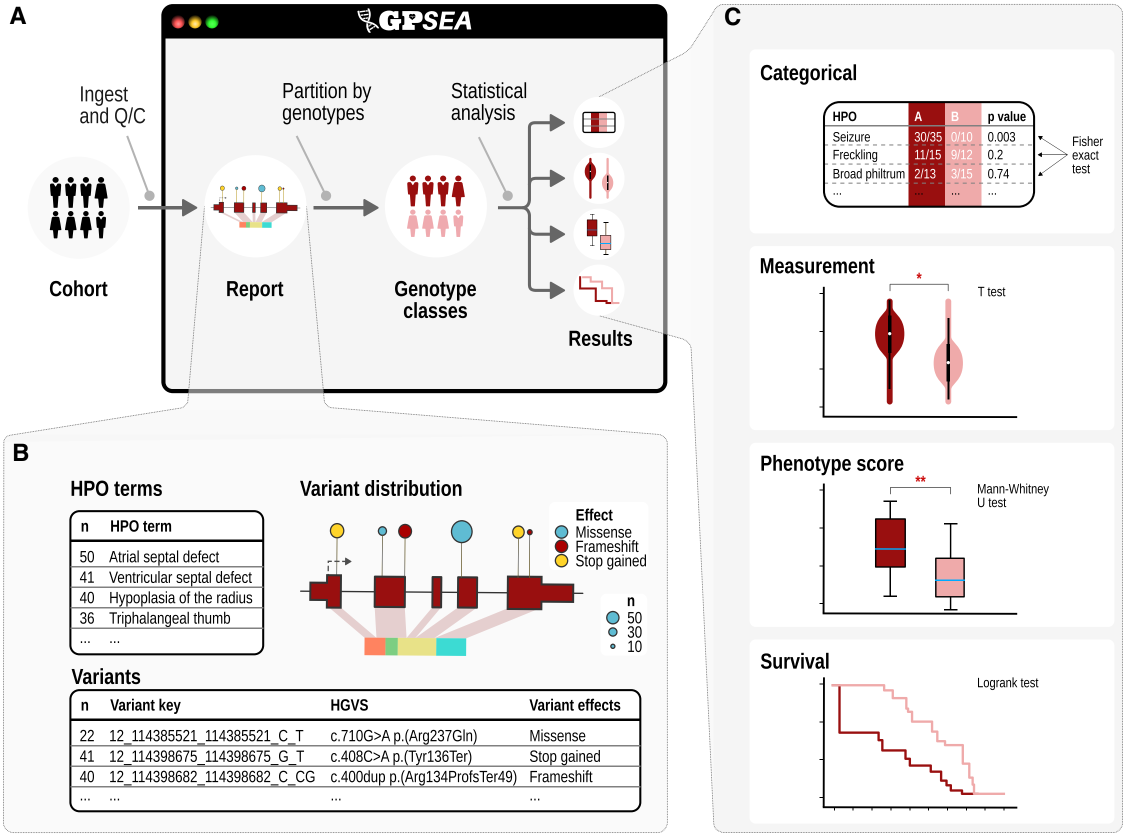

Figure 2. Schematic overview of GPSEA workflow

(A) Overview. GPSEA is a Python package designed to work well in Jupyter notebooks. GPSEA takes a collection of GA4GH phenopackets as input, performs quality assessment, and visualizes the salient characteristics of the cohort; genotype classes are defined (Figure 3); and one of four classes of statistical test is performed for each hypothesis the user decides to test.

(B) Visualizing data and formulating hypotheses. GPSEA displays tables with the distribution of phenotypic abnormalities, disease diagnoses, variants, and other information, and presents a cartoon with the distribution of variants across the protein. This information intends to help users formulate hypotheses about genotype-phenotype correlations.

(C) Statistical testing. GPSEA offers four main ways of testing phenotypes (see text for details and Figure 5 for examples).

Results

Characterizing genotype-phenotype correlations with GPSEA

We developed GPSEA as an end-to-end software framework for exploring and visualizing the cohorts and for characterizing GPCs. GPSEA is a Python package designed to be used in a Jupyter notebook, but the analysis functions can also be used as a programming library. The GPSEA framework enables testing of existing hypotheses about the disease or gene in question or generation of new hypotheses based on salient aspects of the investigated cohort depicted by the tables and visualizations. Genotypes can be tested for association with four main classes of clinical phenotype data: categorical phenotypic traits (i.e., observed vs. excluded HPO terms or disease diagnoses), numerical values (e.g., laboratory test results), phenotype scores, and survival data (i.e., mortality, disease onset, or onset of a specific HPO term). GPSEA enables definition of reproducible analyses that combine flexible partitioning of the individuals into genotype/phenotype groups followed by standard statistical tests (e.g., FET for categorical phenotypes). Importantly, GPSEA exploits the HPO hierarchy and propagates the HPO annotations when computing the contingency tables and distribution of phenotype scores or the survival data or filtering the HPO terms to reduce the multiple testing burden. All analysis results are formatted as tables and figures suitable for processing in bioinformatics pipelines or interactive exploration within the Python data science environment (Figure 2). We tested GPSEA on 85 cohorts, covering 81 genes and 122 diseases. We first explain the algorithmic approaches to setting up GPC testing and then present an overview of 253 significant correlations identified in the cohorts.

Partitioning the cohort according to genotypes

The GPC analysis starts with defining one or more hypotheses. Ideally, decisions regarding the analysis structure will be based on prior hypotheses about the disease or gene in question. Alternatively or additionally, GPSEA helps users generate hypotheses based on the tables and visualizations. For instance, if GPSEA shows that roughly 50% of the variants observed in a cohort are missense

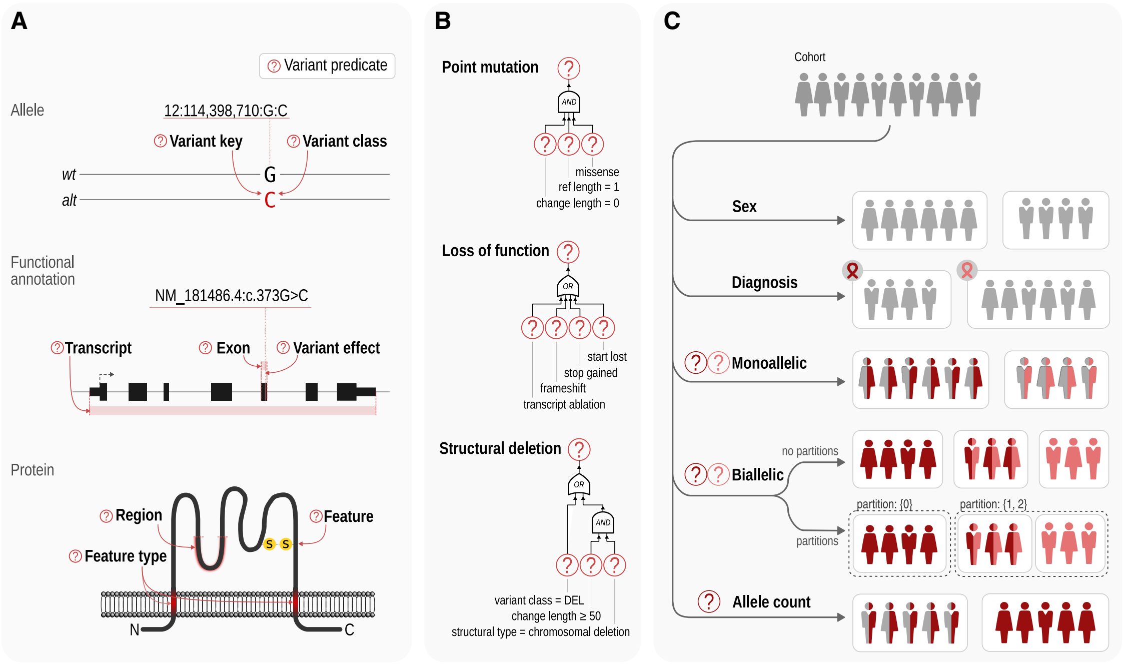

Figure 3. Variant predicates and genotype classifiers

(A) Variant predicate tests. GPSEA provides predicate functions that test whether a variant, such as c.373G>C (GenBank: NM_181486.4) (p.Gly125Arg) in *TBX5* (MIM: 601620), meets a criterion from one of three evidence groups: allele, functional annotation, or protein. For instance, the predicate checks if the variant is a deletion and whether it overlaps with a specific exon or with a protein region of interest.

(B) Boolean algebra. Variant predicates can be combined using AND, OR, and NOT operators of Boolean algebra to test complex criteria. For instance, a predicate for a point mutation can be formulated as a "missense mutation affecting one reference base and change length of zero" (no sequence loss or gain). A predicate for a loss-of-function mutation can be defined as a mutation leading to a transcript ablation, frameshift, introduction of a premature stop codon, or the start codon loss. A predicate for a structural deletion can test whether the variant is either an imprecise chromosomal deletion or a deletion involving 50 or more base pairs (or other thresholds).[33]

(C) Genotype classifiers. Each classifier splits a cohort into two or more classes to enable genotype-phenotype comparisons. GPSEA ships with five built-in classifiers to classify the cohort members using their sex, diagnosis, a fixed count of alleles of different types (mono-allelic and bi-allelic), or by a different allele count of the same type (allele count).

and the other 50% are truncation or presumed LoF variants, users may choose to analyze whether missense are associated with significantly different phenotypes than LoF variants. Other GPSEA visualizations may help to formulate hypotheses about commonly occurring variants, protein domains, exons, or other classes of variation. Once a hypothesis has been conceived, it must be encoded into a genotype classifier, i.e., a GPSEA component that assigns each cohort member into one of the (typically two) classes.

Variant predicates are key building blocks for classification based on genomic variants. GPSEA offers predicates for specific variants, variant effect categories such as missense and stop-gained variants, specific exons, protein regions, and types of structural variant and others. Predicates can be combined using Boolean algebra to create more expressive predicates. The framework provides five genotype classifiers that are used to divide the cohort into groups for statistical testing. The mono- and bi-allelic classifiers use variant predicates to select the variants of interest and assign individuals to genotype classes. The sex classifier investigates differences between males and females. The diagnosis classifier tests for differences in the phenotypic spectrum of different diseases (e.g., Loeys-Dietz syndrome 1 [MIM: 609192] vs. Loeys-Dietz syndrome 2 [MIM: 610168]). The allele count classifier takes a variant predicate to select the variants of interest and classify the individuals according to the number of variant alleles. For instance, mono-allelic and bi-allelic variants in *EZH1* cause dominant and recessive neurodevelopmental disorders[32]; with the allele count classifier, the distribution of phenotypic features can be compared between individuals with mono-allelic and bi-allelic variants (Figure 3 and Table S1).

Analyzing the cohort according to phenotypes

GPSEA offers four major tests for different kinds of clinical data. The categorical test is designed to be used for observations of HPO terms (observed/excluded) or disease diagnoses. Numerical values such as laboratory measurements can be analyzed with a *t* test. Phenotype scores can be derived as a proxy of clinical severity and are analyzed by a Mann-Whitney U test. Finally, survival analysis can be performed for age of disease onset, onset of a phenotypic feature (HPO term), or death. The following sections explain the approach. The examples are taken from the 85

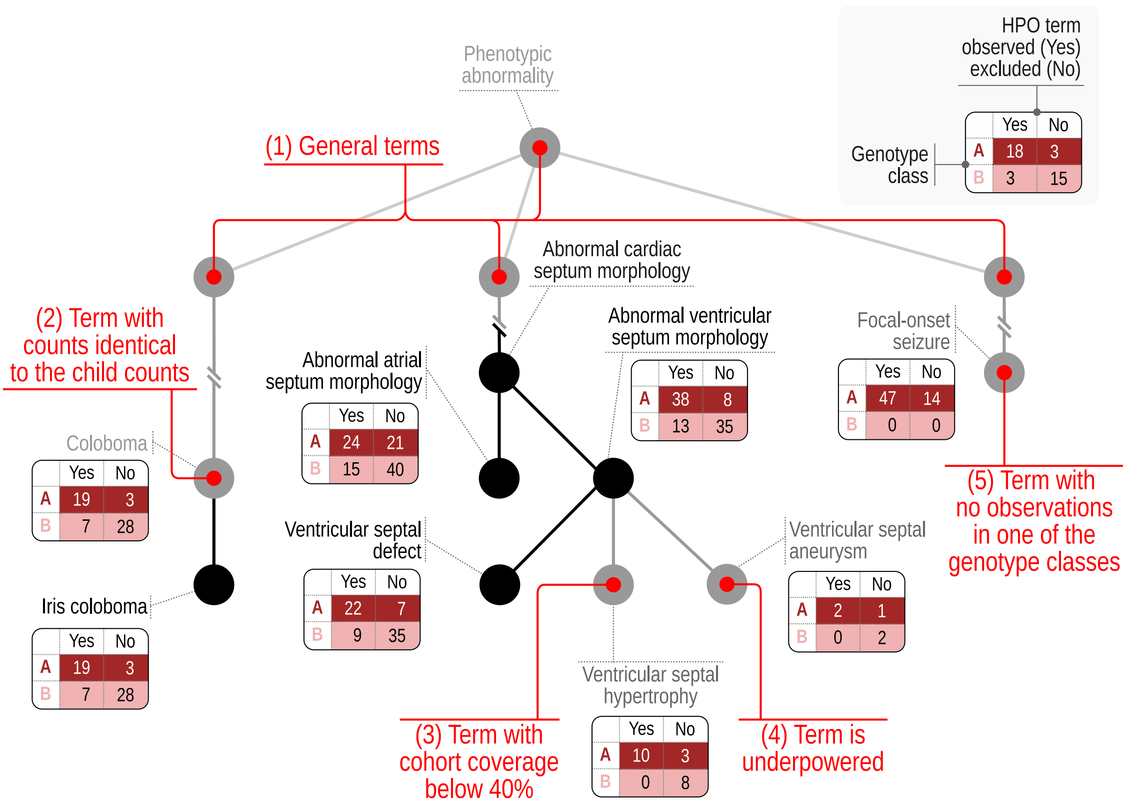

Figure 4. Independent filtering for human phenotype ontology
Independent filtering for HPO (IF-HPO) removes hypotheses (here, HPO terms) by criteria independent of the test statistic to reduce the multiple testing burden and boost power. The HPO has a hierarchical structure going from general to specific terms. (1) IF-HPO does not test the top two levels of the HPO under the *Phenotypic abnormality* root or the terms that are not descendants of the *Phenotypic abnormality* under the assumption that more specific terms are of higher medical and scientific interest and the signal is likely to be driven by a more specific clinical manifestation. (2) Terms are not tested if they have the exact same counts as one of their child terms, because in this case the annotations of the parent term are derived entirely from those of the child term by the true path rule. (3) Terms are not tested if the coverage is less than 40% of the entire cohort (assuming a cohort of 100 individuals in the figure), under the assumption that the result would not be representative of the cohort. (4) Terms are not tested if the total count is below a threshold for reaching the nominal statistical power. (5) Finally, terms are not tested if one of the genotype classes has neither present nor excluded observations.

cohorts (for an overview, see Tables S2 and S3–S10; for a summary of results for each cohort, see Figures S1–S88; for source code for analyses, see data and code availability).

Categorical association

The FET calculates the exact probability for observing as extreme a contingency table for the relationship between two categorical variables, if in fact they are independent. In our implementation, the two categorical variables are the genotype and the phenotype. For instance, the individuals of the cohort may be divided according to whether or not they have a stop-gained (nonsense) variant and according to whether or not they have *Strabismus*.

IF-HPO

Larger cohorts may include several hundreds of HPO terms. Even though many published articles on GPC analysis do not apply an MTC to the tests, we feel it is appropriate to do so unless users have a well-defined hypothesis prior to performing the analysis. In the cohorts analyzed here, up to hundreds of HPO terms are used, so MTC can result in low statistical power. In high-dimensional data such as analysis of mRNA expression in cohorts, a two-stage approach termed independent filtering prefilters hypotheses (e.g., expression differences per gene) by a criterion independent of the test statistic under the null hypothesis, before testing any hypotheses, to reduce the number of the hypotheses tested at stage 2, leading to a milder MTC effect and, thereby, increased power.[26] We developed an analogous approach, IF-HPO, to reduce the testing burden before MTC is applied (Figure 4). This rule-based approach leverages the hierarchical structure of the HPO to avoid unnecessary tests and the tests that are unlikely to reveal an interesting result. IF-HPO reduces the total number of tested terms by over 10-fold in the cohorts analyzed here (before filtering: mean 304, median 277, minimum 45, maximum 967; following independent filtering: mean 40, median 28, minimum 1, maximum 225). While any such heuristic has its trade-offs and it is possible that some significant and interesting results are removed, the IF-HPO procedure provides a substantial boost in statistical power for the remaining terms.

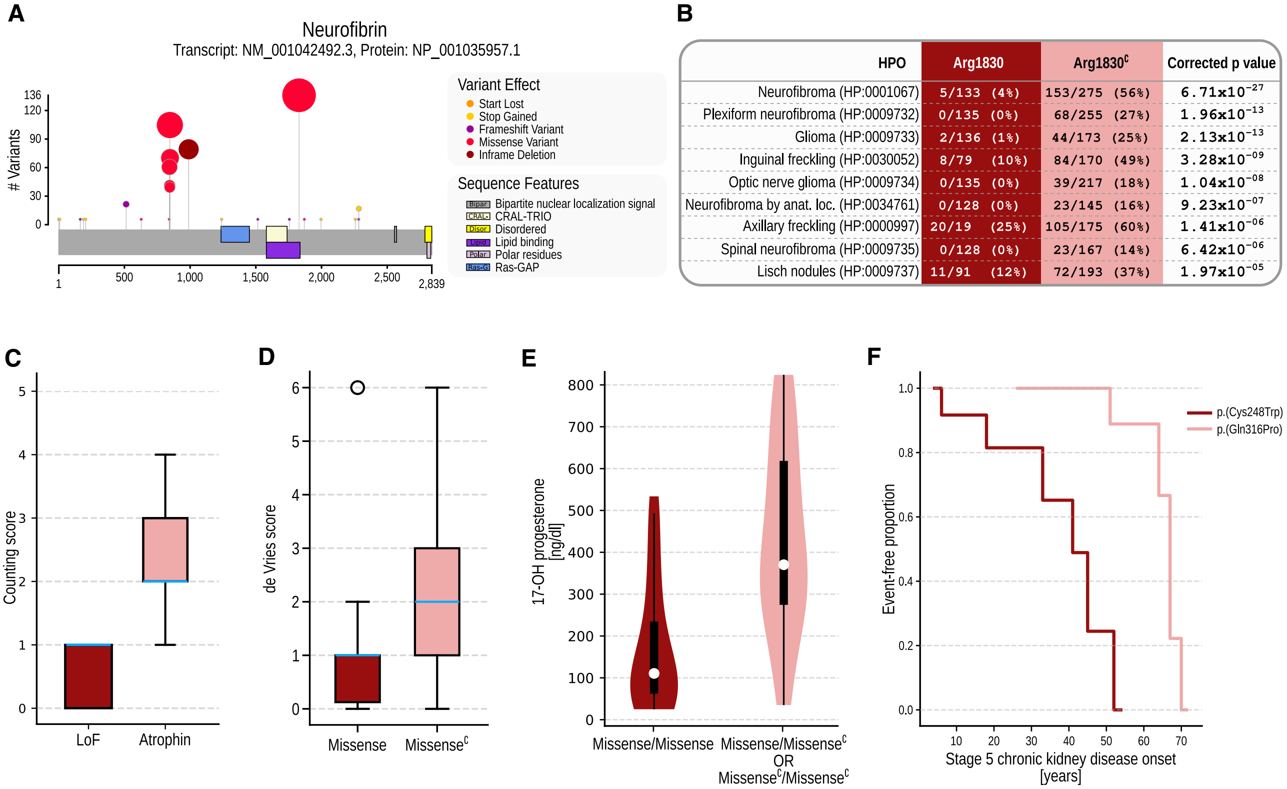

Figure 5. Excerpted results from five example analyses

(A) Visualization. GPSEA generates a cartoon showing the location and frequency of variants in protein sequences. The following panels show examples of statistically significant GPCs identified by GPSEA.

(B) Categorical analysis. Several phenotypic abnormalities (HPO terms) such as neurofibromas, optic nerve glioma, and Lisch nodules are significantly less frequent in individuals with neurofibromatosis type 1 due to variants located at the arginine residue at position 1,830 of neurofibromin isoform 1 than in those with different mutations (FET, IF-HPO, Benjamini-Hochberg correction).

(C) Severity score. A boxplot with counts of abnormalities in five organ systems in the individuals with mutations in *RERE* showing the association of the mutations in the Atrophin domain with abnormalities in multiple organ systems[27] (Mann-Whitney U test, $p = 1.44 \times 10^{-3}$). The boxes represent the Q1–Q3 range, and the whiskers extend to the farthest score lying within 1.5× the interquartile range. The blue line denotes the median score.

(D) de Vries score. Boxplots representing the association of the de Vries phenotype score[13] and missense variants in *CHD8* (Mann-Whitney U test, $p = 8.99 \times 10^{-4}$).

(E) Continuous phenotypes. Association of *CYP21A2* genotype (homozygous missense vs. other) with concentration of 17-OH progesterone (*t* test, $p = 7.91 \times 10^{-6}$).

(F) Survival analysis. Comparison of the onset of *Stage 5 chronic kidney disease* (HP:0003774) in individuals with *UMOD* mutations showing a significantly earlier onset of the disease in the individuals with NM_003361.4:c.744C>G; p.(Cys248Trp) than in those with NM_003361.4:c.947A>C; p.(Gln316Pro) (log-rank test, $p = 4.1 \times 10^{-4}$). Missense^{c}, set complement of "missense," i.e., any mutation that is *not* missense; LoF, loss of function.

Alternatively, users can choose to test specific HPO terms if there is a prior hypothesis or to test all terms. Standard MTC is applied to the tests performed following IF-HPO. By default, GPSEA applies the BH method[34] (ten other standard MTC approaches are available).

Figures 5A and 5B show an example cartoon generated for a cohort of individuals with *NF1* variants and results of a categorical analysis to test for associations of variants at residue Arg1830 (GenBank: NP_001035957.1) in individuals diagnosed with Neurofibromatosis, type 1 (MIM: 162200). Twelve HPO terms were found to have a significantly lower or higher frequency in individuals with variants at this position as compared to other variants in the *NF1* (for additional results for *NF1*, see Figure S42; for a summary of all significant categorical test results, see Table S2).

Phenotype scores

Phenotype scores have been developed for some diseases to provide a semi-objective assessment of disease severity. Many such scores count the total number of observed phenotypic features from a list of stipulated terms. Other scores involve more complicated systems that use Boolean logic or thresholding. An example of the first score is provided in the analysis of Atrophin domain variants in *RERE*, which were previously found to be significantly associated with higher scores defined by the counts of structural defects of the brain, eye, heart, kidney, and sensorineural hearing loss.[27] GPSEA provides Counting scorer, which allows users to indicate relevant HPO terms. The hierarchical structure of the HPO is used to count annotations to the term itself or any of its descendents; for

each of the terms, a count of 1 is given if one or more such annotations were found; otherwise, a count of zero is assigned for the term. The phenotype score thus ranges from 0 if no relevant abnormalities were recorded to the total count of specified HPO terms if an abnormality was found in all items. For instance, one of the terms was *Abnormal brain morphology* (HP:0012443). Therefore, one point would be given if the individual was annotated to any of the descendant terms, for instance, *Agenesis of corpus callosum* (HP:0001274). There was a significantly higher severity score for variants located in the Atrophin domain of *RERE* (Figures 5C and S50).

Other scores have been developed with more involved rules. For instance, the de Vries score was developed as a relatively simple phenotypic severity score for individuals with intellectual disability in which points are given for (severity of) intellectual disability, growth abnormalities (prenatal and postnatal), facial dysmorphisms, non-facial dysmorphisms, and other congenital anomalies.[13] We developed a modified version of this score that uses the structure of the HPO to "roll up" specific terms. Using this, we identified a significantly lower score (corresponding to milder clinical manifestations) in individuals with *CHD8* (MIM: 610528) missense variants than those with other *CHD8* variants, similar to the original application of the score to *CHD8* (Figure 5D).[13] We also applied the score to other cohorts; a similar significant association of missense variants with lower scores was identified for *CTCF* (MIM: 604167) (Figure S15; for a summary of all phenotype score results, see Table S3).

Numerical values

HPO terms are categorical and are not designed to capture continuous (numerical) values. Instead, the GA4GH Phenopacket Schema has a measurement element that can be used to represent the results of laboratory tests for analytes such as enzyme activity or metabolite concentrations. GPSEA can test the association of numerical data, such as metabolite levels or enzyme activities, with genotype classes. For example, in Adrenal hyperplasia, congenital, due to 21-hydroxylase deficiency (MIM: 201910), it is assumed that the mildest mutation determines the phenotype in compound heterozygotes and that missense variants in *CYP21A2* (MIM: 613815) have a less severe effect on enzyme activity than do other variants such as truncation or ablation variants.[35] Using GPSEA, we applied a *t* test and observed significantly lower 17-OH-progesterone levels (which are known to increase with reduced 21-hydroxylase activity) in the individuals with two missense alleles (Figures 5E and S16; for a summary of all *t* test results, see Table S4).

Survival analysis

GPSEA can perform survival analysis to assess associations between genotype classes and mortality, disease onset, or onset of a specific phenotypic abnormality such as *Stage 5 chronic kidney disease* (HP:0003774). The data are plotted as a Kaplan-Meier curve, and a log-rank test is applied to assess statistical significance (Figures 5F and S83). This requires that the phenopackets have information about the ages of onset or mortality; because this information was not available in most of the publications curated for this project, survival analysis was performed only for a subset of cohorts. The analysis leverages the ontological structure of the HPO to roll up annotation from descendant terms, similar to the procedure for categorical analysis. If we are testing for onset of *Seizure* (HP:0001250), and an individual was noted to have both *Tonic seizure* (HP:0032792) and *Generalized myoclonic seizure* (HP:0002123), the youngest age of onset for the latter two terms is chosen (for a summary of all survival analysis results, see Tables S5–S7).

Analysis by disease diagnosis

GPSEA also allows users to search for HPO terms that are different between two diseases. For instance, 8/25 (32%) individuals with Kabuki syndrome 1 (MIM: 147920) displayed *Feeding difficulties* (HP:0011968) compared to 55/63 (87%) individuals with Kabuki syndrome 2 (MIM: 300867) ($p = 2.1 \times 10^{-5}$, FET, IF-HPO, BH correction). A total of 16 significant findings were observed (Figure S28; for a summary of all disease analysis results, see Table S8).

Analysis of sex differences

A categorical analysis can be performed of the association between phenotypic features and sex (male or female). Tests were performed in 44 cohorts, and one significant difference was identified in the cohort for Kabuki syndrome 2, in which 14/18 (78%) males were annotated to the HPO term *Intellectual disability, severe* (HP:0010864), compared to only 7/25 (28%) females ($p = 7.77 \times 10^{-3}$; FET, BH) (Table S9).

GPCs are common in Mendelian disease

We analyzed 85 cohorts with 6,179 individuals (median 49 per cohort, range: 16–462) with 122 Mendelian diseases. Each individual was encoded as a phenopacket with information about the disease diagnosis, phenotypic abnormalities (HPO terms), and, where available, age of onset of the disease and individual features, age of death, and in some cases numerical laboratory test results. GPSEA analysis was applied to each of the cohorts. Existing knowledge about GPCs related to the gene or disease of interest was sought in PubMed (see material and methods) and, if possible, an analysis was performed in GPSEA to reproduce a similar result using the cohorts available. Alternatively or additionally, GPSEA visualizations were consulted to generate hypotheses about testable GPCs for common variant categories (e.g., missense and nonsense), common variants, exons, protein domains, or regions. If relevant information was available about onset or mortality, survival analysis was performed. In some cases, phenotype severity scores were applied or numerical analyses were performed. A total of 253 significant correlations were identified. We did not identify even a single publication for which the data and analysis script were made available in a way that would allow the original analysis to be replicated. Additionally, we attempted to curate data from all available

Table 1. Summary of tests performed according to type of test

Statistical procedure	Cohorts tested	Tests performed	Significant tests
Categorical analysis	78	6,736	217
t test	2	3	3
HPO onset	4	6	3
Disease onset	10	11	6
Mortality	3	3	1
Phenotype scores	6	9	7
Disease diagnosis	8	266	15
Sex differences	44	1,979	1
Total	85[a]	9,013	253

The table provides a summary of the results from the 85 cohorts tested, arranged according to the types of statistical tests offered by GPSEA. Categorical analysis: association of genotypes with phenotypes by a Fisher exact test. *t* test: test of means of continuous values by Student's *t* test. HPO onset: log-rank test for association of genotypes with age of onset of a phenotypic abnormality represented by an HPO term. Disease onset: log-rank test for association of genotypes with age of onset of a disease. Mortality: log-rank test for association of genotypes with age of death. Phenotype scores: Mann-Whitney U test for association of genotypes with magnitude of a phenotype severity score. Multiple testing correction was applied to the categorical tests (Benjamini-Hochberg method), following the independent filtering procedure (IF-HPO). Disease diagnosis: comparison of two or more diseases associated with the variants in the same gene. Sex differences: comparison of frequencies of phenotypic features in a disease between males and females. No multiple testing correction was applied to the remaining tests, which were considered to represent distinct hypotheses (detailed results are shown in Table S10).
[a]Multiple statistical procedure types were performed for some cohorts.

publications for each gene or disease being analyzed and so had different cohorts and a different methodology. Nevertheless, we assessed whether results are similar to previously published ones. Some of our cohorts involved comparison of diseases with well-known phenotypic differences; for instance, we compared Spastic paraplegia 78, autosomal recessive (MIM: 617225) and Kufor-Rakeb syndrome (MIM: 606693), both of which are caused by variants in *ATP13A2* (MIM: 610513), and showed a significantly higher frequency of *Parkinsonism* (HP:0001300) and *Bradykinesia* (HP:0002067) in the individuals with Kufor-Rakeb syndrome (Figure S8). Although statistical tests are rarely conducted to characterize allelic diseases in this way, we regard such differences as well known and record them as previously published in the literature for the purposes of Table 1. Other differences, such as a higher prevalence of *Osteoarthritis* (HP:0002758) in Loeys-Dietz syndrome 3 in individuals with the missense variant c.859C>T (GenBank: NM_005902.4) (p.Arg287Trp) (19/19) compared to individuals with other variants (7/19; 37%; $p = 3.7 \times 10^{-5}$) could not be identified in previous literature. Significant GPCs were identified for 48 cohorts. We identified previously published GPCs for 29 of these cohorts, many of which overlapped with our findings (references and detailed analysis of which are presented in Figures S1–S88). Seventy-one significant findings in the remaining 19 cohorts represent candidate GPCs that should be validated by independent studies on validation cohorts (Table 1; references for previously published findings are available in Figures S1–S88).

Distribution of phenotypic features with significant GPCs

We analyzed the distribution of HPO terms for which significant GPCs were identified by identifying the top-level term (direct child of *Phenotypic abnormality*). The distribution of terms was significantly different from what one would expect based on the counts of all terms in the *Phenotypic abnormality* subhierarchy of the HPO (exact multinomial test, $p = 3.17 \times 10^{-36}$). The largest differences were observed for *Abnormality of the nervous system* (HP:0000707; expected 10.7%, observed 23.0%), *Abnormality of metabolism/homeostasis* (HP:0001939; expected 9.5%, observed 1.1%), and *Neoplasm* (HP:0002664; expected 2.7%, observed 9.2%). This raises the possibility that phenotypic features in different organ systems may have a differential tendency to display GPCs, although our observation may also be the result of an ascertainment or other bias (Table S11).

Discussion

Precision genomic medicine is an emerging medical discipline that aims to apply genomic information for prediction, prevention for early diagnosis, or tailored treatment in order to improve clinical care. Although precision approaches have been applied successfully to some Mendelian diseases such as Cystic fibrosis (MIM: 219700),[36] our understanding of disease subtypes and GPCs is limited for the vast majority of the roughly 7,000 characterized rare Mendelian diseases. Understanding GPCs can contribute toward understanding disease pathophysiology and stratified clinical management. A barrier has been the lack of standardized data exchange and analysis schemas, which means that it is difficult to combine data from multiple sources and that scripts or program code need to be created anew for each project. The GA4GH Phenopacket Schema was released in 2022 and approved by the International Standards

Organization (ISO 4454:2022) as a standard for sharing clinical and genomic information about an individual. Each phenopacket is a computational representation of the clinical trajectory of one individual and can contain data about phenotypic descriptions, numerical measurements, genetic information, diagnoses, and treatments. A phenopacket can be used for data exchange and as a computational model for clinical decision support systems scoped on individuals and their families, such as Exomiser,[37] LIRICAL,[38] and Emedgene, as well as for the algorithms that facilitate classification, stratification, or GPC discovery in cohorts of individuals, such as GPSEA.[22] Therefore, when phenopackets serve as a unifying standard across projects, locations, and registries, they enable machine readability and reusability for multiple analyses while offering precise, ontology-based semantics and adherence to the findable, accessible, interoperable, and reusable (FAIR) data principles. The GA4GH Phenopacket Schema thus enables software such as GPSEA to be used for any relevant dataset that is available as or can be transformed into phenopackets.

GPCs can be of utility for clinical management decisions or translational research. GPSEA is not designed for use in clinical care but rather provides a framework that is helpful for characterizing GPCs in cohorts of individuals, and it is our hope that it will contribute to the discovery of novel GPCs. In Mendelian disease, GPCs display a spectrum of association strength. With some genes associated with Mendelian disease, different mutations lead deterministically to distinct diseases. For instance, different germline *FGFR3* (MIM: 134934) variants cause Achondroplasia (MIM: 100800), Hypochondroplasia (MIM: 146000), and other disorders; timely diagnosis of *FGFR3*-related skeletal dysplasia is essential for timely management of complications and genetic counseling.[39] Specific *NF1* variants are associated with mild clinical manifestations of neurofibromatosis type 1; for instance, variants affecting Arg1809 tend to show café-au-lait macules and Noonan-like dysmorphic features but do not have neurofibromas and some other typical neurofibromatosis features.[40] In other cases, the degree of association of a GPC is weaker, so that individuals with a certain variant or category of variant tend to have a lower or higher frequency of a feature. For instance, premature termination codon variants in *FBN1* (MIM: 134797) are associated with a higher risk of aortic dissection in Marfan syndrome (MIM: 154700), but individuals with other categories of variants may also experience aortic dissection.[41] Understanding GPCs in these and other Mendelian diseases may help guide clinical management in some cases. In a few cases, correlations of specific variants with clinical data are included in clinical guidelines, as is the case for *CFTR* (MIM: 602421) variants.[42] GPCs have also been used as the starting point for experimental work to understand disease biology and gene function; for instance, a *de novo* single-base substitution within the *LMNA* (MIM: 150330) exon 11 (c.1824C>T [GenBank: NM_170707.4] [p.(=)]) activates a cryptic splice site, leading to an in-frame deletion of 50 amino acids near the C terminus of prelamin A; this mutant form of lamin A acts in a dominant fashion to induce a whole variety of abnormalities in nuclear processes, which eventually lead to cellular and organismal decline and cause Hutchinson-Gilford progeria (MIM: 176670). This pathomechanism is distinct from that observed in other laminopathies such as Cardiomyopathy, dilated, 1A (MIM: 115200), Emery-Dreifuss muscular dystrophy 2, autosomal dominant (MIM: 181350), and Mandibuloacral dysplasia (MIM: 248370).[43] The identification of GPCs can thus help to formulate well-targeted hypotheses for molecular research.

An area where genotype-phenotype stratification approach could provide immediate value is in prenatal genomics. Increasingly, diagnostic genomic sequencing is performed during pregnancy following an abnormal fetal phenotype, often with results available between 16 and 18 weeks gestation. At this stage, decisions about clinical management can be extremely difficult and time sensitive. Having a clearer, evidence-based understanding of likely phenotypic outcomes based on genotype could support more informed and personalized counseling for families and help them to weigh risks and make decisions aligned with their values and tolerance for uncertainty.

As more and more data in human genetics becomes available with HPO annotations, new challenges arise for analysis because of the ontological structure of the HPO. Performing GPC analysis with HPO annotations raises challenges for analysis because of the ontological structure of the HPO. Analysis software needs to roll up annotations; for instance, if an individual is annotated to *Nuclear cataract* (HP:0100018), it is always true that the individual also has the manifestation described by the parent term *Zonular cataract* (HP:0010920) and the grandparent term *Cataract* (HP:0000518), and so forth (this is termed the "true path rule"). However, HPO annotation is performed to the most specific level, and typical statistical software used to work with data frames is not able to perform the rolling up that is needed for correct analysis. Another challenge when using ontologies for analysis relates to the redundancies inherent in the hierarchical structure.[44] GPSEA addresses both challenges by preparing data for analysis using the true path rule and minimizing redundancy and multiple-testing burden by the IF-HPO procedure.

Many publications on GPC analysis either do not provide raw data or provide a summary of data in the supplement as an Excel file or related format. Rarely is analysis code provided to reproduce the results, and since the formats used in the supplemental files are diverse, it requires a substantial amount of work to prepare them for statistical analysis. The approach we have presented here makes the investigation of GPCs FAIR. The entirety of the data used for the analysis is freely available in Phenopacket Store,[25] and all code used for analysis is available in the

GitHub repository (one notebook is provided for each cohort). An additional advantage of this approach is that results for new cohorts can be assessed in comparison to a body of previous results using the same software.

For this project, we used data derived from published cohorts for our analysis. The cohorts we present here were all derived from published case and cohort reports. In general, published cohorts do not contain comprehensive clinical information but instead present features deemed most relevant or important by the authors. Furthermore, phenotype is not solely determined by genetic factors; environmental and lifestyle influences can interact with allelic variability and modifier genes to further shape phenotype. For this reason, and also because of potential publication biases that may lead to an overestimation of clinical severity,[45] the results about specific GPCs presented here should be regarded as hypotheses that will require confirmation in independent studies.

Allelic variability is only one of many factors that determine phenotype. However, phenotypic severity or penetrance can be influenced by the genotype at another locus, which is referred to as a modifier gene.[46] Indeed, in hereditary breast cancer, polygenic risk scores (PRSs) for ovarian cancer are associated with penetrance of ovarian cancer in individuals harboring mutations in *BRCA1* or *BRCA2* (MIM: 113705 and 600185).[47] GPSEA could be easily extended to evaluate the effects of PRSs or variants in modifier genes, but the main challenge will be in the collection of comprehensive clinical and genomic data. As a general rule, the ability to identify GPCs will depend in general on the presence of true biomedical differences, on the quality and comprehensiveness of reporting of clinical data, and on statistical power related to the size of the cohort. Prospectively capturing clinical data using a common data model could be beneficial.[48]

GPSEA serves as a foundational tool, enabling correlation studies to be conducted at any scale, within any setup, and for any hypothesis. For instance, we envision that GPSEA can be used for comprehensive research databases with more balanced datasets, whether or not the raw data can be shared publicly. Wide community adoption of the GA4GH Phenopacket Schema and application of consistent practices for recording and reporting phenotypic features in publications and databases would accelerate characterization of GPCs across the Mendeliome and thereby contribute to our knowledge of the natural history of rare diseases.

Data and code availability

- No original data were generated for this analysis. GPSEA is available at https://github.com/P2GX/gpsea under an MIT license. Documentation and a tutorial are provided at https://p2gx.github.io/gpsea/stable/.
- The GPSEA case studies (gpsea-cs) repository provides one Jupyter notebook for each of the cohorts analyzed in this work and is available at https://github.com/P2GX/gpsea-cs. This repository additionally contains code we used to generate the supplemental figures and tables that is not needed by new users of GPSEA.

Acknowledgments

This work was supported by grants from the National Human Genome Research Institute (A Phenomics-First Resource for Interpretation of Variants, 5RM1HG010860 and The Human Phenotype Ontology: Accelerating Computational Integration of Clinical Data for Genomics, 5U24HG011449; J.X.C. and A.J.M.D. were supported by 1R35HG011297). P.N.R. was supported by a Professorship of the Alexander von Humboldt Foundation. A.K. and O.V. were supported by grant NU23-05-00097 issued by the Czech Health Research Council, Ministry of Health of the Czech Republic.

Author contributions

P.N.R. and D.D. conceived and designed the project and methodology; L.R. and D.D. wrote the Python code with contributions from P.N.R., J.R., and F.R.; P.N.R. developed the statistical modeling with input from M.I.L.; the cohorts were analyzed by L.R., A.S.L.G., V.B., A.C.-O., P.C., L.C., J.X.C., E.C., A.J.M.D., B.B.A.d.V., M.H.D., T.F., P.G., P.H., A.K., M.S.L., A.M., A.J.M., J.R., C.S., T.S., O.V., D.Z., and P.N.R. using the GPSEA software; M.A.H., T.G., J.O.B.J., C.J.M., M.C.M.-T., S.T., and D.S. advised about the use and creation of phenopackets; D.D. and P.N.R. wrote the manuscript; and all authors reviewed and approved the final version.

Declaration of interests

The authors declare no competing interests.

Web resources

GenBank, https://www.ncbi.nlm.nih.gov/genbank/
Human Phenotype Ontology (HPO), https://hpo.jax.org/
OMIM, https://www.omim.org/
Phenopacket Store, https://github.com/monarch-initiative/phenopacket-store

Supplemental information

Supplemental information can be found online at https://doi.org/10.1016/j.ajhg.2025.12.001.

Received: April 8, 2025
Accepted: December 1, 2025
Published: December 23, 2025

References

1. Ries, M., and Gal, A. (2006). Genotype–phenotype correlation in Fabry disease. In Fabry Disease: Perspectives from 5 Years of FOS, A. Mehta, M. Beck, and G. Sunder-Plassmann, eds. (Oxford PharmaGenesis).
2. Bettegowda, C., Upadhayaya, M., Evans, D.G., Kim, A., Mathios, D., Hanemann, C.O.; and REiNS International Collaboration

(2021). Genotype-phenotype correlations in neurofibromatosis and their potential clinical use. Neurology *97*, S91–S98. https://doi.org/10.1212/WNL.0000000000012436.
3. MacRae, C.A., and Seidman, C.E. (2017). Closing the Genotype-Phenotype Loop for Precision Medicine. Circulation *136*, 1492–1494. https://doi.org/10.1161/CIRCULATIONAHA.117.030831.
4. Robinson, P.N., Köhler, S., Bauer, S., Seelow, D., Horn, D., and Mundlos, S. (2008). The Human Phenotype Ontology: a tool for annotating and analyzing human hereditary disease. Am. J. Hum. Genet. *83*, 610–615. https://doi.org/10.1016/j.ajhg.2008.09.017.
5. Köhler, S., Vasilevsky, N.A., Engelstad, M., Foster, E., McMurry, J., Aymé, S., Baynam, G., Bello, S.M., Boerkoel, C.F., Boycott, K.M., et al. (2017). The Human Phenotype Ontology in 2017. Nucleic Acids Res. *45*, D865–D876. https://doi.org/10.1093/nar/gkw1039.
6. Köhler, S., Doelken, S.C., Mungall, C.J., Bauer, S., Firth, H.V., Bailleul-Forestier, I., Black, G.C.M., Brown, D.L., Brudno, M., Campbell, J., et al. (2014). The Human Phenotype Ontology project: linking molecular biology and disease through phenotype data. Nucleic Acids Res. *42*, D966–D974. https://doi.org/10.1093/nar/gkt1026.
7. Pehlivan, D., Bengtsson, J.D., Bajikar, S.S., Grochowski, C.M., Lun, M.Y., Gandhi, M., Jolly, A., Trostle, A.J., Harris, H.K., Suter, B., et al. (2024). Structural variant allelic heterogeneity in MECP2 duplication syndrome provides insight into clinical severity and variability of disease expression. Genome Med. *16*, 146. https://doi.org/10.1186/s13073-024-01411-7.
8. Alecu, J.E., Tam, A., Richter, S., Quiroz, V., Schierbaum, L., Saffari, A., and Ebrahimi-Fakhari, D. (2025). Quantitative natural history modeling of HPDL-related disease based on cross-sectional data reveals genotype-phenotype correlations. Genet. Med. *27*, 101349. https://doi.org/10.1016/j.gim.2024.101349.
9. Dardas, Z., Fatih, J.M., Jolly, A., Dawood, M., Du, H., Grochowski, C.M., Jones, E.G., Jhangiani, S.N., Wehrens, X.H.T., Liu, P., et al. (2024). NODAL variants are associated with a continuum of laterality defects from simple D-transposition of the great arteries to heterotaxy. Genome Med. *16*, 53. https://doi.org/10.1186/s13073-024-01312-9.
10. Bosch, E., Popp, B., Güse, E., Skinner, C., van der Sluijs, P.J., Maystadt, I., Pinto, A.M., Renieri, A., Bruno, L.P., Granata, S., et al. (2023). Elucidating the clinical and molecular spectrum of SMARCC2-associated NDD in a cohort of 65 affected individuals. Genet. Med. *25*, 100950. https://doi.org/10.1016/j.gim.2023.100950.
11. Calame, D.G., Guo, T., Wang, C., Garrett, L., Jolly, A., Dawood, M., Kurolap, A., Henig, N.Z., Fatih, J.M., Herman, I., et al. (2023). Monoallelic variation in DHX9, the gene encoding the DExH-box helicase DHX9, underlies neurodevelopment disorders and Charcot-Marie-Tooth disease. Am. J. Hum. Genet. *110*, 1394–1413. https://doi.org/10.1016/j.ajhg.2023.06.013.
12. Guatibonza Moreno, P., Pardo, L.M., Pereira, C., Schroeder, S., Vagiri, D., Almeida, L.S., Juaristi, C., Hosny, H., Loh, C.C.Y., Leubauer, A., et al. (2023). At a glance: the largest Niemann-Pick type C1 cohort with 602 patients diagnosed over 15 years. Eur. J. Hum. Genet. *31*, 1108–1116. https://doi.org/10.1038/s41431-023-01408-7.
13. Dingemans, A.J.M., Truijen, K.M.G., van de Ven, S., Bernier, R., Bongers, E.M.H.F., Bouman, A., de Graaff-Herder, L., Eichler, E.E., Gerkes, E.H., De Geus, C.M., et al. (2022). The phenotypic spectrum and genotype-phenotype correlations in 106 patients with variants in major autism gene CHD8. Transl. Psychiatry *12*, 421. https://doi.org/10.1038/s41398-022-02189-1.
14. Crawford, K., Xian, J., Helbig, K.L., Galer, P.D., Parthasarathy, S., Lewis-Smith, D., Kaufman, M.C., Fitch, E., Ganesan, S., O'Brien, M., et al. (2021). Computational analysis of 10,860 phenotypic annotations in individuals with SCN2A-related disorders. Genet. Med. *23*, 1263–1272. https://doi.org/10.1038/s41436-021-01120-1.
15. van der Spek, J., den Hoed, J., Snijders Blok, L., Dingemans, A.J.M., Schijven, D., Nellaker, C., Venselaar, H., Astuti, G.D.N., Barakat, T.S., Bebin, E.M., et al. (2022). Inherited variants in CHD3 show variable expressivity in Snijders Blok-Campeau syndrome. Genet. Med. *24*, 1283–1296. https://doi.org/10.1016/j.gim.2022.02.014.
16. Zhang, C., Jolly, A., Shayota, B.J., Mazzeu, J.F., Du, H., Dawood, M., Soper, P.C., Ramalho de Lima, A., Ferreira, B.M., Coban-Akdemir, Z., et al. (2022). Novel pathogenic variants and quantitative phenotypic analyses of Robinow syndrome: WNT signaling perturbation and phenotypic variability. HGG Adv. *3*, 100074. https://doi.org/10.1016/j.xhgg.2021.100074.
17. Hebebrand, M., Hüffmeier, U., Trollmann, R., Hehr, U., Uebe, S., Ekici, A.B., Kraus, C., Krumbiegel, M., Reis, A., Thiel, C.T., and Popp, B. (2019). The mutational and phenotypic spectrum of TUBA1A-associated tubulinopathy. Orphanet J. Rare Dis. *14*, 38. https://doi.org/10.1186/s13023-019-1020-x.
18. Casanova, E.L., Gerstner, Z., Sharp, J.L., Casanova, M.F., and Feltus, F.A. (2018). Widespread genotype-phenotype correlations in intellectual disability. Front. Psychiatry *9*, 535. https://doi.org/10.3389/fpsyt.2018.00535.
19. van der Sluijs, P.J., Jansen, S., Vergano, S.A., Adachi-Fukuda, M., Alanay, Y., AlKindy, A., Baban, A., Bayat, A., Beck-Wödl, S., Berry, K., et al. (2019). The ARID1B spectrum in 143 patients: from nonsyndromic intellectual disability to Coffin-Siris syndrome. Genet. Med. *21*, 1295–1307. https://doi.org/10.1038/s41436-018-0330-z.
20. Chiorean, A., Farncombe, K.M., Delong, S., Andric, V., Ansar, S., Chan, C., Clark, K., Danos, A.M., Gao, Y., Giles, R.H., et al. (2022). Large scale genotype- and phenotype-driven machine learning in Von Hippel-Lindau disease. Hum. Mutat. *43*, 1268–1285. https://doi.org/10.1002/humu.24392.
21. Chiu, T.L.-H., Leung, D., Chan, K.-W., Yeung, H.M., Wong, C.-Y., Mao, H., He, J., Vignesh, P., Liang, W., Liew, W.K., et al. (2021). Phenomic analysis of chronic granulomatous disease reveals more severe integumentary infections in X-linked compared with autosomal recessive chronic granulomatous disease. Front. Immunol. *12*, 803763. https://doi.org/10.3389/fimmu.2021.803763.
22. Jacobsen, J.O.B., Baudis, M., Baynam, G.S., Beckmann, J.S., Beltran, S., Buske, O.J., Callahan, T.J., Chute, C.G., Courtot, M., Danis, D., et al. (2022). The GA4GH Phenopacket schema defines a computable representation of clinical data. Nat. Biotechnol. *40*, 817–820. https://doi.org/10.1038/s41587-022-01357-4.
23. Danis, D., Jacobsen, J.O.B., Wagner, A.H., Groza, T., Beckwith, M.A., Rekerle, L., Carmody, L.C., Reese, J., Hegde, H., Ladewig, M.S., et al. (2023). Phenopacket-tools: Building and validating GA4GH phenopackets. PLoS One *18*, e0285433. https://doi.org/10.1371/journal.pone.0285433.

24. Ladewig, M.S., Jacobsen, J.O.B., Wagner, A.H., Danis, D., El Kassaby, B., Gargano, M., Groza, T., Baudis, M., Steinhaus, R., Seelow, D., et al. (2023). GA4GH phenopackets: A practical introduction. Adv. Genet. *4*, 2200016. https://doi.org/10.1002/ggn2.202200016.
25. Danis, D., Bamshad, M.J., Bridges, Y., Caballero-Oteyza, A., Cacheiro, P., Carmody, L.C., Chimirri, L., Chong, J.X., Coleman, B., Dalgleish, R., et al. (2025). A corpus of GA4GH phenopackets: Case-level phenotyping for genomic diagnostics and discovery. HGG Adv. *6*, 100371. https://doi.org/10.1016/j.xhgg.2024.100371.
26. Bourgon, R., Gentleman, R., and Huber, W. (2010). Independent filtering increases detection power for high-throughput experiments. Proc. Natl. Acad. Sci. USA *107*, 9546–9551. https://doi.org/10.1073/pnas.0914005107.
27. Jordan, V.K., Fregeau, B., Ge, X., Giordano, J., Wapner, R.J., Balci, T.B., Carter, M.T., Bernat, J.A., Moccia, A.N., Srivastava, A., et al. (2018). Genotype-phenotype correlations in individuals with pathogenic RERE variants. Hum. Mutat. *39*, 666–675. https://doi.org/10.1002/humu.23400.
28. de Vries, B.B., White, S.M., Knight, S.J., Regan, R., Homfray, T., Young, I.D., Super, M., McKeown, C., Splitt, M., Quarrell, O.W., et al. (2001). Clinical studies on submicroscopic subtelomeric rearrangements: a checklist. J. Med. Genet. *38*, 145–150. https://doi.org/10.1136/jmg.38.3.145.
29. Bland, J.M., and Altman, D.G. (2004). The logrank test. BMJ *328*, 1073. https://doi.org/10.1136/bmj.328.7447.1073.
30. UniProt Consortium (2025). UniProt: The universal protein knowledgebase in 2025. Nucleic Acids Res. *53*, D609–D617. https://doi.org/10.1093/nar/gkae1010.
31. Amberger, J.S., Bocchini, C.A., Scott, A.F., and Hamosh, A. (2019). OMIM.org: leveraging knowledge across phenotype-gene relationships. Nucleic Acids Res. *47*, D1038–D1043. https://doi.org/10.1093/nar/gky1151.
32. Gracia-Diaz, C., Zhou, Y., Yang, Q., Maroofian, R., Espana-Bonilla, P., Lee, C.-H., Zhang, S., Padilla, N., Fueyo, R., Waxman, E.A., et al. (2023). Gain and loss of function variants in EZH1 disrupt neurogenesis and cause dominant and recessive neurodevelopmental disorders. Nat. Commun. *14*, 4109. https://doi.org/10.1038/s41467-023-39645-5.
33. Alkan, C., Coe, B.P., and Eichler, E.E. (2011). Genome structural variation discovery and genotyping. Nat. Rev. Genet. *12*, 363–376. https://doi.org/10.1038/nrg2958.
34. Benjamini, Y. (2010). Discovering the false discovery rate: False Discovery Rate. J. R. Stat. Soc. Series B Stat. Methodol. *72*, 405–416. https://doi.org/10.1111/j.1467-9868.2010.00746.x.
35. Xu, C., Jia, W., Cheng, X., Ying, H., Chen, J., Xu, J., Guan, Q., Zhou, X., Zheng, D., Li, G., and Zhao, J. (2019). Genotype-phenotype correlation study and mutational and hormonal analysis in a Chinese cohort with 21-hydroxylase deficiency. Mol. Genet. Genomic Med. *7*, e671. https://doi.org/10.1002/mgg3.671.
36. Chang, E.H., and Zabner, J. (2015). Precision genomic medicine in cystic fibrosis. Clin. Transl. Sci. *8*, 606–610. https://doi.org/10.1111/cts.12292.
37. Vestito, L., Jacobsen, J.O.B., Walker, S., Cipriani, V., Harris, N.L., Haendel, M.A., Mungall, C.J., Robinson, P., and Smedley, D. (2024). Efficient reinterpretation of rare disease cases using Exomiser. NPJ Genom. Med. *9*, 65. https://doi.org/10.1038/s41525-024-00456-2.
38. Robinson, P.N., Ravanmehr, V., Jacobsen, J.O.B., Danis, D., Zhang, X.A., Carmody, L.C., Gargano, M.A., Thaxton, C.L., UNC Biocuration Core, and Karlebach, G., et al. (2020). Interpretable Clinical Genomics with a Likelihood Ratio Paradigm. Am. J. Hum. Genet. *107*, 403–417. https://doi.org/10.1016/j.ajhg.2020.06.021.
39. Kim, H.Y., and Ko, J.M. (2022). Clinical management and emerging therapies of FGFR3-related skeletal dysplasia in childhood. Ann. Pediatr. Endocrinol. Metab. *27*, 90–97. https://doi.org/10.6065/apem.2244114.057.
40. Rojnueangnit, K., Xie, J., Gomes, A., Sharp, A., Callens, T., Chen, Y., Liu, Y., Cochran, M., Abbott, M.-A., Atkin, J., et al. (2015). High incidence of Noonan syndrome features including short stature and pulmonic stenosis in patients carrying NF1 missense mutations affecting p.Arg1809: Genotype-phenotype correlation: Human mutation. Hum. Mutat. *36*, 1052–1063. https://doi.org/10.1002/humu.22832.
41. Arnaud, P., Milleron, O., Hanna, N., Ropers, J., Ould Ouali, N., Affoune, A., Langeois, M., Eliahou, L., Arnoult, F., Renard, P., et al. (2021). Clinical relevance of genotype-phenotype correlations beyond vascular events in a cohort study of 1500 Marfan syndrome patients with FBN1 pathogenic variants. Genet. Med. *23*, 1296–1304. https://doi.org/10.1038/s41436-021-01132-x.
42. Castellani, C., De Boeck, K., De Wachter, E., Sermet-Gaudelus, I., Simmonds, N.J., Southern, K.W.; and ECFS Diagnostic Network Working Group (2022). ECFS standards of care on CFTR-related disorders: Updated diagnostic criteria. J. Cyst. Fibros. *21*, 908–921. https://doi.org/10.1016/j.jcf.2022.09.011.
43. Gonzalo, S., Kreienkamp, R., and Askjaer, P. (2017). Hutchinson-Gilford Progeria Syndrome: A premature aging disease caused by LMNA gene mutations. Ageing Res. Rev. *33*, 18–29. https://doi.org/10.1016/j.arr.2016.06.007.
44. Grossmann, S., Bauer, S., Robinson, P.N., and Vingron, M. (2007). Improved detection of overrepresentation of Gene-Ontology annotations with parent child analysis. Bioinformatics *23*, 3024–3031. https://doi.org/10.1093/bioinformatics/btm440.
45. Nannenberg, E.A., van Rijsingen, I.A.W., van der Zwaag, P.A., van den Berg, M.P., van Tintelen, J.P., Tanck, M.W.T., Ackerman, M.J., Wilde, A.A.M., and Christiaans, I. (2018). Effect of ascertainment bias on estimates of patient mortality in inherited cardiac diseases. Circ. Genom. Precis. Med. *11*, e001797. https://doi.org/10.1161/CIRCGEN.117.001797.
46. Corvol, H., Blackman, S.M., Boëlle, P.-Y., Gallins, P.J., Pace, R.G., Stonebraker, J.R., Accurso, F.J., Clement, A., Collaco, J.M., Dang, H., et al. (2015). Genome-wide association meta-analysis identifies five modifier loci of lung disease severity in cystic fibrosis. Nat. Commun. *6*, 8382. https://doi.org/10.1038/ncomms9382.
47. Dareng, E.O., Tyrer, J.P., Barnes, D.R., Jones, M.R., Yang, X., Aben, K.K.H., Adank, M.A., Agata, S., Andrulis, I.L., Anton-Culver, H., et al. (2022). Polygenic risk modeling for prediction of epithelial ovarian cancer risk. Eur. J. Hum. Genet. *30*, 349–362. https://doi.org/10.1038/s41431-021-00987-7.
48. Graefe, A.S.L., Hübner, M.R., Rehburg, F., Sander, S., Klopfenstein, S.A.I., Alkarkoukly, S., Grönke, A., Weyersberg, A., Danis, D., Zschüntzsch, J., et al. (2025). An ontology-based rare disease common data model harmonising international registries, FHIR, and Phenopackets. Sci. Data *12*, 234. https://doi.org/10.1038/s41597-025-04558-z.

The American Journal of Human Genetics, Volume *113*

Supplemental information

GA4GH phenopacket-driven characterization of genotype-phenotype correlations in Mendelian disorders

Lauren Rekerle, Daniel Danis, Filip Rehburg, Adam S.L. Graefe, Viktor Bily, Andrés Caballero-Oteyza, Pilar Cacheiro, Leonardo Chimirri, Jessica X. Chong, Evan Connelly, Bert B.A. de Vries, Alexander J.M. Dingemans, Michael H. Duyzend, Tomas Freiberger, Petra Gehle, Tudor Groza, Peter Hansen, Julius O.B. Jacobsen, Adam Klocperk, Markus S. Ladewig, Michael I. Love, Allison J. Marcello, Alexander Mordhorst, Monica C. Munoz-Torres, Justin Reese, Catharina Schuetz, Damian Smedley, Timmy Strauss, Ondrej Vladyka, David Zocche, Sylvia Thun, Christopher J. Mungall, Melissa A. Haendel, and Peter N. Robinson

ABCB7

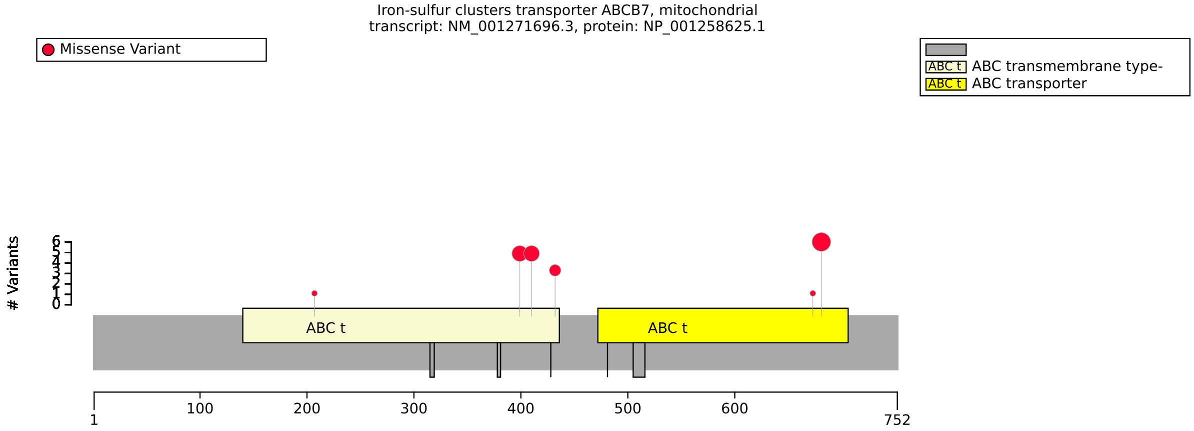

(a) Distribution of variants in *ABCB7*

Genotype (A)	Genotype (B)	total tests performed	significant results
ABC transmembrane type-1	Other region	12	0
p.Gly682Ser	Other variant	10	0

(b) Fisher Exact Test performed to compare HPO annotation frequency with respect to variants located in the ABC transmembrane type-1 region and p.Gly682Ser.

Figure S1: The cohort comprised 18 individuals (0 females, 18 males). A total of 52 HPO terms were used to annotate the cohort. Disease diagnosis: Anemia, sideroblastic, and spinocerebellar ataxia (OMIM:301310). No statistically significant results identified. A total of 18 unique variant alleles were found in *ABCB7* (transcript: `NM_001271696.3`, protein id: `NP_001258625.1`).

ACADM

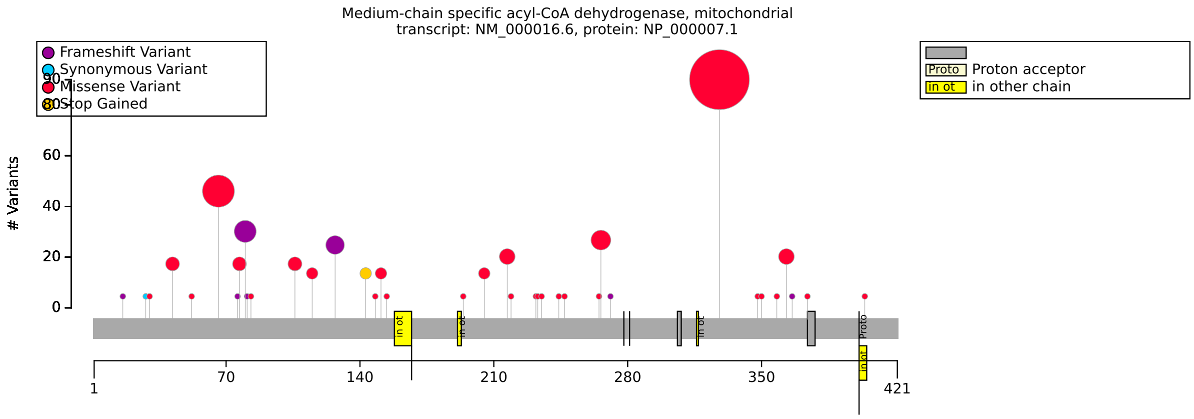

(a) Distribution of variants in *ACADM*

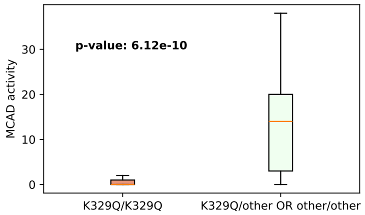

(b) Lys329Glu: t test for MCAD Activity (% normal; LOINC:74892-1): p=6.12×10^{-10}.

Description	Variable	Genotype (A)	Genotype (B)	p-value	ref
Value of MCAD Activity% [LOINC:74892-1]	LOINC:74892-1	K329Q/K329Q	K329Q/other OR other/other	6.12×10^{-10}	[1]

(c) t-test to compare K329Q/K329Q and K329Q/other OR other/other with respect to LOINC:74892-1. Mean MCAD activity for K329/K329: 0.52%, and for K329/other or other/other: 13.23%

Description	Variable	Genotype (A)	Genotype (B)	p-value	xrefs
Value of MCAD Activity% [LOINC:74892-1]	LOINC:74892-1	Y67H/Y67H OR Y67H/other	other/other	2.01×10^{-5}	[1]

(d) t-test to compare Y67H/Y67H OR Y67H/other and other/other with respect to LOINC:74892-1. Mean MCAD activity for Y67H/Y67H: 18.60%, and for Y67H/other or other/other: 7.68%

Figure S2: The cohort comprised 115 individuals (0 females, 0 males, 115 with unknown sex). The cohort had data about medium chain Acyl-CoA dehydrogenase (MCAD), expressed as percentage of normal. The variant c.985G>A (p.Lys329Glu) is known to be severe, and the variant c.199T>C (p.Tyr67His) is known to be mild [1]. Disease diagnosis: Acyl-CoA dehydrogenase, medium chain, deficiency of (OMIM:201450). No statistically significant results identified. A total of 47 unique variant alleles were found in *ACADM* (transcript: `NM_000016.6`, protein id: `NP_000007.1`).

ACBD6

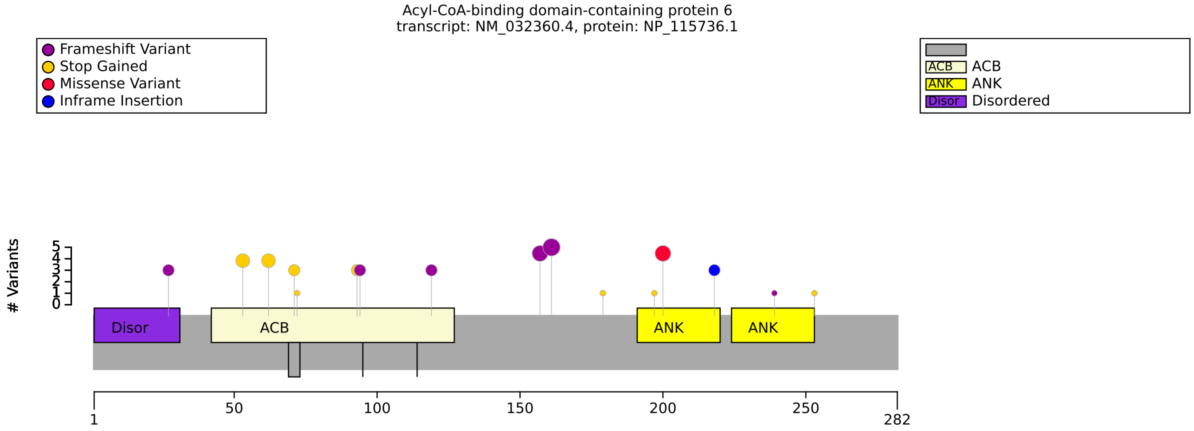

(a) Distribution of variants in *ACBD6*

Genotype (A)	Genotype (B)	total tests performed	significant results
missense/missense OR missense/other	other/other	99	0

(b) Fisher Exact Test performed to compare HPO annotation frequency with respect to missense/missense OR missense/other and other/other.

Genotype (A)	Genotype (B)	total tests performed	significant results
ACB/ACB OR ACB/other	other/other	99	0

(c) Fisher Exact Test performed to compare HPO annotation frequency with respect to ACB/ACB OR ACB/other and other/other.

Genotype (A)	Genotype (B)	total tests performed	significant results
FEMALE	MALE	99	0

(d) Fisher Exact Test performed to compare HPO annotation frequency with respect to FEMALE and MALE.

Figure S3: The cohort comprised 45 individuals (22 females, 23 males). A total of 79 HPO terms were used to annotate the cohort. Disease diagnosis: Neurodevelopmental disorder with progressive movement abnormalities (OMIM:620785). No statistically significant results identified. A total of 20 unique variant alleles were found in *ACBD6* (transcript: `NM_032360.4`, protein id: `NP_115736.1`).

AIRE

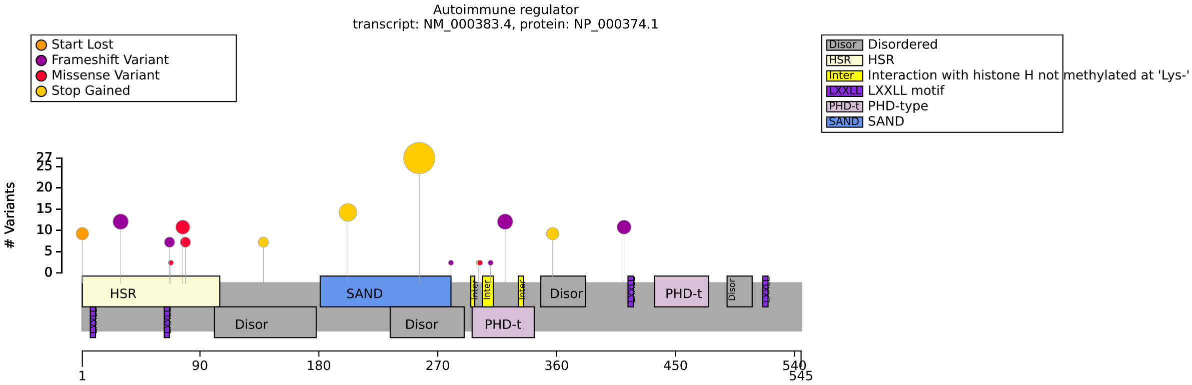

(a) Distribution of variants in *AIRE*

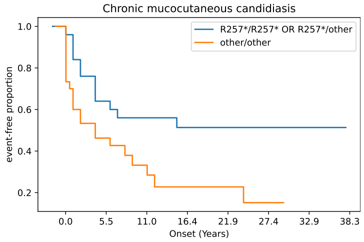

(b) log-rank p-value for Chronic mucocutaneous candidiasis (HP:0002728) and R357*/R357* or R357*/other versus other/other 0.0192

Genotype (A)	Genotype (B)	total tests performed	significant results
R257*/R257* OR R257*/other	other/other	18	0

(c) Fisher Exact Test performed to compare HPO annotation frequency with respect to R257*/R257* OR R257*/other and other/other.

Description	Variable	Genotype (A)	Genotype (B)	p-value	xrefs
Survival analysis: Chronic mucocutaneous candidiasis	Onset of HP:0002728	R257*/R257* OR R257*/other	other/other	0.019	[2]

(d) Onset of Chronic mucocutaneous candidiasis (HP:0002728) to compare R257*/R257* OR R257*/other and other/other with respect to Onset of HP:0002728.

Figure S4: The cohort comprised 58 individuals (31 females, 27 males). 2 of these individuals were reported to be deceased. A total of 47 HPO terms were used to annotate the cohort. Disease diagnosis: Autoimmune polyendocrinopathy syndrome , type I, with or without reversible metaphyseal dysplasia (OMIM:240300). A higher prevalence of chronic mucocutanous candidiasis with the variant Arg357Ter than with other variants was reported previously [2]. We did not identify a significant difference in prevalence A total of 18 unique variant alleles were found in *AIRE* (transcript: `NM_000383.4`, protein id: `NP_000374.1`).

ANKRD11

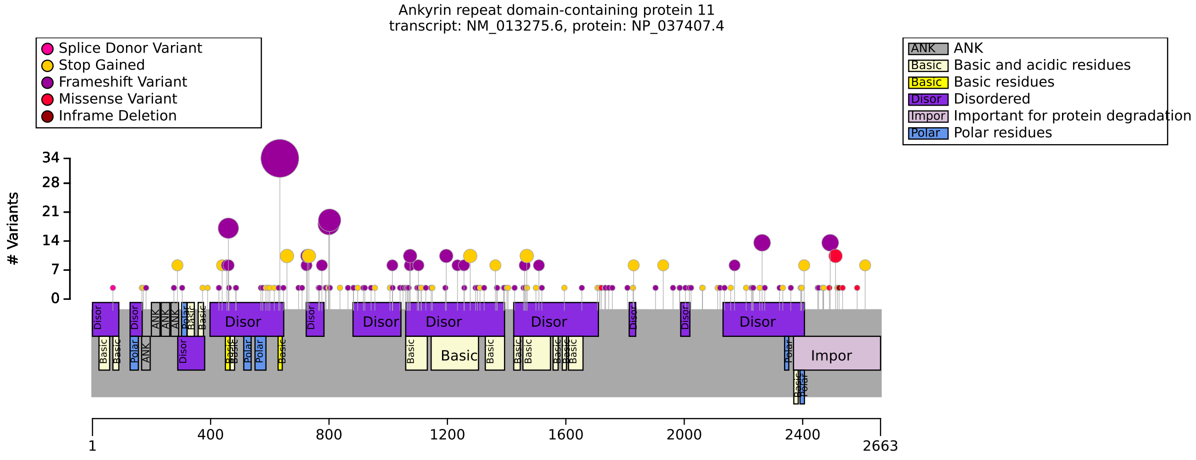

(a) Distribution of variants in *ANKRD11*

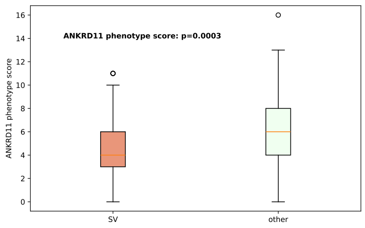

(b) ANKRD11 phenotypical score. Mean for structural variants: 4.62, and for other variants: 5.94

Genotype (A)	Genotype (B)	total tests performed	significant results
SV	other	23	0
exon 9	other	23	0
c.1903_1907del	other	23	0

(c) Fisher Exact Test performed to compare HPO annotation frequency with respect to SV, exon 9, and c.1903_1907del.

Description	Variable	Genotype (A)	Genotype (B)	p-value	xrefs
ANKRD11 phenotype score	HPO group count	SV	other	2.64×10^{-4}	[3]
ANKRD11 phenotype score	HPO group count	FEMALE	MALE	0.0075	-

(d) AKKRD11 phenotype score for structural variants: 4.62; other variants: 5.94. male/female comparison: Female 5.28, Male: 6.15.

Figure S5: The cohort comprised 337 individuals (143 females, 175 males, 19 with unknown sex). A total of 48 HPO terms were used to annotate the cohort. Disease diagnosis: KBG syndrome (OMIM:148050). Validated results on ANKRD11 phenotypical score. Other results did not survive multiple testing correction. Awamleh et al. (2023) stated that no significant DNAm differences based on sex at the identified KBGS-specific signature sites [4]. We are aware of no other publication that mentions sex-specific differences in KBGS. A total of 163 unique variant alleles were found in *ANKRD11* (transcript: `NM_013275.6`, protein id: `NP_037407.4`).

ASPM

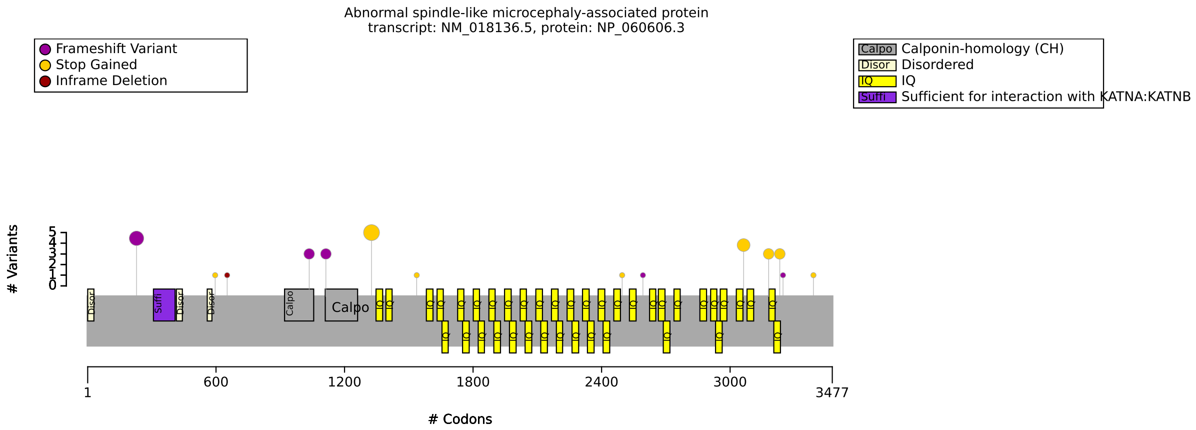

(a) Distribution of variants in *ASPM*

Genotype (A)	Genotype (B)	total tests performed	significant results
N Term/N Term OR N Term/other	other/other	14	0
FEMALE	MALE	14	0

(b) Fisher Exact Test performed to compare HPO annotation frequency with respect to N Term/N Term OR N Term/other and other/other, as well as male/female comparison.

Figure S6: The cohort comprised 22 individuals (14 females, 8 males). A total of 17 HPO terms were used to annotate the cohort. Disease diagnosis: Microcephaly 5, primary, autosomal recessive (OMIM:608716). No statistically significant results identified. A total of 15 unique variant alleles were found in *ASPM* (transcript: `NM_018136.5`, protein id: `NP_060606.3`).

ATP6V0C

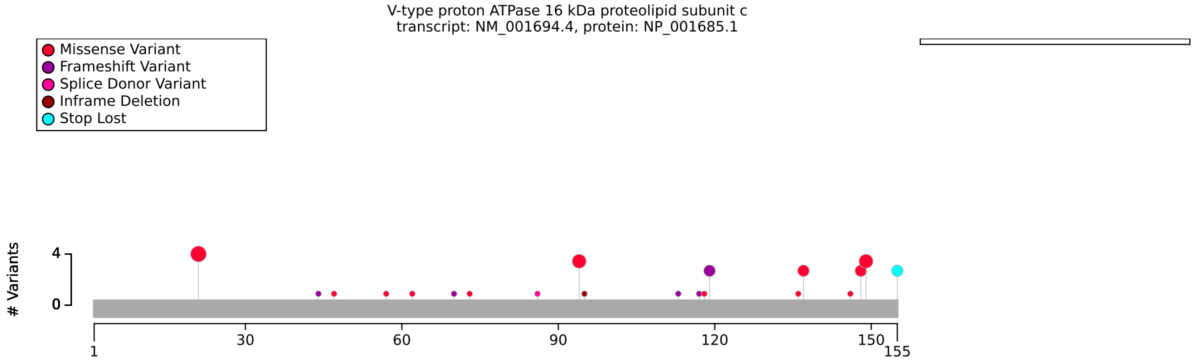

(a) Distribution of variants in *ATP6V0C*

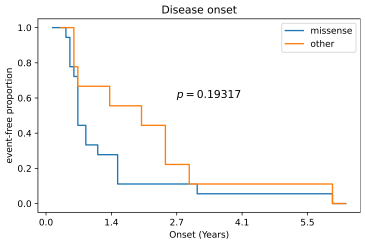

(b) Disease onset for *ATP6V0C* missense vs. other variants.

Genotype (A)	Genotype (B)	total tests performed	significant results
1-100	100+	80	0
missense	other	80	0
FEMALE	MALE	80	0

(c) Fisher Exact Test performed to compare HPO annotation frequency with respect to genotypes.

Description	Variable	Genotype (A)	Genotype (B)	p-value	xrefs
Compute time until OMIM:620465 onset	Onset of OMIM:620465	missense	other	0.193	-

(d) Onset of OMIM:620465 to compare missense and other with respect to Onset of OMIM:620465.

Figure S7: The cohort comprised 31 individuals (12 females, 19 males). A total of 83 HPO terms were used to annotate the cohort. Disease diagnosis: Epilepsy, early-onset, 3, with or without developmental delay (OMIM:620465). to do. A total of 23 unique variant alleles were found in *ATP6V0C* (transcript: `NM_001694.4`, protein id: `NP_001685.1`).

ATP13A2

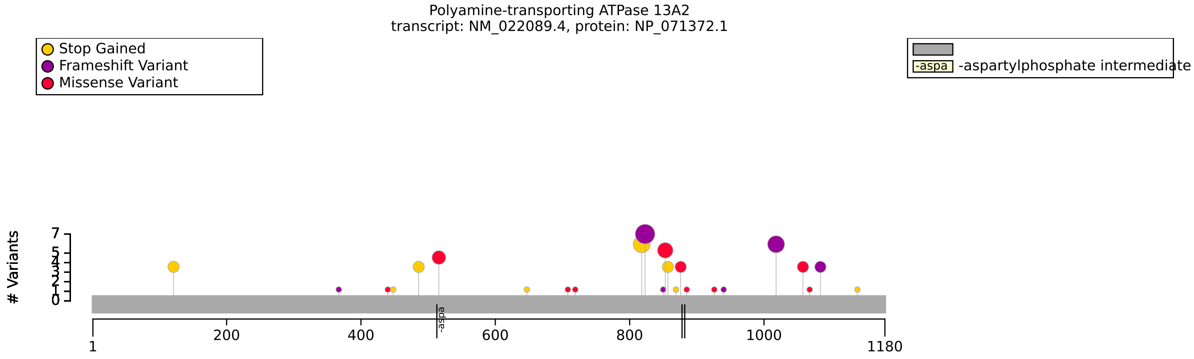

(a) Distribution of variants in *ATP13A2*

HPO term	OMIM:606693	OMIM:617225	p-value	adj. p-value
Parkinsonism [HP:0001300]	28/28 (100%)	3/11 (27%)	2.68×10^{-6}	7.78×10^{-5}
Bradykinesia [HP:0002067]	30/32 (94%)	4/10 (40%)	9.15×10^{-4}	0.013

(b) Fisher Exact Test performed to compare HPO annotation frequency with respect to Kufor-Rakeb syndrome (OMIM:606693) and Spastic paraplegia 78, autosomal recessive (OMIM:617225). Total of 29 tests were performed.

Genotype (A)	Genotype (B)	total tests performed	significant results
missense/missense OR missense/other	other/other	30	0

(c) Fisher Exact Test performed to compare HPO annotation frequency with respect to genotypes.

Figure S8: The cohort comprised 45 individuals (18 females, 27 males). A total of 71 HPO terms were used to annotate the cohort. Disease diagnoses: Kufor-Rakeb syndrome (OMIM:606693) (34 individuals), Spastic paraplegia 78, autosomal recessive (OMIM:617225) (11 individuals). As expected, the frequency of Parkinsonian manifestations is higher in Kufor-Rakeb syndrome, a rare autosomal recessive form of juvenile-onset atypical Parkinson disease. A total of 24 unique variant alleles were found in *ATP13A2* (transcript: `NM_022089.4`, protein id: `NP_071372.1`).

BRD4

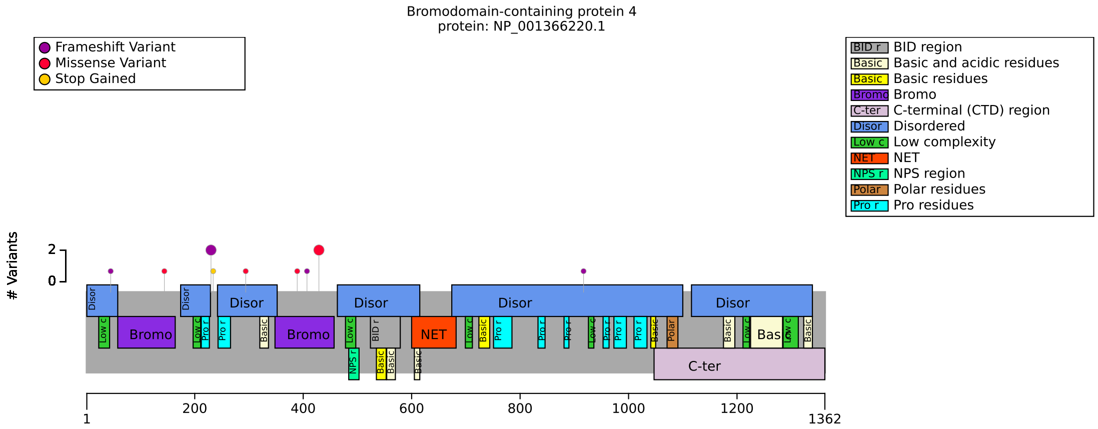

(a) Distribution of variants in *BRD4*

Genotype (A)	Genotype (B)	total tests performed	significant results
ablation	other	27	0
NIPBL	BRD4	41	0

(b) Fisher Exact Test performed to compare HPO annotation frequency with respect to genotypes.

Figure S9: The cohort comprised 18 individuals (8 females, 10 males). A total of 40 HPO terms were used to annotate the cohort. Disease diagnosis: Cornelia de Lange syndrome 6 (OMIM:620568). A total of 10 unique variant alleles were found in *BRD4* (transcript: `NM_001379291.1`, protein id: `NP_001366220.1`).

CHD8

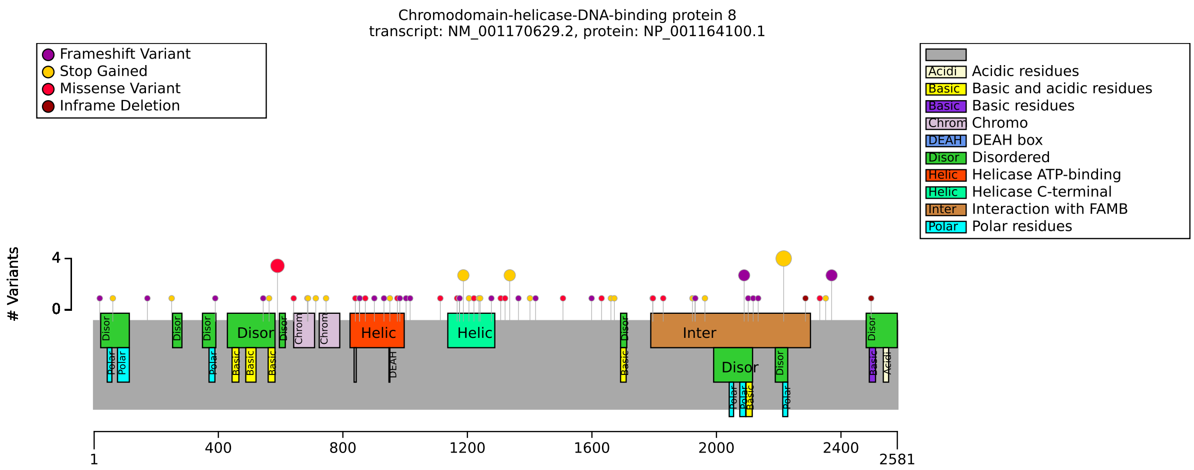

(**a**) Distribution of variants in *CHD8*

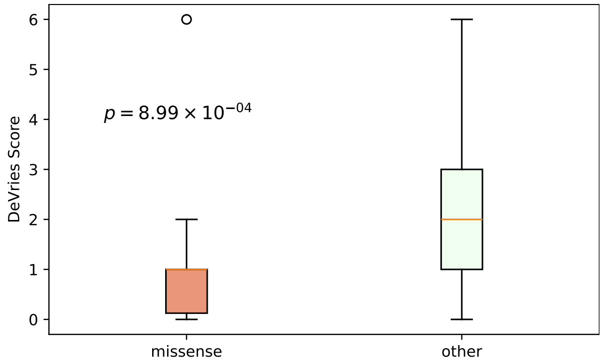

(**b**) De Vries Score to compare Missense and other variants.

Genotype (A)	Genotype (B)	total tests performed	significant results
Missense	Not Missense	36	0
FEMALE	MALE	36	0

(**c**) Fisher Exact Test performed to compare HPO annotation frequency with respect to genotypes.

Description	Variable	Genotype (A)	Genotype (B)	p-value	xrefs
De Vries score	De Vries score	Missense	Not Missense	8.99×10^{-4}	[5]
De Vries score	De Vries score	FEMALE	MALE	0.006	-

(d) De Vries Score to compare Missense and Other variants and for M/F sex differences.

Figure S10: The cohort comprised 79 individuals (26 females, 53 males). 1 of these individuals was reported to be deceased. A total of 81 HPO terms were used to annotate the cohort. Disease diagnosis: Intellectual developmental disorder with autism and macrocephaly (OMIM:615032). Dingemans et al. (2022) identified a correlation between the severity of the phenotypes (as measured by a phenotype severity score termed a DeVries test) and missense variants on the CHD8 gene, specifically that those with a missense variant were significantly less affected than other individuals [5]. A total of 70 unique variant alleles were found in *CHD8* (transcript: `NM_001170629.2`, protein id: `NP_001164100.1`).

CLDN16

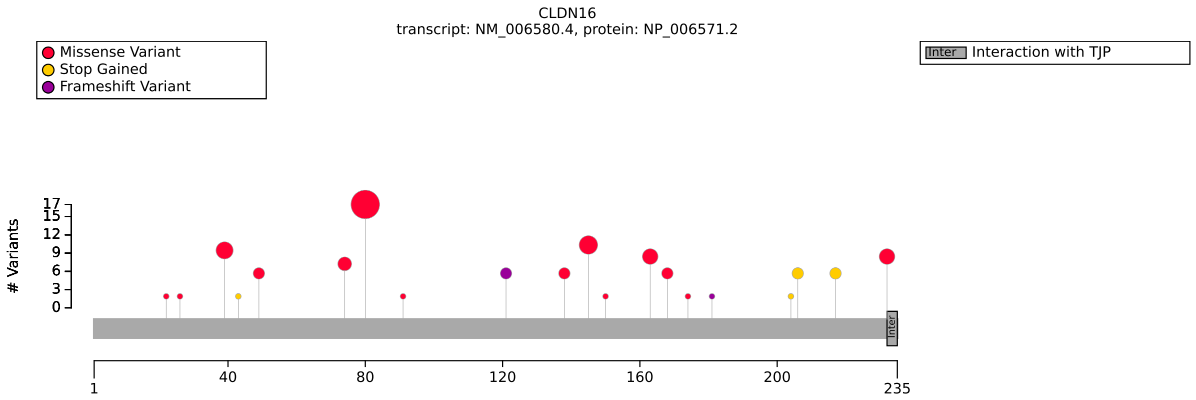

(a) Distribution of variants in *CLDN16*

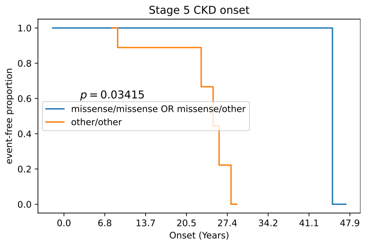

(b) Onset of stage 5 chronic kidney disease for *CLDN16* missense vs. other variants. This result is similar to that of Konrad et al. (2008) [6].

Genotype (A)	Genotype (B)	total tests performed	significant results
Leu81Phe/Leu81Phe	other/other OR Leu81Phe/other	26	0
missense/missense OR missense/other	other/other	23	0
FEMALE	MALE	27	0

(c) Fisher Exact Test performed to compare HPO annotation frequency with respect to genotypes.

Description	Variable	Genotype (A)	Genotype (B)	p-value	xrefs
Hypomagnesemia 3, renal (OMIM:248250) disease onset	Onset of OMIM:248250	missense/missense OR missense/other	other/other	0.333	[6]

(d) Onset of OMIM:248250 to compare missense/missense OR missense/other and other/other with respect to Onset of OMIM:248250.

Description	Variable	Genotype (A)	Genotype (B)	p-value	xrefs
Survival analysis: Stage 5 chronic kidney disease	Onset of HP:0003774	missense/missense OR missense/other	other/other	0.034	[6]

(e) Onset of Stage 5 chronic kidney disease to compare missense/missense OR missense/other and other/other with respect to Onset of HP:0003774.

Figure S11: The cohort comprised 51 individuals (21 females, 29 males, 1 with unknown sex). A total of 53 HPO terms were used to annotate the cohort. Disease diagnosis: Hypomagnesemia 3, renal (OMIM:248250). A total of 23 unique variant alleles were found in *CLDN16* (transcript: `NM_006580.4`, protein id: `NP_006571.2`).

CNTNAP2

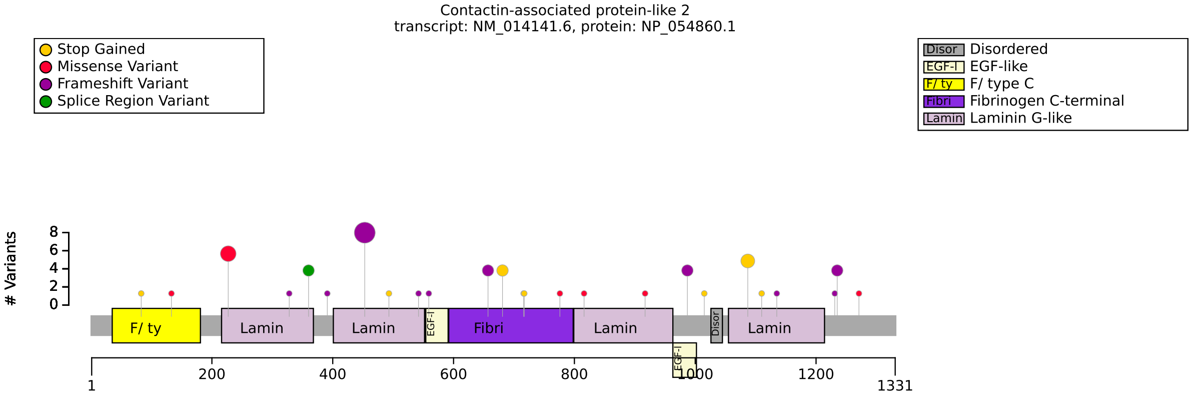

(a) Distribution of variants in *CNTNAP2*

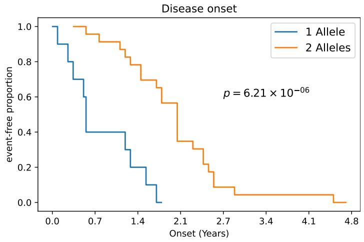

(b) Disease onset: Count of pathogenic *CNTNAP2* alleles

Genotype (A)	Genotype (B)	total tests performed	significant results
Ablation/Ablation	other/other OR Ablation/other	46	0
FEMALE	MALE	48	0
1 allele	2 alleles	43	0

(c) Fisher Exact Test performed to compare HPO annotation frequency with respect to genotypes.

Description	Variable	Genotype (A)	Genotype (B)	p-value	xrefs
Pitt-Hopkins like syndrome 1 (OMIM:610042) disease onset	Onset of OMIM:610042	1 allele	2 alleles	6.21×10^{-6}	-

(d) Onset of OMIM:610042 to compare 1 allele and 2 alleles with respect to Onset of OMIM:610042.

Figure S12: The cohort comprised 63 individuals (29 females, 32 males, 2 with unknown sex). 2 of these individuals were reported to be deceased. A total of 72 HPO terms were used to annotate the cohort. Disease diagnosis: Pitt-Hopkins like syndrome 1 (OMIM:610042). D'Onofrio et al. (2023) reported several GPCs, but the authors did not apply multiple-testing correction [7]. These authors did not perform survival analysis for age of onset of disease. A total of 30 unique variant alleles were found in *CNTNAP2* (transcript: `NM_014141.6`, protein id: `NP_054860.1`).

COQ4

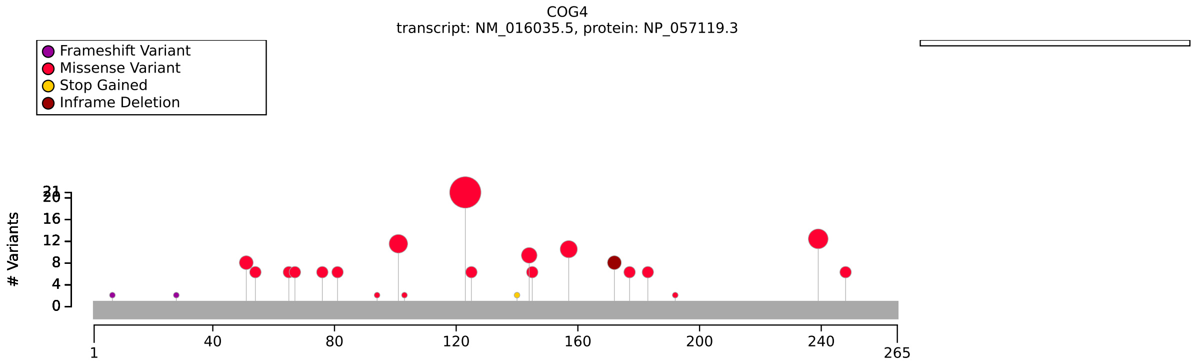

(a) Distribution of variants in *COQ4*

Genotype (A)	Genotype (B)	total tests performed	significant results
missense/other OR missense/missense	other/other	4	0
G124S/other OR G124S/G124S	other/other	13	0
G124S/other OR G124S/G124S	other/other	13	0
Exon 1-4/other OR Exon 1-4/Exon 1-4	other/other	13	0

(b) Fisher Exact Test performed to compare HPO annotation frequency with respect to genotypes.

Figure S13: The cohort comprised 51 individuals (30 females, 21 males). 19 of these individuals were reported to be deceased. A total of 91 HPO terms were used to annotate the cohort. A previous work found that pathogenic *COQ4* variants in exons 1-4 are associated with less life-threating presentations, late onset, responsiveness to CoQ10 therapy, and a relatively long lifespan, but correction for multiple testing was not performed [8]. Disease diagnoses: Coenzyme Q10 deficiency, primary, 7 (OMIM:616276) (35 individuals), Spastic ataxia 10, autosomal recessive (OMIM:620666) (16 individuals). A total of 32 unique variant alleles were found in *COQ4* (transcript: `NM_016035.5`, protein id: `NP_057119.3`).

COL3A1

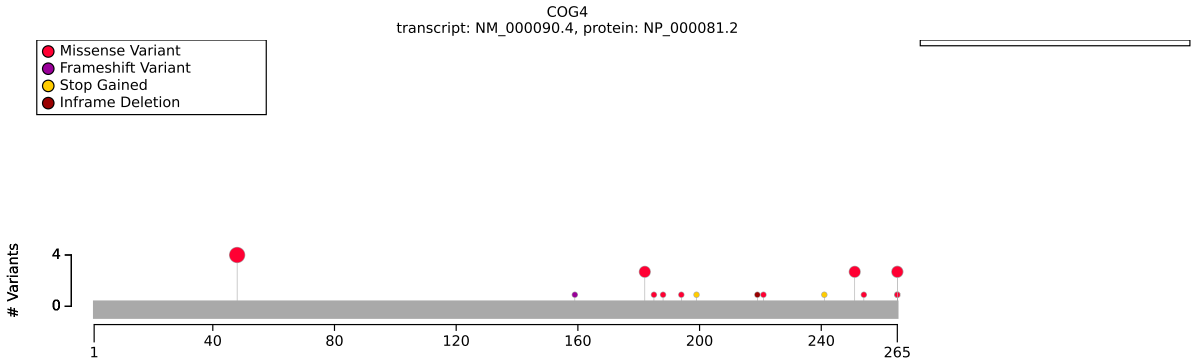

(a) Distribution of variants in *COL3A1*

Genotype (A)	Genotype (B)	total tests performed	significant results
Missense	Other	20	0
Missense	Other	20	0
triple helix region	Other	20	0
OMIM:130050	OMIM:618343	15	0
Gly missense	Other	20	0

(b) Fisher Exact Test performed to compare HPO annotation frequency with respect to genotypes.

Description	Variable	Genotype (A)	Genotype (B)	p-value	xrefs
Age of OMIM:130050 onset	Onset of OMIM:130050	missense	other	0.317	-

(c) Onset of OMIM:130050 to compare missense and other with respect to Onset of OMIM:130050.

Figure S14: The cohort comprised 41 individuals (24 females, 17 males). A total of 43 HPO terms were used to annotate the cohort. Disease diagnoses: Ehlers-Danlos syndrome, vascular type (OMIM:130050) (35 individuals), Polymicrogyria with or without vascular-type EDS (OMIM:618343) (6 individuals). No significant GPC identified. Frank et al (2015) found that glycine missense variants were associated with a higher degree of severity in a cohort of 215 individuals. Primary data was not made available. A total of 38 unique variant alleles were found in *COL3A1* (transcript: `NM_000090.4`, protein id: `NP_000081.2`).

CTCF

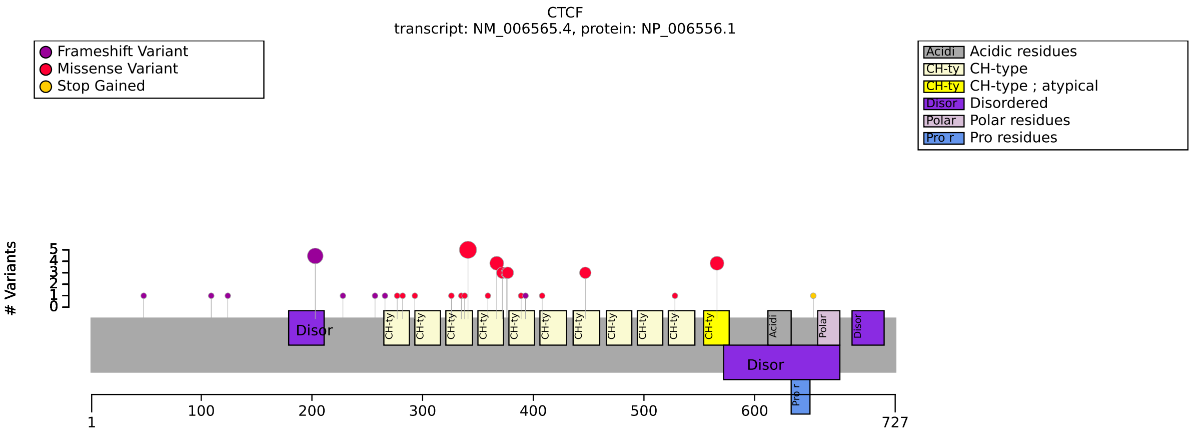

(a) Distribution of variants in *CTCF*

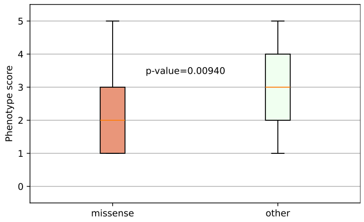

(b) DeVries score to compare *CTCF* missense vs. other variants.

Genotype (A)	Genotype (B)	total tests performed	significant results
missense	other	27	0

(c) Fisher Exact Test performed to compare HPO annotation frequency with respect to genotypes.

Description	Variable	Genotype (A)	Genotype (B)	p-value	xrefs
A phenotypic severity score for individuals with intellectual disability	De Vries score	missense	other	0.009	-

(d) De Vries Score to compare missense and other with respect to De Vries score.

Figure S15: The cohort comprised 46 individuals (18 females, 28 males). A total of 110 HPO terms were used to annotate the cohort. Disease diagnosis: Intellectual developmental disorder, autosomal dominant 21 (OMIM:615502). Previously, a group did not observe a correlation between the location of the variants and the overall severity of various phenotypes, including the level of intellectual function, presence of congenital anomalies, or poor growth [9], but analysis using a score such as the DeVries score was not attempted. A total of 32 unique variant alleles were found in *CTCF* (transcript: `NM_006565.4`, protein id: `NP_006556.1`).

CYP21A2

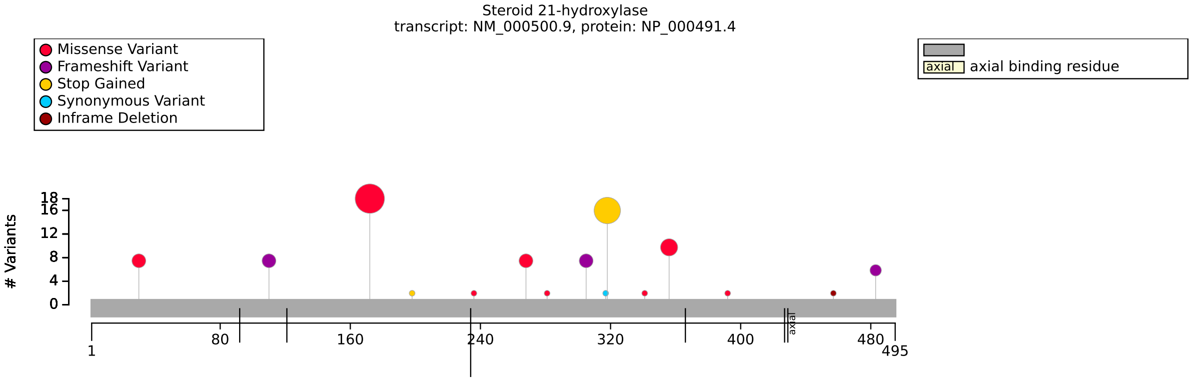

(a) Distribution of variants in *CYP21A2*

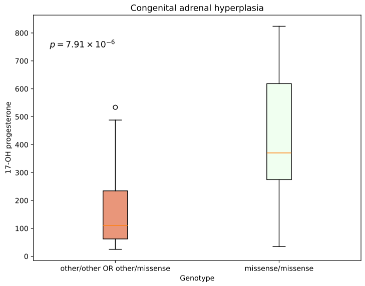

(b) t-test. 17-hydroxyprogesterone level. *CYP21A2* missense variants are compared with other variants.

Description	Variable	Genotype (A)	Genotype (B)	p-value	xrefs
17-Hydroxyprogesterone [Mass/volume] in Serum or Plasma [LOINC:1668-3]	LOINC:1668-3	other/other OR other/missense	missense/missense	7.91×10^{-6}	[10]

(c) t-test to compare other/other OR other/missense and missense/missense with respect to LOINC:1668-3.

Figure S16: The cohort comprised 69 individuals (34 females, 35 males). A total of 27 HPO terms were used to annotate the cohort. Disease diagnosis: Adrenal hyperplasia, congenital, due to 21-hydroxylase deficiency (OMIM:201910). It is assumed that the mildest mutation determines the phenotype in compound heterozygotes. Several missense variants display the highest residual activities. High levels of 17-hydroxyprogesterone may be observed with 21-hydroxylase deficiency [10]. A total of 22 unique variant alleles were found in *CYP21A2* (transcript: `NM_000500.9`, protein id: `NP_000491.4`).

EHMT1

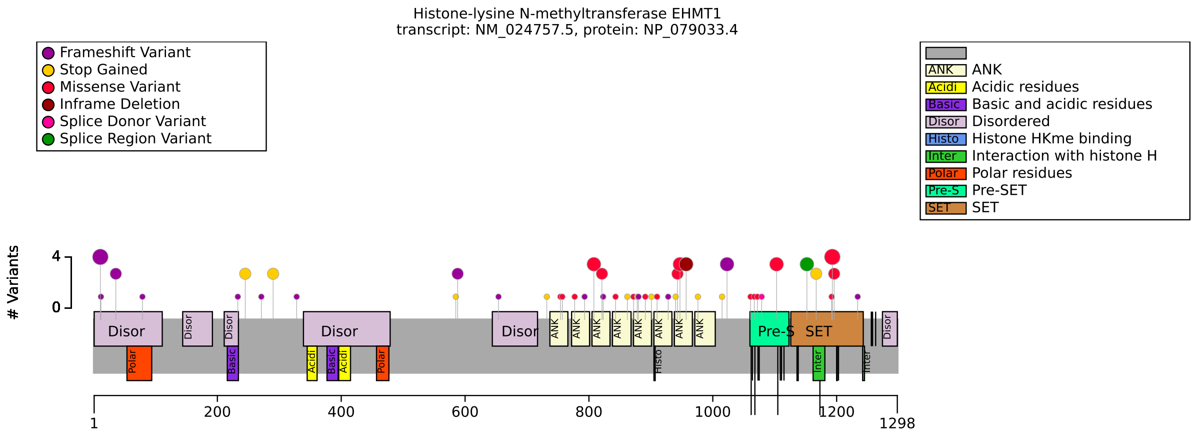

(a) Distribution of variants in *EHMT1*

HPO term	N Term Frameshift	other	p-value	adj. p-value
Attention deficit hyperactivity disorder [HP:0007018]	4/8 (50%)	3/96 (3%)	4.83×10^{-4}	0.029

(b) Fisher Exact Test performed to compare HPO annotation frequency with respect to N Term Frameshift and other. Total of 60 tests were performed.

Genotype (A)	Genotype (B)	total tests performed	significant results
Missense	Other	62	0
FEMALE	MALE	62	0
ANKR	other	62	0
SET	other	62	0

(c) Fisher Exact Test performed to compare HPO annotation frequency with respect to genotypes.

Figure S17: The cohort comprised 125 individuals (81 females, 44 males). A total of 60 HPO terms were used to annotate the cohort. Disease diagnosis: Kleefstra syndrome 1 (OMIM:610253). Rots et al. reported several correlations [11]. Multiple-testing correction was not performed. Frazier et al (2025) also reported significant correlations but the original data was not made available [12]. A total of 62 unique variant alleles were found in *EHMT1* (transcript: `NM_024757.5`, protein id: `NP_079033.4`).

EZH1

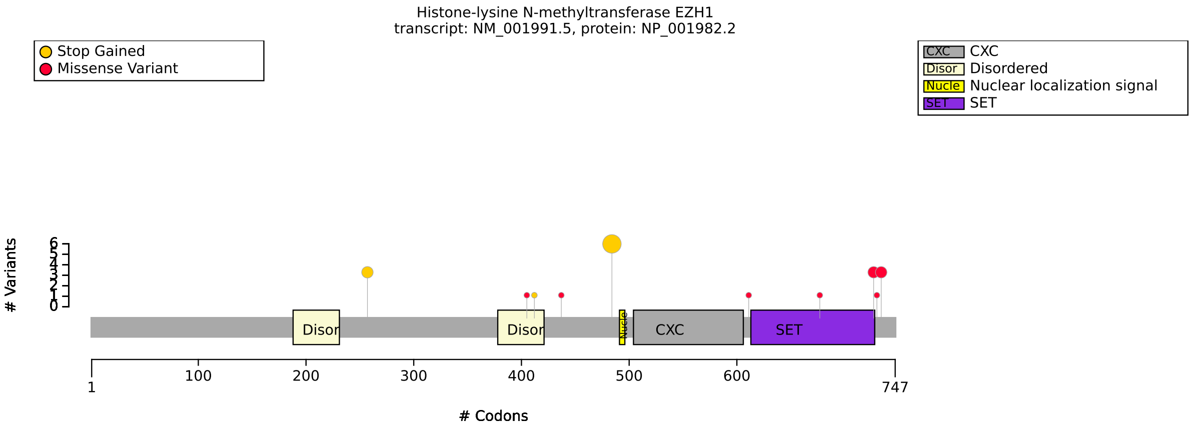

(a) Distribution of variants in *EZH1*

Genotype (A)	Genotype (B)	total tests performed	significant results
1 allele	2 alleles	33	0

(b) Fisher Exact Test performed to compare HPO annotation frequency with respect to monoallelic and biallelic pathogenic variants.

Figure S18: The cohort comprised 19 individuals (10 females, 8 males, 1 with unknown sex). A total of 106 HPO terms were used to annotate the cohort. Disease diagnosis: *EZH1*-related neurodevelopmental disorder (OMIM:601674). No statistically significant results identified. A total of 12 unique variant alleles were found in *EZH1* (transcript: `NM_001991.5`, protein id: `NP_001982.2`). In the original publication, it is stated that patients show neurodevelopmental delay with variable clinical presentations regardless of variant type and zygosity [13].

FBN1

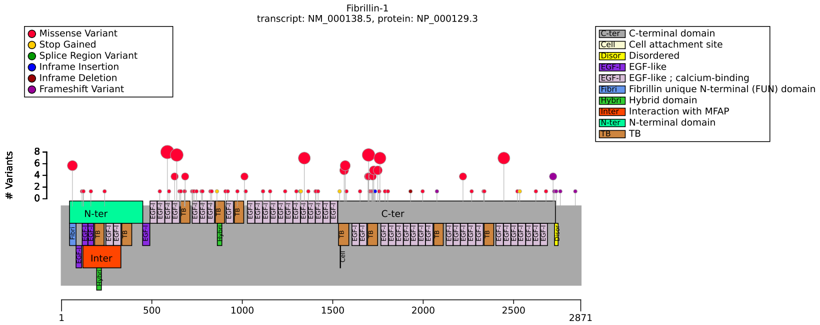

(a) Distribution of variants in *FBN1*

HPO term	missense	other	p-value	adj. p-value
Arachnodactyly [HP:0001166]	34/81 (42%)	18/22 (82%)	0.001	0.026
Thoracic aortic aneurysm [HP:0012727]	25/64 (39%)	12/14 (86%)	0.002	0.026
Hyperextensibility of the finger joints [HP:0001187]	0/78 (0%)	3/14 (21%)	0.003	0.026

(b) Fisher Exact Test: missense vs. other. Total of 27 tests were performed.

HPO term	TB domain	cbEGF	p-value	adj. p-value
Ectopia lentis [HP:0001083]	9/18 (50%)	48/59 (81%)	0.013	0.038
Mitral valve prolapse [HP:0001634]	1/28 (4%)	13/47 (28%)	0.012	0.038
Disproportionate tall stature [HP:0001519]	2/40 (5%)	12/43 (28%)	0.007	0.029
Tall stature [HP:0000098]	7/38 (18%)	21/40 (52%)	0.002	0.011
Severe short stature [HP:0003510]	15/36 (42%)	0/24 (0%)	1.36×10^{-4}	9.04×10^{-4}
Proportionate short stature [HP:0003508]	20/36 (56%)	0/24 (0%)	2.35×10^{-6}	2.35×10^{-5}
Short stature [HP:0004322]	23/39 (59%)	0/24 (0%)	4.39×10^{-7}	8.79×10^{-6}

(c) Fisher Exact Test: TB domain vs cbEGF. Total of 20 tests were performed.

HPO term	exon 37	other	p-value	adj. p-value
Arachnodactyly [HP:0001166]	0/8 (0%)	52/95 (55%)	0.003	0.019
Ectopia lentis [HP:0001083]	1/9 (11%)	78/100 (78%)	1.12×10^{-4}	0.001
Stiff skin [HP:0030053]	8/9 (89%)	0/50 (0%)	4.06×10^{-9}	8.52×10^{-8}

(d) Fisher Exact Test: exon 37 vs. other. Total of 21 tests were performed.

HPO term	fs last two	other	p-value	adj. p-value
Hyperextensibility of the finger joints [HP:0001187]	2/2 (100%)	1/90 (1%)	7.17×10^{-4}	0.012

(e) Fisher Exact Test: Frameshift in last two exons vs other. Total of 12 tests were performed.

Figure S19: The cohort comprised 144 individuals (57 females, 55 males, 32 with unknown sex). 4 of these individuals were reported to be deceased. A total of 109 HPO terms were used to annotate the cohort. Numerous articles on genotype-phenotype correlations have been published [14, 15, 16], but the available data is said to be be imprecise and incomplete [17] and many studies only share aggregate data. Disease diagnoses: Marfan syndrome (n=51), Ectopia lentis, familial (n=44), Geleophysic dysplasia 2 (n=19), Acromicric dysplasia (n=13), Marfan lipodystrophy syndrome (OMIM:616914) (n=9), Stiff skin syndrome (n=8). A total of 94 unique variant alleles were found in *FBN1* (transcript: `NM_000138.5`, protein id: `NP_000129.3`).

FBXL4

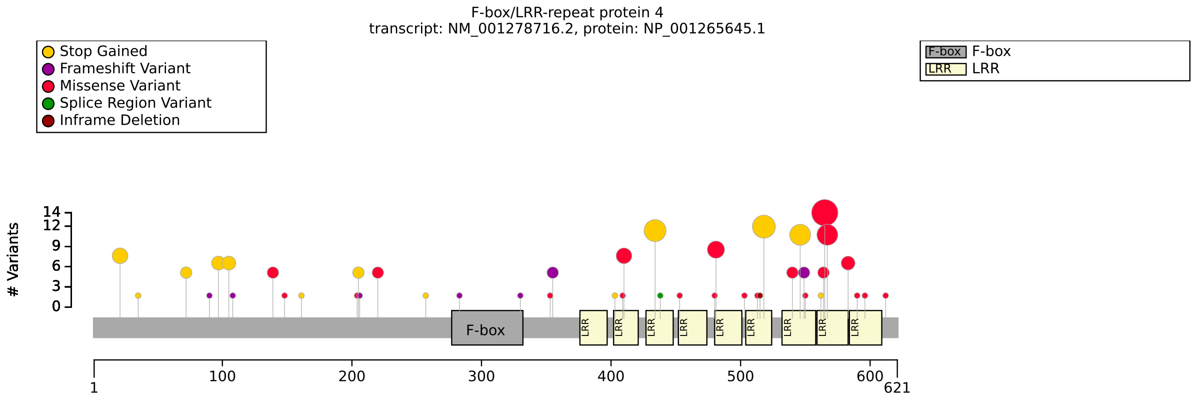

(a) Distribution of variants in *FBXL4*

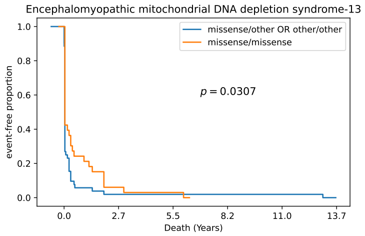

(b) Survival analysis.

HPO term	missense/other OR other/other	missense/missense	p-value	adj. p-value
Feeding difficulties [HP:0011968]	23/24 (96%)	13/27 (48%)	1.73×10^{-4}	0.010

(c) Fisher Exact Test performed to compare HPO annotation frequency with respect to missense/other OR other/other and missense/missense. Total of 55 tests were performed.

Genotype (A)	Genotype (B)	total tests performed	significant results
LRR domain/LRR domain OR LRR domain/other	other/other	55	0
N-Terminal (1-276)/N-Terminal (1-276) OR N-Terminal (1-276)/other	other/other	55	0
FEMALE	MALE	53	0

(d) Fisher Exact Test performed to compare HPO annotation frequency with respect to genotypes.

Description	Variable	Genotype (A)	Genotype (B)	p-value	xrefs
Age of OMIM:615471 onset	Onset of OMIM:615471	missense/other OR other/other	missense/missense	0.031	-
Age at postnatal death	Age of death	missense/other OR other/other	missense/missense	0.080	[18]

(e) Survival analysis for Age of death and onset, comparing compare missense/other OR other/other and missense/missense.

Figure S20: The cohort comprised 95 individuals (37 females, 58 males). 26 of these individuals were reported to be deceased. A total of 91 HPO terms were used to annotate the cohort. Disease diagnosis: Mitochondrial DNA depletion syndrome 13 (encephalomyopathic type) (OMIM:615471). It was reported that pathological variants in *FBXL4* indicated that genotypes with missense variants are frequently associated with longer survival [18]. Using the larger cohort analyzed here, mortality is not significantly associated with missense variants (p-value 0.08), but there was a significant association with age of onset (being later for missense variants) [18]. A total of 51 unique variant alleles were found in *FBXL4* (transcript: `NM_001278716.2`, protein id: `NP_001265645.1`).

FBXO11

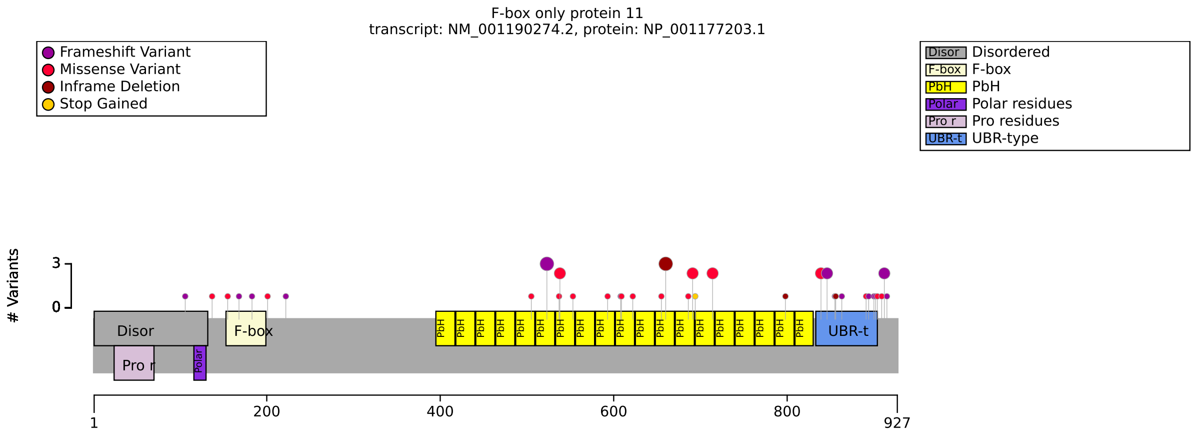

(a) Distribution of variants in *FBXO11*

Genotype (A)	Genotype (B)	total tests performed	significant results
Missense	Other	96	0
FEMALE	MALE	96	0

(b) Fisher Exact Test performed to compare HPO annotation frequency with respect to genotypes.

Figure S21: The cohort comprised 56 individuals (21 females, 35 males). A total of 128 HPO terms were used to annotate the cohort. Disease diagnosis: Intellectual developmental disorder with dysmorphic facies and behavioral abnormalities (OMIM:618089). No statistically significant results identified. A total of 43 unique variant alleles were found in *FBXO11* (transcript: `NM_001190274.2`, protein id: `NP_001177203.1`).

FGD1

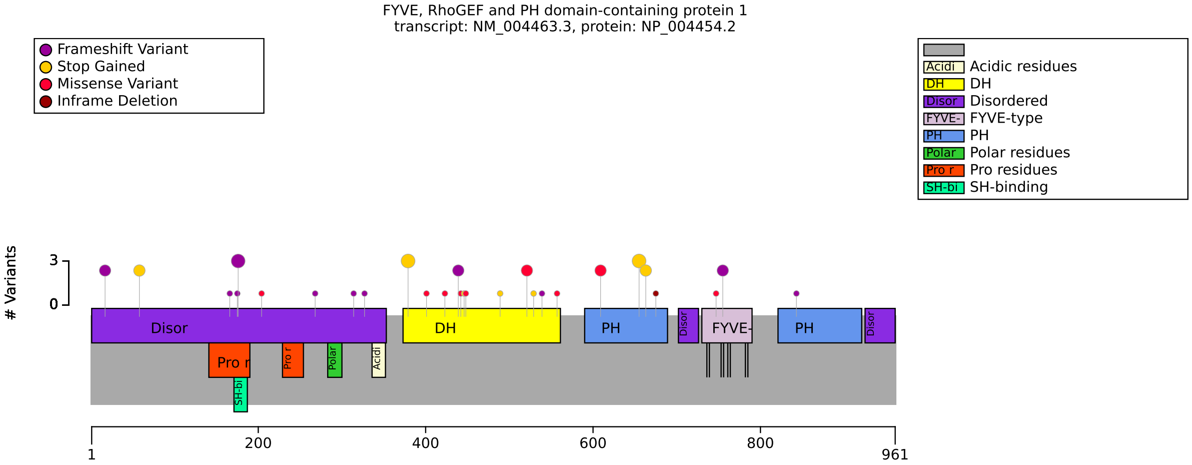

(a) Distribution of variants in *FGD1*

HPO term	Missense	Other	p-value	adj. p-value
Broad foot [HP:0001769]	1/7 (14%)	14/15 (93%)	6.22×10^{-4}	0.032

(b) Fisher Exact Test performed to compare HPO annotation frequency with respect to Missense and Other. Total of 52 tests were performed.

Genotype (A)	Genotype (B)	total tests performed	significant results
DH domain	Other	52	0

(c) Fisher Exact Test performed to compare HPO annotation frequency with respect to genotypes.

Figure S22: The cohort comprised 48 individuals (0 females, 48 males). A total of 73 HPO terms were used to annotate the cohort. Disease diagnosis: Aarskog-Scott syndrome (OMIM:305400). Li et al (2024) identified a number of correlations including a lower frequency of Deformity of foot (8/20) with missense than with ”drastic” variants (29/41); p=0.03. Multiple testing correction was not performed [19, 20]. A total of 35 unique variant alleles were found in *FGD1* (transcript: `NM_004463.3`, protein id: `NP_004454.2`).

FZD5

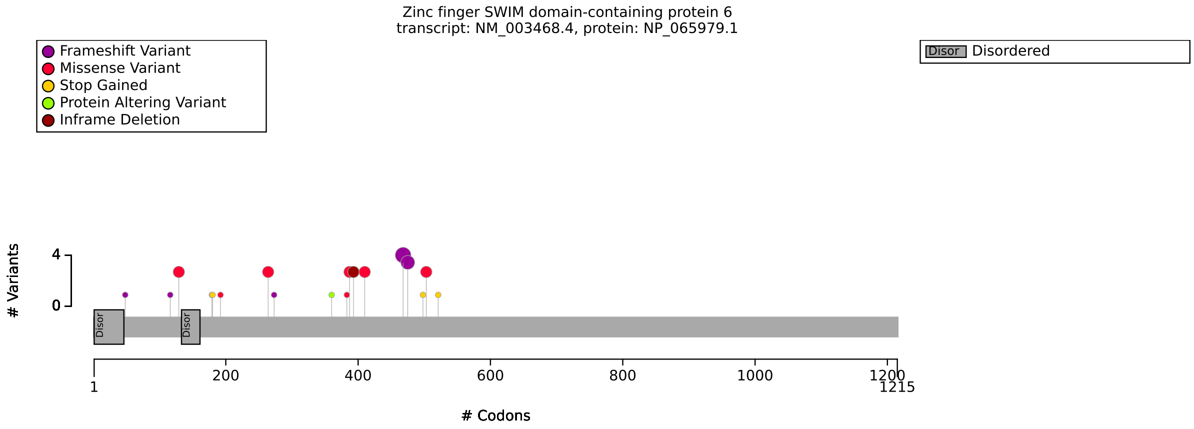

(a) Distribution of variants in *FZD5*

Genotype (A)	Genotype (B)	total tests performed	significant results
missense	other	18	0

(b) Fisher Exact Test performed to compare HPO annotation frequency with respect to genotypes.

Figure S23: The cohort comprised 29 individuals (11 females, 10 males, 8 with unknown sex). A total of 16 HPO terms were used to annotate the cohort. Disease diagnosis: Microphthalmia/coloboma 11 (OMIM:620731). No significant correlations identified. A total of 19 unique variant alleles were found in *FZD5* (transcript: NM_003468.4, protein id: NP_065979.1).

GLI3

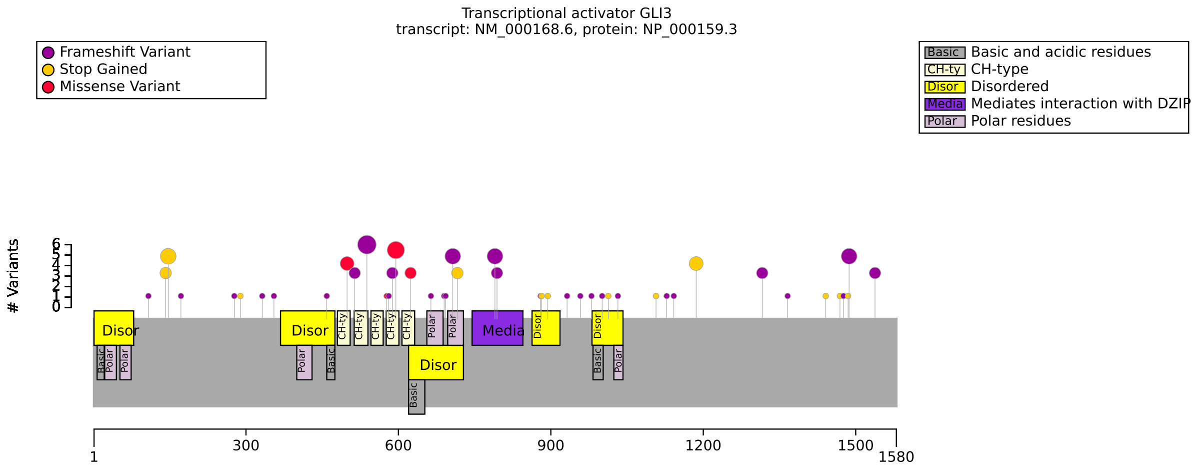

(a) Distribution of variants in *GLI3*

HPO term	Fs in mid region or splice	other	p-value	adj. p-value
Preaxial foot polydactyly [HP:0001841]	5/25 (20%)	32/57 (56%)	0.003	0.021
Y-shaped metacarpals [HP:0006042]	11/23 (48%)	4/56 (7%)	9.94×10^{-5}	0.001
Y-shaped metatarsals [HP:0010567]	11/19 (58%)	4/50 (8%)	3.25×10^{-5}	7.80×10^{-4}
Nail dysplasia [HP:0002164]	7/20 (35%)	2/54 (4%)	0.001	0.009
Anal atresia [HP:0002023]	7/25 (28%)	3/57 (5%)	0.007	0.036

(b) Fisher Exact Test performed to compare HPO annotation frequency with respect to Fs in mid region or splice and other. Total of 24 tests were performed.

HPO term	Truncating variants in Exon 15	other	p-value	adj. p-value
Macrocephaly [HP:0000256]	13/16 (81%)	15/42 (36%)	0.003	0.017
Preaxial foot polydactyly [HP:0001841]	7/28 (25%)	30/54 (56%)	0.010	0.050
Syndactyly [HP:0001159]	5/17 (29%)	33/38 (87%)	4.82×10^{-5}	0.001
Postaxial foot polydactyly [HP:0001830]	9/20 (45%)	2/35 (6%)	8.91×10^{-4}	0.011
Anal atresia [HP:0002023]	8/28 (29%)	2/54 (4%)	0.002	0.017

(c) Fisher Exact Test performed to compare HPO annotation frequency with respect to Truncating variants in Exon 15 and other. Total of 24 tests were performed.

HPO term	Variants in C-terminal third	other	p-value	adj. p-value
Macrocephaly [HP:0000256]	13/15 (87%)	15/43 (35%)	7.31×10^{-4}	0.004
Postaxial hand polydactyly [HP:0001162]	15/16 (94%)	21/49 (43%)	3.40×10^{-4}	0.003
Syndactyly [HP:0001159]	5/16 (31%)	33/39 (85%)	2.25×10^{-4}	0.003
Postaxial foot polydactyly [HP:0001830]	9/16 (56%)	2/39 (5%)	7.35×10^{-5}	0.002

(d) Fisher Exact Test performed to compare HPO annotation frequency with respect to Variants in C-terminal third and other. Total of 24 tests were performed.

Figure S24: The cohort comprised 82 individuals (3 females, 7 males, 72 with unknown sex). A total of 34 HPO terms were used to annotate the cohort. Disease diagnoses: Greig cephalopolysyndactly syndrome (OMIM:175700) (51 individuals), Pallister-Hall syndrome (OMIM:146510) (21 individuals), Polydactyly, postaxial, types A1 and B (OMIM:174200) (10 individuals). Genotype-Phenotype-Correlations in *GLI3* have been extensively investigated, with findings being similar but not identical to those reported here [21, 22, 23, 24, 25, 26, 27]. A total of 49 unique variant alleles were found in *GLI3* (transcript: `NM_000168.6`, protein id: `NP_000159.3`).

HMGCS2

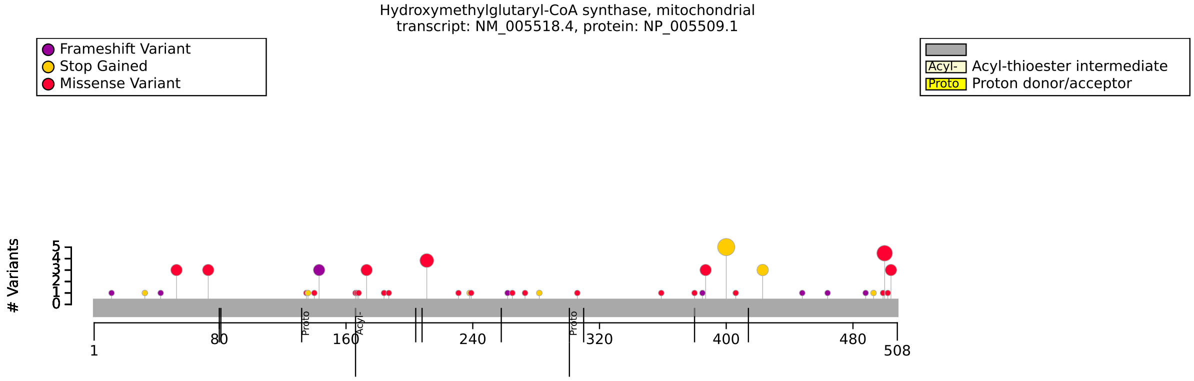

(a) Distribution of variants in *HMGCS2*

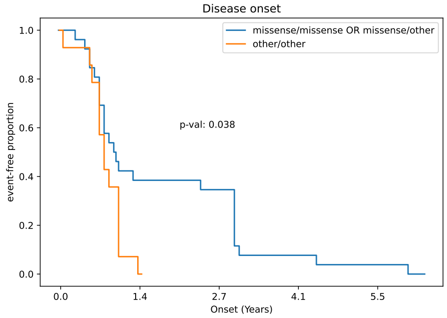

(b) Onset of *HMGCS2* for missense/missense and missense/other vs. other/other variants.

Genotype (A)	Genotype (B)	total tests performed	significant results
missense/missense OR missense/other	other/other	19	0
missense/missense OR missense/other	other/other	19	0
FEMALE	MALE	17	0

(c) Fisher Exact Test performed to compare HPO annotation frequency with respect to genotypes.

Description	Variable	Genotype (A)	Genotype (B)	p-value	xrefs
HMG-CoA synthase-2 deficiency (OMIM:605911) disease onset	Onset of OMIM:605911	missense/missense OR missense/other	other/other	0.038	-

(d) Onset of OMIM:605911 to compare missense/missense OR missense/other and other/other with respect to Onset of OMIM:605911.

Figure S25: The cohort comprised 40 individuals (14 females, 13 males, 13 with unknown sex). 1 of these individuals were reported to be deceased. A total of 44 HPO terms were used to annotate the cohort. Disease diagnosis: HMG-CoA synthase-2 deficiency (OMIM:605911). No previous publications with results on genotype-phenotype correlations in *HMGCS2* were identified. A total of 46 unique variant alleles were found in *HMGCS2* (transcript: `NM_005518.4`, protein id: `NP_005509.1`).

IKZF1

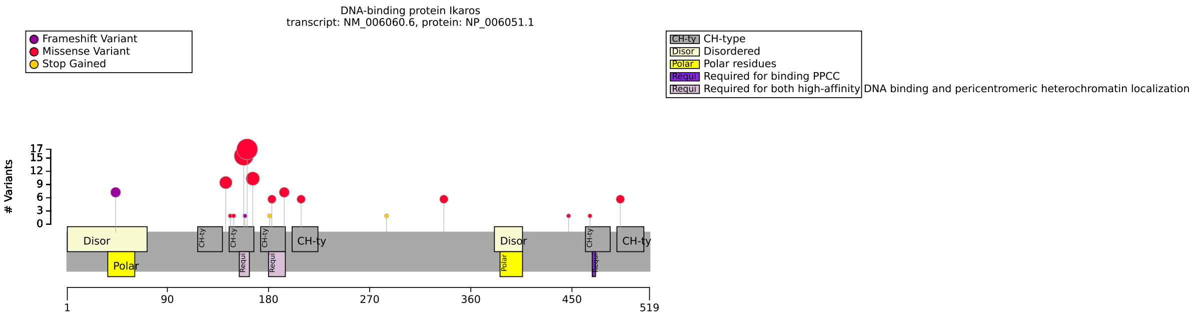

(a) Distribution of variants in *IKZF1*

HPO term	p.Asn159Ser	Other variant	p-value	adj. p-value
Recurrent pneumonia [HP:0006532]	7/9 (78%)	7/39 (18%)	0.001	0.035
T-cell acute lymphoblastic leukemias [HP:0006727]	3/13 (23%)	0/69 (0%)	0.003	0.047

(b) Fisher Exact Test performed to compare HPO annotation frequency with respect to p.Asn159Ser and Other variant. Total of 29 tests were performed.

Genotype (A)	Genotype (B)	total tests performed	significant results
Missense	oher	34	0

(c) Fisher Exact Test performed to compare HPO annotation frequency with respect to genotypes.

Figure S26: The cohort comprised 82 individuals (34 females, 37 males, 11 with unknown sex). 5 of these individuals were reported to be deceased. A total of 66 HPO terms were used to annotate the cohort. Disease diagnosis: Immunodeficiency, common variable, 13 (OMIM:616873). The variant p.Asn159Ser was functionally characterized to be dominant-negative and result in a combined-immunodeficiency phenotype that was distinct from other *IKZF1* variants [28]. A total of 26 unique variant alleles were found in *IKZF1* (transcript: `NM_006060.6`, protein id: `NP_006051.1`).

ITPR1

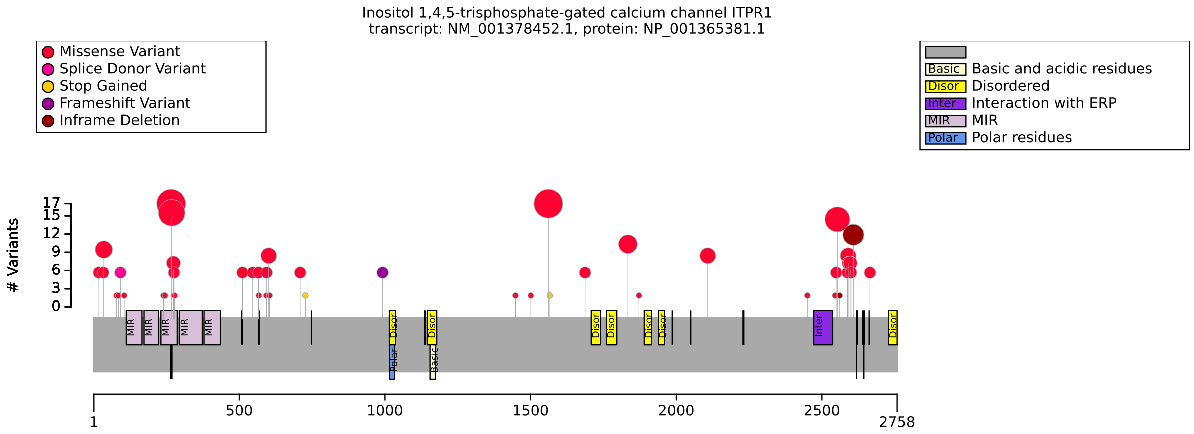

(a) Distribution of variants in *ITPR1*

HPO term	IP3 binding	other	p-value	adj. p-value
Delayed speech and language development [HP:0000750]	13/13 (100%)	37/63 (59%)	0.003	0.019
Neurodevelopmental delay [HP:0012758]	29/29 (100%)	70/90 (78%)	0.003	0.019
Aniridia [HP:0000526]	0/14 (0%)	20/54 (37%)	0.007	0.024
Delayed gross motor development [HP:0002194]	18/18 (100%)	57/83 (69%)	0.005	0.024
Motor delay [HP:0001270]	24/24 (100%)	63/89 (71%)	0.002	0.019
Nystagmus [HP:0000639]	25/26 (96%)	53/78 (68%)	0.003	0.019
Delayed ability to sit [HP:0025336]	11/11 (100%)	42/70 (60%)	0.013	0.043
Delayed ability to walk [HP:0031936]	13/13 (100%)	41/68 (60%)	0.004	0.019

(b) Fisher Exact Test performed to compare HPO annotation frequency with respect to IP3 binding and other. Total of 26 tests were performed.

HPO term	SV Deletion	other	p-value	adj. p-value
Delayed speech and language development [HP:0000750]	0/19 (0%)	50/57 (88%)	1.72×10^{-12}	7.57×10^{-12}
Neurodevelopmental delay [HP:0012758]	0/19 (0%)	99/100 (99%)	4.07×10^{-21}	8.96×10^{-20}
Delayed gross motor development [HP:0002194]	0/19 (0%)	75/82 (91%)	4.04×10^{-15}	2.96×10^{-14}
Motor delay [HP:0001270]	0/19 (0%)	87/94 (93%)	3.90×10^{-16}	4.29×10^{-15}
Global developmental delay [HP:0001263]	0/19 (0%)	48/57 (84%)	1.81×10^{-11}	5.68×10^{-11}
Nystagmus [HP:0000639]	17/17 (100%)	61/87 (70%)	0.006	0.016
Delayed ability to sit [HP:0025336]	0/19 (0%)	53/62 (85%)	4.56×10^{-12}	1.67×10^{-11}
Delayed ability to walk [HP:0031936]	0/19 (0%)	54/62 (87%)	1.47×10^{-12}	7.57×10^{-12}

(c) Fisher Exact Test performed to compare HPO annotation frequency with respect to SV Deletion and other. Total of 22 tests were performed.

HPO term	GS Hotspot	other	p-value	adj. p-value
Aniridia [HP:0000526]	14/20 (70%)	6/48 (12%)	6.13×10^{-6}	1.59×10^{-4}
Delayed gross motor development [HP:0002194]	18/18 (100%)	57/83 (69%)	0.005	0.032
Motor delay [HP:0001270]	19/19 (100%)	68/94 (72%)	0.006	0.032
Delayed ability to sit [HP:0025336]	13/13 (100%)	40/68 (59%)	0.003	0.030
Delayed ability to walk [HP:0031936]	14/14 (100%)	40/67 (60%)	0.003	0.030

(d) Fisher Exact Test performed to compare HPO annotation frequency with respect to GS Hotspot and other. Total of 25 tests were performed.

Figure S27: The cohort comprised 170 individuals (65 females, 41 males, 64 with unknown sex). 1 of these individuals was reported to be deceased. A Fisher exact test for male/female differences revealed no significant associations. A total of 196 HPO terms were used to annotate the cohort. Disease diagnoses: Spinocerebellar ataxia 29, congenital nonprogressive (OMIM:117360) (104 individuals), Gillespie syndrome (OMIM:206700) (39 individuals), Spinocerebellar ataxia 15 (OMIM:606658) (27 individuals). A total of 62 unique variant alleles were found in *ITPR1* (transcript: `NM_001378452.1`, protein id: `NP_001365381.1`).

Kabuki

HPO term	OMIM:147920	OMIM:300867	p-value	adj. p-value
Feeding difficulties [HP:0011968]	8/25 (32%)	55/63 (87%)	7.41×10^{-7}	2.52×10^{-5}
Motor delay [HP:0001270]	4/10 (40%)	58/61 (95%)	1.04×10^{-4}	0.002

(a) Fisher Exact Test performed to compare HPO annotation frequency with respect to OMIM:147920 and OMIM:300867. Total of 34 tests were performed.

Figure S28: The cohort comprised 146 individuals (81 females, 65 males). 5 of these individuals were reported to be deceased. A total of 200 HPO terms were used to annotate the cohort. Disease diagnoses: Kabuki syndrome 2 (OMIM:300867) (81 individuals), Kabuki Syndrome 1 (OMIM:147920) (65 individuals). Previous studies have found that the phenotype of KS2 is different from that of KS1, for example, patients with KS1 type have a higher risk of typical facial features, short stature, and frequent infections compared to those with KS2 [29, 30, 31]. A total of 105 unique variant alleles were found in *n/a* (transcript: `n/a`, protein id: `n/a`).

KCNH5

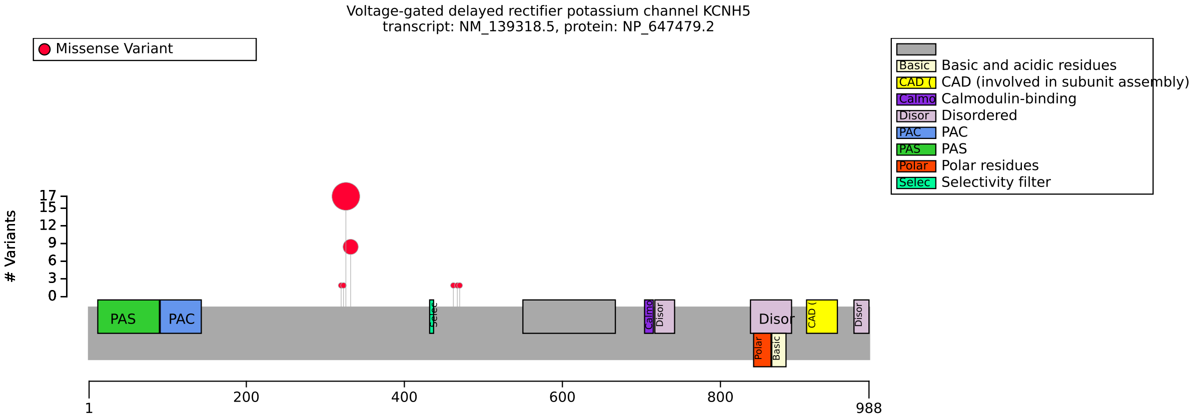

(a) Distribution of variants in *KCNH5*

HPO term	Arg327His	Arg333His	p-value	adj. p-value
Epileptic encephalopathy [HP:0200134]	15/15 (100%)	0/3 (0%)	0.001	0.032

(b) Fisher Exact Test performed to compare HPO annotation frequency with respect to Arg327His and Arg333His. Total of 26 tests were performed.

Genotype (A)	Genotype (B)	total tests performed	significant results
FEMALE	MALE	32	0

(c) Fisher Exact Test performed to compare HPO annotation frequency with respect to genotypes.

Figure S29: The cohort comprised 27 individuals (14 females, 13 males). A total of 28 HPO terms were used to annotate the cohort. Disease diagnosis: Developmental and epileptic encephalopathy 112 (OMIM:620537). Two recurrent variants have been reported, p.Arg327His and p.Arg333His, both of which are located in or near the functionally critical voltage-sensing or pore domains. Happ et al (2023) state that in their cohort, Individuals with the recurrent p.Arg333His variant had a self-limited drug-responsive focal or generalized epilepsy and normal intellect, whereas the recurrent p.Arg327His variant was associated with infantile-onset DEE [32]. A total of 8 unique variant alleles were found in *KCNH5* (transcript: `NM_139318.5`, protein id: `NP_647479.2`).

KDM6A

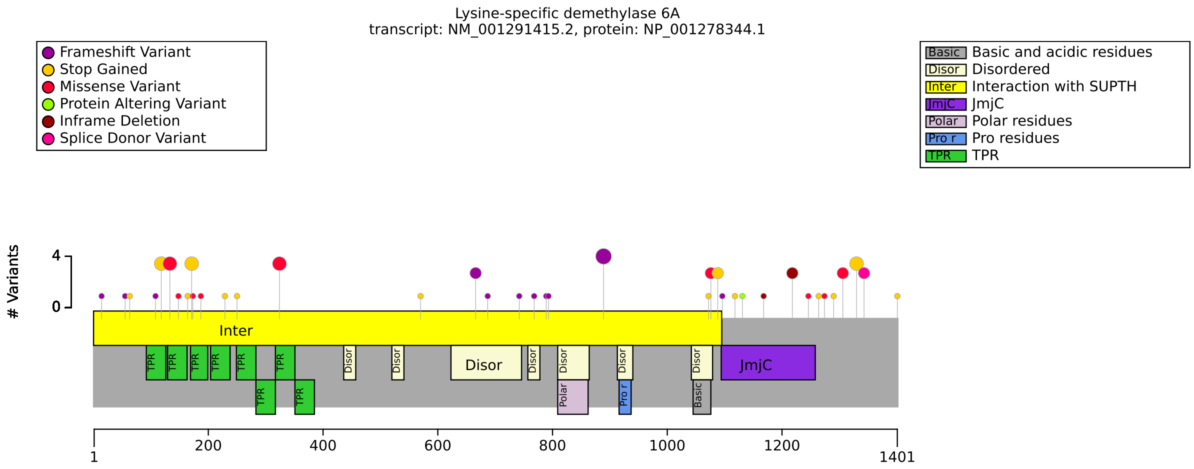

(a) Distribution of variants in *KDM6A*

HPO term	FEMALE	MALE	p-value	adj. p-value
Intellectual disability, severe [HP:0010864]	7/25 (28%)	14/18 (78%)	0.002	0.008

(b) Fisher Exact Test performed to compare HPO annotation frequency with respect to FEMALE and MALE. Total of 4 tests were performed. A similar sex difference was reported in ref. [33].

HPO term	p.Asn891ValfsTer27	Other	p-value	adj. p-value
Pulmonic stenosis [HP:0001642]	2/2 (100%)	0/59 (0%)	5.46×10^{-4}	0.016

(c) Fisher Exact Test performed to compare HPO annotation frequency with respect to p.Asn891ValfsTer27 and Other. Total of 29 tests were performed.

Genotype (A)	Genotype (B)	total tests performed	significant results
missense	other	47	0
SV	other	47	0

(d) Fisher Exact Test performed to compare HPO annotation frequency with respect to genotypes.

Figure S30: The cohort comprised 81 individuals (46 females, 35 males). A total of 87 HPO terms were used to annotate the cohort. Disease diagnosis: Kabuki syndrome 2 (OMIM:300867). . A total of 53 unique variant alleles were found in *KDM6A* (transcript: NM_001291415.2, protein id: NP_001278344.1).

KDM6B

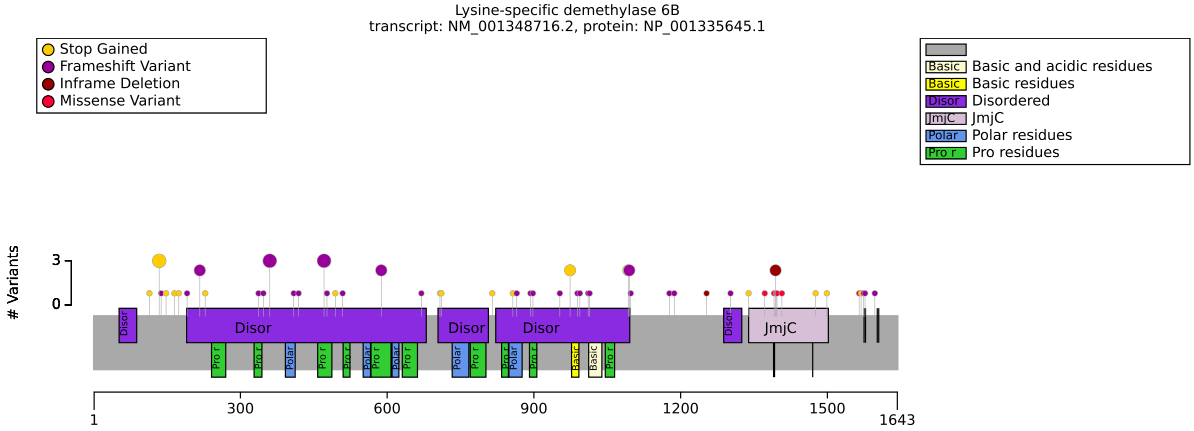

(a) Distribution of variants in *KDM6B*

Genotype (A)	Genotype (B)	total tests performed	significant results
JmjC domain	Other	54	0
N term	C term	54	0

(b) Fisher Exact Test performed to compare HPO annotation frequency with respect to genotypes.

Figure S31: The cohort comprised 73 individuals (19 females, 54 males). A total of 251 HPO terms were used to annotate the cohort. Disease diagnosis: Neurodevelopmental disorder with coarse facies and mild distal skeletal abnormalities (OMIM:618505). Stolerman et al (2016) identified no significant genotype-phenotype correlations [34] A total of 61 unique variant alleles were found in *KDM6B* (transcript: `NM_001348716.2`, protein id: `NP_001335645.1`).

KMT2D

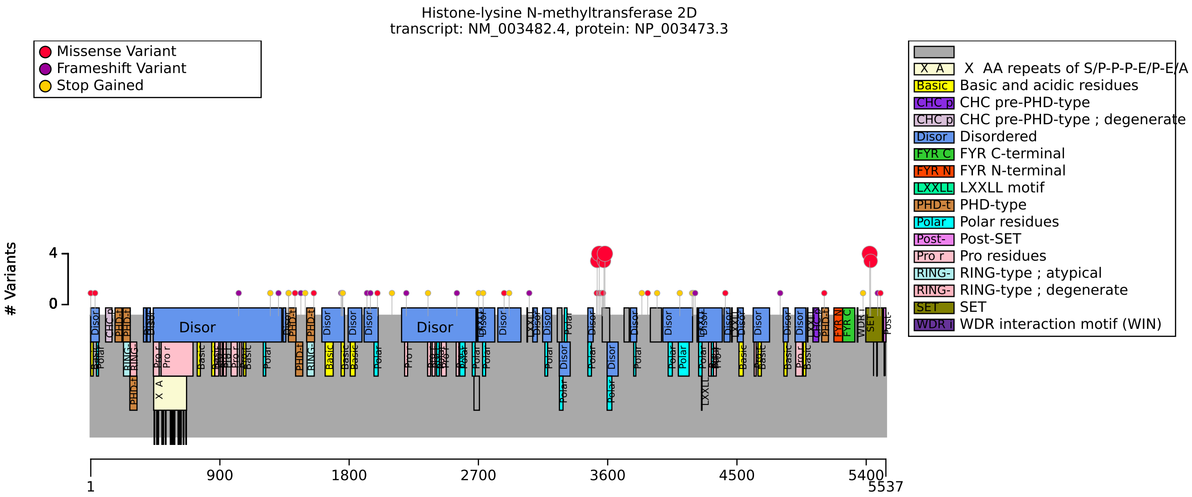

(a) Distribution of variants in *KMT2D*

Genotype (A)	Genotype (B)	total tests performed	significant results
FEMALE	MALE	42	0
missense	other	42	0
n_term	other	41	0
p.Glu5425Lys	other	36	0
p.Leu3542Pro	Other variant	8	0

(b) Fisher Exact Test performed to compare HPO annotation frequency with respect to genotypes.

Figure S32: The cohort comprised 65 individuals (35 females, 30 males). 5 of these individuals were reported to be deceased. A total of 151 HPO terms were used to annotate the cohort. Disease diagnosis: Kabuki Syndrome 1 (OMIM:147920). A total of 52 unique variant alleles were found in *KMT2D* (transcript: `NM_003482.4`, protein id: `NP_003473.3`).

LDS 1 and 2

Genotype (A)	Genotype (B)	total tests performed	significant results
OMIM:609192	OMIM:610168	36	0

(a) Fisher Exact Test performed to compare HPO annotation frequency with respect to genotypes.

Figure S33: The cohort comprised 94 individuals (30 females, 46 males, 18 with unknown sex). 6 of these individuals were reported to be deceased. A total of 110 HPO terms were used to annotate the cohort. Disease diagnoses: Loeys-Dietz syndrome 2 (OMIM:610168) (53 individuals), Loeys-Dietz syndrome 1 (OMIM:609192) (23 individuals), Multiple self-healing squamous epithelioma, susceptibility to (OMIM:132800) (18 individuals). A total of 61 unique variant alleles were found.

LDS 1 and 3

HPO term	OMIM:609192	OMIM:613795	p-value	adj. p-value
Scoliosis [HP:0002650]	18/21 (86%)	20/43 (47%)	0.003	0.027
Hypertelorism [HP:0000316]	15/19 (79%)	13/35 (37%)	0.004	0.027
Aortic aneurysm [HP:0004942]	11/11 (100%)	26/48 (54%)	0.004	0.027
Osteoarthritis [HP:0002758]	0/11 (0%)	26/38 (68%)	4.64×10^{-5}	0.001

(a) Fisher Exact Test performed to compare HPO annotation frequency with respect to OMIM:609192 and OMIM:613795. Total of 24 tests were performed.

Figure S34: The cohort comprised 90 individuals (29 females, 43 males, 18 with unknown sex). 2 of these individuals were reported to be deceased. A total of 89 HPO terms were used to annotate the cohort. Disease diagnoses: Loeys-Dietz syndrome 3 (OMIM:613795) (49 individuals), Loeys-Dietz syndrome 1 (OMIM:609192) (23 individuals), Multiple self-healing squamous epithelioma, susceptibility to (OMIM:132800) (18 individuals). A total of 37 unique variant alleles were found.

LDS 3 and 6

HPO term	OMIM:613795	OMIM:619656	p-value	adj. p-value
Thoracic aortic aneurysm [HP:0012727]	0/22 (0%)	10/16 (62%)	1.69×10^{-5}	4.23×10^{-4}

(a) Fisher Exact Test performed to compare HPO annotation frequency with respect to OMIM:613795 and OMIM:619656. Total of 25 tests were performed.

Figure S35: The cohort comprised 72 individuals (35 females, 35 males, 2 with unknown sex). A total of 98 HPO terms were used to annotate the cohort. Disease diagnoses: Loeys-Dietz syndrome 3 (OMIM:613795) (49 individuals), Loeys-Dietz syndrome 6 (OMIM:619656) (18 individuals), Congenital heart defects, multiple types, 8, with or without heterotaxy (OMIM:619657) (5 individuals). A total of 25 unique variant alleles were found.

LMNA (Part 1 of 2)

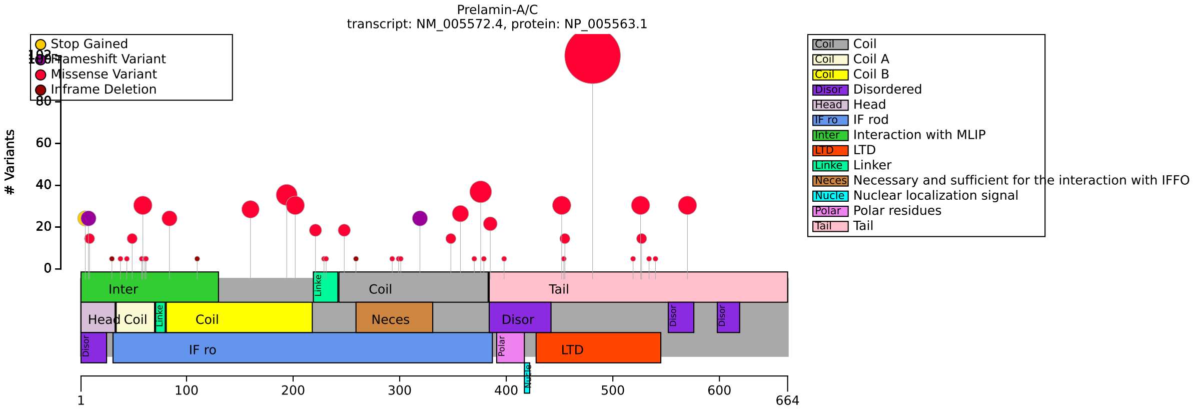

(a) Distribution of variants in *LMNA*

HPO term	missense	other	p-value	adj. p-value
Loss of truncal subcutaneous adipose tissue [HP:0009002]	104/104 (100%)	4/11 (36%)	7.53×10^{-9}	2.86×10^{-7}
Elevated hemoglobin A1c [HP:0040217]	73/101 (72%)	6/24 (25%)	3.11×10^{-5}	5.90×10^{-4}

(b) Fisher Exact Test performed to compare HPO annotation frequency with respect to missense and other. Total of 38 tests were performed.

HPO term	Upstream of NLS	other	p-value	adj. p-value
Lipodystrophy [HP:0009125]	10/93 (11%)	132/158 (84%)	1.73×10^{-31}	6.58×10^{-30}
Pancreatitis [HP:0001733]	4/7 (57%)	14/110 (13%)	0.011	0.033
Dilated cardiomyopathy [HP:0001644]	35/70 (50%)	5/102 (5%)	4.20×10^{-12}	7.98×10^{-11}
Second degree atrioventricular block [HP:0011706]	6/68 (9%)	1/115 (1%)	0.011	0.033
Atrioventricular block [HP:0001678]	17/36 (47%)	8/116 (7%)	2.11×10^{-7}	2.67×10^{-6}
Achilles tendon contracture [HP:0001771]	21/85 (25%)	19/30 (63%)	2.66×10^{-4}	0.001

(c) Fisher Exact Test: HPO annotation frequency and NLS Upstream vs other variants. 38 tests were performed in total.

Figure S36: See following page for caption

LMNA (Part 2 of 2)

HPO term	Upstream of NLS	other	p-value	adj. p-value
Foot joint contracture [HP:0008366]	21/81 (26%)	19/27 (70%)	6.20×10^{-5}	5.89×10^{-4}
Lower-limb joint contracture [HP:0005750]	22/82 (27%)	19/27 (70%)	8.20×10^{-5}	6.23×10^{-4}
Limb joint contracture [HP:0003121]	23/83 (28%)	19/27 (70%)	1.71×10^{-4}	8.14×10^{-4}
Elbow contracture [HP:0034391]	21/83 (25%)	18/26 (69%)	1.04×10^{-4}	6.59×10^{-4}
Upper-limb joint contracture [HP:0100360]	21/81 (26%)	18/26 (69%)	1.23×10^{-4}	6.66×10^{-4}
Hip contracture [HP:0003273]	4/83 (5%)	9/26 (35%)	2.68×10^{-4}	0.001
First degree atrioventricular block [HP:0011705]	7/63 (11%)	2/115 (2%)	0.010	0.033

(a) Fisher Exact Test performed to compare HPO annotation frequency with respect to NLS Upstream and other. Total of 38 tests were performed.

HPO term	Gly608=	Other	p-value	adj. p-value
Lipodystrophy [HP:0009125]	15/15 (100%)	127/236 (54%)	1.87×10^{-4}	7.47×10^{-4}
Elevated hemoglobin A1c [HP:0040217]	0/15 (0%)	79/110 (72%)	5.65×10^{-8}	6.77×10^{-7}
Muscle weakness [HP:0001324]	0/15 (0%)	63/124 (51%)	6.07×10^{-5}	3.64×10^{-4}
Proximal muscle weakness [HP:0003701]	0/15 (0%)	53/114 (46%)	3.63×10^{-4}	0.001
Distal muscle weakness [HP:0002460]	0/15 (0%)	36/97 (37%)	0.002	0.004
Proximal muscle weakness in upper limbs [HP:0008997]	0/15 (0%)	35/102 (34%)	0.005	0.007
Upper limb muscle weakness [HP:0003484]	0/15 (0%)	35/96 (36%)	0.003	0.004
Limb muscle weakness [HP:0003690]	0/15 (0%)	38/99 (38%)	0.002	0.004

(b) Fisher Exact Test performed to compare HPO annotation frequency with respect to A and B. Total of 12 tests were performed.

Description	Variable	Genotype (A)	Genotype (B)	p-value	xrefs
Phenotype score (see notebook)	HPO group count	NLS Upstream	other	1.83×10^{-16}	[35]

(c) HPO Group Count to compare NLS Upstream and other with respect to HPO group count.

Figure S37: The cohort comprised 266 individuals (150 females, 99 males, 17 with unknown sex). 11 of these individuals were reported to be deceased. A total of 152 HPO terms were used to annotate the cohort. Disease diagnoses: Lipodystrophy, familial partial, type 2 (OMIM:151660) (127 individuals), Cardiomyopathy, dilated, 1A (OMIM:115200) (68 individuals), Emery-Dreifuss muscular dystrophy 2, autosomal dominant (OMIM:181350) (41 individuals), Hutchinson-Gilford progeria (OMIM:176670) (15 individuals), *LMNA*-related congenital muscular dystrophy (OMIM:613205) (15 individuals). Numerous *LMNA* genotype-phenotype correlations have been described. The literature is summarized in [36]. Cardiac involvement in multisystem laminopathies prevails with mutations upstream of the nuclear localisation signal [35]. A total of 55 unique variant alleles were found in *LMNA* (transcript: `NM_005572.4`, protein id: `NP_005563.1`).

LZTR1

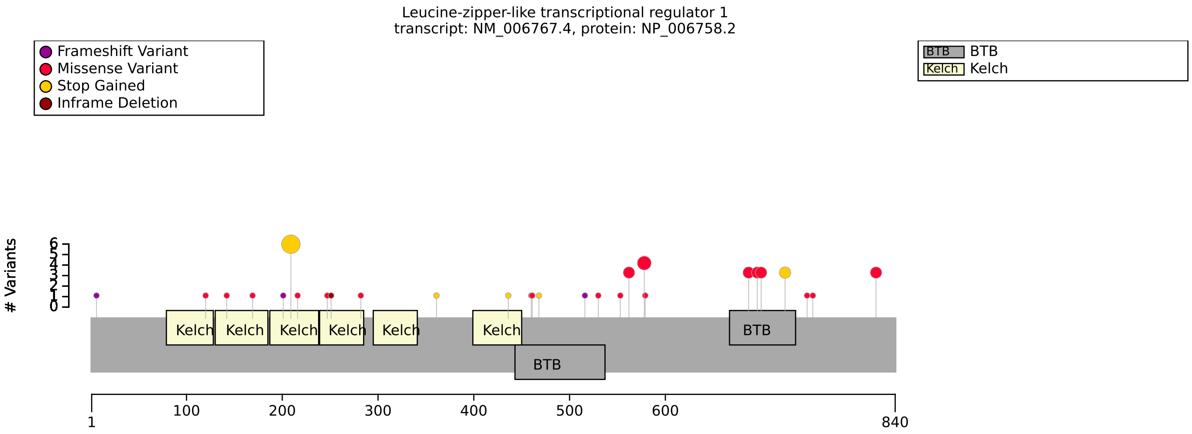

(a) Distribution of variants in *LZTR1*

Genotype (A)	Genotype (B)	total tests performed	significant results
N Term/N Term OR N Term/Other	Other/Other	49	0
Missense/Missense OR Missense/Other	Other/Other	49	0
FEMALE	MALE	54	0

(b) Fisher Exact Test performed to compare HPO annotation frequency with respect to genotypes.

Figure S38: The cohort comprised 38 individuals (18 females, 20 males). 3 of these individuals were reported to be deceased. A total of 96 HPO terms were used to annotate the cohort. Disease diagnosis: Noonan syndrome 2 (OMIM:605275). No significant association identified. A total of 38 unique variant alleles were found in *LZTR1* (transcript: `NM_006767.4`, protein id: `NP_006758.2`).

MAPK8IP3

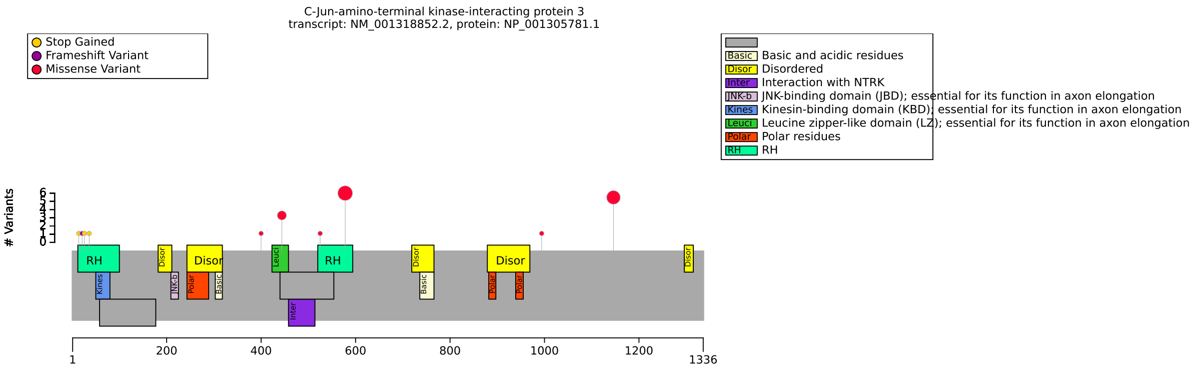

(a) Distribution of variants in *MAPK8IP3*

Genotype (A)	Genotype (B)	total tests performed	significant results
N term	other	68	0
p.Arg579Cys	Other variant	68	0
RH2	Other region	68	0
FEMALE	MALE	68	0

(b) Fisher Exact Test performed to compare HPO annotation frequency with respect to genotypes.

Figure S39: The cohort comprised 20 individuals (9 females, 11 males). A total of 93 HPO terms were used to annotate the cohort. Disease diagnosis: Neurodevelopmental disorder with or without variable brain abnormalities (OMIM:618443). No significant association identified. A total of 10 unique variant alleles were found in *MAPK8IP3* (transcript: `NM_001318852.2`, protein id: `NP_001305781.1`).

MPV17

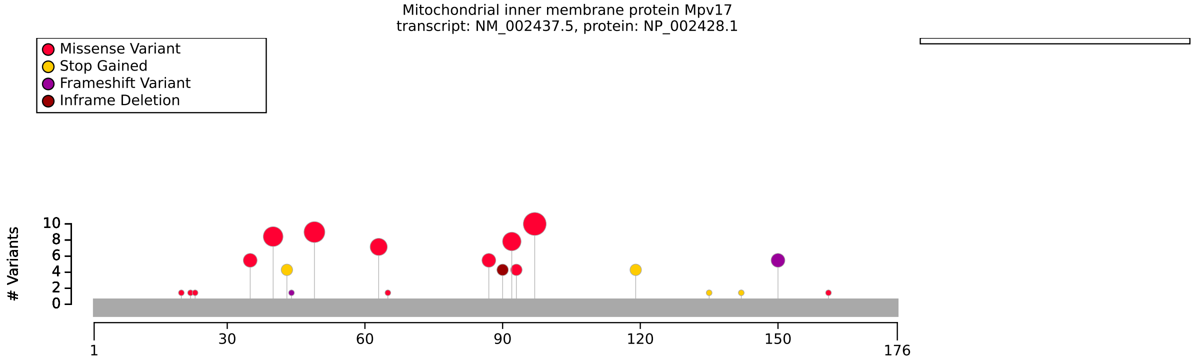

(a) Distribution of variants in *MPV17*

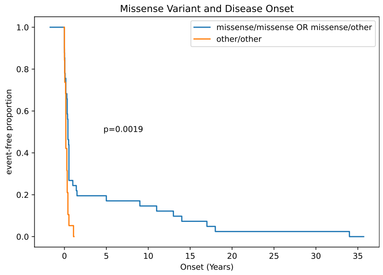

(b) *MPV17* disease onset

HPO term	Pro98Leu/Pro98Leu	other/other OR Pro98Leu/other	p-value	adj. p-value
Peripheral axonal neuropathy [HP:0003477]	3/3 (100%)	1/22 (5%)	0.002	0.037

(c) Fisher Exact Test performed to compare HPO annotation frequency with respect to Pro98Leu/Pro98Leu and other/other OR Pro98Leu/other. Total of 21 tests were performed.

Genotype (A)	Genotype (B)	total tests performed	significant results
missense/missense OR missense/other	other/other	32	0
Arg50Gln/Arg50Gln OR Arg50Gln/other	other/other	29	0
FEMALE	MALE	32	0

(d) Fisher Exact Test performed to compare HPO annotation frequency with respect to genotypes.

Description	Variable	Genotype (A)	Genotype (B)	p-value	xrefs
MTDPS6 (OMIM:256810) age at death	Age of death	Pro98Leu/Pro98Leu	other/other OR Pro98Leu/other	0.010	[37, 38]

(e) Age of death to compare Pro98Leu/Pro98Leu and other/other OR Pro98Leu/other with respect to Age of death.

Description	Variable	Genotype (A)	Genotype (B)	p-value	xrefs
MTDPS6 (OMIM:256810) disease onset	Onset of OMIM:256810	missense/missense OR missense/other	other/other	0.002	-

(f) Onset of OMIM:256810 to compare missense/missense OR missense/other and other/other with respect to Onset of OMIM:256810.

Figure S40: The cohort comprised 60 individuals (30 females, 30 males). 39 of these individuals were reported to be deceased. A total of 156 HPO terms were used to annotate the cohort. Disease diagnosis: Mitochondrial DNA depletion syndrome 6 (hepatocerebral type) (MTDPS6; OMIM:256810). No clear genotype-phenotype correlation exists. However, a trend for longer survival can be observed in individuals with biallelic pathogenic missense variants compared to individuals with biallelic null [37, 38]. A total of 31 unique variant alleles were found in *MPV17* (transcript: `NM_002437.5`, protein id: `NP_002428.1`).

NBAS

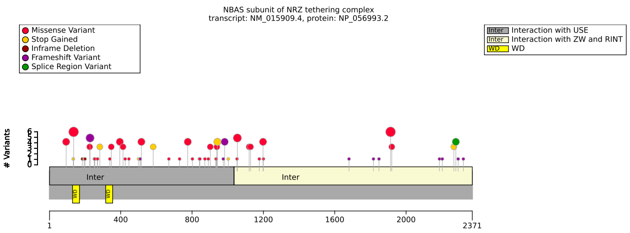

(a) Distribution of variants in *NBAS*

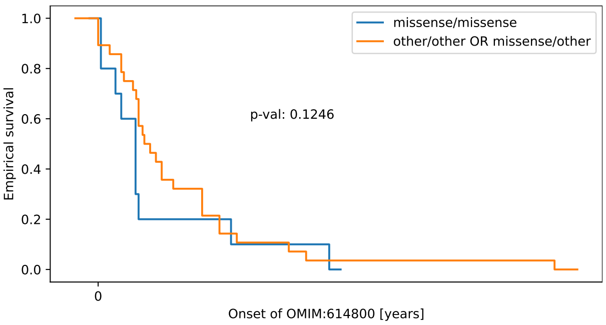

(b) *NBAS* missense variants vs. age at death.

HPO term	missense/missense	other/other OR missense/other	p-value	adj. p-value
Decreased circulating IgG concentration [HP:0004315]	1/12 (8%)	15/23 (65%)	0.002	0.035

(c) Fisher Exact Test performed to compare HPO annotation frequency with respect to missense/missense and other/other OR missense/other. Total of 22 tests were performed.

Description	Variable	Genotype (A)	Genotype (B)	p-value	xrefs
Age of death	Age of death	missense/missense	other/other OR missense/other	0.125	-

(d) Age of death to compare missense/missense and other/other OR missense/other with respect to Age of death.

Figure S41: The cohort comprised 67 individuals (19 females, 21 males, 27 with unknown sex). 9 of these individuals were reported to be deceased. A total of 134 HPO terms were used to annotate the cohort. Disease diagnosis: Short stature, optic nerve atrophy, and Pelger-Huet anomaly (OMIM:614800). It was reported that missense or in-frame deletions in the C-terminal region of *NBAS* are associated with SOPH syndrome (614800), missense or in-frame deletions in the Sec30 domain of *NBAS* are associated with infantile liver failure syndrome type 2 (616483), while missense or in-frame deletions in the beta-propeller domain of *NBAS* are associated with a combined phenotype of multisystem involvement with acute liver failure [39]. In our dataset, we found an association with missense or in-frame deletions in the Sec30 with decreased circulating IgG. A similar finding was obtain for missense variants in general. Due to the small size of our cohort, this finding should be regarded as preliminary. A total of 74 unique variant alleles were found in *NBAS* (transcript: `NM_015909.4`, protein id: `NP_056993.2`).

NF1

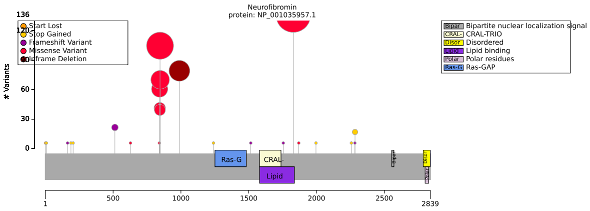

(a) Distribution of variants in *NF1*

HPO term	Missense	other	p-value	adj. p-value
Freckling [HP:0001480]	181/278 (65%)	96/117 (82%)	7.20×10^{-4}	0.009
Glioma [HP:0009733]	35/282 (12%)	11/27 (41%)	5.31×10^{-4}	0.009
Lipoma [HP:0012032]	4/277 (1%)	5/51 (10%)	0.006	0.030
Spinal neurofibroma [HP:0009735]	23/228 (10%)	0/67 (0%)	0.003	0.021
Neurofibroma by anatomical location [HP:0034761]	23/206 (11%)	0/67 (0%)	0.002	0.015

(b) Fisher Exact Test performed to compare HPO annotation frequency with respect to Missense and other. Total of 26 tests were performed.

HPO term	p.Arg1830	other	p-value	adj. p-value
Freckling [HP:0001480]	76/133 (57%)	201/262 (77%)	1.11×10^{-4}	2.99×10^{-4}
Lisch nodules [HP:0009737]	11/91 (12%)	72/193 (37%)	6.58×10^{-6}	1.97×10^{-5}
Neurofibroma [HP:0001067]	5/133 (4%)	153/275 (56%)	2.48×10^{-28}	6.71×10^{-27}
Axillary freckling [HP:0000997]	20/79 (25%)	105/175 (60%)	3.66×10^{-7}	1.41×10^{-6}
Inguinal freckling [HP:0030052]	8/79 (10%)	84/170 (49%)	4.86×10^{-10}	3.28×10^{-9}
Plexiform neurofibroma [HP:0009732]	0/135 (0%)	68/255 (27%)	1.45×10^{-14}	1.96×10^{-13}
Scoliosis [HP:0002650]	8/125 (6%)	48/240 (20%)	4.02×10^{-4}	9.87×10^{-4}
Optic nerve glioma [HP:0009734]	0/135 (0%)	39/217 (18%)	1.92×10^{-9}	1.04×10^{-8}
Glioma [HP:0009733]	2/136 (1%)	44/173 (25%)	2.36×10^{-10}	2.13×10^{-9}
Spinal neurofibroma [HP:0009735]	0/128 (0%)	23/167 (14%)	1.90×10^{-6}	6.42×10^{-6}
Neurofibroma by anatomical location [HP:0034761]	0/128 (0%)	23/145 (16%)	2.05×10^{-7}	9.23×10^{-7}
Pulmonic stenosis [HP:0001642]	13/105 (12%)	5/162 (3%)	0.005	0.011

(c) Fisher Exact Test performed to compare HPO annotation frequency with respect to p.Arg1830 and other. Total of 27 tests were performed.

HPO term	Met992del	other	p-value	adj. p-value
Lisch nodules [HP:0009737]	3/36 (8%)	80/248 (32%)	0.003	0.008
Neurofibroma [HP:0001067]	0/44 (0%)	158/364 (43%)	1.68×10^{-10}	2.68×10^{-9}
Axillary freckling [HP:0000997]	0/14 (0%)	125/240 (52%)	8.57×10^{-5}	6.86×10^{-4}
Inguinal freckling [HP:0030052]	0/14 (0%)	92/235 (39%)	0.003	0.008
Plexiform neurofibroma [HP:0009732]	0/44 (0%)	68/346 (20%)	2.00×10^{-4}	0.001
Lipoma [HP:0012032]	5/44 (11%)	4/284 (1%)	0.003	0.008

(d) Fisher Exact Test performed to compare HPO annotation frequency with respect to Met992del and other. Total of 16 tests were performed.

NF1 (part 2)

HPO term	Leu847Pro	other	p-value	adj. p-value
Freckling [HP:0001480]	54/64 (84%)	223/331 (67%)	0.007	0.023
Lisch nodules [HP:0009737]	22/42 (52%)	61/242 (25%)	7.48×10^{-4}	0.004
Neurofibroma [HP:0001067]	47/67 (70%)	111/341 (33%)	1.58×10^{-8}	2.14×10^{-7}
Axillary freckling [HP:0000997]	50/61 (82%)	75/193 (39%)	3.55×10^{-9}	9.58×10^{-8}
Inguinal freckling [HP:0030052]	34/60 (57%)	58/189 (31%)	3.92×10^{-4}	0.003
Plexiform neurofibroma [HP:0009732]	24/65 (37%)	44/325 (14%)	4.45×10^{-5}	4.00×10^{-4}
Optic nerve glioma [HP:0009734]	15/65 (23%)	24/287 (8%)	0.002	0.008
Glioma [HP:0009733]	18/67 (27%)	28/242 (12%)	0.003	0.013
Neurofibroma by anatomical location [HP:0034761]	6/26 (23%)	17/247 (7%)	0.014	0.041

(a) Fisher Exact Test performed to compare HPO annotation frequency with respect to Leu847Pro and other. Total of 27 tests were performed.

HPO term	SV	other	p-value	adj. p-value
Freckling [HP:0001480]	53/60 (88%)	224/335 (67%)	6.36×10^{-4}	0.005
Neurofibroma [HP:0001067]	41/60 (68%)	117/348 (34%)	6.03×10^{-7}	1.39×10^{-5}
Plexiform neurofibroma [HP:0009732]	16/50 (32%)	52/340 (15%)	0.008	0.037
Scoliosis [HP:0002650]	18/57 (32%)	38/308 (12%)	9.71×10^{-4}	0.006
Glioma [HP:0009733]	8/11 (73%)	38/298 (13%)	1.60×10^{-5}	1.84×10^{-4}

(b) Fisher Exact Test performed to compare HPO annotation frequency with respect to SV and other. Total of 23 tests were performed.

Figure S43: The cohort comprised 419 individuals (192 females, 169 males, 58 with unknown sex). A total of 36 HPO terms were used to annotate the cohort. Disease diagnosis: Neurofibromatosis, type 1 (OMIM:162200). A substantial body of literature exists about genotype-phenotype correlations. The correlations described in this notebook have been previously reported [40, 41, 42]. A total of 36 unique variant alleles were found in *NF1* (transcript: `NM_001042492.3`, protein id: `NP_001035957.1`).

NIPBL

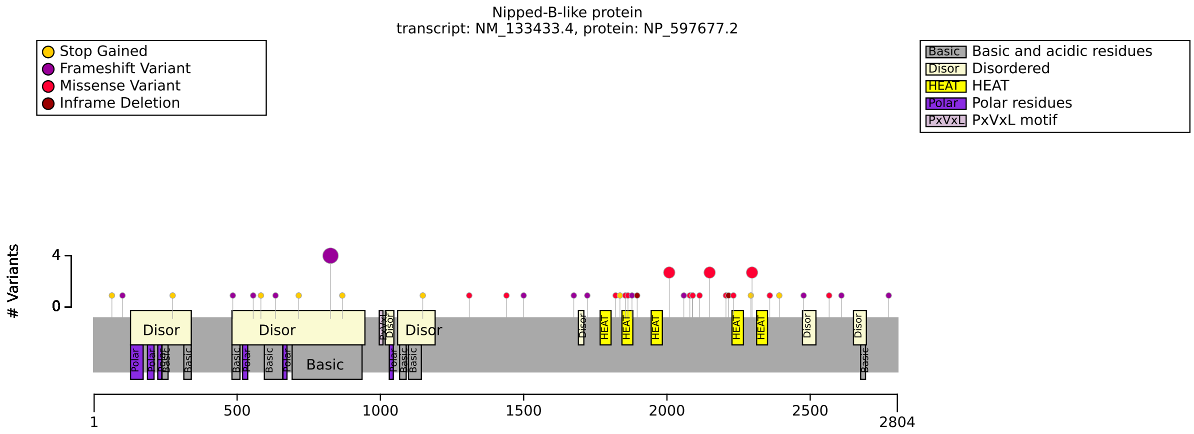

(a) Distribution of variants in *NIPBL*

Genotype (A)	Genotype (B)	total tests performed	significant results
missense	other	86	0
disordered	other	86	0
FEMALE	MALE	81	0

(b) Fisher Exact Test performed to compare HPO annotation frequency with respect to genotypes.

Figure S44: The cohort comprised 60 individuals (21 females, 31 males, 8 with unknown sex). A total of 90 HPO terms were used to annotate the cohort. Disease diagnosis: Cornelia de Lange syndrome 1 (OMIM:122470). No statistically significant genotype-phenotype correlation identified. A total of 50 unique variant alleles were found in *NIPBL* (transcript: `NM_133433.4`, protein id: `NP_597677.2`).

NKX6-2

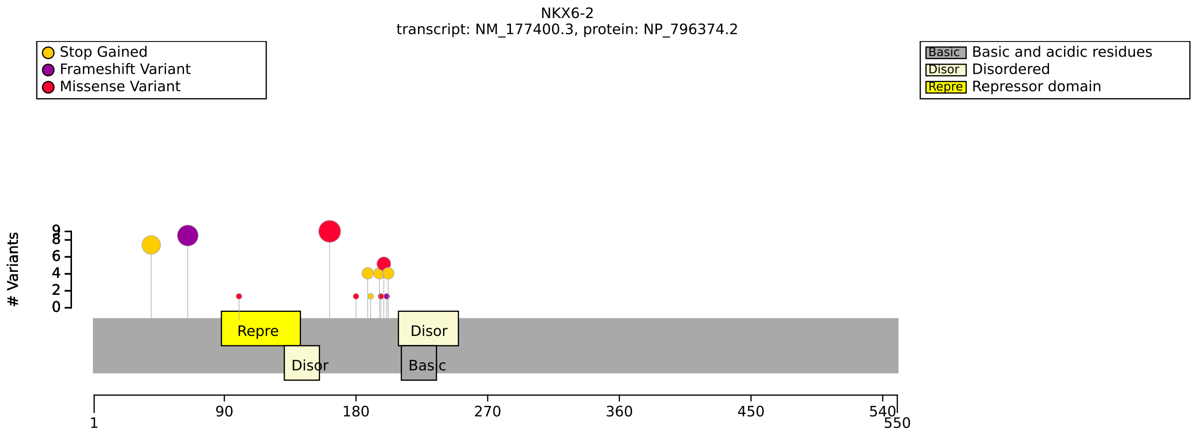

(a) Distribution of variants in *NKX6-2*

Genotype (A)	Genotype (B)	total tests performed	significant results
missense/missense	other/other OR missense/other	21	0
L163V/L163V	L163V/other OR other/other	21	0
N term/N term OR N term/other	other/other	21	0
FEMALE	MALE	21	0

(b) Fisher Exact Test performed to compare HPO annotation frequency with respect to genotypes.

Figure S45: The cohort comprised 33 individuals (15 females, 18 males). A total of 39 HPO terms were used to annotate the cohort. Disease diagnosis: Spastic ataxia 8, autosomal recessive, with hypomyelinating leukodystrophy (OMIM:617560). No statistically significant results identified. A total of 13 unique variant alleles were found in *NKX6-2* (transcript: `NM_177400.3`, protein id: `NP_796374.2`).

PIGA

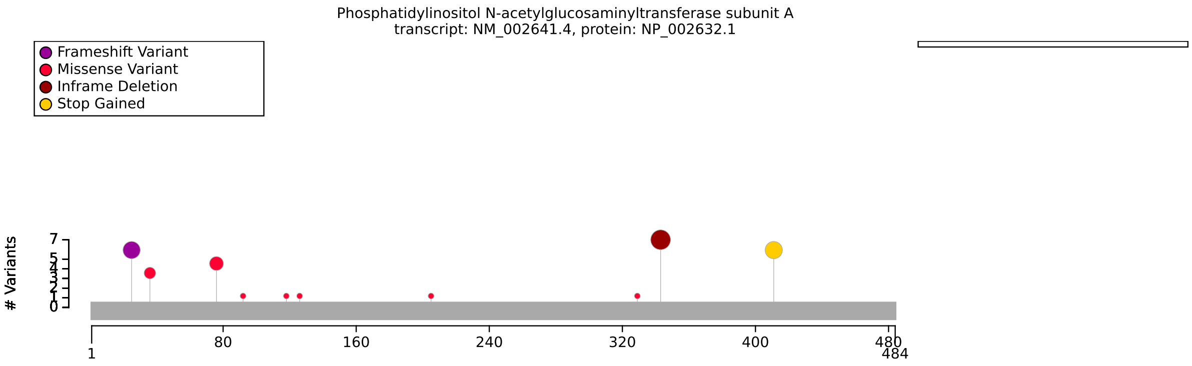

(a) Distribution of variants in *PIGA*

Genotype (A)	Genotype (B)	total tests performed	significant results
missense	other	23	0
p.Leu344del	other	20	0
1-100	100+	23	0

(b) Fisher Exact Test performed to compare HPO annotation frequency with respect to missense, p.Leu344del, and N-terminal (1-100) and other.

Figure S46: The cohort comprised 27 individuals (0 females, 27 males). A total of 188 HPO terms were used to annotate the cohort. Disease diagnoses: Multiple congenital anomalies-hypotonia-seizures syndrome 2 (OMIM:300868) (21 individuals), Neurodevelopmental disorder with epilepsy and hemochromatosis (OMIM:301072) (6 individuals). No statistically significant results identified. A total of 12 unique variant alleles were found in *PIGA* (transcript: `NM_002641.4`, protein id: `NP_002632.1`).

POGZ

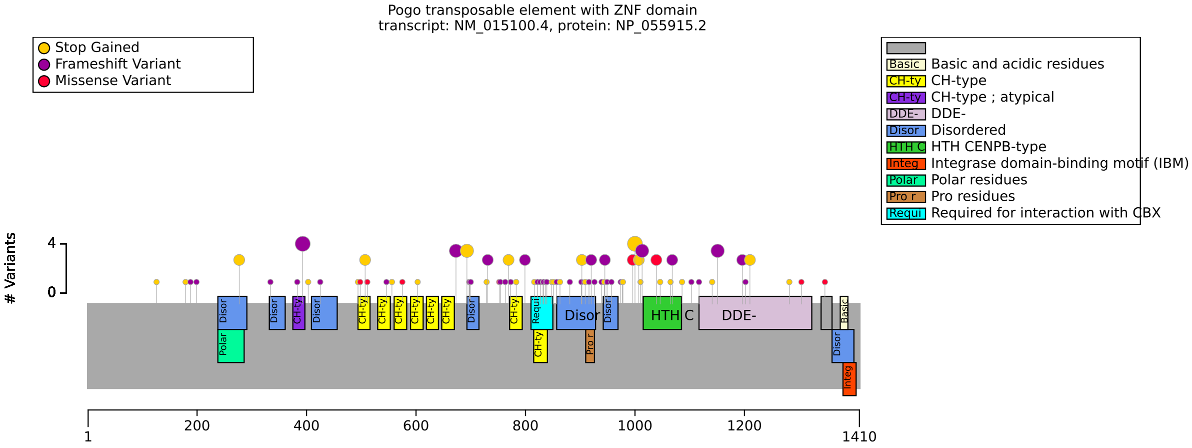

(a) Distribution of variants in *POGZ*

Genotype (A)	Genotype (B)	total tests performed	significant results
Missense	Other	24	0
Frameshift	Other	24	0

(b) Fisher Exact Test performed to compare HPO annotation frequency with respect to genotypes.

Description	Variable	Genotype (A)	Genotype (B)	p-value	xrefs
A phenotypic severity score according to Nagy et al. 2022	*POGZ* Severity Score	Missense	Other	0.429	-

(c) POGZ Severity Score to compare Missense and Other with respect to *POGZ* Severity Score.

Description	Variable	Genotype (A)	Genotype (B)	p-value	xrefs
A phenotypic severity score for individuals with intellectual disability	De Vries score	Missense	Other	0.429	-

(d) De Vries Score to compare Missense and Other with respect to De Vries score.

Figure S47: The cohort comprised 117 individuals (54 females, 62 males, 1 with unknown sex). A total of 94 HPO terms were used to annotate the cohort. Disease diagnosis: White-Sutton syndrome (OMIM:616364). Negy et al. (2022) summarized data on 117 individuals with White-Sutton syndrome. They identified a correlation between a severity score and nonsense-mediated RNA decay (NMD). Missense variants were more often associated with mild phenotypes and truncating variants predicted to escape NMD presented with more severe phenotypes. Within this group, variants in the proline-rich region of the POGZ protein were associated with the most severe phenotypes. These authors did not apply multiple testing correction [43]. Our analysis did not identify a significant GPC. A total of 90 unique variant alleles were found in *POGZ* (transcript: `NM_015100.4`, protein id: `NP_055915.2`).

PPP2R1A

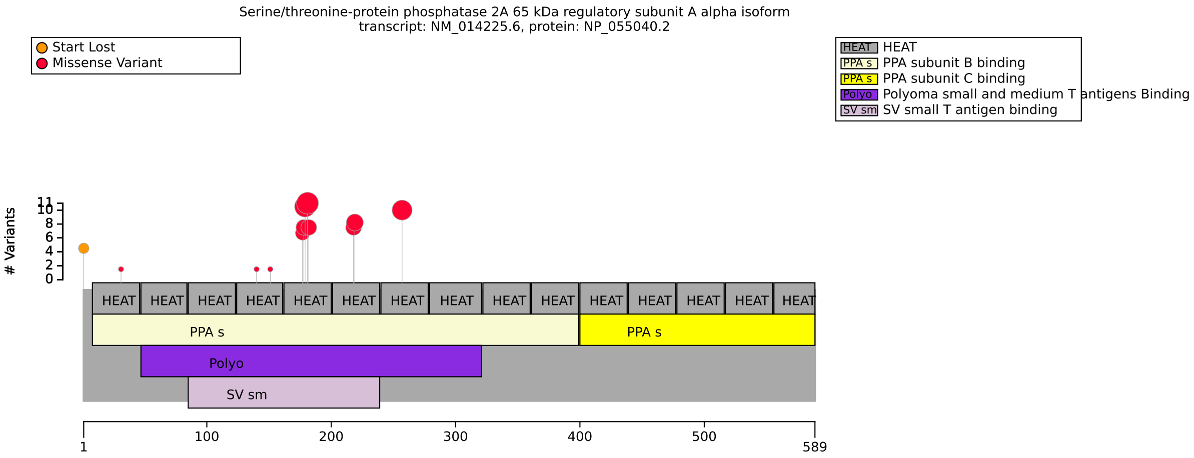

(a) Distribution of variants in *PPP2R1A*

Genotype (A)	Genotype (B)	total tests performed	significant results
Absent in COSMIC	Present in COSMIC	18	0
Arg182Trp	other	19	0
SV40 small T antigen binding	other	19	0

(b) Fisher Exact Test performed to compare HPO annotation frequency with respect to genotypes.

Figure S48: The cohort comprised 60 individuals (24 females, 32 males, 4 with unknown sex). A total of 52 HPO terms were used to annotate the cohort. Disease diagnosis: Houge-Janssen syndrome 2 (OMIM:616362). No statistically significant results identified. A total of 20 unique variant alleles were found in *PPP2R1A* (transcript: `NM_014225.6`, protein id: `NP_055040.2`).

PTPN11

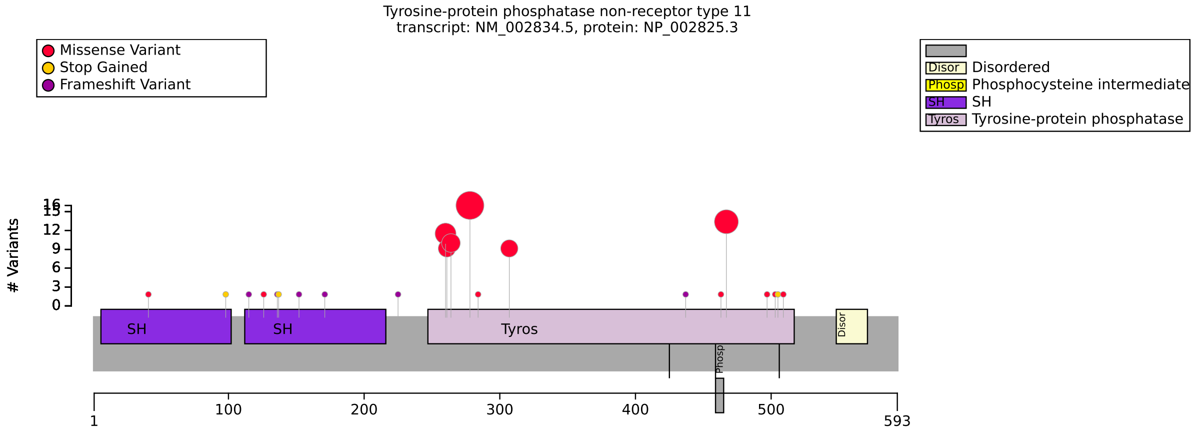

(a) Distribution of variants in *PTPN11*

HPO term	Missense	Other	p-value	adj. p-value
Hypertelorism [HP:0000316]	37/41 (90%)	0/12 (0%)	6.82×10^{-9}	2.73×10^{-8}
Intellectual disability, mild [HP:0001256]	8/23 (35%)	0/12 (0%)	0.032	0.032
Pulmonic stenosis [HP:0001642]	18/34 (53%)	0/12 (0%)	0.001	0.002
Webbed neck [HP:0000465]	15/20 (75%)	0/12 (0%)	2.94×10^{-5}	5.88×10^{-5}

(b) Fisher Exact Test performed to compare HPO annotation frequency with respect to Missense and Other. Total of 4 tests were performed.

Genotype (A)	Genotype (B)	total tests performed	significant results
Tyr279Cys	Other	8	0
TK domain N term	TK domain C term	5	0
FEMALE	MALE	6	0

(c) Fisher Exact Test performed to compare HPO annotation frequency with respect to genotypes.

Figure S49: The cohort comprised 70 individuals (27 females, 33 males, 10 with unknown sex). A total of 69 HPO terms were used to annotate the cohort. Disease diagnoses: LEOPARD syndrome 1 (OMIM:151100) (31 individuals), Noonan syndrome 1 (OMIM:163950) (27 individuals), Metachondromatosis (OMIM:156250) (12 individuals). No previous statistical analysis of correlations with *PTPN11* missense variants was identified in the medical literature. A total of 27 unique variant alleles were found in *PTPN11* (transcript: `NM_002834.5`, protein id: `NP_002825.3`).

RERE

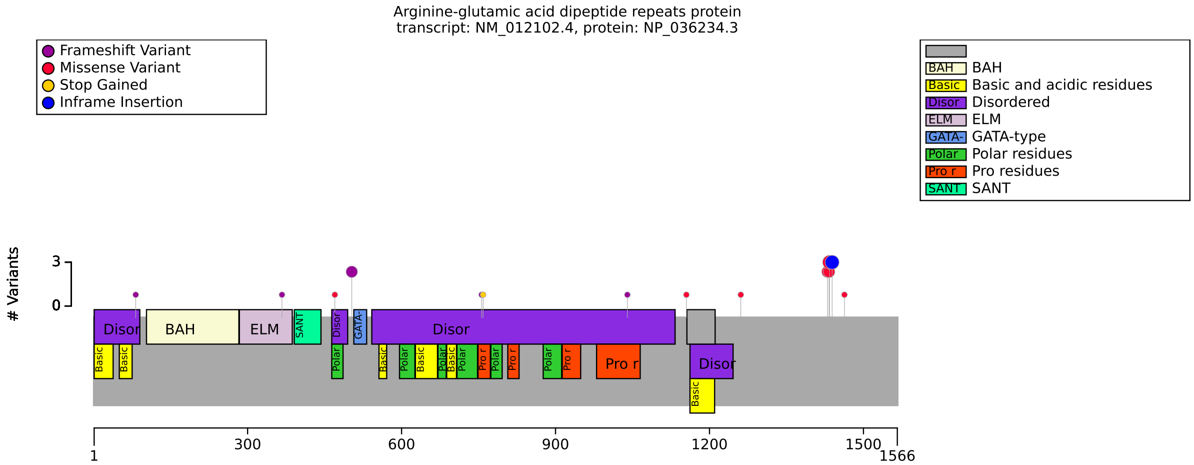

(a) Distribution of variants in *RERE*

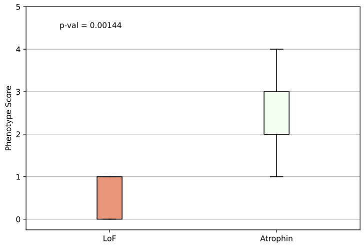

(b) Phenotype score adapted from Jordan et al. [44]. Variants in atrophin domain (residues 1425-1445) vs. others. Mann-Whitney U test.

Genotype (A)	Genotype (B)	total tests performed	significant results
LoF	Atrophin	56	0

(c) Fisher Exact Test performed to compare HPO annotation frequency with respect to genotypes.

Description	Variable	Genotype (A)	Genotype (B)	p-value	xrefs
Phenotype score (see notebook)	HPO group count	LoF	Atrophin	0.001	[44]

(d) HPO Group Count to compare LoF and Atrophin with respect to HPO group count (HP:0012443, HP:0012372,HP:0001627,HP:0012210, HP:0000407).

Figure S50: The cohort comprised 22 individuals (9 females, 13 males). A total of 115 HPO terms were used to annotate the cohort. Disease diagnosis: Neurodevelopmental disorder with or without anomalies of the brain, eye, or heart (OMIM:616975). Our results recapitulate the results of Jordan et al. [44] that The total number of structural defects and sensorineural hearing loss diagnoses seen in individuals with point mutations in the Atrophin-1 domain is significantly higher than expected based on the number of similar defects seen in individuals with putative loss-of-function variants. A total of 18 unique variant alleles were found in *RERE* (transcript: `NM_012102.4`, protein id: `NP_036234.3`).

RNU4-2

Genotype (A)	Genotype (B)	total tests performed	significant results
n.64_65insT	other	176	0
insertion	other	176	0
FEMALE	MALE	176	0

(a) Fisher Exact Test performed to compare HPO annotation frequency with respect to genotypes.

Figure S51: The cohort comprised 61 individuals (28 females, 33 males). A total of 209 HPO terms were used to annotate the cohort. Disease diagnosis: ReNU syndrome (OMIM:620851). No previous statistical analysis of correlations with *RNU4-2* missense variants identified in the medical literature. A total of 7 unique variant alleles were found in *RNU4-2* (transcript: `NR_003137.3`, protein id:).

Robinow syndrome

HPO term	OMIM:268310	OMIM:616331	p-value	adj. p-value
Mesomelia [HP:0003027]	31/31 (100%)	10/15 (67%)	0.002	0.048
Hearing impairment [HP:0000365]	3/22 (14%)	7/7 (100%)	7.69×10^{-5}	0.003
Short stature [HP:0004322]	29/29 (100%)	3/11 (27%)	2.15×10^{-6}	1.89×10^{-4}
Cleft palate [HP:0000175]	0/17 (0%)	5/8 (62%)	0.001	0.031

(a) Fisher Exact Test performed to compare HPO annotation frequency with respect to Robinow syndrome, autosomal recessive (OMIM:268310) and Robinow syndrome, autosomal dominant 2 (OMIM:616331). Total of 88 tests were performed.

Figure S52: The cohort comprised 48 individuals (10 females, 14 males, 24 with unknown sex). A total of 103 HPO terms were used to annotate the cohort. Disease diagnoses: Robinow syndrome, autosomal recessive (OMIM:268310) (32 individuals), Robinow syndrome, autosomal dominant 2 (OMIM:616331) (16 individuals). Robinow syndrome is a skeletal dysplasia characterized by dysmorphic facial features, short-limbed dwarfism, vertebral segmentation, and genital hypoplasia. A total of 44 unique variant alleles were found.

ROR2

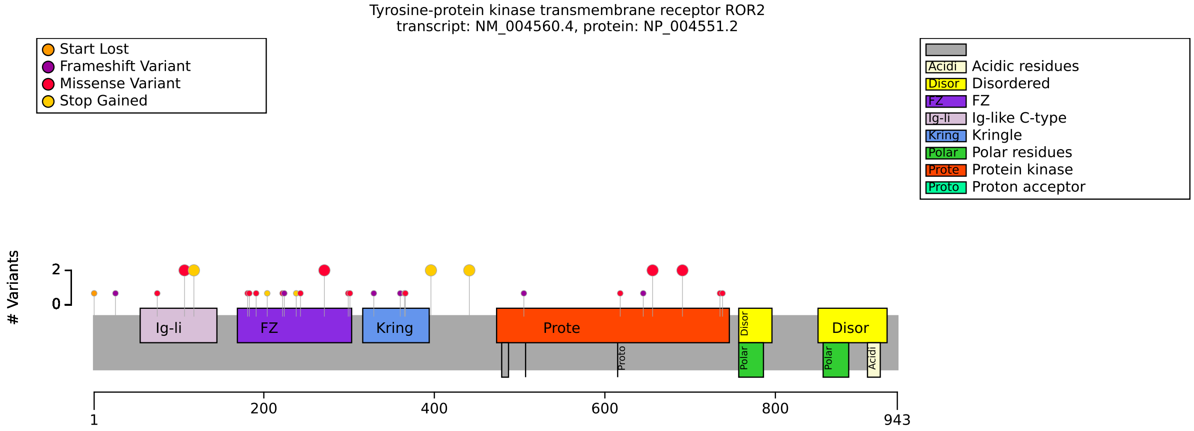

(a) Distribution of variants in *ROR2*

Genotype (A)	Genotype (B)	total tests performed	significant results
missense/missense OR missense/other	other/other	116	0
FZ domain/FZ domain OR FZ domain/other	other/other	116	0

(b) Fisher Exact Test performed to compare HPO annotation frequency with respect to genotypes.

Figure S53: The cohort comprised 32 individuals (3 females, 7 males, 22 with unknown sex). A total of 81 HPO terms were used to annotate the cohort. Disease diagnosis: Robinow syndrome, autosomal recessive (OMIM:268310). No statistically significant genotype-phenotype correlation was identified. A total of 32 unique variant alleles were found in *ROR2* (transcript: `NM_004560.4`, protein id: `NP_004551.2`).

RPGRIP1

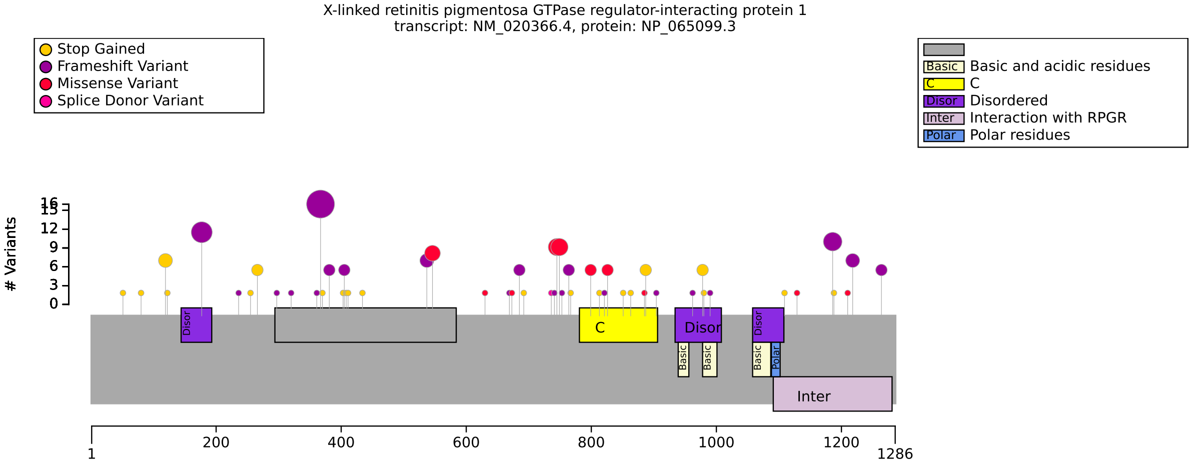

(a) Distribution of variants in *RPGRIP1*

HPO term	1107del/1107del OR 1107del/other	other/other	p-value	adj. p-value
Eye poking [HP:0001483]	16/16 (100%)	19/41 (46%)	1.30×10^{-4}	0.002

(b) Fisher Exact Test performed to compare HPO annotation frequency with respect to 1107del/1107del OR 1107del/other and other/other. Total of 16 tests were performed.

HPO term	OMIM:613826	OMIM:608194	p-value	adj. p-value
Nystagmus [HP:0000639]	64/66 (97%)	11/16 (69%)	0.003	0.020
Very low visual acuity [HP:0032122]	35/39 (90%)	4/16 (25%)	5.21×10^{-6}	7.81×10^{-5}

(c) Fisher Exact Test performed to compare HPO annotation frequency with respect to OMIM:613826 and OMIM:608194. Total of 15 tests were performed.

Genotype (A)	Genotype (B)	total tests performed	significant results
missense/missense	missense/other OR other/other	17	0
frameshift/frameshift	frameshift/other OR other/other	16	0
FEMALE	MALE	16	0

(d) Fisher Exact Test performed to compare HPO annotation frequency with respect to genotypes.

Figure S54: The cohort comprised 100 individuals (45 females, 49 males, 6 with unknown sex). A total of 45 HPO terms were used to annotate the cohort. Disease diagnoses: Leber congenital amaurosis 6 (OMIM:613826) (79 individuals), Cone-rod dystrophy 13 (OMIM:608194) (21 individuals). It was reported that Patients with a double null genotype may develop symptoms earlier and have worse vision [45]. We did not observe a corresponding significant correlation in our dataset. A total of 71 unique variant alleles were found in *RPGRIP1* (transcript: `NM_020366.4`, protein id: `NP_065099.3`).

SAMD9L

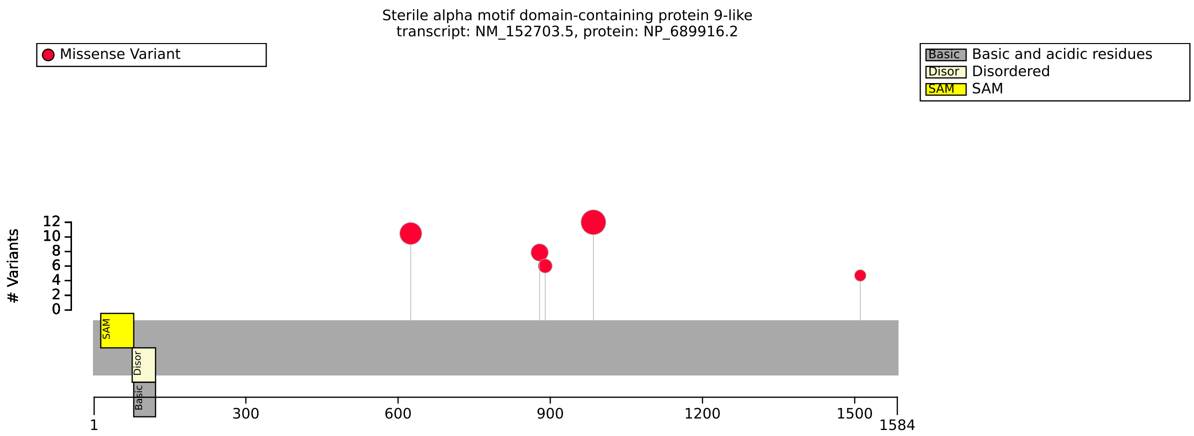

(a) Distribution of variants in *SAMD9L*

HPO term	Arg986Cys	Ser626Leu	p-value	adj. p-value
Pancytopenia [HP:0001876]	4/6 (67%)	0/9 (0%)	0.011	0.026
Thrombocytopenia [HP:0001873]	7/9 (78%)	0/9 (0%)	0.002	0.008
Neutropenia [HP:0001875]	7/9 (78%)	0/9 (0%)	0.002	0.008

(b) Fisher Exact Test performed to compare HPO annotation frequency with respect to Arg986Cys and Ser626Leu. Total of 7 tests were performed.

Genotype (A)	Genotype (B)	total tests performed	significant results
N Term	other	16	0

(c) Fisher Exact Test performed to compare HPO annotation frequency with respect to genotypes.

Figure S55: The cohort comprised 31 individuals (15 females, 16 males). 5 of these individuals were reported to be deceased. A total of 41 HPO terms were used to annotate the cohort. Disease diagnoses: Ataxia-pancytopenia syndrome (OMIM:159550) (22 individuals), Spinocerebellar ataxia 49 (OMIM:619806) (9 individuals). A recent summary of *SAMD9L* variants stated there was no evidence of phenotype–genotype correlation [46]. A total of 6 unique variant alleles were found in *SAMD9L* (transcript: `NM_152703.5`, protein id: `NP_689916.2`).

SATB2

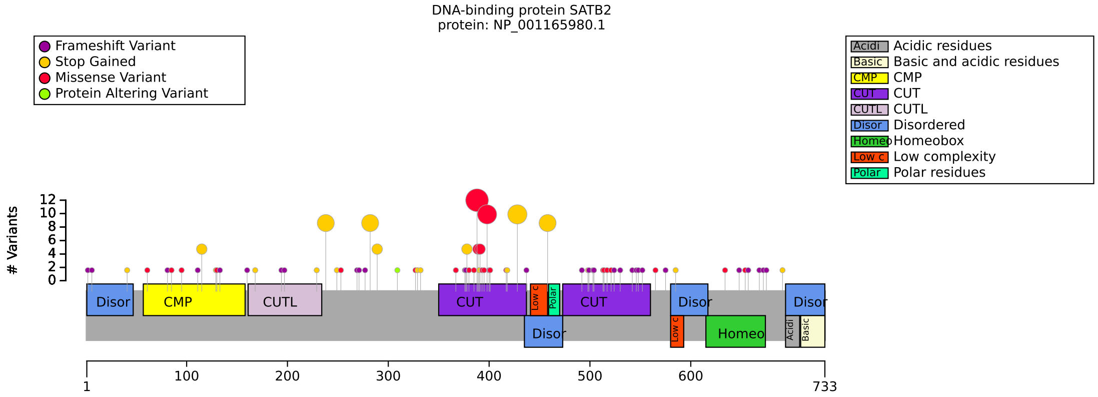

(a) Distribution of variants in *SATB2*

HPO term	Missense	Other	p-value	adj. p-value
Cleft palate [HP:0000175]	11/49 (22%)	59/105 (56%)	1.11×10^{-4}	0.002

(b) Fisher Exact Test performed to compare HPO annotation frequency with respect to Missense and Other. Total of 20 tests were performed.

Genotype (A)	Genotype (B)	total tests performed	significant results
ULD	Other	20	0
FEMALE	MALE	20	0
transcript ablation	other	20	0

(c) Fisher Exact Test performed to compare HPO annotation frequency with respect to genotypes.

Figure S56: The cohort comprised 158 individuals (62 females, 90 males, 6 with unknown sex). A total of 11 HPO terms were used to annotate the cohort. Disease diagnosis: Glass syndrome (OMIM:612313). Individuals with large chromosomal deletions were diagnosed at earlier ages (mean 2.5 years, $p \leq 0.0006$). Individuals with missense or disruptive pathogenic variants were more commonly reported to have sialorrhea (p = 0.0115), and those with large deletions were more likely to have a history of growth retardation (p = 0.0033). The authors reported a higher prevalence of chronic mucocutanous candidiasis with the variant Arg357Ter than with other variants. We did not identify a significant difference in prevalence [47]. A total of 92 unique variant alleles were found in *SATB2* (transcript: `NM_001172509.2`, protein id: `NP_001165980.1`).

SCN2A

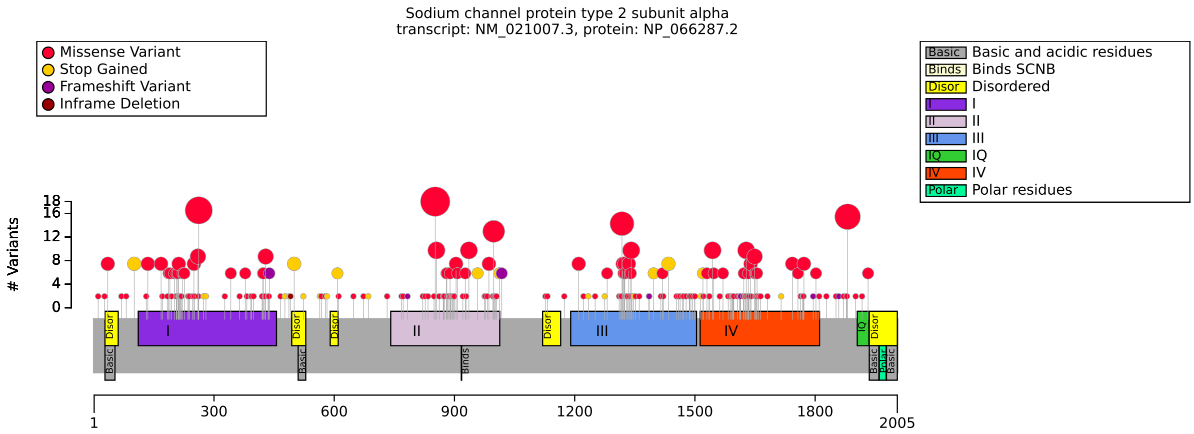

(a) Distribution of variants in *SCN2A*

HPO term	Missense	Other	p-value	adj. p-value
Neurodevelopmental abnormality [HP:0012759]	201/238 (84%)	45/45 (100%)	0.001	0.003
Motor seizure [HP:0020219]	146/175 (83%)	6/31 (19%)	4.51×10^{-12}	7.22×10^{-11}
Seizure [HP:0001250]	298/327 (91%)	28/53 (53%)	1.57×10^{-10}	8.38×10^{-10}
Autism [HP:0000717]	59/146 (40%)	33/43 (77%)	2.69×10^{-5}	8.61×10^{-5}
Focal-onset seizure [HP:0007359]	141/170 (83%)	8/33 (24%)	8.66×10^{-11}	6.93×10^{-10}
Generalized-onset seizure [HP:0002197]	104/133 (78%)	6/31 (19%)	1.43×10^{-9}	5.72×10^{-9}
Intellectual disability [HP:0001249]	144/198 (73%)	34/34 (100%)	9.39×10^{-5}	2.50×10^{-4}

(b) Fisher Exact Test performed to compare HPO annotation frequency with respect to Missense and Other. Total of 16 tests were performed.

HPO term	I repeat	Other	p-value	adj. p-value
Neurodevelopmental abnormality [HP:0012759]	48/65 (74%)	198/218 (91%)	0.001	0.009
Intellectual disability [HP:0001249]	21/42 (50%)	157/190 (83%)	2.59×10^{-5}	4.15×10^{-4}

(c) Fisher Exact Test performed to compare HPO annotation frequency with respect to I repeat and Other. Total of 16 tests were performed.

Genotype (A)	Genotype (B)	total tests performed	significant results
Arg853Gln	Other	16	0
Exon 27	Other	16	0

(d) Fisher Exact Test performed to compare HPO annotation frequency with respect to genotypes.

Figure S57: The cohort comprised 393 individuals (0 females, 0 males, 393 with unknown sex). A total of 289 HPO terms were used to annotate the cohort. Disease diagnoses: Developmental and epileptic encephalopathy 11 (OMIM:613721) (342 individuals), Seizures, benign familial infantile, 3 (OMIM:607745) (51 individuals). Similar genotype-phenotype correlations have been previously published [48]. A total of 264 unique variant alleles were found in *SCN2A* (transcript: `NM_021007.3`, protein id: `NP_066287.2`).

SCO2

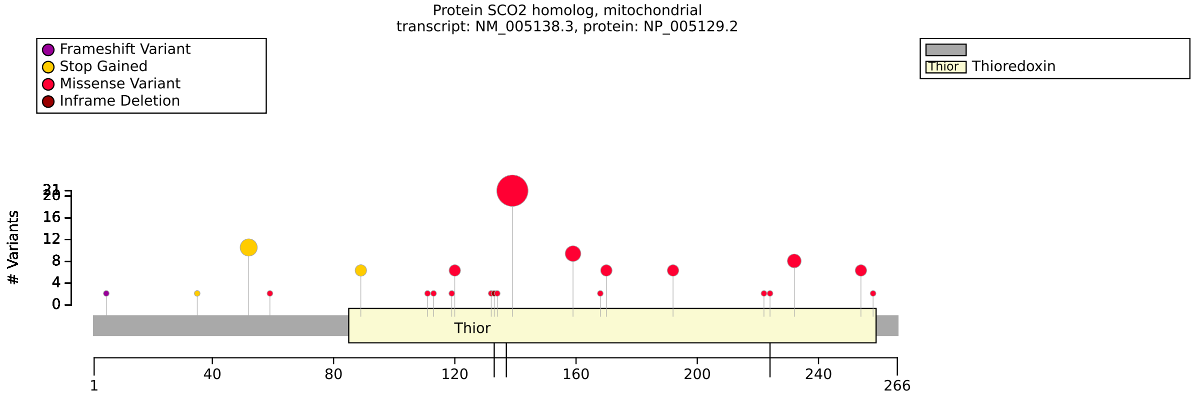

(a) Distribution of variants in *SCO2*

HPO term	Glu140Lys/Glu140Lys	other/other OR Glu140Lys/other	p-value	adj. p-value
Hypertrophic cardiomyopathy [HP:0001639]	2/6 (33%)	13/13 (100%)	0.004	0.027

(b) Fisher Exact Test performed to compare HPO annotation frequency with respect to Glu140Lys/Glu140Lys and other/other OR Glu140Lys/other. Total of 7 tests were performed.

Description	Variable	Genotype (A)	Genotype (B)	p-value	xrefs
Survival analysis: Hypertrophic cardiomyopathy	Onset of HP:0001639	Glu140Lys/Glu140Lys	other/other OR Glu140Lys/other	0.219	-

(c) Onset of Hypertrophic cardiomyopathy to compare Glu140Lys/Glu140Lys and other/other OR Glu140Lys/other with respect to Onset of HP:0001639.

Description	Variable	Genotype (A)	Genotype (B)	p-value	xrefs
Compute time until OMIM:604377 onset	Onset of OMIM:604377	Glu140Lys/Glu140Lys	other/other OR Glu140Lys/other	0.415	-

(d) Onset of OMIM:604377 to compare Glu140Lys/Glu140Lys and other/other OR Glu140Lys/other with respect to Onset of OMIM:604377.

Figure S58: The cohort comprised 37 individuals (10 females, 23 males, 4 with unknown sex). 24 of these individuals were reported to be deceased. A total of 107 HPO terms were used to annotate the cohort. Disease diagnoses: Mitochondrial complex IV deficiency, nuclear type 2 (OMIM:604377) (31 individuals), Myopia 6 (OMIM:608908) (6 individuals). The glu140lys correlation is driven by 5 patients found to be homozygous for the Glu140Lys variant [49]. The children were between the age of 8 months and 1 year and 8 months. Because of the relatively small cohort and the fact that other members of the cohort were not as young, caution is advised in the interpretation of this finding. A total of 25 unique variant alleles were found in *SCO2* (transcript: `NM_005138.3`, protein id: `NP_005129.2`).

SEC61A1

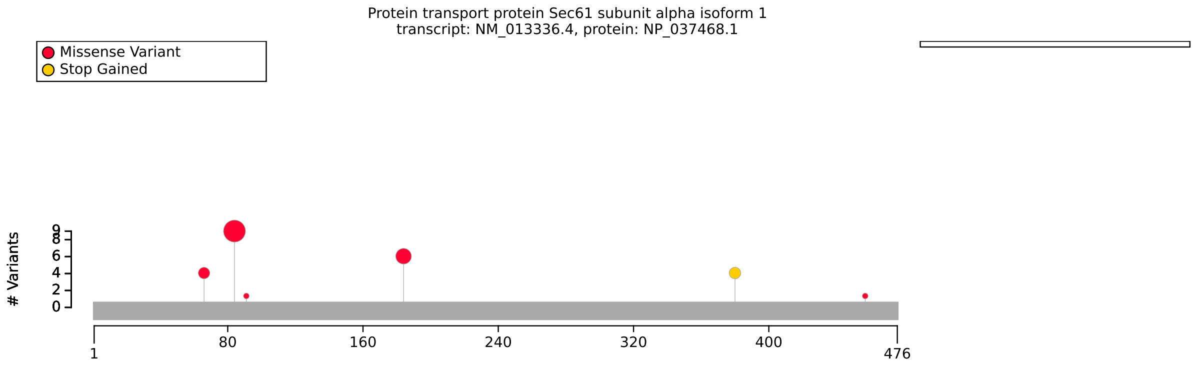

(a) Distribution of variants in *SEC61A1*

Genotype (A)	Genotype (B)	total tests performed	significant results
p.Val85Asp	Other variant	24	0
FEMALE	MALE	30	0

(b) Fisher Exact Test performed to compare HPO annotation frequency with respect to genotypes.

Figure S59: The cohort comprised 19 individuals (8 females, 11 males). 1 of these individuals were reported to be deceased. A total of 76 HPO terms were used to annotate the cohort. Disease diagnoses: Immunodeficiency, common variable, 15 (OMIM:620670) (11 individuals), Tubulointerstitial kidney disease, autosomal dominant, 5 (OMIM:617056) (7 individuals), Neutropenia, severe congenital, 11, autosomal dominant (OMIM:620674) (1 individuals). The origin of clinical diversity in patients with *SEC61A1* mutation is currently unclear. With our present patient set, a particular phenotype cannot be predicted on the basis of location or nature of the mutation [50]. A total of 6 unique variant alleles were found in *SEC61A1* (transcript: `NM_013336.4`, protein id: `NP_037468.1`).

SETD2

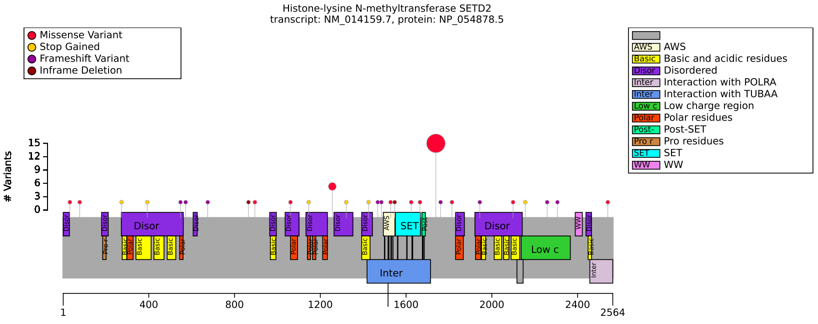

(a) Distribution of variants in *SETD2*

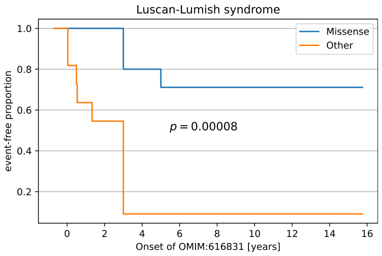

(b) Luscan-Lumish syndrome onset.

HPO term	Arg1740Trp	Other	p-value	adj. p-value
Macrocephaly [HP:0000256]	0/11 (0%)	19/28 (68%)	1.45×10^{-4}	0.002
Hypertelorism [HP:0000316]	11/11 (100%)	5/23 (22%)	1.53×10^{-5}	2.60×10^{-4}
Wide nasal bridge [HP:0000431]	9/9 (100%)	2/9 (22%)	0.002	0.012
Ventriculomegaly [HP:0002119]	4/4 (100%)	2/17 (12%)	0.003	0.012
Severe global developmental delay [HP:0011344]	9/9 (100%)	0/12 (0%)	3.40×10^{-6}	1.16×10^{-4}
Delayed ability to walk [HP:0031936]	8/8 (100%)	1/10 (10%)	4.11×10^{-4}	0.003
Scoliosis [HP:0002650]	6/6 (100%)	2/14 (14%)	7.22×10^{-4}	0.005

(c) Fisher Exact Test:HPO annotation frequency vs. Arg1740Trp / Other. Total of 34 tests were performed.

HPO term	Missense	Other	p-value	adj. p-value
Macrocephaly [HP:0000256]	4/24 (17%)	15/15 (100%)	1.54×10^{-7}	6.32×10^{-6}

(d) Fisher Exact Test: HPO annotation frequency vs. Missense / Other. Total of 41 tests were performed.

Genotype (A)	Genotype (B)	total tests performed	significant results
FEMALE	MALE	41	0

(e) Fisher Exact Test performed to compare HPO annotation frequency with respect to genotypes.

Description	Variable	Genotype (A)	Genotype (B)	p-value	xrefs
Luscan-Lumish syndrome (OMIM:616831) onset	Onset of OMIM:616831	Missense	Other	8.47×10^{-5}	-

(f) Onset of OMIM:616831 to compare Missense and Other variant.

Figure S60: The cohort comprised 45 individuals (18 females, 27 males). 1 of these individuals was reported to be deceased. A total of 202 HPO terms were used to annotate the cohort. Disease diagnoses: Luscan-Lumish syndrome (OMIM:616831) (28 individuals), Rabin-Pappas syndrome (OMIM:620155) (14 individuals), Intellectual developmental disorder, autosomal dominant 70 (OMIM:620157) (3 individuals). Van Nieuwenhove et al. (2020) stated that the origin of clinical diversity in patients with SEC61A1 mutation is currently unclear [50]. Rabin et al. (2023) found that variants in codon 1740 of *SETD2* whose features differ from those with LLS [51]. A total of 31 unique variant alleles were found in *SETD2* (transcript: `NM_014159.7`, protein id: `NP_054878.5`).

SF3B4

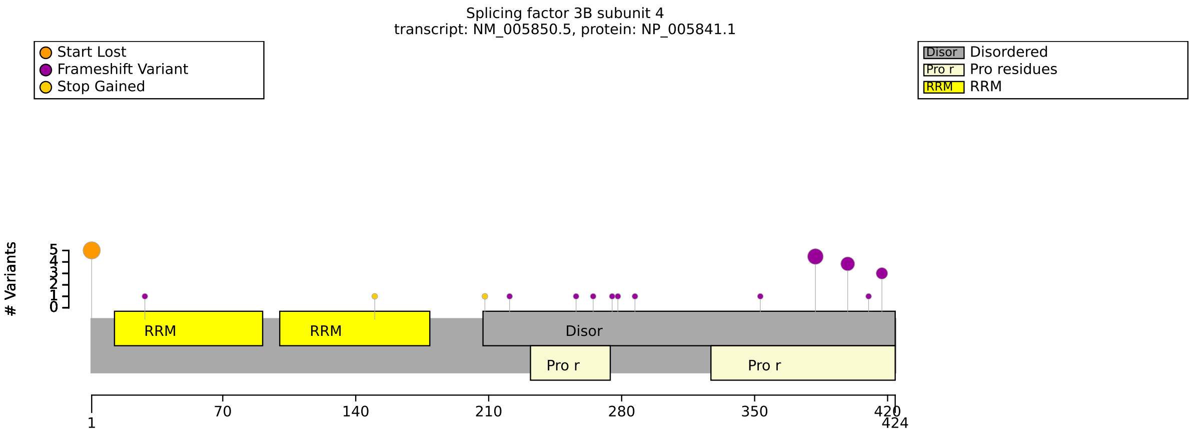

(a) Distribution of variants in *SF3B4*

Genotype (A)	Genotype (B)	total tests performed	significant results
p.Met1?	Other	46	0
FEMALE	MALE	46	0

(b) Fisher Exact Test performed to compare HPO annotation frequency with respect to genotypes.

Figure S61: The cohort comprised 26 individuals (18 females, 8 males). A total of 41 HPO terms were used to annotate the cohort. Disease diagnosis: Acrofacial dysostosis 1, Nager type (OMIM:154400). In one published analysis, it was stated that "although no significant genotype–phenotype association was found, it is notable that patients with frameshift *SF3B4* variants and predicted to lead to nonsense-mediated RNA decay (NMD) of the transcripts tended to have a more severe clinical manifestation". The authors did not correct for multiple testing and did not find nominally significant associations [52]. No significant correlation found in our study. A total of 18 unique variant alleles were found in *SF3B4* (transcript: NM_005850.5, protein id: NP_005841.1).

SLC4A1

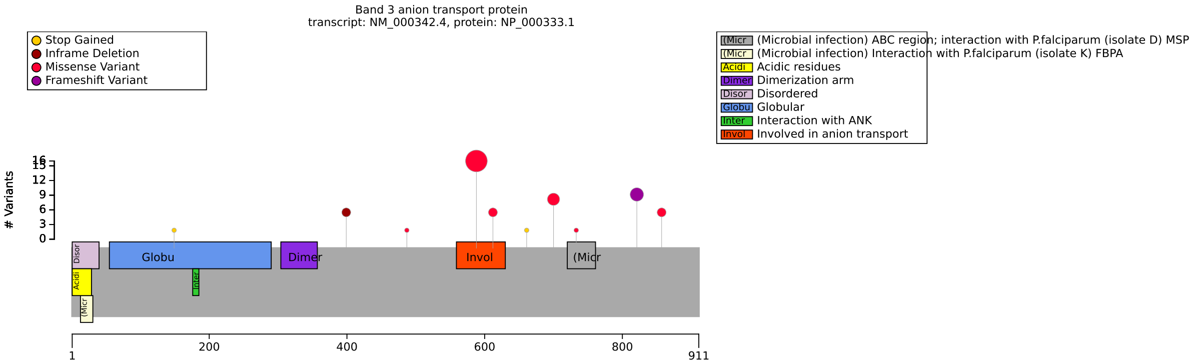

(a) Distribution of variants in *SLC4A1*

Genotype (A)	Genotype (B)	total tests performed	significant results
r149w	other	14	0

(b) Fisher Exact Test performed to compare HPO annotation frequency with respect to genotypes.

Figure S62: The cohort comprised 33 individuals (16 females, 16 males, 1 with unknown sex). A total of 49 HPO terms were used to annotate the cohort. Disease diagnoses: Distal renal tubular acidosis 1 (OMIM:179800) (18 individuals), Spherocytosis, type 4 (OMIM:612653) (7 individuals), Distal renal tubular acidosis 4 with hemolytic anemia (OMIM:611590) (7 individuals), Cryohydrocytosis (OMIM:185020) (1 individuals). No statistically significant results identified. A total of 11 unique variant alleles were found in *SLC4A1* (transcript: `NM_000342.4`, protein id: `NP_000333.1`).

SLC32A1

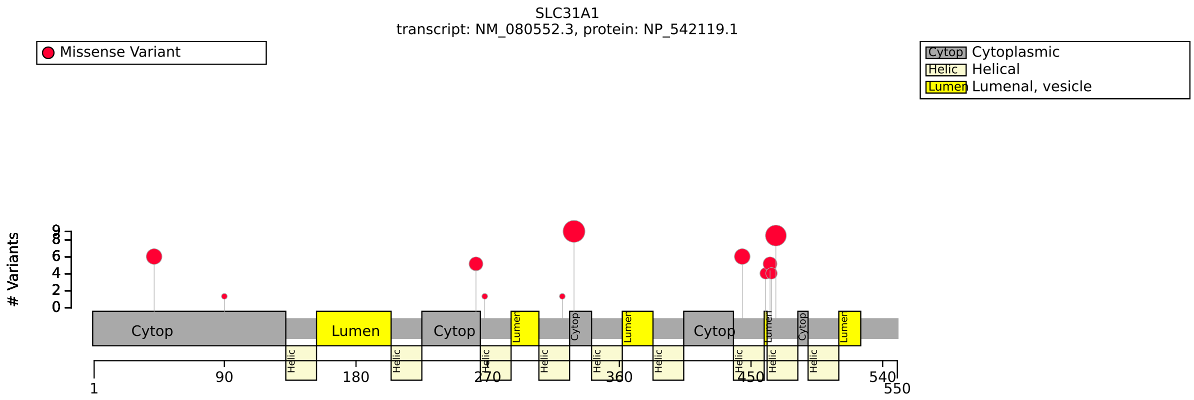

(a) Distribution of variants in *SLC32A1*

Genotype (A)	Genotype (B)	total tests performed	significant results
N term	other	11	0
p.Met330Thr	Other variant	11	0
FEMALE	MALE	11	0

(b) Fisher Exact Test performed to compare HPO annotation frequency with respect to genotypes.

Figure S63: The cohort comprised 38 individuals (19 females, 19 males). A total of 44 HPO terms were used to annotate the cohort. Disease diagnoses: Generalized epilepsy with febrile seizures plus, type 12 (OMIM:620755) (34 individuals), Developmental and epileptic encephalopathy 114 (OMIM:620774) (4 individuals). No significant correlation identified. A total of 12 unique variant alleles were found in *SLC32A1* (transcript: `NM_080552.3`, protein id: `NP_542119.1`).

SLC45A2

Genotype (A)	Genotype (B)	total tests performed	significant results
missense/missense OR missense/other	other/other	24	0
FEMALE	MALE	25	0

(a) Fisher Exact Test performed to compare HPO annotation frequency with respect to genotypes.

Figure S64: The cohort comprised 30 individuals (17 females, 13 males). A total of 16 HPO terms were used to annotate the cohort. Disease diagnosis: Albinism, oculocutaneous, type IV (OMIM:606574). No significant association identified. A total of 28 unique variant alleles were found in *SLC45A2* (transcript: `NM_016180.5`, protein id: `NP_057264.4`).

SMAD2

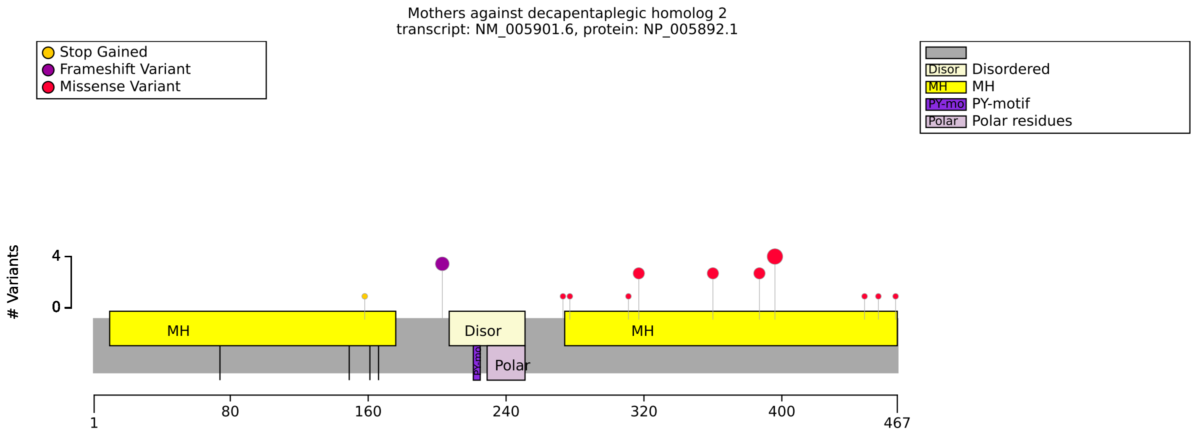

(a) Distribution of variants in *SMAD2*

Genotype (A)	Genotype (B)	total tests performed	significant results
Ser397Tyr	Other	26	0
N Term	Other	27	0
FEMALE	MALE	27	0

(b) Fisher Exact Test performed to compare HPO annotation frequency with respect to genotypes.

Figure S65: The cohort comprised 23 individuals (13 females, 8 males, 2 with unknown sex). A total of 89 HPO terms were used to annotate the cohort. Disease diagnoses: Loeys-Dietz syndrome 6 (OMIM:619656) (18 individuals), Congenital heart defects, multiple types, 8, with or without heterotaxy (OMIM:619657) (5 individuals). No significant correlations with specific *SMAD2* residues or variant categories were identified. A total of 15 unique variant alleles were found in *SMAD2* (transcript: `NM_005901.6`, protein id: `NP_005892.1`).

SMAD3

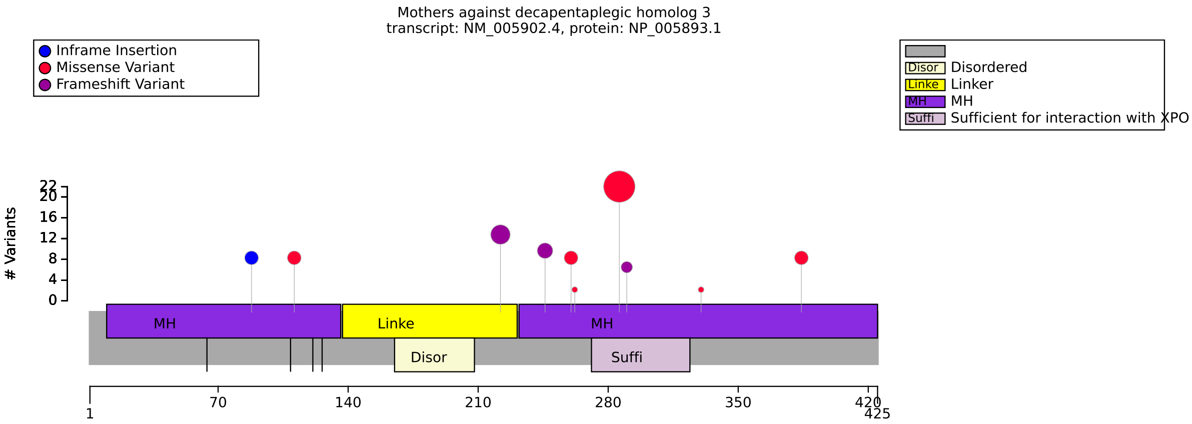

(a) Distribution of variants in *SMAD3*

HPO term	p.Arg287Trp	Other	p-value	adj. p-value
Osteoarthritis [HP:0002758]	19/19 (100%)	7/19 (37%)	3.72×10^{-5}	8.56×10^{-4}

(b) Fisher Exact Test performed to compare HPO annotation frequency with respect to p.Arg287Trp and Other. Total of 23 tests were performed.

Genotype (A)	Genotype (B)	total tests performed	significant results
missense	other	31	0
FEMALE	MALE	31	0

(c) Fisher Exact Test performed to compare HPO annotation frequency with respect to genotypes.

Figure S66: The cohort comprised 49 individuals (22 females, 27 males). A total of 30 HPO terms were used to annotate the cohort. Disease diagnosis: Loeys-Dietz syndrome 3 (OMIM:613795). There was no evidence of a correlation between missense variants and specific phenotypic abnormalities. In contrast, there was a statistically significant correlation with Arg287Trp and Osteoarthritis. The residue Arg287 is located in the Mad homology 2 (MH2) domain in a region that mediates interaction with exportin 4 [53]. Chesneau et al stated that there is an absence of correlation between the *SMAD3* variant type and the occurrence of aortic phenotypes [54]. To the best of our knowledge an association between Arg287Trp and Osteoarthritis has not been previously noted. A total of 10 unique variant alleles were found in *SMAD3* (transcript: `NM_005902.4`, protein id: `NP_005893.1`).

SMARCB1

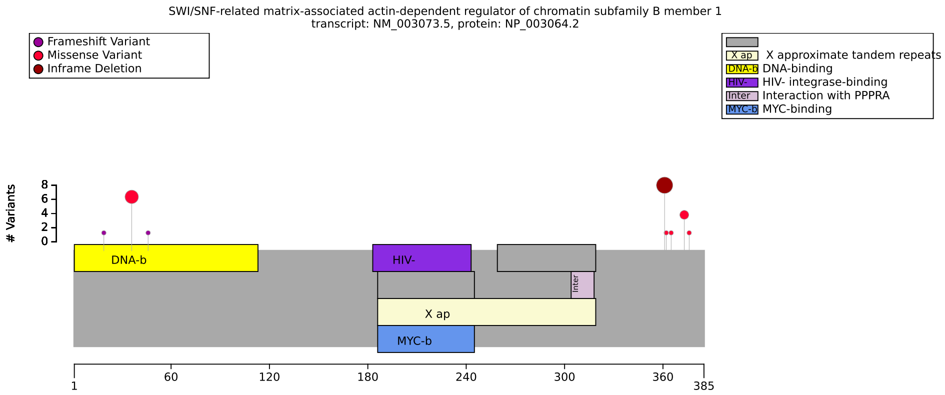

(a) Distribution of variants in *SMARCB1*

HPO term	Structural variant	Other	p-value	adj. p-value
Atypical teratoid/rhabdoid tumor [HP:0034401]	8/9 (89%)	2/19 (11%)	1.19×10^{-4}	5.94×10^{-4}
Embryonal neoplasm [HP:0002898]	8/8 (100%)	2/19 (11%)	2.03×10^{-5}	1.52×10^{-4}
Neoplasm by histology [HP:0011792]	11/11 (100%)	4/21 (19%)	1.06×10^{-5}	1.52×10^{-4}
Rhabdoid tumor [HP:0034557]	4/4 (100%)	2/19 (11%)	0.002	0.006
Neoplasm by anatomical site [HP:0011793]	3/3 (100%)	3/20 (15%)	0.011	0.028
Neuroepithelial neoplasm [HP:0030063]	2/2 (100%)	0/17 (0%)	0.006	0.018

(b) Fisher Exact Test. Total of 15 tests were performed.

Genotype (A)	Genotype (B)	total tests performed	significant results
Lys364del	Other	52	0
DNA binding	Other	51	0

(c) Fisher Exact Test performed to compare HPO annotation frequency with respect to genotypes.

Figure S67: The cohort comprised 32 individuals (12 females, 8 males, 12 with unknown sex). 9 of these individuals were reported to be deceased. A total of 110 HPO terms were used to annotate the cohort. Disease diagnoses: Coffin-Siris syndrome 3 (OMIM:614608) (18 individuals), Rhabdoid tumor predisposition syndrome 1 (OMIM:609322) (14 individuals). Our analysis of *SMARCB1* included variants associated with Coffin-Siris syndrome 3 (OMIM:614608). A total of 11 unique variant alleles were found in *SMARCB1* (transcript: `NM_003073.5`, protein id: `NP_003064.2`). Presumably the result of embryonal neoplasm being signficantly associated with structural variants relates to a different distribution of variants in rhabdoid tumor predisposition syndrome-1 than in Coffin-Siris syndrome 3. A published analysis of GPCs in Coffin-Siris syndrome did not present a statistical analysis and did not note the current association [55].

SMARCC2

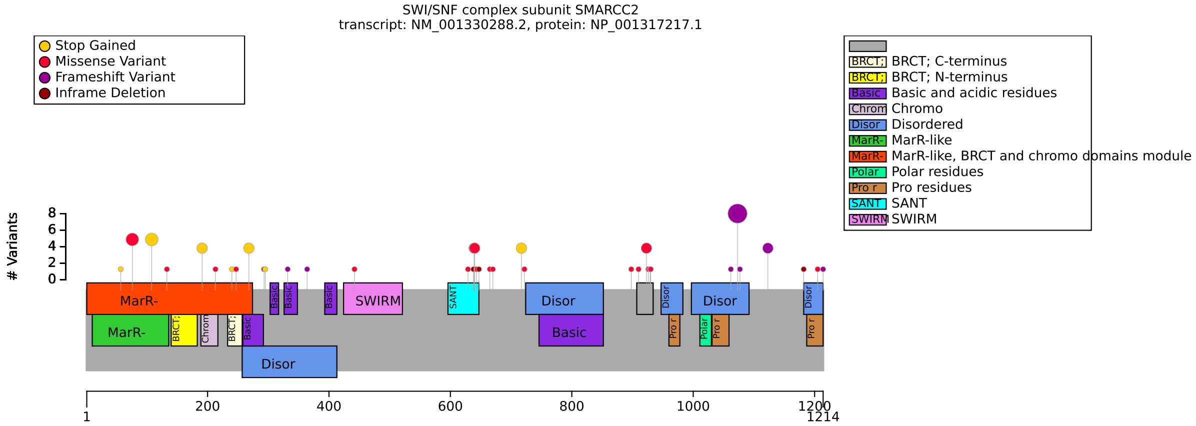

(a) Distribution of variants in *SMARCC2*

HPO term	c.3222del	other	p-value	adj. p-value
Intellectual disability [HP:0001249]	1/6 (17%)	49/52 (94%)	6.99×10^{-5}	0.006

(b) Fisher Exact Test performed to compare HPO annotation frequency with respect to c.3222del and other. Total of 83 tests were performed.

Genotype (A)	Genotype (B)	total tests performed	significant results
Missense	Truncating	72	0
N term	Other	83	0
FEMALE	MALE	83	0

(c) Fisher Exact Test performed to compare HPO annotation frequency with respect to genotypes.

Figure S68: The cohort comprised 65 individuals (24 females, 37 males, 4 with unknown sex). A total of 118 HPO terms were used to annotate the cohort. Disease diagnosis: Coffin-Siris syndrome 8 (OMIM:618362). Bosch et al. (2023) reported correlations for missense versus truncating variant cohorts for Global developmental delay; Intellectual disability (HP:0001263;HP:0001249): p = 0.005 (FDR-corr: 0.033); Muscular hypotonia (HP:0001252): p = 0.004 (FDR-corr: 0.033); mild GDD/ID (HP:0011342;HP:0001256): p = 0.012 (FDR-corr: 0.051); Abnormality of the outer ear (HP:0000356): p = 0.013 (FDR-corr: 0.051); Decreased body weight (HP:0004325): p = 0.002 (FDR-corr: 0.024); Abnormality of the eye (HP:0000478): p = 0.000 (FDR-corr: 0.008); Short stature (HP:0004322): p = 0.006 (FDR-corr: 0.035); Feeding difficulties/failure to thrive (HP:0011968;HP:0001508): p = 0.013 (FDR-corr: 0.051) [56]. The authors did not specifically test c.3222del. Differences in results may be due to different assumptions of the analysis procedure. Code to reproduce the results in Bosch et al. was not available. A total of 46 unique variant alleles were found in *SMARCC2* (transcript: `NM_001330288.2`, protein id: `NP_001317217.1`).

SON

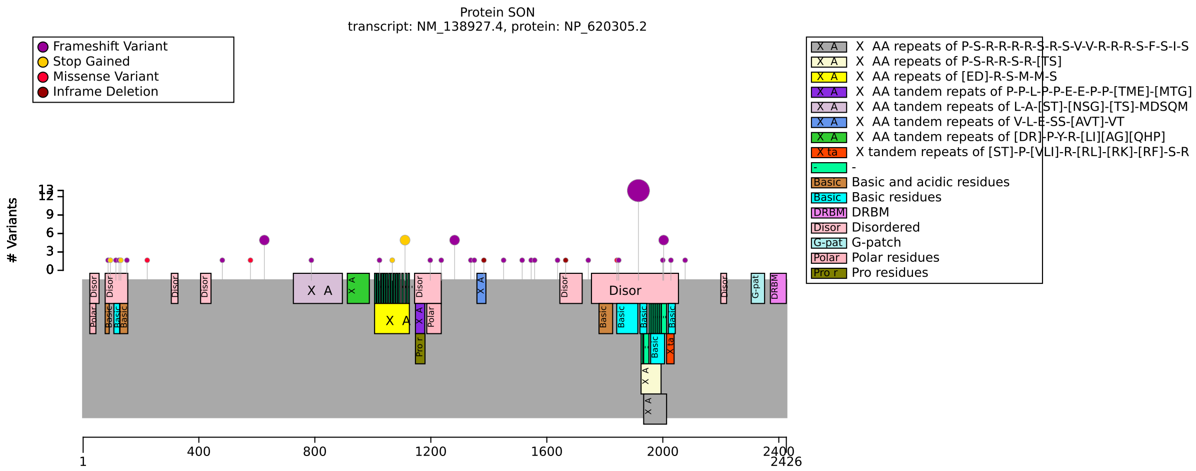

(**a**) Distribution of variants in *SON*

Genotype (A)	Genotype (B)	total tests performed	significant results
missense	Other	48	0
c.5753_5756del	Other	51	0
FEMALE	MALE	51	0

(**b**) Fisher Exact Test performed to compare HPO annotation frequency with respect to genotypes.

Figure S69: The cohort comprised 52 individuals (26 females, 26 males). A total of 47 HPO terms were used to annotate the cohort. Disease diagnosis: ZTTK SYNDROME (OMIM:617140). Dingemans et al (2020) suggested a different pathomechanism for missense variants, but our cohort only contains 3 individuals with missense variants, so there is no statistical power [57]. A total of 35 unique variant alleles were found in *SON* (transcript: `NM_138927.4`, protein id: `NP_620305.2`).

SPTAN1 (Part 1/2)

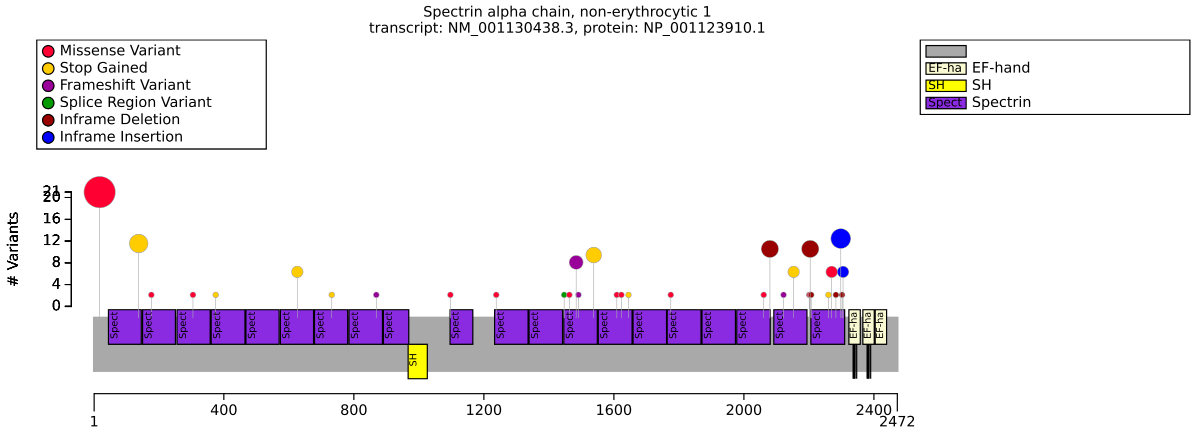

(a) Distribution of variants in *SPTAN1*

HPO term	missense	other	p-value	adj. p-value
Spastic paraplegia [HP:0001258]	21/25 (84%)	0/16 (0%)	4.70×10^{-8}	6.58×10^{-7}
Lower limb spasticity [HP:0002061]	21/22 (95%)	0/12 (0%)	2.37×10^{-8}	6.58×10^{-7}
Appendicular spasticity [HP:0034353]	21/22 (95%)	2/14 (14%)	8.72×10^{-7}	8.14×10^{-6}
Spasticity [HP:0001257]	22/23 (96%)	4/16 (25%)	5.22×10^{-6}	2.92×10^{-5}
Motor axonal neuropathy [HP:0007002]	0/19 (0%)	12/16 (75%)	2.18×10^{-6}	1.53×10^{-5}
Peripheral axonal neuropathy [HP:0003477]	4/23 (17%)	12/16 (75%)	6.69×10^{-4}	0.002
Motor seizure [HP:0020219]	7/27 (26%)	13/17 (76%)	0.002	0.004
Seizure [HP:0001250]	13/33 (39%)	24/28 (86%)	2.48×10^{-4}	9.92×10^{-4}
Intellectual disability [HP:0001249]	9/32 (28%)	18/23 (78%)	3.43×10^{-4}	0.001
Lower limb muscle weakness [HP:0007340]	15/16 (94%)	16/28 (57%)	0.015	0.030
Distal lower limb muscle weakness [HP:0009053]	9/19 (47%)	0/16 (0%)	0.001	0.004
Infantile spasms [HP:0012469]	2/29 (7%)	13/31 (42%)	0.002	0.005
Epileptic spasm [HP:0011097]	2/22 (9%)	13/17 (76%)	2.93×10^{-5}	1.37×10^{-4}
Microcephaly [HP:0000252]	4/34 (12%)	15/32 (47%)	0.002	0.005

(b) Fisher Exact Test performed to compare HPO annotation frequency with respect to missense and other. Total of 28 tests were performed.

HPO term	truncating	other	p-value	adj. p-value
Spastic paraplegia [HP:0001258]	0/8 (0%)	21/33 (64%)	0.001	0.005
Lower limb spasticity [HP:0002061]	0/8 (0%)	21/26 (81%)	7.09×10^{-5}	3.54×10^{-4}
Appendicular spasticity [HP:0034353]	0/8 (0%)	23/28 (82%)	4.25×10^{-5}	2.66×10^{-4}
Spasticity [HP:0001257]	0/8 (0%)	26/31 (84%)	2.09×10^{-5}	1.74×10^{-4}
Hypotonia [HP:0001252]	1/11 (9%)	15/24 (62%)	0.004	0.015
Motor axonal neuropathy [HP:0007002]	12/12 (100%)	0/23 (0%)	1.20×10^{-9}	3.00×10^{-8}
Peripheral axonal neuropathy [HP:0003477]	12/12 (100%)	4/27 (15%)	4.65×10^{-7}	5.82×10^{-6}

(c) Fisher Exact Test performed to compare HPO annotation frequency with respect to truncating and other. Total of 25 tests were performed.

Figure S70: See next page for caption

SPTAN1 (Part 2/2)

HPO term	Arg19Trp	Other	p-value	adj. p-value
Spastic paraplegia [HP:0001258]	21/21 (100%)	0/20 (0%)	3.72×10^{-12}	9.29×10^{-11}
Lower limb spasticity [HP:0002061]	21/21 (100%)	0/13 (0%)	1.08×10^{-9}	1.35×10^{-8}
Appendicular spasticity [HP:0034353]	21/21 (100%)	2/15 (13%)	4.54×10^{-8}	1.89×10^{-7}
Spasticity [HP:0001257]	21/21 (100%)	5/18 (28%)	1.05×10^{-6}	3.30×10^{-6}
Motor axonal neuropathy [HP:0007002]	0/16 (0%)	12/19 (63%)	6.26×10^{-5}	1.74×10^{-4}
Peripheral axonal neuropathy [HP:0003477]	4/20 (20%)	12/19 (63%)	0.010	0.017
Motor seizure [HP:0020219]	0/19 (0%)	20/25 (80%)	3.34×10^{-8}	1.67×10^{-7}
Seizure [HP:0001250]	2/21 (10%)	35/40 (88%)	2.36×10^{-9}	1.47×10^{-8}
Intellectual disability [HP:0001249]	0/21 (0%)	27/34 (79%)	1.76×10^{-9}	1.47×10^{-8}
Lower limb muscle weakness [HP:0007340]	14/14 (100%)	17/30 (57%)	0.003	0.007
Distal lower limb muscle weakness [HP:0009053]	8/15 (53%)	1/20 (5%)	0.002	0.004
Infantile spasms [HP:0012469]	0/19 (0%)	15/41 (37%)	0.001	0.003
Epileptic spasm [HP:0011097]	0/19 (0%)	15/20 (75%)	7.71×10^{-7}	2.75×10^{-6}
Microcephaly [HP:0000252]	0/21 (0%)	19/45 (42%)	2.45×10^{-4}	6.14×10^{-4}

(a) Fisher Exact Test performed to compare HPO annotation frequency with respect to Arg19Trp and Other. Total of 25 tests were performed.

Genotype (A)	Genotype (B)	total tests performed	significant results
Lys2083del	Other	21	0
FEMALE	MALE	28	0

(b) Fisher Exact Test performed to compare HPO annotation frequency with respect to genotypes.

Figure S71: The cohort comprised 85 individuals (40 females, 45 males). 3 of these individuals were reported to be deceased. A total of 96 HPO terms were used to annotate the cohort. Disease diagnoses: Spastic paraplegia 91, autosomal dominant, with or without cerebellar ataxia (OMIM:620538) (28 individuals), Developmental and epileptic encephalopathy 5 (OMIM:613477) (22 individuals), Developmental delay with or without epilepsy (OMIM:620540) (21 individuals), Neuronopathy, distal hereditary motor, autosomal dominant 11 (OMIM:620528) (14 individuals). Several publications have identified genotype-phenotype correlations in *SPTAN1* that are comparable to those identified here [58, 59, 60]. A total of 36 unique variant alleles were found in *SPTAN1* (transcript: `NM_001130438.3`, protein id: `NP_001123910.1`).

STXBP1

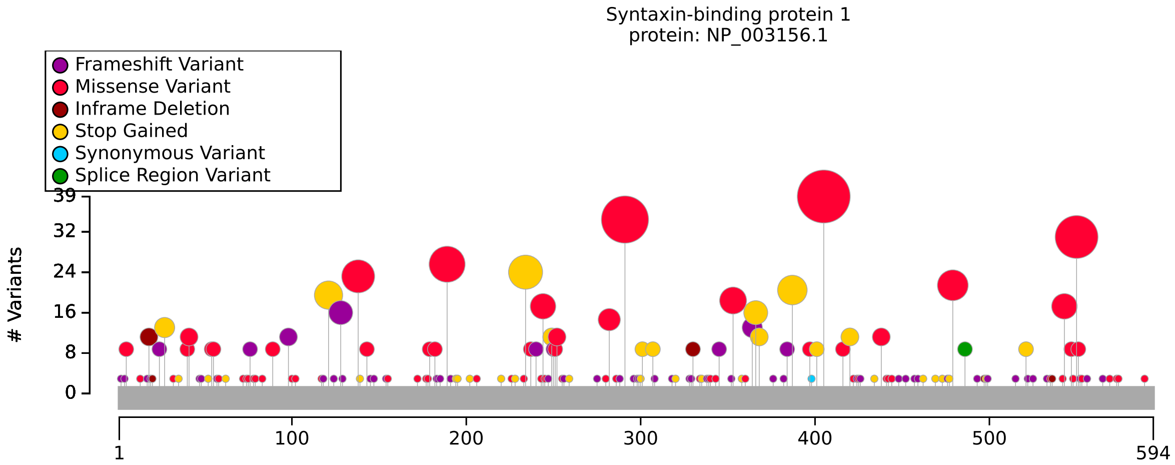

(a) Distribution of variants in *STXBP1*

Genotype (A)	Genotype (B)	total tests performed	significant results
Missense	Other	18	0
Arg406 Variants	Other	18	0
Exon 14	Other	18	0

(b) Fisher Exact Test performed to compare HPO annotation frequency with respect to genotypes.

Figure S72: The cohort comprised 462 individuals (206 females, 220 males, 36 with unknown sex). A total of 516 HPO terms were used to annotate the cohort. Disease diagnosis: Developmental and epileptic encephalopathy 4 (OMIM:612164). No significant genotype-phenotype correlations identified. A total of 259 unique variant alleles were found in *STXBP1* (transcript: `NM_003165.6`, protein id: `NP_003156.1`).

SUOX

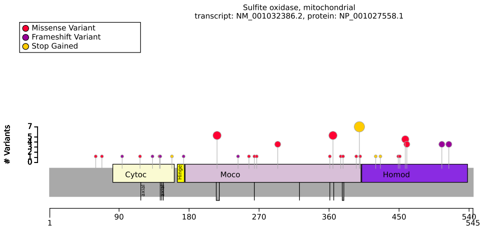

(a) Distribution of variants in *SUOX*

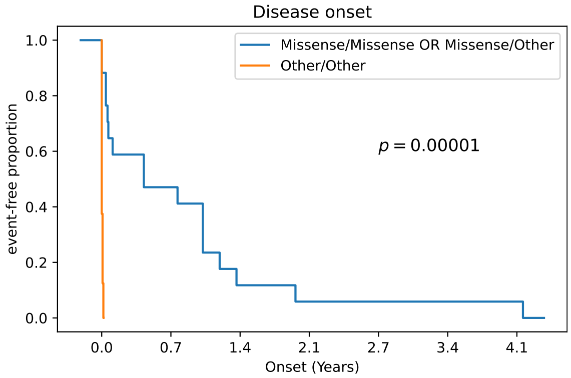

(b) Survival analysis: *SUOX* missense variants and disease onset

HPO term	homodimerization/homodimerization OR homodimerization/Other	Other/Other	p-value	adj. p-value
Microcephaly [HP:0000252]	0/9 (0%)	10/12 (83%)	2.21×10^{-4}	0.003

(c) Fisher Exact Test performed to compare HPO annotation frequency with respect to homodimerization/homodimerization OR homodimerization/Other and Other/Other. Total of 15 tests were performed.

Genotype (A)	Genotype (B)	total tests performed	significant results
Missense/Missense OR Missense/Other	Other/Other	15	0
Moco domain/Moco domain OR Moco domain/Other	Other/Other	15	0

(d) Fisher Exact Test performed to compare HPO annotation frequency with respect to genotypes.

Description	Variable	Genotype (A)	Genotype (B)	p-value	xrefs
Onset of Sulfite oxidase deficiency (OMIM:272300)	Onset of OMIM:272300	Missense/Missense OR Missense/Other	Other/Other	9.18×10^{-6}	-

(e) Onset of OMIM:272300 to compare Missense/Missense OR Missense/Other and Other/Other with respect to Onset of OMIM:272300.

Description	Variable	Genotype (A)	Genotype (B)	p-value	xrefs
Onset of Sulfite oxidase deficiency (OMIM:272300)	Onset of OMIM:272300	homodimerization/homodimerization OR homodimerization/Other	Other/Other	0.853	[61]

(f) Onset of OMIM:272300 to compare homodimerization/homodimerization OR homodimerization/Other and Other/Other with respect to Onset of OMIM:272300.

Figure S73: The cohort comprised 35 individuals (11 females, 14 males, 10 with unknown sex). 8 of these individuals were reported to be deceased. A total of 20 HPO terms were used to annotate the cohort. Disease diagnosis: Sulfite oxidase deficiency (OMIM:272300). A published Genotype-phenotype analysis demonstrated patients with biallelic missense mutations had milder symptoms (P = 0.023), later age of onset (P < 0.001), and a higher incidence of regression (P = 0.017) than other genotypes [61]. A total of 32 unique variant alleles were found in *SUOX* (transcript: `NM_001032386.2`, protein id: `NP_001027558.1`).

TBCK

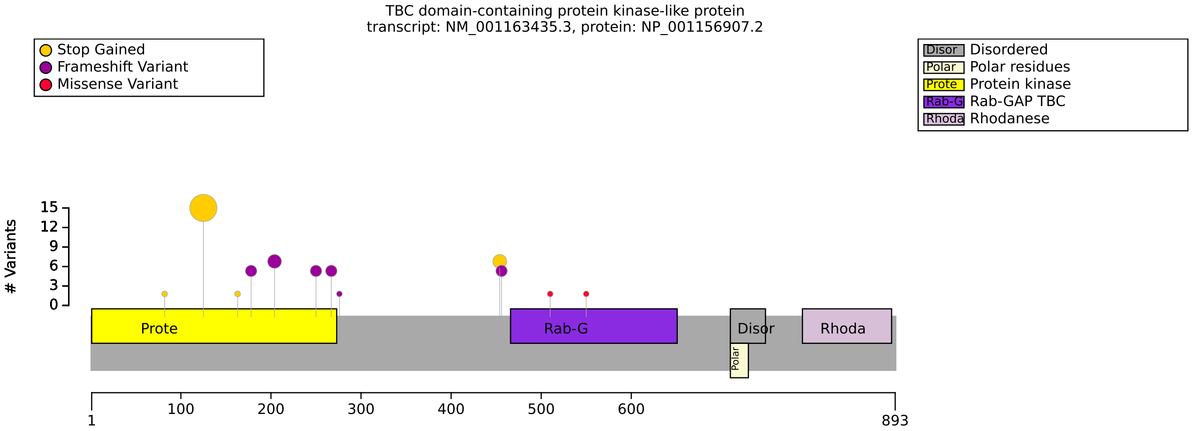

(a) Distribution of variants in *TBCK*

HPO term	R126*/R126*	R126*/other OR other/other	p-value	adj. p-value
Macroglossia [HP:0000158]	11/12 (92%)	3/22 (14%)	1.34×10^{-5}	4.30×10^{-4}
Developmental regression [HP:0002376]	9/12 (75%)	2/22 (9%)	1.83×10^{-4}	0.003

(b) Fisher Exact Test performed to compare HPO annotation frequency with respect to R126*/R126* and R126*/other OR other/other. Total of 32 tests were performed.

Genotype (A)	Genotype (B)	total tests performed	significant results
missense/missense OR missense/other	other/other	30	0
FEMALE	MALE	32	0

(c) Fisher Exact Test performed to compare HPO annotation frequency with respect to genotypes.

Figure S74: The cohort comprised 41 individuals (17 females, 24 males). 3 of these individuals were reported to be deceased. A total of 97 HPO terms were used to annotate the cohort. Disease diagnosis: Hypotonia, infantile, with psychomotor retardation and characteristic facies 3 (OMIM:616900). Durham et al (2023) stated that several studies have touched on a genotype-phenotype correlation of TBCK syndrome; however, more data are required for statistically significant conclusions [62]. A total of 18 unique variant alleles were found in *TBCK* (transcript: `NM_001163435.3`, protein id: `NP_001156907.2`).

TBX1

HPO term	Tyr418PhefsTer42	Other	p-value	adj. p-value
Global developmental delay [HP:0001263]	5/5 (100%)	3/20 (15%)	0.001	0.023
Narrow nose [HP:0000460]	5/5 (100%)	0/6 (0%)	0.002	0.024

(a) Fisher Exact Test performed to compare HPO annotation frequency with respect to Tyr418PhefsTer42 and Other. Total of 22 tests were performed.

Genotype (A)	Genotype (B)	total tests performed	significant results
Missense	Other	21	0

(b) Fisher Exact Test performed to compare HPO annotation frequency with respect to genotypes.

Figure S75: The cohort comprised 26 individuals (10 females, 15 males, 1 with unknown sex). A total of 25 HPO terms were used to annotate the cohort. Disease diagnosis: DiGeorge syndrome (OMIM:188400). A total of 12 unique variant alleles were found in *TBX1* (transcript: `NM_001379200.1`, protein id: `NP_001366129.1`).

TBX5

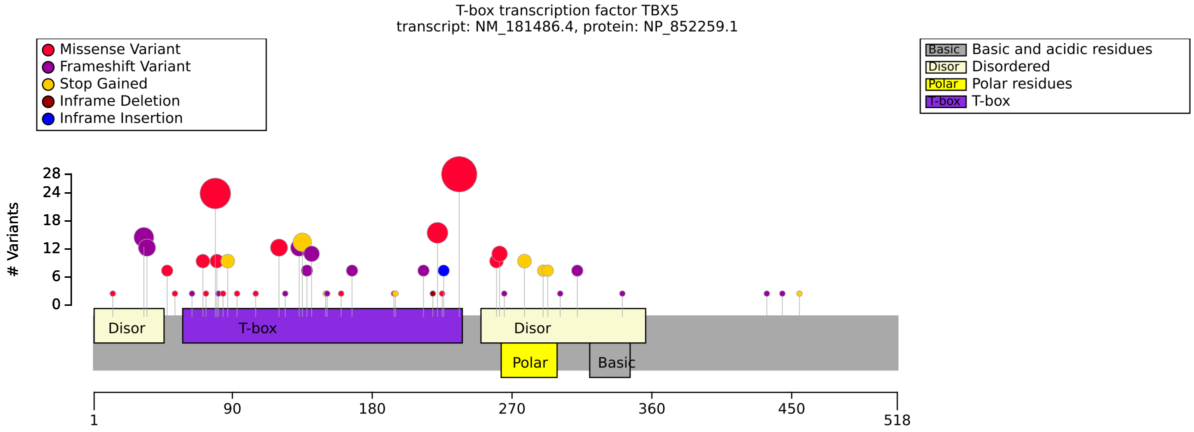

(a) Distribution of variants in *TBX5*

HPO term	missense	other	p-value	adj. p-value
Ventricular septal defect [HP:0001629]	31/60 (52%)	30/30 (100%)	4.63×10^{-7}	1.48×10^{-5}

(b) Fisher Exact Test performed to compare HPO annotation frequency with respect to missense and other. Total of 32 tests were performed.

HPO term	Arg237Gln	other	p-value	adj. p-value
Ventricular septal defect [HP:0001629]	0/17 (0%)	61/73 (84%)	5.55×10^{-11}	1.78×10^{-9}
Upper limb phocomelia [HP:0009813]	7/22 (32%)	3/131 (2%)	4.55×10^{-5}	7.28×10^{-4}

(c) Fisher Exact Test performed to compare HPO annotation frequency with respect to Arg237Gln and other. Total of 32 tests were performed.

Genotype (A)	Genotype (B)	total tests performed	significant results
Gly80Arg	other	28	0
FEMALE	MALE	13	0

(d) Fisher Exact Test performed to compare HPO annotation frequency with respect to genotypes.

Figure S76: The cohort comprised 156 individuals (56 females, 46 males, 54 with unknown sex). A total of 90 HPO terms were used to annotate the cohort. Disease diagnosis: Holt-Oram syndrome (OMIM:142900). Vanlerberghe et al. (2019) observed that isolated septal CHD are more common in the truncating than in the missense variants (p=0.02) [63]. A total of 53 unique variant alleles were found in *TBX5* (transcript: `NM_181486.4`, protein id: `NP_852259.1`).

TGFB2

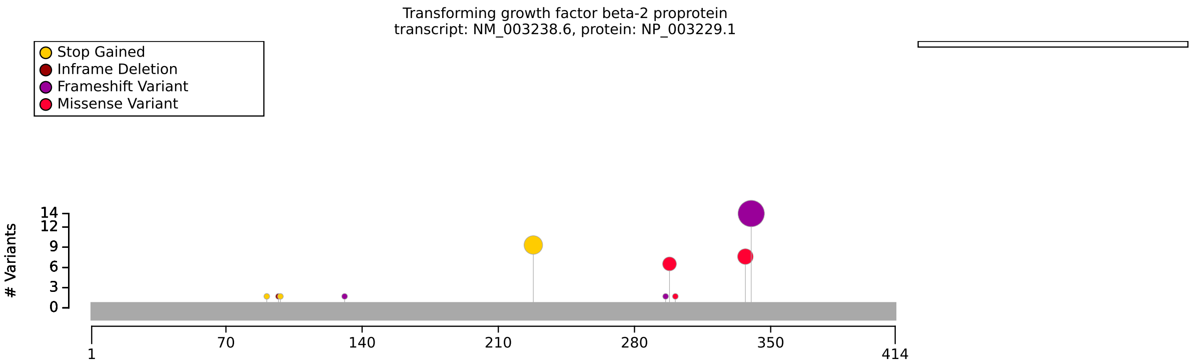

(a) Distribution of variants in *TGFB2*

Genotype (A)	Genotype (B)	total tests performed	significant results
missense	other	37	0
FEMALE	MALE	38	0

(b) Fisher Exact Test performed to compare HPO annotation frequency with respect to genotypes.

Figure S77: The cohort comprised 36 individuals (10 females, 26 males). 1 of these individuals were reported to be deceased. A total of 68 HPO terms were used to annotate the cohort. Disease diagnosis: Loeys-Dietz syndrome 4 (OMIM:614816). No significant association found. A total of 12 unique variant alleles were found in *TGFB2* (transcript: `NM_003238.6`, protein id: `NP_003229.1`).

TGFB3

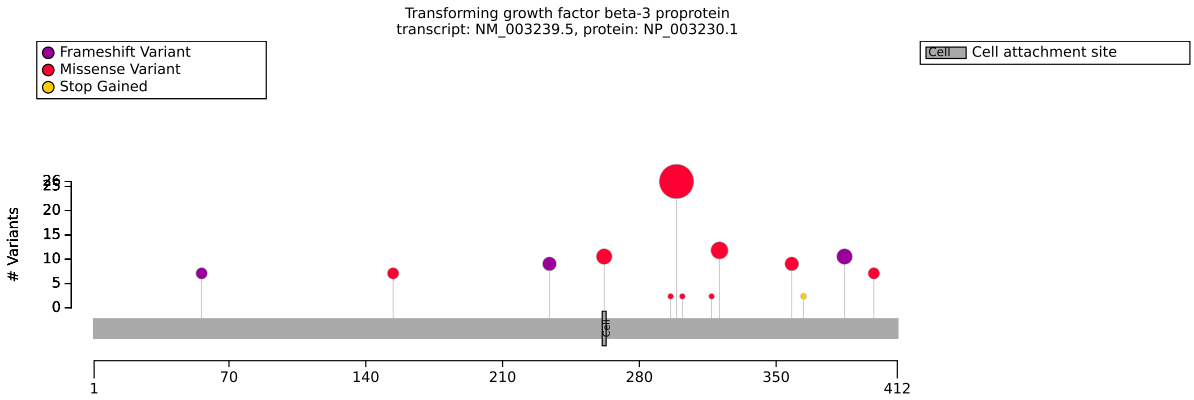

(a) Distribution of variants in *TGFB3*

Genotype (A)	Genotype (B)	total tests performed	significant results
Missense	Other	57	0
Asp263His	Other	18	0
FEMALE	MALE	57	0

(b) Fisher Exact Test performed to compare HPO annotation frequency with respect to genotypes.

Figure S78: The cohort comprised 75 individuals (34 females, 41 males). 7 of these individuals were reported to be deceased. A total of 73 HPO terms were used to annotate the cohort. Disease diagnosis: Loeys-Dietz syndrome 5 (OMIM:615582). No significant correlations identified. A total of 18 unique variant alleles were found in *TGFB3* (transcript: `NM_003239.5`, protein id: `NP_003230.1`).

TGFBR1

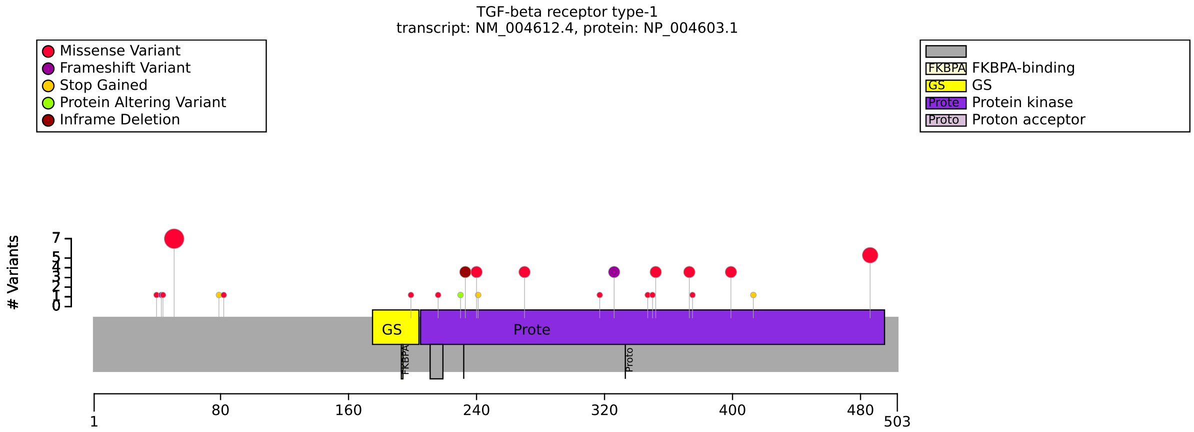

(a) Distribution of variants in *TGFBR1*

HPO term	MSSE var	other	p-value	adj. p-value
Self-healing squamous epithelioma [HP:0034720]	18/19 (95%)	0/21 (0%)	1.68×10^{-10}	2.68×10^{-9}
Hypertelorism [HP:0000316]	0/18 (0%)	15/19 (79%)	5.01×10^{-7}	4.01×10^{-6}
Arterial tortuosity [HP:0005116]	1/19 (5%)	8/16 (50%)	0.005	0.026

(b) Fisher Exact Test performed to compare HPO annotation frequency with respect to MSSE var and other. Total of 16 tests were performed.

HPO term	Gly52Arg	Other	p-value	adj. p-value
Self-healing squamous epithelioma [HP:0034720]	7/7 (100%)	11/33 (33%)	0.002	0.012

(c) Fisher Exact Test performed to compare HPO annotation frequency with respect to Gly52Arg and Other. Total of 7 tests were performed.

Genotype (A)	Genotype (B)	total tests performed	significant results
FEMALE	MALE	21	0

(d) Fisher Exact Test performed to compare HPO annotation frequency with respect to genotypes.

Figure S79: The cohort comprised 41 individuals (7 females, 16 males, 18 with unknown sex). 2 of these individuals were reported to be deceased. A total of 78 HPO terms were used to annotate the cohort. Disease diagnoses: Loeys-Dietz syndrome 1 (OMIM:609192) (23 individuals), Multiple self-healing squamous epithelioma, susceptibility to (OMIM:132800) (18 individuals). A total of 27 unique variant alleles were found in *TGFBR1* (transcript: NM_004612.4, protein id: NP_004603.1). Goudie et al. (2011) reported that the nature of the sequence variants, which include mutations in the extracellular ligand-binding domain and a series of truncating mutations in the kinase domain, indicates a clear genotype-phenotype correlation between loss-of-function *TGFBR1* mutations and MSSE [64].

TGFBR2

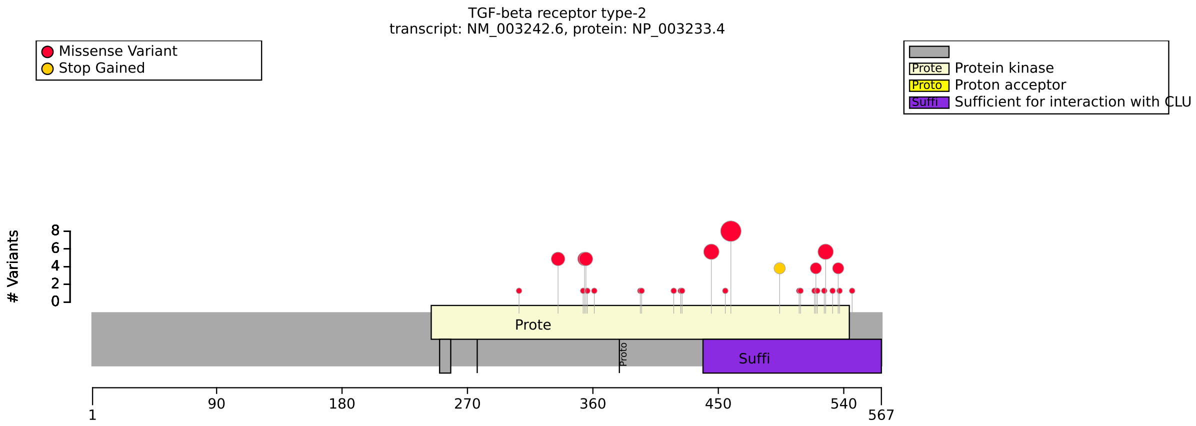

(a) Distribution of variants in *TGFBR2*

Genotype (A)	Genotype (B)	total tests performed	significant results
CLU interaction region	Other	42	0
Missense	Other	42	0
FEMALE	MALE	42	0

(b) Fisher Exact Test performed to compare HPO annotation frequency with respect to genotypes.

Figure S80: The cohort comprised 53 individuals (23 females, 30 males). 4 of these individuals were reported to be deceased. A total of 96 HPO terms were used to annotate the cohort. Disease diagnosis: Loeys-Dietz syndrome 2 (OMIM:610168). No statistically significant results identified. A total of 34 unique variant alleles were found in *TGFBR2* (transcript: NM_003242.6, protein id: NP_003233.4).

TRAF7

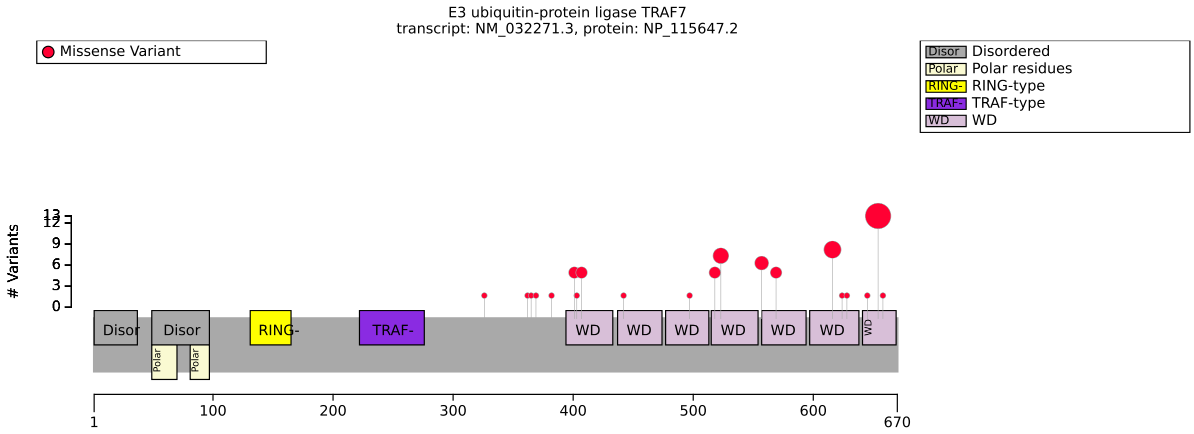

(a) Distribution of variants in *TRAF7*

Genotype (A)	Genotype (B)	total tests performed	significant results
WD7	other	40	0
Arg655Gln	Other variant	40	0
FEMALE	MALE	40	0

(b) Fisher Exact Test performed to compare HPO annotation frequency with respect to genotypes.

Figure S81: The cohort comprised 45 individuals (17 females, 28 males). A total of 360 HPO terms were used to annotate the cohort. Disease diagnosis: Cardiac, facial, and digital anomalies with developmental delay (OMIM:618164). No significant correlations identified. A total of 23 unique variant alleles were found in *TRAF7* (transcript: `NM_032271.3`, protein id: `NP_115647.2`).

U2AF2

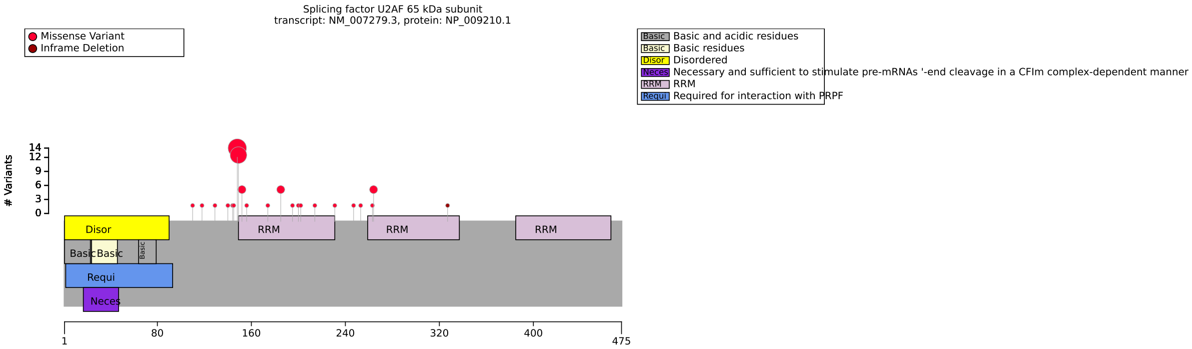

(a) Distribution of variants in *U2AF2*

Genotype (A)	Genotype (B)	total tests performed	significant results
r149W	other	177	0
R149,R150 variants	other	177	0
RRM 1	other	177	0
FEMALE	MALE	177	0

(b) Fisher Exact Test performed to compare HPO annotation frequency with respect to genotypes.

Figure S82: The cohort comprised 48 individuals (28 females, 20 males). 2 of these individuals were reported to be deceased. A total of 178 HPO terms were used to annotate the cohort. Disease diagnosis: Developmental delay, dysmorphic facies, and brain anomalies (OMIM:620535). No statistical analysis of GPCs in *U2AF2* identified in published literature. A total of 24 unique variant alleles were found in *U2AF2* (transcript: `NM_007279.3`, protein id: `NP_009210.1`).

UMOD

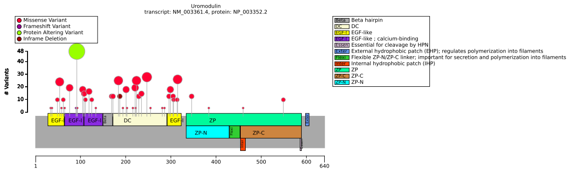

(a) Distribution of variants in *UMOD*

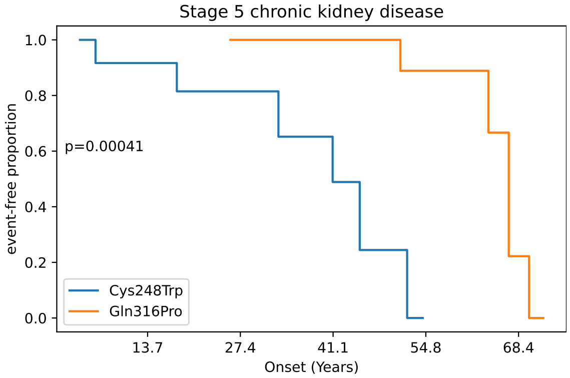

(b) Onset of Stage 5 chronic kidney disease (HP:0003774)

HPO term	EGF	other	p-value	adj. p-value
Hyperuricemia [HP:0002149]	14/32 (44%)	50/57 (88%)	1.77×10^{-5}	1.06×10^{-4}

(c) Fisher Exact Test: HPO annotation frequency and EGF vs. other. Total of 6 tests were performed.

HPO term	cysteine	other	p-value	adj. p-value
Hyperuricemia [HP:0002149]	38/41 (93%)	26/48 (54%)	4.48×10^{-5}	2.69×10^{-4}

(d) Fisher Exact Test: HPO annotation frequency and cysteine vs other. Total of 6 tests were performed.

Genotype (A)	Genotype (B)	total tests performed	significant results
FEMALE	MALE	5	0

(e) Fisher Exact Test performed to compare HPO annotation frequency with respect to genotypes.

Description	Variable	Genotype (A)	Genotype (B)	p-value	xrefs
Survival analysis: Stage 5 chronic kidney disease	Onset of HP:0003774	EGF	other	0.284	-

(f) Onset of Stage 5 chronic kidney disease to compare EGF and other with respect to Onset of HP:0003774.

Description	Variable	Genotype (A)	Genotype (B)	p-value	xrefs
Survival analysis: Stage 5 chronic kidney disease	Onset of HP:0003774	278_289delins	other	0.835	-

(g) Onset of Stage 5 chronic kidney disease to compare 278_289delins and other with respect to Onset of HP:0003774.

Description	Variable	Genotype (A)	Genotype (B)	p-value	xrefs
Survival analysis: Stage 5 chronic kidney disease	Onset of HP:0003774	Cys248Trp	Gln316Pro	4.10×10^{-4}	-

(h) Onset of Stage 5 chronic kidney disease to compare Cys248Trp and Gln316Pro with respect to Onset of Stage 5 chronic kidney disease (HP:0003774).

Figure S83: The cohort comprised 207 individuals (80 females, 88 males, 39 with unknown sex). 30 HPO terms were used to annotate the cohort. Disease diagnoses: Tubulointerstitial kidney disease, autosomal dominant, 1 (OMIM:162000) (183 individuals), Tubulointerstitial kidney disease, autosomal dominant, 3 (OMIM:162002) (12 individuals), Tubulointerstitial kidney disease, autosomal dominant, 2 (OMIM:162001) (12 individuals). One study on *UMOD* variants showed that median ages at ESRD development were lowest with Cys77Tyr and highest with Gln316Pro [65], which is compatible with our finding here. Another showed that indel mutation p.Val93_Gly97delinsAlaAlaSerCys is associated with a relatively mild clinical UAKD phenotype [66]. We did not observe a significant association between this variant and age of onset with our dataset. A total of 53 unique variant alleles were found in *UMOD* (transcript: `NM_003361.4`, protein id: `NP_003352.2`).

WWOX

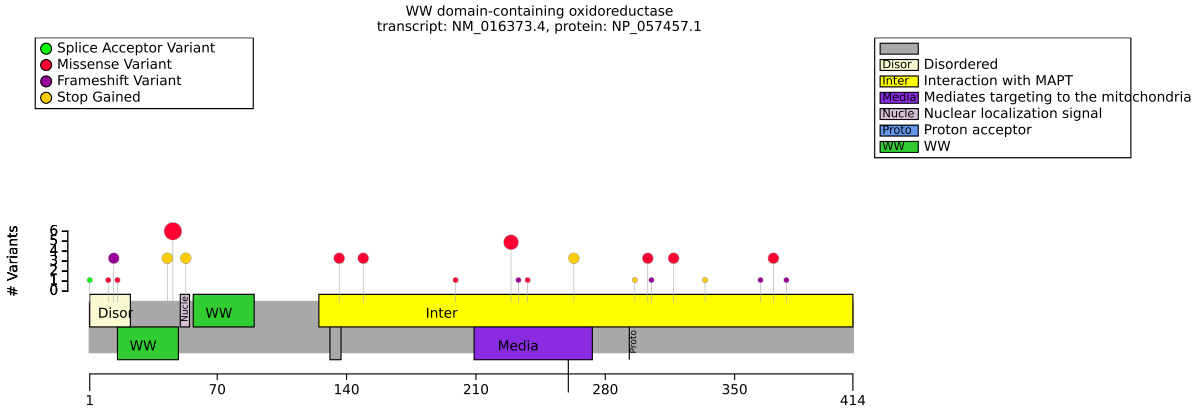

(a) Distribution of variants in *WWOX*

HPO term	MAPT Interaction/MAPT Interaction OR MAPT Interaction/Other	Other/Other	p-value	adj. p-value
Bilateral tonic-clonic seizure with focal onset [HP:0007334]	0/18 (0%)	7/9 (78%)	4.05×10^{-5}	0.002

(b) Fisher Exact Test performed to compare HPO annotation frequency with respect to MAPT Interaction/MAPT Interaction OR MAPT Interaction/Other and Other/Other. Total of 44 tests were performed.

Genotype (A)	Genotype (B)	total tests performed	significant results
Missense/Missense OR Missense/Other	Other/Other	44	0
FEMALE	MALE	44	0

(c) Fisher Exact Test performed to compare HPO annotation frequency with respect to genotypes.

Figure S84: The cohort comprised 38 individuals (25 females, 13 males). 11 of these individuals were reported to be deceased. A total of 72 HPO terms were used to annotate the cohort. Disease diagnoses: Developmental and epileptic encephalopathy 28 (OMIM:616211) (32 individuals), Spinocerebellar ataxia, autosomal recessive 12 (OMIM:614322) (6 individuals). Phenotype/genotype correlations were recently suggested for *WWOX*-related neurodevelopmental disorders with a classification of *WWOX* genotypes into three groups. According to the tentative classification, patients carrying two predicted null alleles were more likely to present with the most severe WOREE phenotype whereas hypomorphic genotypes with two missense variants would instead result in spinocerebellar ataxia (SCAR12) [67, 68, 69]. A total of 33 unique variant alleles were found in *WWOX* (transcript: `NM_016373.4`, protein id: `NP_057457.1`).

ZFX

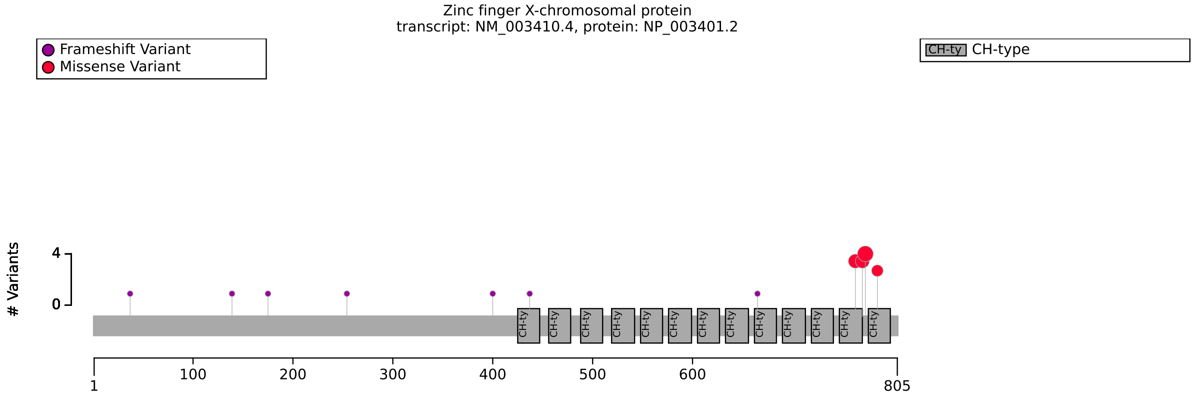

(a) Distribution of variants in *ZFX*

HPO term	missense	other	p-value	adj. p-value
Hyperparathyroidism [HP:0000843]	7/9 (78%)	0/5 (0%)	0.021	0.021

(b) Fisher Exact Test performed to compare HPO annotation frequency with respect to missense and other. Total of 1 test was performed.

Genotype (A)	Genotype (B)	total tests performed	significant results
missense	other	225	0

(c) Fisher Exact Test performed to compare HPO annotation frequency with respect to genotypes.

Figure S85: The cohort comprised 19 individuals (4 females, 14 males, 1 with unknown sex). A total of 203 HPO terms were used to annotate the cohort. Disease diagnosis: Intellectual developmental disorder, X-linked syndromic 37 (OMIM:301118). The small cohort size and the fact that the majority of the *ZFX* variants were private to each proband or family made the assessment of genotype-phenotype correlation difficult. A recent report of a germline *ZFX* missense variant in a patient With primary hyperparathyroidism suggested the hypothesis of testing for correlation between missense variants and hyperparathyroidism [70, 71]. A total of 11 unique variant alleles were found in *ZFX* (transcript: `NM_003410.4`, protein id: `NP_003401.2`).

ZMYM3

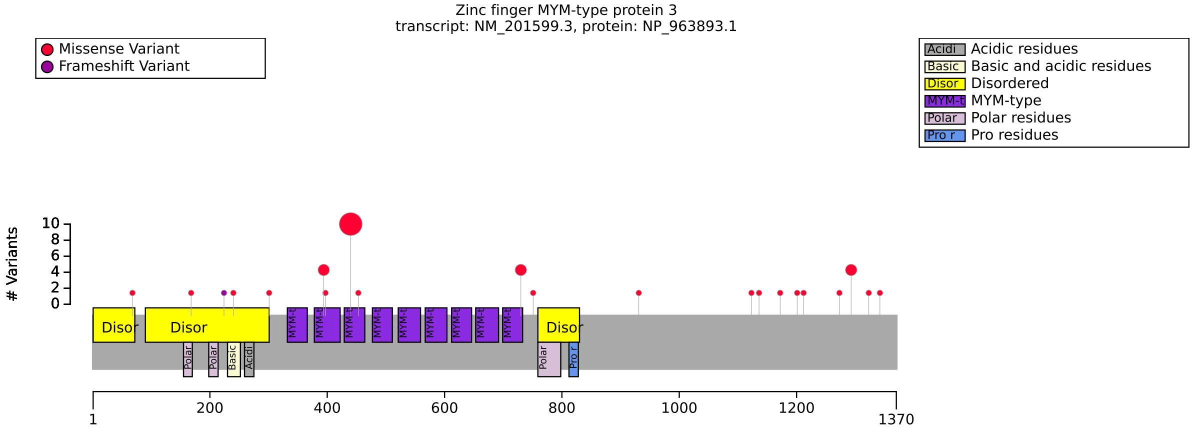

(a) Distribution of variants in *ZMYM3*

HPO term	R441	other	p-value	adj. p-value
Cupped ear [HP:0000378]	7/10 (70%)	1/23 (4%)	2.02×10^{-4}	0.012

(b) Fisher Exact Test performed to compare HPO annotation frequency with respect to R441 and other. Total of 60 tests were performed.

Genotype (A)	Genotype (B)	total tests performed	significant results
N term	other	60	0

(c) Fisher Exact Test performed to compare HPO annotation frequency with respect to genotypes.

Figure S86: The cohort comprised 33 individuals (3 females, 30 males). 1 of these individuals were reported to be deceased. A total of 78 HPO terms were used to annotate the cohort. Disease diagnosis: Intellectual developmental disorder, X-linked 112 (OMIM:301111). A total of 22 unique variant alleles were found in *ZMYM3* (transcript: `NM_201599.3`, protein id: `NP_963893.1`). No analysis of *ZMYM3* genotype phgenotype correlation was presented in the two studies on *ZMYM3* variants study to be published to date [72, 73].

ZNF462

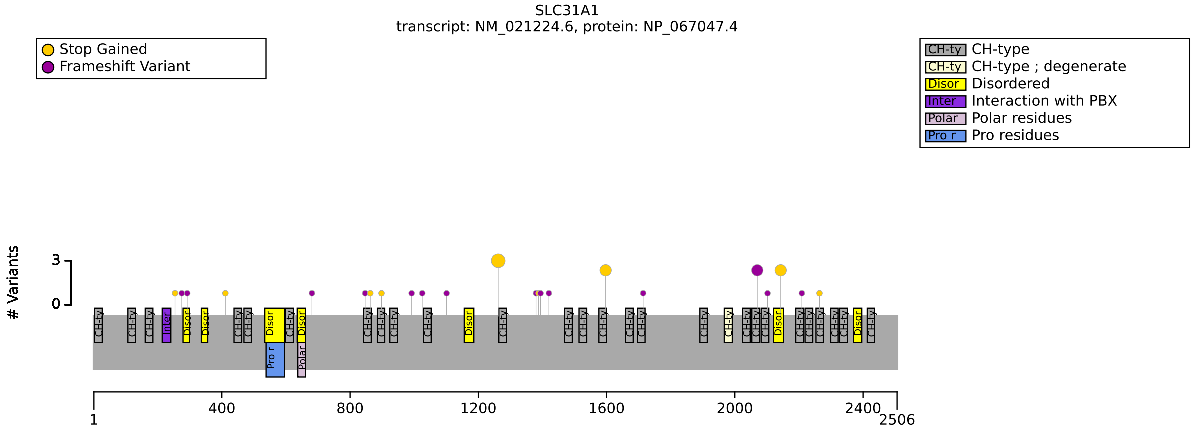

(a) Distribution of variants in *ZNF462*

Genotype (A)	Genotype (B)	total tests performed	significant results
Ablation	Other	43	0
FEMALE	MALE	41	0

(b) Fisher Exact Test performed to compare HPO annotation frequency with respect to genotypes.

Figure S87: The cohort comprised 39 individuals (11 females, 25 males, 3 with unknown sex). A total of 32 HPO terms were used to annotate the cohort. Disease diagnosis: Weiss-Kruszka syndrome (OMIM:618619). No statistically significant genotype phenotype association was identified. A total of 26 unique variant alleles were found in *ZNF462* (transcript: `NM_021224.6`, protein id: `NP_067047.4`).

ZSWIM6

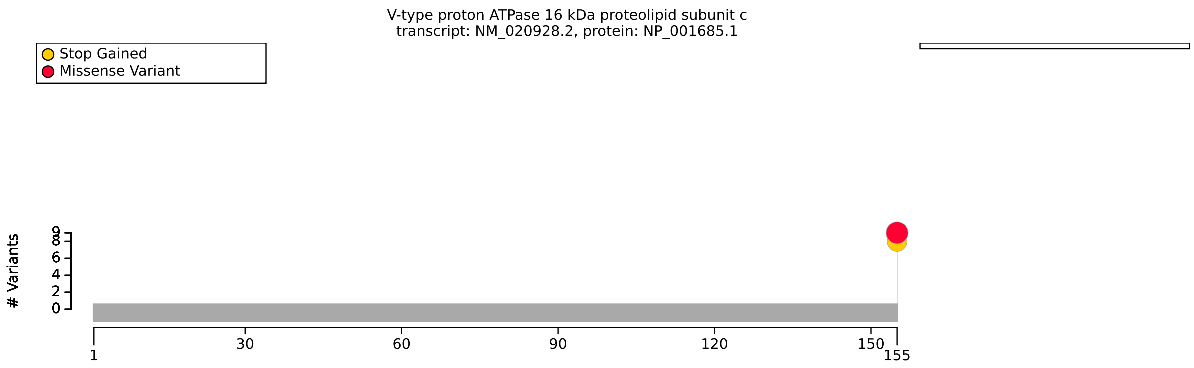

(a) Distribution of variants in *ZSWIM6*

Genotype (A)	Genotype (B)	total tests performed	significant results
p.Arg913Ter	Arg1163Trp	12	0

(b) Fisher Exact Test performed to compare HPO annotation frequency with respect to genotypes.

Figure S88: The cohort comprised 16 individuals (8 females, 8 males). A total of 85 HPO terms were used to annotate the cohort. Disease diagnoses: Acromelic frontonasal dysostosis (OMIM:603671) (9 individuals), Neurodevelopmental disorder with movement abnormalities, abnormal gait, and autistic features (OMIM:617865) (7 individuals). No statistically significant results identified. A total of 2 unique variant alleles were found in *ZSWIM6* (transcript: NM_020928.2, protein id: NP_001685.1).

Name	Match criterion	Example
variant key	Specific variant	1_8364773_8364773_A_AC (NM_001042681.2:c.1512dup)
variant effect	Leads to the target effect	START_LOST
variant class	Variant has the target type	SNV, DEL, DUP, INS, INV
gene	Variant affects gene	Affects *RERE*
transcript	Variant affects transcript	Overlaps NM_12345.01
exon	Variant overlaps exon	Located in exon 3
region	Variant overlaps region on protein	Located between amino acid residues 1 to 77
is large imprecise sv	Variant is structural variant without exact breakpoint coordinates	–
is structural variant	The variant affects at least n base pairs (n=50 by default) or is a large imprecise SV or a translocation	–
structural type	The target ontology class for a variant with imprecise/unknown breakpoints	Chromosomal deletion (SO:1000029)
is structural deletion	Variant is a Chromosomal deletion (SO:1000029) or an SV with known breakpoints that deletes at least a n base pairs (`n=50bp` by default)	–
ref length	Length of the reference sequence is above, below, or (not) equal to n bases	$n > 50$ for variants affecting more than 50 bp
change length	Change of length between the REF/ALT is above, below, or (not) equal to `n` bases	$n \leq -50$ for testing if a variant removes at least 50 base pairs
protein feature type	Variant affects protein feature type	DOMAIN
protein feature	Variant region overlaps a protein feature	Overlaps with "Bipartite nuclear localization signal"
allof	Logical AND for two or more predicates to test if *all* predicates match	missense AND exon 7
anyof	Logical OR for two or more predicates to test if *any* predicate matches	START_LOST OR STOP_GAINED
not	Logical NOT	NOT(missense)

Table S1: Variant predicates. Version 0.9.11 of GPSEA offers the variant predicates shown in this table. Each predicate evaluates a variant and returns `True` or `False`.

**cohort**	**genotype (A)**	**genotype (B)**	**Scorer**	**p-val**	**xrefs**
ANKRD11	FEMALE	MALE	HPO Group Count	0.007	-
ANKRD11	SV	C	HPO Group Count	2.64×10^{-4}	[3]
CHD8	FEMALE	MALE	De Vries Score	0.006	-
CHD8	missense	C	De Vries Score	9.0×10^{-4}	-
CTCF	missense	C	De Vries Score	0.009	-
LMNA	Upstream of NLS	C	HPO Group Count	1.83×10^{-16}	-
RERE	LoF	Atrophin	HPO Group Count	1.0×10^{-3}	-

Table S3: Phenotype severity scores. Mann-Whitney U tests performed using GPSEA to assess the association between a genotype and the total value of a phenotype severity score. The references in the xrefs column show previous publications that have presented similar findings. HPO Group Count scorer assigns a phenotype score that is equivalent to the count of present phenotypes that are either an exact match to the query terms or their descendants. DeVries scorer is an adaption of the DeVries score [57] using HPO. C: set complement of a variant predicate.

cohort	genotype (A)	genotype (B)	Outcome Variable	p-val	xrefs
ACADM	K329Q: 1/1	$1/\complement$ OR $\complement/\complement$	MCAD Activity% [LOINC:74892-1]	6.1×10^{-10}	[1]
ACADM	Y67H: 1/1 OR $1/\complement$	$\complement/\complement$	MCAD Activity% [LOINC:74892-1]	2.0×10^{-5}	[1]
CYP21A2	missense: $1/\complement$ OR $\complement/\complement$	1/1	17-Hydroxyprogesterone [LOINC:1668-3]	7.9×10^{-6}	-

Table S4: Student t-tests performed using GPSEA. $\complement$: set complement of a variant predicate. Citations in the xrefs column show previous publications that have presented similar findings.

cohort	genotype (A)	genotype (B)	HPO Term	p-val	xrefs
AIRE	R257*/R257* OR R257*/C	C/C	Survival analysis: Chronic mucocutaneous candidiasis	0.019	-
CLDN16	missense/missense OR missense/C	C/C	Survival analysis: Stage 5 chronic kidney disease	0.034	-
UMOD	Cys248Trp	Gln316Pro	Survival analysis: Stage 5 chronic kidney disease	4.1×10^{-4}	-

Table S5: Age of onset of phenotypic abnormality. Logrank tests performed using GPSEA to assess association between a genotype and the age of onset of a phenotypic feature represented by an HPO term. C: set complement of a variant predicate. Citations in the xrefs column show previous publications that have presented similar findings.

cohort	genotype (A)	genotype (B)	Disease onset	p-val	xrefs
CNTNAP2	1 allele	2 alleles	Compute time until OMIM:610042 onset	6.2×10^{-6}	-
FBXL4	missense/$\complement$ OR $\complement$/$\complement$	missense/missense	Compute time until OMIM:615471 onset	0.031	-
HMGCS2	missense/missense OR missense/$\complement$	$\complement$/$\complement$	Compute time until OMIM:605911 onset	0.038	-
MPV17	missense/missense OR missense/$\complement$	$\complement$/$\complement$	Compute time until OMIM:256810 onset	0.002	-
SETD2	Missense	$\complement$	Compute time until OMIM:616831 onset	8.5×10^{-5}	-
SUOX	Missense/Missense OR Missense/$\complement$	$\complement$/$\complement$	Compute time until OMIM:272300 onset	9.2×10^{-6}	-

Table S6: Age of onset of disease. Log rank tests performed using GPSEA to assess association between a genotype and the age of onset of a disease. $\complement$: set complement of a variant predicate. Citations in the xrefs column show previous publications that have presented similar findings. `hdim`: homodimerization; `OMIM:610042`: Pitt-Hopkins like syndrome 1; `OMIM:615471`: Mitochondrial DNA depletion syndrome 13 (encephalomyopathic type); `OMIM:605911`: HMG-CoA synthase-2 deficiency; `OMIM:256810`: Mitochondrial DNA depletion syndrome 6 (hepatocerebral type); `OMIM:616831`: Luscan-Lumish syndrome; `OMIM:272300`: Sulfite oxidase deficiency.

cohort	genotype (A)	genotype (B)	Disease	p-val	xrefs
MPV17	Pro98Leu: 1/1	Pro98Leu: 1/C or C/C	OMIM:256810	0.010	-

Table S7: Age of death. Log rank tests performed using GPSEA to assess association between a genotype and the age of death of individuals with a disease. C: set complement of a variant predicate. Citations in the xrefs column show previous publications that have presented similar findings. OMIM:256810: Mitochondrial DNA depletion syndrome 6 (hepatocerebral type).

Cohort	HPO	disease A		disease B		p-val	adj. p
ATP13A2	Bradykinesia [HP:0002067]	OMIM:606693	30/32 (94%)	OMIM:617225	4/10 (40%)	9.2×10^{-4}	0.013
ATP13A2	Parkinsonism [HP:0001300]	OMIM:606693	28/28 (100%)	OMIM:617225	3/11 (27%)	2.7×10^{-6}	7.8×10^{-5}
Kabuki	Feeding difficulties [HP:0011968]	OMIM:147920	8/25 (32%)	OMIM:300867	55/63 (87%)	7.4×10^{-7}	2.5×10^{-5}
Kabuki	Motor delay [HP:0001270]	OMIM:147920	4/10 (40%)	OMIM:300867	58/61 (95%)	1.0×10^{-4}	0.002
LDS 1 and 3	Aortic aneurysm [HP:0004942]	OMIM:609192	11/11 (100%)	OMIM:613795	26/48 (54%)	0.004	0.027
LDS 1 and 3	Hypertelorism [HP:0000316]	OMIM:609192	15/19 (79%)	OMIM:613795	13/35 (37%)	0.004	0.027
LDS 1 and 3	Osteoarthritis [HP:0002758]	OMIM:609192	0/11 (0%)	OMIM:613795	26/38 (68%)	4.6×10^{-5}	1.0×10^{-3}
LDS 1 and 3	Scoliosis [HP:0002650]	OMIM:609192	18/21 (86%)	OMIM:613795	20/43 (47%)	0.003	0.027
LDS 3 and 6	Thoracic aortic aneurysm [HP:0012727]	OMIM:613795	0/22 (0%)	OMIM:619656	10/16 (62%)	1.7×10^{-5}	4.2×10^{-4}
RPGRIP1	Nystagmus [HP:0000639]	OMIM:613826	64/66 (97%)	OMIM:608194	11/16 (69%)	0.003	0.020
RPGRIP1	Very low visual acuity [HP:0032122]	OMIM:613826	35/39 (90%)	OMIM:608194	4/16 (25%)	5.2×10^{-6}	7.8×10^{-5}
Robinow	Cleft palate [HP:0000175]	OMIM:268310	0/17 (0%)	OMIM:616331	5/8 (62%)	1.0×10^{-3}	0.031
Robinow	Hearing impairment [HP:0000365]	OMIM:268310	3/22 (14%)	OMIM:616331	7/7 (100%)	7.7×10^{-5}	0.003
Robinow	Mesomelia [HP:0003027]	OMIM:268310	31/31 (100%)	OMIM:616331	10/15 (67%)	0.002	0.048
Robinow	Short stature [HP:0004322]	OMIM:268310	29/29 (100%)	OMIM:616331	3/11 (27%)	2.2×10^{-6}	1.9×10^{-4}

Table S8: Fischer exact test for association between disease diagnosis and phenotypic features. `OMIM:606693`: Kufor-Rakeb syndrome (KRS); `OMIM:617225`: Spastic paraplegia 78, autosomal recessive (SPG78); `OMIM:147920`: Kabuki syndrome 1 (KABUK1); `OMIM:300867`: Kabuki syndrome 2 (KABUK2); `OMIM:609192`: Loeys-Dietz syndrome 1 (LDS1); `OMIM:613795`: Loeys-Dietz syndrome 3 (LDS3); `OMIM:619656`: Loeys-Dietz syndrome 6 (LDS6); `OMIM:613826`: Leber congenital amaurosis 6 (LCA6); `OMIM:608194`: Cone-rod dystrophy 13 (CORD13); `OMIM:268310`: Robinow syndrome, autosomal recessive 1 (RRS1); `OMIM:616331`: Robinow syndrome, autosomal dominant 2 (DRS2).

cohort	HPO	genotype (A)	Counts (A)	genotype (B)	Counts (B)	p-val	adj. p
KDM6A	Intellectual disability, severe [HP:0010864]	FEMALE	7/25 (28%)	MALE	14/18 (78%)	0.002	0.008

Table S9: Fischer exact test for association between phenotypic features and sex (male, female). Adj. p: p value adjusted with Benjamini-Hochberg method.

Table S10: Detailed results for Table 1 of the main manuscript. Sig: Number of significant associations. **Pub**: Number of these identified in the literature. **Categorical**: Association of genotypes with phenotypes by Fisher exact test. **t test**: Test of means of continuous values by Student t test. **HPO**: Logrank test for association of genotypes with age of onset of a phenotypic abnormality represented by an HPO term. **Disease**: Logrank test for association of genotypes with age of onset of a disease. **Mortality**: Logrank test for association of genotypes with age of death. **Phenotype score**: Mann Whitney U test for association of genotypes with magnitude of a phenotype severity score. Cohorts with no found significant associations are not reported. **Disease diagnosis**: Comparison of two or more diseases associated with the variants in the same gene. **Sex differences**: Comparison of frequencies of phenotypic features in a disease between males and females.

Cohort	Sig	Pub	Categorical	t test	HPO	Disease onset	Mortality	Phenotype score	Disease diagnosis	Sex differences
ACADM	2	2	-	2/2	-	-	-	-	-	-
AIRE	1	1	-	-	1/1	-	-	-	-	-
ANKRD11	2	1	-	-	-	-	-	1/2	-	-
ATP13A2	2	2	-	-	-	-	-	-	2/2	-
CHD8	2	1	-	-	-	-	-	1/2	-	-
CLDN16	1	1	-	-	1/1	-	-	-	-	-
CNTNAP2	1	0	-	-	-	0/1	-	-	-	-
CTCF	1	0	-	-	-	-	-	0/1	-	-
CYP21A2	1	1	-	1/1	-	-	-	-	-	-
EHMT1	1	1	1/1	-	-	-	-	-	-	-
FBN1	14	14	14/14	-	-	-	-	-	-	-
FBXL4	2	0	0/1	-	-	0/1	-	-	-	-
FGD1	1	1	1/1	-	-	-	-	-	-	-
GLI3	14	14	14/14	-	-	-	-	-	-	-
HMGCS2	1	0	-	-	-	0/1	-	-	-	-
IKZF1	2	2	2/2	-	-	-	-	-	-	-
ITPR1	21	0	0/21	-	-	-	-	-	-	-
Kabuki	2	0	-	-	-	-	-	-	0/2	-
KCNH5	1	1	1/1	-	-	-	-	-	-	-
KDM6A	2	1	1/1	-	-	-	-	-	-	0/1
LDS 1 and 3	4	0	-	-	-	-	-	-	0/4	-
LDS 3 and 6	1	0	-	-	-	-	-	-	0/1	-
LMNA	36	36	35/35	-	-	-	-	1/1	-	-

Continued on next page

Table S10 – continued from previous page

Cohort	Sig	Pub	Categorical	t test	HPO	Disease onset	Mortality	Phenotype score	Disease diagnosis	Sex differences
MPV17	3	1	0/1	-	-	0/1	1/1	-	-	-
NBAS	1	0	0/1	-	-	-	-	-	-	-
NF1	37	37	37/37	-	-	-	-	-	-	-
PTPN11	4	0	0/4	-	-	-	-	-	-	-
RERE	1	1	-	-	-	-	-	1/1	-	-
Robinow	4	0	-	-	-	-	-	-	0/4	-
RPGRIP1	3	1	1/1	-	-	-	-	-	0/2	-
SAMD9L	3	0	0/3	-	-	-	-	-	-	-
SATB2	1	0	0/1	-	-	-	-	-	-	-
SCN2A	9	9	9/9	-	-	-	-	-	-	-
SCO2	1	0	0/1	-	-	-	-	-	-	-
SETD2	9	8	8/8	-	-	0/1	-	-	-	-
SMAD3	1	0	0/1	-	-	-	-	-	-	-
SMARCB1	6	0	0/6	-	-	-	-	-	-	-
SMARCC2	1	1	1/1	-	-	-	-	-	-	-
SPTAN1	35	35	35/35	-	-	-	-	-	-	-
SUOX	2	1	1/1	-	-	0/1	-	-	-	-
TBCK	2	0	0/2	-	-	-	-	-	-	-
TBX1	2	0	0/2	-	-	-	-	-	-	-
TBX5	3	3	3/3	-	-	-	-	-	-	-
TGFBR1	4	1	1/4	-	-	-	-	-	-	-
UMOD	3	3	2/2	-	1/1	-	-	-	-	-
WWOX	1	1	1/1	-	-	-	-	-	-	-
ZFX	1	1	1/1	-	-	-	-	-	-	-
ZMYM3	1	0	0/1	-	-	-	-	-	-	-

HPO	id	Count	Observed (%)	Expected (%)
Abnormality of the musculoskeletal system	HP:0033127	38	21.8%	17.9%
Abnormality of limbs	HP:0040064	19	10.9%	11.4%
Abnormality of the nervous system	HP:0000707	40	23.0%	10.7%
Abnormality of metabolism/homeostasis	HP:0001939	2	1.1%	9.5%
Abnormality of the genitourinary system	HP:0000119	0	0.0%	6.5%
Abnormality of the cardiovascular system	HP:0001626	11	6.3%	5.7%
Abnormality of head or neck	HP:0000152	8	4.6%	5.6%
Abnormality of the immune system	HP:0002715	5	2.9%	5.3%
Abnormality of the eye	HP:0000478	6	3.4%	4.6%
Abnormality of the integument	HP:0001574	10	5.7%	4.2%
Abnormality of blood and blood-forming tissues	HP:0001871	6	3.4%	3.6%
Abnormality of the digestive system	HP:0025031	3	1.7%	3.3%
Neoplasm	HP:0002664	16	9.2%	2.7%
Abnormality of the respiratory system	HP:0002086	1	0.6%	2.5%
Abnormality of the endocrine system	HP:0000818	1	0.6%	1.8%
Abnormal cellular phenotype	HP:0025354	1	0.6%	1.2%
Abnormality of the ear	HP:0000598	2	1.1%	1.2%
Abnormality of prenatal development or birth	HP:0001197	0	0.0%	0.9%
Constitutional symptom	HP:0025142	0	0.0%	0.6%
Growth abnormality	HP:0001507	5	2.9%	0.4%
Abnormality of the breast	HP:0000769	0	0.0%	0.2%
Abnormality of the voice	HP:0001608	0	0.0%	0.1%
Abnormality of the thoracic cavity	HP:0045027	0	0.0%	0.0%

Table S11: Distribution of significant Fisher exact test results according to top-level HPO term

References

[1] S. Tucci, C. Wagner, S. C. Grünert, U. Matysiak, N. Weinhold, J. Klein, F. Porta, M. Spada, A. Bordugo, G. Rodella, et al. (2021). Genotype and residual enzyme activity in medium-chain acyl-CoA dehydrogenase (MCAD) deficiency: Are predictions possible? en. J. Inherit. Metab. Dis. *44*, 916–925.

[2] M. Halonen, P. Eskelin, A.-G. Myhre, J. Perheentupa, E. S. Husebye, O. Kämpe, F. Rorsman, L. Peltonen, I. Ulmanen, and J. Partanen (2002). AIRE mutations and human leukocyte antigen genotypes as determinants of the autoimmune polyendocrinopathy-candidiasis-ectodermal dystrophy phenotype. en. J. Clin. Endocrinol. Metab. *87*, 2568–2574.

[3] E. Martinez-Cayuelas, F. Blanco-Kelly, F. Lopez-Grondona, S. T. Swafiri, R. Lopez-Rodriguez, R. Losada-Del Pozo, I. Mahillo-Fernandez, B. Moreno, M. Rodrigo-Moreno, D. Casas-Alba, et al. (2023). Clinical description, molecular delineation and genotype-phenotype correlation in 340 patients with KBG syndrome: addition of 67 new patients. en. J. Med. Genet. *60*, 644–654.

[4] Z. Awamleh, S. Choufani, C. Cytrynbaum, F. S. Alkuraya, S. Scherer, S. Fernandes, C. Rosas, P. Louro, P. Dias, M. T. Neves, et al. (2023). ANKRD11 pathogenic variants and 16q24.3 microdeletions share an altered DNA methylation signature in patients with KBG syndrome. Human molecular genetics, *32*, 1429–1438. `https://doi.org/10.1093/hmg/ddac289`.

[5] A. J. M. Dingemans, K. M. G. Truijen, S. van de Ven, R. Bernier, E. M. H. F. Bongers, A. Bouman, L. de Graaff-Herder, E. E. Eichler, E. H. Gerkes, C. M. De Geus, et al. (2022). The phenotypic spectrum and genotype-phenotype correlations in 106 patients with variants in major autism gene CHD8. en. Transl. Psychiatry, *12*, 421.

[6] M. Konrad, J. Hou, S. Weber, J. Dötsch, J. A. Kari, T. Seeman, E. Kuwertz-Bröking, A. Peco-Antic, V. Tasic, K. Dittrich, et al. (2008). CLDN16 genotype predicts renal decline in familial hypomagnesemia with hypercalciuria and nephrocalcinosis. en. J. Am. Soc. Nephrol. *19*, 171–181.

[7] G. D'Onofrio, A. Accogli, M. Severino, H. Caliskan, T. Kokotović, A. Blazekovic, K. G. Jercic, S. Markovic, T. Zigman, K. Goran, et al. (2023). Genotype-phenotype correlation in contactin-associated protein-like 2 (CNTNAP-2) developmental disorder. en. Hum. Genet. *142*, 909–925.

[8] J. Xie, J. Jiang, and Q. Guo (2021). Primary Coenzyme Q10 Deficiency-7 and Pathogenic COQ4 Variants: Clinical Presentation, Biochemical Analyses, and Treatment. Frontiers in genetics, *12*, 776807. `https://doi.org/10.3389/fgene.2021.776807`.

[9] H. G. Valverde de Morales, H.-L. V. Wang, K. Garber, X. Cheng, V. G. Corces, and H. Li (2023). Expansion of the genotypic and phenotypic spectrum of CTCF-related disorder guides clinical management: 43 new subjects and a comprehensive literature review. en. Am. J. Med. Genet. A, *191*, 718–729.

[10] C. Xu, W. Jia, X. Cheng, H. Ying, J. Chen, J. Xu, Q. Guan, X. Zhou, D. Zheng, G. Li, et al. (2019). Genotype-phenotype correlation study and mutational and hormonal analysis in a Chinese cohort with 21-hydroxylase deficiency. Molecular genetics and genomic medicine, *7*, e671. https://doi.org/10.1002/mgg3.671.

[11] D. Rots, A. Bouman, A. Yamada, M. Levy, A. J. M. Dingemans, B. B. A. de Vries, M. Ruiterkamp-Versteeg, N. de Leeuw, C. W. Ockeloen, R. Pfundt, et al. (2024). Comprehensive EHMT1 variants analysis broadens genotype-phenotype associations and molecular mechanisms in Kleefstra syndrome. American journal of human genetics, *111*, 1605–1625. https://doi.org/10.1016/j.ajhg.2024.06.008.

[12] Z. J. Frazier, S. Kilic, H. Osika, A. Mo, M. Quinn, S. Ballal, T. Katz, A. E. Shearer, M. A. Horlbeck, L. S. Pais, et al. (2025). Novel Phenotypes and Genotype-Phenotype Correlations in a Large Clinical Cohort of Patients With Kleefstra Syndrome. Clinical genetics. https://doi.org/10.1111/cge.14697.

[13] C. Gracia-Diaz, Y. Zhou, Q. Yang, R. Maroofian, P. Espana-Bonilla, C.-H. Lee, S. Zhang, N. Padilla, R. Fueyo, E. A. Waxman, et al. (2023). Gain and loss of function variants in EZH1 disrupt neurogenesis and cause dominant and recessive neurodevelopmental disorders. en. Nat. Commun. *14*, 4109.

[14] V. M. Becerra-Muñoz, J. J. Gómez-Doblas, C. Porras-Martín, M. Such-Martínez, M. G. Crespo-Leiro, R. Barriales-Villa, E. de Teresa-Galván, M. Jiménez-Navarro, and F. Cabrera-Bueno (2018). The importance of genotype-phenotype correlation in the clinical management of Marfan syndrome. Orphanet journal of rare diseases, *13*, 16. https://doi.org/10.1186/s13023-017-0754-6.

[15] P. Arnaud, O. Milleron, N. Hanna, J. Ropers, N. Ould Ouali, A. Affoune, M. Langeois, L. Eliahou, F. Arnoult, P. Renard, et al. (2021). Clinical relevance of genotype-phenotype correlations beyond vascular events in a cohort study of 1500 Marfan syndrome patients with FBN1 pathogenic variants. Genetics in medicine : official journal of the American College of Medical Genetics, *23*, 1296–1304. https://doi.org/10.1038/s41436-021-01132-x.

[16] A. Hernándiz, A. Zúñiga, F. Valera, D. Domingo, I. Ontoria-Oviedo, J. F. Marí, J. A. Román, I. Calvo, B. Insa, R. Gómez, et al. (2021). Genotype FBN1/phenotype relationship in a cohort of patients with Marfan syndrome. Clinical genetics, *99*, 269–280. https://doi.org/10.1111/cge.13879.

[17] K. A. Groth, Y. Von Kodolitsch, K. Kutsche, M. Gaustadnes, K. Thorsen, N. H. Andersen, and C. H. Gravholt (2017). Evaluating the quality of Marfan genotype-phenotype correlations in existing FBN1 databases. Genetics in medicine : official journal of the American College of Medical Genetics, *19*, 772–777. https://doi.org/10.1038/gim.2016.181.

[18] A. W. El-Hattab, H. Dai, M. Almannai, J. Wang, E. A. Faqeih, A. Al Asmari, M. A. M. Saleh, M. A. O. Elamin, M. Alfadhel, F. S. Alkuraya, et al. (2017). Molecular and clinical spectra of FBXL4 deficiency. en. Hum. Mutat. *38*, 1649–1659.

[19] S. Li, A. Tian, Y. Wen, W. Gu, W. Li, X. Qiao, C. Zhang, and X. Luo (2024). FGD1-related Aarskog-Scott syndrome: Identification of four novel variations and a literature review of clinical and molecular aspects. European journal of pediatrics, *183*, 2257–2272. https://doi.org/10.1007/s00431-024-05484-9.

[20] V. Zanetti Drumond, L. Sousa Salgado, C. Sousa Salgado, V. A. d. L. Oliveira, E. M. de Assis, M. Campos Ribeiro, A. Furtado Valadão, and A. Orrico (2021). The Prevalence of Clinical Features in Patients with Aarskog-Scott Syndrome and Assessment of Genotype-Phenotype Correlation: A Systematic Review. Genetics research, *2021*, 6652957. https://doi.org/10.1155/2021/6652957.

[21] J. J. Johnston, I. Olivos-Glander, C. Killoran, E. Elson, J. T. Turner, K. F. Peters, M. H. Abbott, D. J. Aughton, A. S. Aylsworth, M. J. Bamshad, et al. (2005). Molecular and clinical analyses of Greig cephalopolysyndactyly and Pallister-Hall syndromes: robust phenotype prediction from the type and position of GLI3 mutations. American journal of human genetics, *76*, 609–622. https://doi.org/10.1086/429346.

[22] A. Jamsheer, A. Sowińska, T. Trzeciak, M. Jamsheer-Bratkowska, A. Geppert, and A. Latos-Bieleńska (2012). Expanded mutational spectrum of the GLI3 gene substantiates genotype-phenotype correlations. Journal of applied genetics, *53*, 415–422. https://doi.org/10.1007/s13353-012-0109-x.

[23] R. Patel, S. K. Singh, V. Bhattacharya, and A. Ali (2021). Novel GLI3 pathogenic variants in complex pre- and postaxial polysyndactyly and Greig cephalopolysyndactyly syndrome. American journal of medical genetics. Part A, *185*, 97–104. https://doi.org/10.1002/ajmg.a.61919.

[24] F. Démurger, A. Ichkou, S. Mougou-Zerelli, M. Le Merrer, G. Goudefroye, A.-L. Delezoide, C. Quélin, S. Manouvrier, G. Baujat, M. Fradin, et al. (2015). New insights into genotype-phenotype correlation for GLI3 mutations. European journal of human genetics : EJHG, *23*, 92–102. https://doi.org/10.1038/ejhg.2014.62.

[25] L. G. Biesecker (1997). Strike three for GLI3. Nature genetics, *17*, 259–260. https://doi.org/10.1038/ng1197-259.

[26] M. M. Al-Qattan, H. E. Shamseldin, M. A. Salih, and F. S. Alkuraya (2017). GLI3-related polydactyly: a review. Clinical genetics, *92*, 457–466. https://doi.org/10.1111/cge.12952.

[27] H. L. Sczakiel, W. Hülsemann, M. Holtgrewe, A. T. Abad-Perez, J. Elsner, S. Schwartzmann, D. Horn, M. Spielmann, S. Mundlos, and M. A. Mensah (2021). GLI3 variants causing isolated polysyndactyly are not restricted to the protein's C-terminal third. Clinical genetics, *100*, 758–765. https://doi.org/10.1111/cge.14059.

[28] D. Boutboul, H. S. Kuehn, Z. Van de Wyngaert, J. E. Niemela, I. Callebaut, J. Stoddard, C. Lenoir, V. Barlogis, C. Farnarier, F. Vely, et al. (2018). Dominant-negative IKZF1 mutations cause a T, B, and myeloid cell combined immunodeficiency. The Journal of clinical investigation, *128*, 3071–3087. https://doi.org/10.1172/JCI98164.

[29] M. P. Adam, S. Banka, H. T. Bjornsson, O. Bodamer, A. E. Chudley, J. Harris, H. Kawame, B. C. Lanpher, A. W. Lindsley, G. Merla, et al. (2019). Kabuki syndrome: international consensus diagnostic criteria. Journal of medical genetics, *56*, 89–95. https://doi.org/10.1136/jmedgenet-2018-105625.

[30] P. Makrythanasis, B. W. van Bon, M. Steehouwer, B. Rodríguez-Santiago, M. Simpson, P. Dias, B. M. Anderlid, P. Arts, M. Bhat, B. Augello, et al. (2013). MLL2 mutation detection in 86 patients with Kabuki syndrome: a genotype-phenotype study. Clinical genetics, *84*, 539–545. https://doi.org/10.1111/cge.12081.

[31] Y. Wang, Y. Xu, Y. Chen, Y. Hu, Q. Li, S. Liu, J. Wang, and X. Wang (2024). Sex-specific difference in phenotype of Kabuki syndrome type 2 patients: a matched case-control study. BMC pediatrics, *24*, 133. https://doi.org/10.1186/s12887-024-04562-z.

[32] H. C. Happ, L. G. Sadleir, M. Zemel, G. de Valles-Ibáñez, M. S. Hildebrand, A. McConkie-Rosell, M. McDonald, H. May, T. Sands, V. Aggarwal, et al. (2023). Neurodevelopmental and Epilepsy Phenotypes in Individuals With Missense Variants in the Voltage-Sensing and Pore Domains of KCNH5. Neurology, *100*, e603–e615. https://doi.org/10.1212/WNL.0000000000201492.

[33] V. Faundes, S. Goh, R. Akilapa, H. Bezuidenhout, H. T. Bjornsson, L. Bradley, A. F. Brady, E. Brischoux-Boucher, H. Brunner, S. Bulk, et al. (2021). Clinical delineation, sex differences, and genotype-phenotype correlation in pathogenic KDM6A variants causing X-linked Kabuki syndrome type 2. Genetics in medicine : official journal of the American College of Medical Genetics, *23*, 1202–1210. https://doi.org/10.1038/s41436-021-01119-8.

[34] E. S. Stolerman, E. Francisco, J. L. Stallworth, J. R. Jones, K. G. Monaghan, J. Keller-Ramey, R. Person, I. M. Wentzensen, K. McWalter, B. Keren, et al. (2019). Genetic variants in the KDM6B gene are associated with neurodevelopmental delays and dysmorphic features. American journal of medical genetics. Part A, *179*, 1276–1286. https://doi.org/10.1002/ajmg.a.61173.

[35] G. Captur, E. Arbustini, P. Syrris, D. Radenkovic, B. O'Brien, W. J. Mckenna, and J. C. Moon (2018). Lamin mutation location predicts cardiac phenotype severity: combined analysis of the published literature. Open heart, *5*, e000915. https://doi.org/10.1136/openhrt-2018-000915.

[36] E. W. Lin, G. F. Brady, R. Kwan, A. I. Nesvizhskii, and M. B. Omary (2020). Genotype-phenotype analysis of LMNA-related diseases predicts phenotype-selective alterations in lamin phosphorylation. FASEB journal : official publication of the Federation of American Societies for Experimental Biology, *34*, 9051–9073. https://doi.org/10.1096/fj.202000500R.

[37] A. W. El-Hattab, F.-Y. Li, E. Schmitt, S. Zhang, W. J. Craigen, and L.-J. C. Wong (2010). MPV17-associated hepatocerebral mitochondrial DNA depletion syndrome: new patients and novel mutations. Molecular genetics and metabolism, *99*, 300–308. https://doi.org/10.1016/j.ymgme.2009.10.003.

[38] M. P. Adam, J. Feldman, G. M. Mirzaa, R. A. Pagon, S. E. Wallace, and A. Amemiya (1993). GeneReviews (®).

[39] N. Hammann, D. Lenz, I. Baric, E. Crushell, C. D. Vici, F. Distelmaier, F. Feillet, P. Freisinger, M. Hempel, A. L. Khoreva, et al. (2024). Impact of genetic and non-genetic factors on phenotypic diversity in NBAS-associated disease. Molecular genetics and metabolism, *141*, 108118. https://doi.org/10.1016/j.ymgme.2023.108118.

[40] K. Rojnueangnit, J. Xie, A. Gomes, A. Sharp, T. Callens, Y. Chen, Y. Liu, M. Cochran, M.-A. Abbott, J. Atkin, et al. (2015). High Incidence of Noonan Syndrome Features Including Short Stature and Pulmonic Stenosis in Patients carrying NF1 Missense Mutations Affecting p.Arg1809: Genotype-Phenotype Correlation. Human mutation, *36*, 1052–1063. https://doi.org/10.1002/humu.22832.

[41] M. Upadhyaya, S. M. Huson, M. Davies, N. Thomas, N. Chuzhanova, S. Giovannini, D. G. Evans, E. Howard, B. Kerr, S. Griffiths, et al. (2007). An absence of cutaneous neurofibromas associated with a 3-bp inframe deletion in exon 17 of the NF1 gene (c.2970-2972 delAAT): evidence of a clinically significant NF1 genotype-phenotype correlation. American journal of human genetics, *80*, 140–151. https://doi.org/10.1086/510781.

[42] E. Pasmant, A. Sabbagh, G. Spurlock, I. Laurendeau, E. Grillo, M.-J. Hamel, L. Martin, S. Barbarot, B. Leheup, D. Rodriguez, et al. (2010). NF1 microdeletions in neurofibromatosis type 1: from genotype to phenotype. Human mutation, *31*, E1506–E1518. https://doi.org/10.1002/humu.21271.

[43] D. Nagy, S. Verheyen, K. M. Wigby, A. Borovikov, A. Sharkov, V. Slegesky, A. Larson, C. Fagerberg, C. Brasch-Andersen, M. Kibæk, et al. (2022). Genotype-Phenotype Comparison in POGZ-Related Neurodevelopmental Disorders by Using Clinical Scoring. Genes, *13*. https://doi.org/10.3390/genes13010154.

[44] V. K. Jordan, B. Fregeau, X. Ge, J. Giordano, R. J. Wapner, T. B. Balci, M. T. Carter, J. A. Bernat, A. N. Moccia, A. Srivastava, et al. (2018). Genotype-phenotype correlations in individuals with pathogenic RERE variants. Human mutation, *39*, 666–675. https://doi.org/10.1002/humu.23400.

[45] M. Daich Varela, M. Jeste, T. A. C. de Guimaraes, O. A. Mahroo, G. Arno, A. R. Webster, and M. Michaelides (2024). Clinical, Ophthalmic, and Genetic Characterization of RPGRIP1-Associated Leber Congenital Amaurosis/Early-Onset Severe Retinal Dystrophy. American journal of ophthalmology, *266*, 255–263. https://doi.org/10.1016/j.ajo.2024.05.007.

[46] C. D. Zingariello, D.-H. Chen, W. H. Raskind, W. B. Slayton, S. Subramony, J. Severance, M. Feagle, and S. A. Rasmussen (2024). Assessing Long-Term Neurologic Outcomes in SAMD9L-Related Ataxia-Pancytopenia Syndrome. Movement disorders clinical practice, *11*, 728–733. https://doi.org/10.1002/mdc3.14038.

[47] Y. A. Zarate, C. L. Smith-Hicks, C. Greene, M.-A. Abbott, V. M. Siu, A. R. U. L. Calhoun, A. Pandya, C. Li, E. A. Sellars, J. Kaylor, et al. (2018). Natural history and genotype-phenotype correlations in 72 individuals with SATB2-associated syndrome. American journal of medical genetics. Part A, *176*, 925–935. https://doi.org/10.1002/ajmg.a.38630.

[48] M. Wolff, A. Brunklaus, and S. M. Zuberi (2019). Phenotypic spectrum and genetics of SCN2A-related disorders, treatment options, and outcomes in epilepsy and beyond. Epilepsia, *60 Suppl 3*, S59–S67. https://doi.org/10.1111/epi.14935.

[49] K. Vesela, H. Hansikova, M. Tesarova, P. Martasek, M. Elleder, J. Houstek, and J. Zeman (2004). Clinical, biochemical and molecular analyses of six patients with isolated cytochrome c oxidase deficiency due to mutations in the SCO2 gene. Acta paediatrica (Oslo, Norway : 1992), *93*, 1312–1317. https://doi.org/10.1080/08035250410008761.

[50] E. Van Nieuwenhove, J. S. Barber, J. Neumann, E. Smeets, M. Willemsen, E. Pasciuto, T. Prezzemolo, V. Lagou, L. Seldeslachts, B. Malengier-Devlies, et al. (2020). Defective Sec61α1 underlies a novel cause of autosomal dominant severe congenital neutropenia. en. J. Allergy Clin. Immunol. *146*, 1180–1193.

[51] R. Rabin, A. Radmanesh, I. A. Glass, W. B. Dobyns, K. A. Aldinger, J. T. Shieh, S. Romoser, H. Bombei, L. Dowsett, P. Trapane, et al. (2020). Genotype-phenotype correlation at codon 1740 of SETD2. American journal of medical genetics. Part A, *182*, 2037–2048. https://doi.org/10.1002/ajmg.a.61724.

[52] Z. S. Ulhaq, G. V. Soraya, L. A. Istifiani, S. A. Pamungkas, and W. K. F. Tse (2023). SF3B4 frameshift variants represented a more severe clinical manifestation in Nager syndrome. en. Cleft Palate Craniofac. J. *60*, 1041–1047.

[53] A. Kurisaki, K. Kurisaki, M. Kowanetz, H. Sugino, Y. Yoneda, C.-H. Heldin, and A. Moustakas (2006). The mechanism of nuclear export of Smad3 involves exportin 4 and Ran. en. Mol. Cell. Biol. *26*, 1318–1332.

[54] B. Chesneau, T. Edouard, Y. Dulac, H. Colineaux, M. Langeois, N. Hanna, C. Boileau, P. Arnaud, N. Chassaing, S. Julia, et al. (2020). Clinical and genetic data of 22 new patients with SMAD3 pathogenic variants and review of the literature. en. Mol. Genet. Genomic Med. *8*, e1132.

[55] T. Kosho, N. Okamoto, and Coffin-Siris Syndrome International Collaborators (2014). Genotype-phenotype correlation of Coffin-Siris syndrome caused by mutations in SMARCB1, SMARCA4, SMARCE1, and ARID1A. en. Am. J. Med. Genet. C Semin. Med. Genet. *166C*, 262–275.

[56] E. Bosch, B. Popp, E. Güse, C. Skinner, P. J. van der Sluijs, I. Maystadt, A. M. Pinto, A. Renieri, L. P. Bruno, S. Granata, et al. (2023). Elucidating the clinical and molecular spectrum of SMARCC2-associated NDD in a cohort of 65 affected individuals. en. Genet. Med. *25*, 100950.

[57] A. J. M. Dingemans, K. M. G. Truijen, J.-H. Kim, Z. Alaçam, L. Faivre, K. M. Collins, E. H. Gerkes, M. van Haelst, I. M. B. H. van de Laar, K. Lindstrom, et al. (2022). Establishing the phenotypic spectrum of ZTTK syndrome by analysis of 52 individuals with variants in SON. en. Eur. J. Hum. Genet. *30*, 271–281.

[58] J. Tohyama, M. Nakashima, S. Nabatame, C. Gaik-Siew, R. Miyata, Z. Rener-Primec, M. Kato, N. Matsumoto, and H. Saitsu (2015). SPTAN1 encephalopathy: distinct phenotypes and genotypes. Journal of human genetics, *60*, 167–173. https://doi.org/10.1038/jhg.2015.5.

[59] H. Morsy, M. Benkirane, E. Cali, C. Rocca, K. Zhelcheska, V. Cipriani, E. Galanaki, R. Maroofian, S. Efthymiou, D. Murphy, et al. (2023). Expanding SPTAN1 monoallelic variant associated disorders: From epileptic encephalopathy to pure spastic paraplegia and ataxia. Genetics in medicine : official journal of the American College of Medical Genetics, *25*, 76–89. https://doi.org/10.1016/j.gim.2022.09.013.

[60] L. Van de Vondel, J. De Winter, D. Beijer, G. Coarelli, M. Wayand, R. Palvadeau, M. G. Pauly, K. Klein, M. Rautenberg, L. Guillot-Noël, et al. (2022). De Novo and Dominantly Inherited SPTAN1 Mutations Cause Spastic Paraplegia and Cerebellar Ataxia. Movement disorders : official journal of the Movement Disorder Society, *37*, 1175–1186. https://doi.org/10.1002/mds.28959.

[61] J.-T. Li, Z.-X. Chen, X.-J. Chen, and Y.-X. Jiang (2022). Mutation analysis of SUOX in isolated sulfite oxidase deficiency with ectopia lentis as the presenting feature: insights into genotype-phenotype correlation. Orphanet journal of rare diseases, *17*, 392. https://doi.org/10.1186/s13023-022-02544-x.

[62] E. L. Durham, R. Angireddy, A. Black, A. Melendez-Perez, S. Smith, E. M. Gonzalez, K. G. Navarro, A. Díaz, E. J. K. Bhoj, and K. A. Katsura (2023). TBCK syndrome: a rare multi-organ neurodegenerative disease. en. Trends Mol. Med. *29*, 783–785.

[63] C. Vanlerberghe, A.-S. Jourdain, J. Ghoumid, F. Frenois, A. Mezel, G. Vaksmann, B. Lenne, B. Delobel, N. Porchet, V. Cormier-Daire, et al. (2019). Holt-Oram syndrome: clinical and molecular description of 78 patients with TBX5 variants. en. Eur. J. Hum. Genet. *27*, 360–368.

[64] D. R. Goudie, M. D'Alessandro, B. Merriman, H. Lee, I. Szeverényi, S. Avery, B. D. O'Connor, S. F. Nelson, S. E. Coats, A. Stewart, et al. (2011). Multiple self-healing squamous epithelioma is caused by a disease-specific spectrum of mutations in TGFBR1. Nature genetics, *43*, 365–369. https://doi.org/10.1038/ng.780.

[65] J. L. Moskowitz, S. E. Piret, K. Lhotta, T. M. Kitzler, A. P. Tashman, E. Velez, R. V. Thakker, and P. Kotanko (2013). Association between genotype and phenotype in uromodulin-associated kidney disease. Clinical journal of the American Society of Nephrology : CJASN, *8*, 1349–1357. https://doi.org/10.2215/CJN.11151012.

[66] G. D. Smith, C. Robinson, A. P. Stewart, E. L. Edwards, H. I. Karet, A. G. W. Norden, R. N. Sandford, and F. E. Karet Frankl (2011). Characterization of a recurrent in-frame UMOD indel mutation causing late-onset autosomal dominant end-stage renal failure. Clinical journal of the American Society of Nephrology : CJASN, *6*, 2766–2774. https://doi.org/10.2215/CJN.06820711.

[67] J. Piard, L. Hawkes, M. Milh, L. Villard, R. Borgatti, R. Romaniello, M. Fradin, Y. Capri, D. Héron, M.-C. Nougues, et al. (2019). The phenotypic spectrum of WWOX-related disorders: 20 additional cases of WOREE syndrome and review of the literature. Genetics in medicine : official journal of the American College of Medical Genetics, *21*, 1308–1318. https://doi.org/10.1038/s41436-018-0339-3.

[68] M. Valduga, C. Philippe, L. Lambert, P. Bach-Segura, E. Schmitt, J. P. Masutti, B. François, P. Pinaud, M. Vibert, and P. Jonveaux (2015). WWOX and severe autosomal recessive epileptic encephalopathy: first case in the prenatal period. Journal of human genetics, *60*, 267–271. https://doi.org/10.1038/jhg.2015.17.

[69] C. Mignot, L. Lambert, L. Pasquier, T. Bienvenu, A. Delahaye-Duriez, B. Keren, J. Lefranc, A. Saunier, L. Allou, V. Roth, et al. (2015). WWOX-related encephalopathies: delineation of the phenotypical spectrum and emerging genotype-phenotype correlation. Journal of medical genetics, *52*, 61–70. https://doi.org/10.1136/jmedgenet-2014-102748.

[70] J. L. Shepherdson, K. Hutchison, D. W. Don, G. McGillivray, T.-I. Choi, C. A. Allan, D. J. Amor, S. Banka, D. G. Basel, L. D. Buch, et al. (2024). Variants in ZFX are associated with an X-linked neurodevelopmental disorder with recurrent facial gestalt. American journal of human genetics, *111*, 487–508. https://doi.org/10.1016/j.ajhg.2024.01.007.

[71] B. Guan, S. K. Agarwal, J. M. Welch, S. Jha, L. S. Weinstein, and W. F. Simonds (2024). A Germline ZFX Missense Variant in a Patient With Primary Hyperparathyroidism. JCEM case reports, *2*, luae115. https://doi.org/10.1210/jcemcr/luae115.

[72] A. K. Philips, A. Sirén, K. Avela, M. Somer, M. Peippo, M. Ahvenainen, F. Doagu, M. Arvio, H. Kääriäinen, H. Van Esch, et al. (2014). X-exome sequencing in Finnish families with intellectual disability–four novel mutations and two novel syndromic phenotypes. Orphanet journal of rare diseases, *9*, 49. https://doi.org/10.1186/1750-1172-9-49.

[73] S. M. Hiatt, S. Trajkova, M. R. Sebastiano, E. C. Partridge, F. E. Abidi, A. Anderson, M. Ansar, S. E. Antonarakis, A. Azadi, R. Bachmann-Gagescu, et al. (2023). Deleterious, protein-altering variants in the transcriptional coregulator ZMYM3 in 27 individuals with a neurodevelopmental delay phenotype. American journal of human genetics, *110*, 215–227. https://doi.org/10.1016/j.ajhg.2022.12.007.
